# *Z*‑Selective Synthesis of Trisubstituted Alkenes Bearing Allylic Tertiary Amines via Rhodium-Catalyzed Allylic Amination of 1,1-Disubstituted Fluoromethyl Trichloroacetimidates

**DOI:** 10.1021/jacsau.6c00604

**Published:** 2026-07-03

**Authors:** Felix O. Chukwu, Neha Rani, Rollanda Tella, H. Bernhard Schlegel, Hien M. Nguyen

**Affiliations:** Department of Chemistry, 2954 Wayne State University, Detroit, Michigan 48202, United States

**Keywords:** *Z*-trisubstituted alkene, allylic amination, rhodium, transition-metal catalysis, trichloroacetimidate, stereoselective amination, tertiary allylic electrophile

## Abstract

*Z*-trisubstituted alkenes serve as key pharmaceutical building blocks and essential feedstocks for the selective synthesis and modification of drug molecules. Among these compounds, *Z*-trisubstituted alkenes that contain allylic tertiary amines are significant due to the increased solubility, lipophilicity, and metabolic stability provided by the tertiary amines. However, controlled synthesis of *Z*-trisubstituted alkenes with allylic tertiary amines has proven difficult, primarily due to thermodynamic challenges associated with the *Z*-isomer and the synthetic difficulties of accessing allylic tertiary amines. Here, we report a diene ligand-controlled rhodium-catalyzed amination of 1,1-disubstituted fluoromethyl allylic trichloroacetimidates with secondary aliphatic and aryl amines to afford the *Z*-trisubstituted alkenes bearing allylic tertiary amines in good yields (45–98% yields) and excellent selectivity (*E/Z* > 1:20). A mechanistic investigation was conducted using both experimental and computational Density Functional Theory (DFT). DFT calculations showed a preference for bidentate coordination of the diene ligand to the rhodium, which is needed for the ionization of the 1,1-disubstituted fluoromethyl allylic trichloroacetimidate to generate *anti*- and *syn*-π-allyl-rhodium intermediates. The *E*- and *Z*-differentiation occurs prior to nucleophilic attack of the secondary amines onto the more reactive π-allyl intermediates. The *syn* π-allyl intermediates lead to the minor *E*-trisubstituted alkene product, whereas the more reactive *anti* intermediates give the major *Z*-product. DFT calculations showed that the rate of conversion between reactive *anti* and *syn π*-allyl intermediates via π–σ–π interconversion was faster than the rate of nucleophilic attack, establishing a Curtin–Hammett condition. The fluoromethyl substrate was essential for the linear regioselectivity, as it induced a crucial hydrogen bonding interaction between the 1,1-disubstituted fluoromethyl allylic trichloroacetimidate and the secondary amine nucleophile. Overall, we anticipate that this methodology will be broadly applicable to the development of medically relevant *Z*-alkenes bearing allylic tertiary amines. The mechanistic insights gained will also serve as a foundation for addressing the challenges involved in other classes of *Z*-configured alkenes.

## Graphical abstract

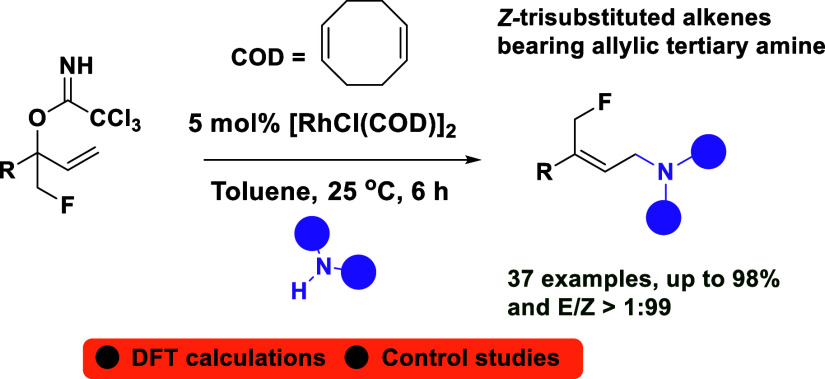

## Introduction

Trisubstituted alkenes are a highly attractive motif in pharmaceuticals, found in both natural and synthetic organic compounds.^1^ They also serve as versatile building blocks in synthetic processes, including conjugate addition,^2^ enantioselective hydrogenation,^3^ and cross-coupling.^4^ The stereoconfiguration and the substitution pattern of the alkene motif directly influence its pharmacological properties.^5^ In particular, the substitution pattern of trisubstituted alkenes featuring an allylic tertiary amine is particularly important for pharmaceutical applications. Besides the biological modulation afforded by *E*- or *Z*-trisubstituted alkene, access to the allylic tertiary amine is critical since tertiary amines are among the most prevalent candidates among drug compounds.^6,7^

Tertiary allylic amines are prevalent in small-molecule pharmaceuticals because of the physiological properties of the amine motif, which enables tuning of ADMET (absorption, distribution, metabolism, excretion, and toxicity).^8^ As a result, the stereoselective synthesis of trisubstituted alkenes containing the allylic tertiary amine is highly desirable (Figure 1A). However, the controlled synthesis of trisubstituted alkenes bearing the allylic tertiary amine presents significant challenges. Specifically, the selective construction of *Z*-trisubstituted motifs has proven difficult due to the challenges in controlling the stereochemistry of the thermodynamically unfavorable *Z*-isomer.^9,10,14–17^ This issue is further complicated in acyclic all-carbon trisubstituted alkenes, where increased steric interactions create additional obstacles.^11^ Additionally, the reliance on secondary amines to access allylic tertiary amines introduces further complications,^12,13^ making it a two-component problem. There are a few methodologies available for the stereoselective synthesis of *Z*-trisubstituted alkenes containing allylic amines. Notable approaches include Young’s palladium-catalyzed diarylation of allylamines, which enables the formation of *Z*-trisubstituted alkenes with allylic tertiary amines,^14^ and Kleij’s palladium-catalyzed amination of secondary amines with vinyl cyclic carbonates.^15^ However, these studies primarily examine access to secondary amines, with only a few examples of allylic tertiary amines.

**Figure 1:**
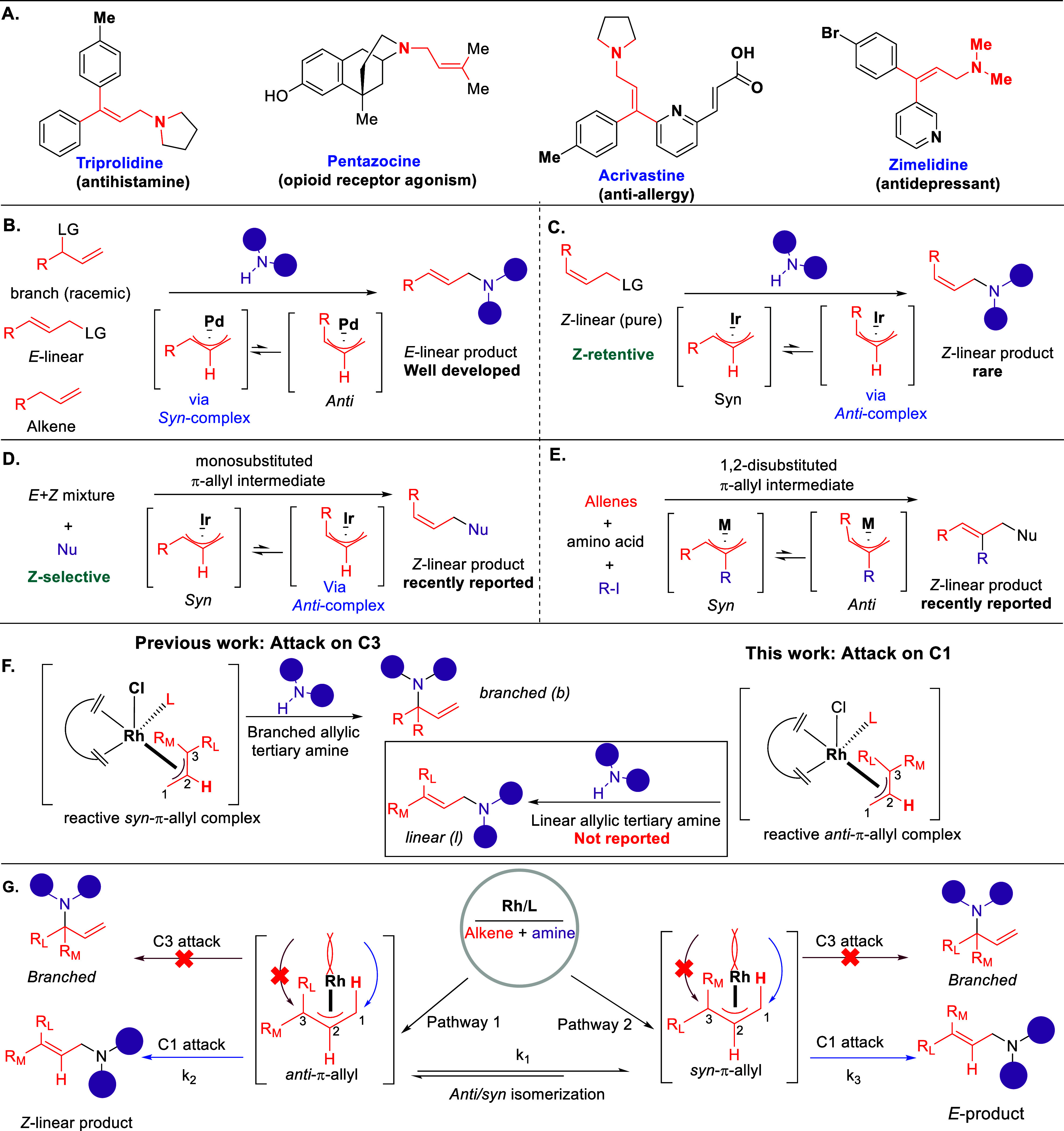
Background and the design of the trisubstituted alkene containing tertiary amines. (a) Examples of small organic molecules that contain trisubstituted alkenes with allylic tertiary amines. (b) Regioselective synthesis of *E*-disubstituted alkenes bearing allylic tertiary amines via a monosubstituted π-allylpalladium intermediate. (c) Regioselective synthesis of *Z*-disubstituted alkenes bearing allylic tertiary amines via a monosubstituted π-allyliridium intermediate. (d) *Z*-selective disubstituted alkenes via monosubstituted π-allyliridium intermediate. (e) Stereodivergent access to trisubstituted alkenes via 1,2-disubstituted π-allylrhodium intermediate. (f) Reaction development: *syn* complex (R_L_ and H are on the same side) and *anti* complex (R_L_ and H are on the opposite side) (g) Development of allylic amination of 1,1-disubstituted allyl intermediate via *syn-* and *anti-*π-allylrhodium intermediate reactive species.

Transition-metal-catalyzed allylic amination, which transforms allylic electrophiles or olefins into π-allyl-metal intermediates for C–N bond formation, has addressed some of the problems associated with accessing allylic tertiary amines (Figure 1B). Mechanistically, the generated π-allyl-metal intermediate exists in two distinct isomeric forms, namely the *syn* and *anti* π-allyl. The relative population of the different isomeric intermediates is influenced by steric interactions within each complex and is controlled by the rapid π–σ–π interconversion between the *syn* and *anti* complexes.^16–18^ The nucleophilic addition favors the more reactive thermodynamically stable *syn*-π-allyl-metal isomer, leading to the *E*-linear product from the monosubstituted π-allyl intermediates (Figure 1B). This thermodynamically driven *E*-allylic tertiary amine access from π-allyl-metal intermediates has been recently reported by White (olefin),^19,20^ Gevorgyan (allylic electrophiles),^21,22^ Jiang (allylic electrophiles),^23^ among others. In the same model, to access the thermodynamically unfavored *Z*-linear product, a *Z*-linear starting material is needed to generate the *anti*-π-allyl-metal complex (Figure 1C).^24^ The challenge in this approach lies in selectively enabling nucleophilic attack on the thermodynamically unfavorable intermediate. Studies in this area have focused on the *Z*-retentive access, either by trapping^25^ or interrupting the isomerization of the *anti*-isomer.^26^ The You group has reported *Z*-alkene synthesis via iridium-catalyzed allylic substitution reactions (Figure 1C).^27,28^ The *Z*-linear allylic product was formed by capturing the transient *anti*-π-allyl-iridium from the *Z*-allylic starting material before it isomerized to the stable *syn*-π-allyl intermediate. The dependence solely on a *Z*-allylic substrate was later addressed by using an *E/Z* mixture (Figure 1D), in which the *E*-allylic substrate was rapidly isomerized into the reactive *Z*-allylic substrate using a photosensitizer.^29^ Zhang and coworkers recently developed stereodivergent access to the trisubstituted alkene using ligand-controlled selectivity of a 1,2-disubstituted π-allyl intermediate (Figure 1E).^30^ Despite the significant recent breakthroughs for the *Z*-selective alkene synthesis from both the monosubstituted and the 1,2-disubstituted π-allyl intermediate, to the best of our knowledge, there are no reports on the transformation of the 1,1-disubstituted π-allyl intermediate for the selective formation of the *Z*-trisubstituted alkene using the transition-metal-catalyzed allylic substitution (Figure 1F). One primary reason for the challenges in this area is that existing studies on both the *Z*-selective and retentive pathways of monosubstituted π-allyl-metal intermediates have focused on preventing the rapid π–σ–π interconversion of the *syn*/*anti* intermediates. However, the synthesis^31,32^ and reactivity^33,34^ of the linear tertiary starting material to access the 1,1-disubstituted π-allyl intermediate present significant difficulties. In addition, the isomerization of the trisubstituted *E*/*Z* starting materials introduces even greater complications.^31^

To address this critical concern, we explored the possibility of accessing the *Z*-trisubstituted alkene generated from 1,1-disubstituted fluoromethyl allylic trichloroacetimidate. Our group has established the reactivity and selectivity of the 1,1-disubstituted π-allyl-rhodium intermediate from a tertiary allylic trichloroacetimidate starting material in asymmetric amination (Figure 1F).^35–37^ Notably, regioselective control between the C1 and C3 carbons is critical for achieving a linear allylic tertiary amine. Our previous studies have focused on C3 asymmetric amination. We therefore hypothesized that, by properly designing the electrophile and ligand on the rhodium catalyst, we could tune regioselectivity toward the C1 carbon to afford linear Z-trisubstituted alkenes bearing allylic tertiary amines. Previously, we reported that the fluoromethyl substrate stabilizes the 1,1-disubstituted trichloroacetimidate and induces critical hydrogen bonding interactions.^35^ We rationalize that this fluoromethyl stabilization could reduce the rate of amination, as well as control regioselectivity and stereoselectivity via critical hydrogen bond interactions. Furthermore, this hypothesis could be achieved by leveraging the rapid π–σ–π interconversion (*k*_1_) of the *syn*-π-allyl intermediate to the more reactive *anti*-π-allyl intermediate, rather than by trying to avoid it (Figure 1G).^38^ To achieve high levels of *Z*-selectivity, the rate of interconversion (*k*_1_) of the *syn/anti* intermediates must be faster than the rate of nucleophilic attack (*k*_1_ ≫ *k*_2_, *k*_3_). Additionally, the rate of nucleophilic attack by the secondary amine on the *anti* intermediate should be faster than the *syn* intermediate (*k*_2_ *> k*_3_), establishing a Curtin-Hammett condition (Figure 1G).^39,40^ Herein, we report the scope and mechanism of the *Z*-selective synthesis of trisubstituted alkenes bearing allylic tertiary amines using cyclooctadiene-ligated rhodium catalyst. We combined experimental and computational data to determine a detailed mechanism for our reaction and to explain the origin of both regio- and stereoselectivity. This methodology furnished 38 trisubstituted alkenes bearing tertiary aliphatic and aryl amines in good yields (45–98% yields) and excellent selectivities (*E/Z* > 1:20 and > 99:1 linear/branched).

## Results and Discussion

### Reaction and Development

We commenced our study by evaluating the equally critical, regioselective parameter for the stereoselective *Z*-selective amination of the 1,1-disubstituted π-allyl intermediate. To achieve this goal, our reaction condition must be able to (i) prevent both the rearrangement of the 1,1-disubstituted allylic trichloroacetimidate and amine-induced elimination side product;^41,42^ (ii) avoid the regioselective C3 preference of amination previously reported by our group using the 1,1-disubstituted allylic trichloroacetimidate.^35,36,43^ Given our long-standing interest in asymmetric tertiary amine synthesis using the rhodium-catalyzed allylic substitution reaction, we envisioned that selecting a specific Rh-ligand complex could induce the linear amination. This would favor the C1 attack of the 1,1-disubstituted π-allyl species by a secondary amine nucleophile. Although we have established that secondary amines can erode the branched selectivity of amination at the π-allyl intermediate,^43^ the importance of the 1,1-disubstituted allylic electrophile in controlling regioselectivity cannot be overstated. As a result, we began by examining how the tertiary allylic trichloroacetimidate influences the regioselectivity. In our previous study on the regioselectivity of the methyl-substituted 1,1-disubstituted trichloroacetimidate **1a** using 4-methoxy-*N*-methylaniline (**2a**) as the secondary amine, we observed a minor linear product **3**, along with the major branched product **4** with excellent levels of regioselectivity (*l*/*b* > 1:30) (Scheme 1A).^44^ In a recent study on amination involving the 1,1-disubstituted fluoromethyl allylic trichloroacetimidates **1b**, we explored the potential reactivity and regioselective effects of the fluoromethyl group on the branched selectivity of amination.^35^ Although we did not observe a reversal in branched regioselectivity with a primary aniline, we rationalized that a secondary aryl amine nucleophile could exacerbate the linear to branched ratio of the fluoromethyl group. Building on this hypothesis, we investigated the reaction of trichloroacetimidate **1b** with secondary aniline **2a** under optimized conditions.^35^ We observed reverse regioselectivity, favoring the linear product **5** (*l*/*b* > 4:1; Scheme 1B). Due to the reduced reactivity of trichloroacetimidate **1b** compared to trichloroacetimidate **1a** under the amination conditions, conversion to the allylic products **5** and **6** was incomplete, resulting in the recovery of **1b**. Nevertheless, encouraged by this development in regioselective bias, we next aimed to examine the impact of the allylic electrophile on regioselectivity using the 1,1-disubstituted fluoromethyl allylic trichloroacetimidates **1c**. We reasoned that linear regioselectivity could be further improved by replacing the free rotating −CH_2_OBn group in **1b** with the more restricted phenyl group in **1c**. The basis for this hypothesis stems from the knowledge that both the steric and electronic effects within the reactive 1,1-disubstituted π-allyl-rhodium intermediate impart the C1 (*linear*) or C3 (*branched*) preference by the incoming nucleophile (Figure 1F). As a result, increasing the steric interactions between the rhodium center and the allylic substituents would restrict the C3 approach by the bulky secondary amine nucleophile, thereby favoring the C1 attack (Figure 1G). To test this hypothesis, we subjected tertiary allylic trichloroacetimidate **1c** to our standard reaction conditions. We observed a drastic improvement in regioselectivity, from a linear-to-branched (l/b) ratio of 4:1 to 99:1, favoring the linear trisubstituted alkene product **7** (Scheme 1B). The minor branched product **8** was not observed in the amination reaction.

**Scheme 1:**
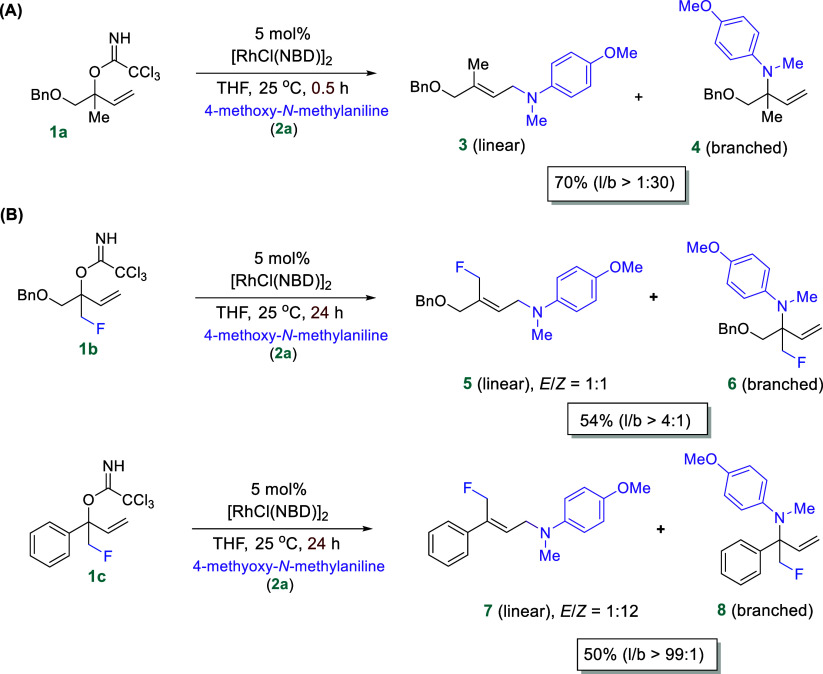
Preliminary Studies of Rhodium-Catalyzed Amination of 1,1-Disubstituted Allylic Trichloroacetimidates with 4-Methoxy *N*-Methylaniline (a) Privious Work (b) Current Work

Next, we shifted our focus to the more demanding and challenging stereoselectivity consideration (*E*/*Z* product). Before proceeding with optimization, we observed that the reaction with the 4-methyl-*N*-methylaniline nucleophile **2b** afforded a higher-yielding product than the reaction with the secondary aniline **2a** in the regioselective studies (Tables 1, entry 1). Therefore, we decided to adopt aniline **2b** for subsequent studies on *E/Z* selectivity. It is important to note that the choice of ligand is critical for controlling the stereoselectivity of the allylic substitution.^45^ From our reaction design (Figure 1G), the ligand should be able to distinguish the reactivity between the *syn/anti* intermediates to induce *E*/*Z* selectivity. As a result, we screened a number of ligands based on our previous work with the rhodium catalyst.^46^ In our earlier reports on tertiary amination, phosphine and phosphite ligands slightly favored linear regioselectivity over diene ligands.^43^ In this study, we hypothesize that the phosphine and phosphite ligands,^44^ could also favor the more reactive isomer of the π-allyl-Rh complex (*syn* or *anti*) and thus induce *E/Z* selectivity in the product (Figure 1G). Unfortunately, the use of phosphine and phosphite ligands did not yield any significant isolable products (Tables 1, entries 2 and 3). Undeterred by this setback, we decided to continue utilizing our established diene ligands for rhodium-catalyzed allylic amination.^48,51,52^ We previously developed a DYKAT-type allylic substitution reaction using Hayashi’s chiral diene ligand that effectively distinguishes between the populations of the *syn*- and *anti*-π-allyl-rhodium intermediates.^46,47^ Based on this finding, we hypothesize that Hayashi’s chiral diene ligand, Ph-bod, may influence the reactivity of one π-allyl species (either *syn* or *anti*), thereby inducing *E*/*Z* selectivity in the amination reaction. However, the yield and selectivity of product **9** were significantly reduced (Tables 1, entry 4), suggesting that the chiral diene ligand, Ph-bod, has a negative impact on both ionization and isomerization of the reactive π-allyl intermediates. Previously, we studied the extensive rhodium-diene ligand-binding strength and its direct effect on the reactivity of the 1,1-disubstituted trichloroacetimidate amination reaction.^36,37^ Here, we rationalized that a slightly weaker rhodium-diene binding could probably have a positive effect on the reactivity and *E*/*Z* selectivity of the 1,1-disubstituted trichloroacetimidate.

**Table 1:**
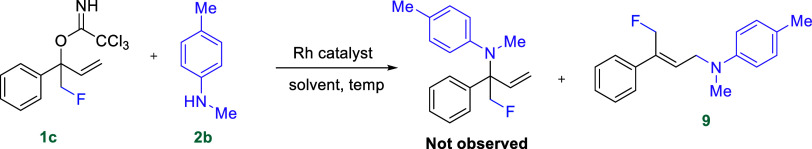

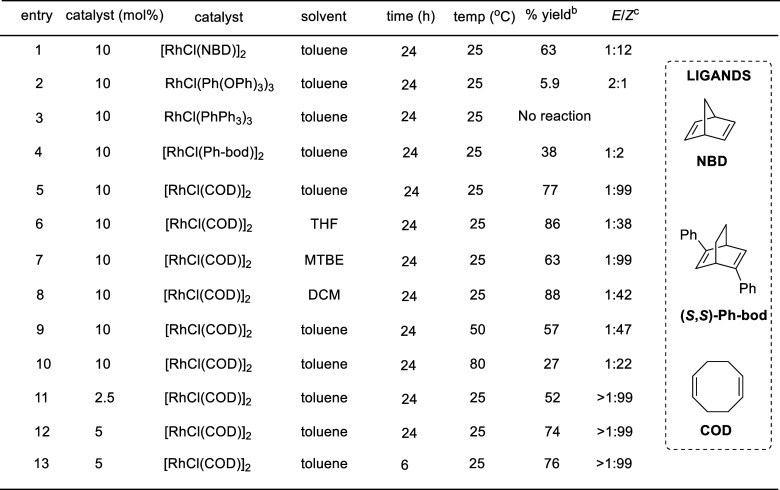
Reaction optimization studies^a^ ^b^ ^c^

a All reactions were conducted at 0.2 M with 0.1 mmol of **1c** and 0.15 mmol of **2b**.

b Isolated yields.

c The E/Z ratio was determined by ^19^F and ^1^H NMR of the crude product. NBD = Norbornadiene, COD = 1,5-cyclooctadiene, and (S,S)-Ph-bod = (1S,4S)-2,5-diphenylbicylo[2.2.2]­octa-2,5-diene.

The 1,5-cyclooctadiene (COD) ligand has been shown to bind weakly to the metal center relative to both the NBD and Ph-bod diene ligands.^48^ As a result, it is often used as a released ligand.^49^ However, the COD ligand can be used effectively as a ligand for transition metals when weak coordinating substrates are used in the reaction.^50,51^ As a result, we performed the amination of aniline **2b** with trichloroacetimidate **1c** using [RhCl­(COD)_2_] as a catalyst. To our excitement, the use of the COD ligand led to an improved yield (77%) and selectivity for product **9** (*E/Z* 1:99; Tables 1, entry 5). Next, we optimized other parameters that could potentially affect reactivity and selectivity. For optimal yield and *Z*-selectivity, the equilibration of the π-allylrhodium intermediates must occur significantly faster than the rate of secondary aniline attack on the π-allyl complex (Figure 1E).^52^ With this in mind, several solvents were screened (Tables 1, entries 5–8), and the use of toluene led to better product yield and selectivity. In view of improving the yield due to the poor reactivity of the fluoromethyl trichloroacetimidate **1c**,^35^ we increased the reaction temperature (entries 9 and 10). However, both yield and selectivity decreased rapidly as the temperature increased. We rationalized that the increased temperature led to higher rates of nucleophile attack (*k*_2_, *k*_3_), which offset any gains from the rapid equilibration of the π-allylrhodium complex. Additionally, the poor yield could be attributed to the rapid decomposition of **1c** at elevated temperatures. Ultimately, optimization studies involving the ligand, reaction temperature, reaction time, and solvent led us to identify the optimized reaction conditions: 5 mol % [RhCl­(COD)]_2_ in toluene at 25 °C for 6 h. This resulted in the formation of *Z*-trisubstituted alkene **9** bearing α-tertiary aniline in 76% yield with *b*/*l* > 1:99 and *E*/*Z* > 1:99 (entry 13).

### Substrate Scope

With the optimized conditions established, we examined the nucleophilic potential of secondary aliphatic amines and anilines in the amination reaction (Scheme 2). We investigated a series of *para*-substituted *N*-methylanilines, leading to the synthesis of *Z*-trisubstituted products (**7**, **9**–**12**, **16**, and **17**) that contain allylic tertiary aliphatic and aryl amines. These products were obtained in good yields (47–74%) and exhibited excellent selectivities (*E*/*Z* > 1:99). The incorporation of both electron-donating (**7**, **9**) and electron-withdrawing (**10**, **12**, **17**) substituents did not significantly impact the yield or *Z*-selectivity. Furthermore, both *ortho* (**15**, **19**) and *meta* (**13**, **14**, **18**) substituted *N*-methylanilines proved suitable for *Z-*selective amination, affording products in good yields (44–82%) with *E*/*Z* selectivity > 1:20. *N*-aryl amines featuring bulky aliphatic groups were also compatible, resulting in the amination products (**20**, **21**) with good yields and selectivity (*E*/*Z* > 1:15). Additionally, diarylamines with acyclic structures (**28**) and cyclic fused diaryl rings (**29**, **30**) performed well under standard amination conditions, affording excellent yields and selectivity. Overall, our allylic amination performed favorably with several substituted aryl amines, affording *Z*-configured alkenes.

**Scheme 2:**
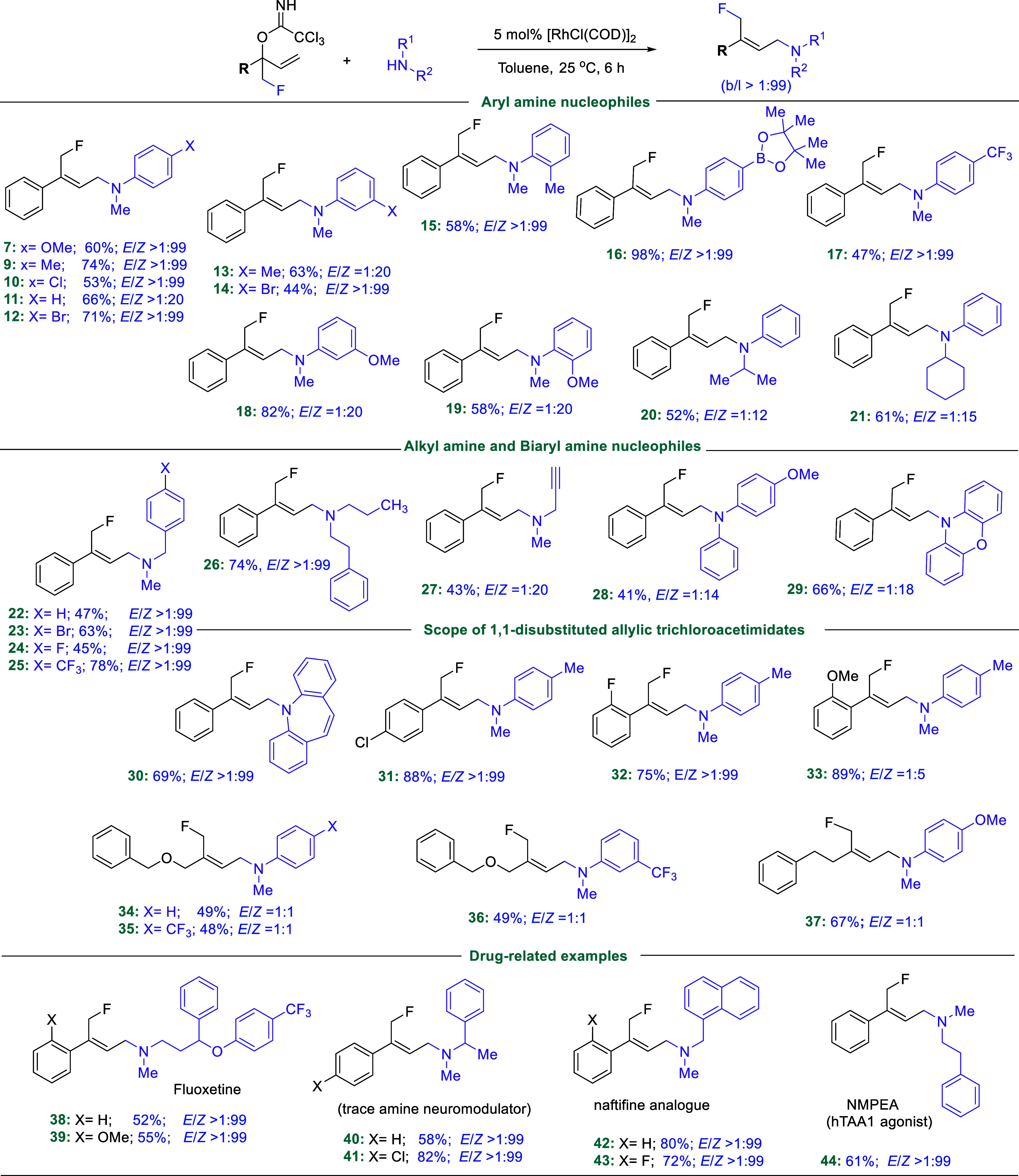
Scope of Secondary Aliphatic and Aryl Amines and 1,1-Disubstituted Fluoromethyl Allylic Trichloroacetimidates^a^

a All reactions were performed using 0.1 mmol of 1,1-disubstituted allylic trichloroacetimidate, 5 mol% [Rh­(Cl­(COD)]_2_, 0.15 mmol of secondary aliphatic amine or aniline. Isolated yields were reported. The E/Z values were determined from the crude ^19^F and ^1^H NMR. Reaction completed in 6 h at 25 °C.

Aliphatic amines are an essential class of amines ubiquitous in agrochemicals and pharmaceuticals, and they form a large class of synthetic amine intermediates, both for catalysis and industrial processes.^7^ Generally, this class of amines is challenging in transition-metal catalysis due to their propensity to coordinate to the metal and undergo β-hydride elimination^53^ and base-catalyzed elimination. As such, we explored the scope and limitations of our methodology for the synthesis of the *Z*-configured trisubstituted alkene bearing allylic tertiary aliphatic amines (Scheme 1). Benzyl amine compounds (**22–25**) showed excellent reactivity (45–78% yields) and selectivity (*E/Z* > 1:99). We found that the nature of the para-substituents on the benzyl group of the amines had minimal effect on the reaction efficiency. To demonstrate the synthetic utility of our reaction design, we conducted an extended gram-scale reaction for the *N*-benzylamine product **23**. The reaction proceeded efficiently, maintaining an *E*/*Z* selectivity ratio of 1:99, and yielded 60% of the desired product.

More significantly, the sterically hindered *N*-propylphenylamine and the acyclic secondary *N*-methylpropargylamine were also highly efficient, affording products **26** and **27**, respectively, with excellent yields and *Z*-selectivity. To assess the functional group tolerance of 1,1-disubstituted fluoromethyl trichloroacetimidates, we attempted to synthesize several substituted aliphatic aryl substrates. Despite our efforts, we were unable to synthesize phenyl-substituted trichloroacetimidates bearing substituents such as I, CN, or Br, as well as a few other allylic starting materials. This limitation arises from the low reactivity of tertiary allylic alcohols toward trichloroacetonitrile, which produces the corresponding trichloroacetimidates. Nevertheless, a few substrates containing *para*-Cl (**31**) and *ortho*-F (**32**) motifs yielded the desired products with excellent yields (>75%) and selectivity (*E/Z* 1:99). In Scheme 1, we demonstrated that the −CH_2_OBn group in the 1,1-disubstituted allylic substrate **1b** produced **5** as a 1:1 mixture of *E*/Z using [RhCl­(NBD)]_2_. Similar 1:1 *E/Z* selectivity was observed in reactions with **1b** with other aniline nucleophiles, leading to the formation of **34**–**36**, even switching to [RhCl­(COD)]_2_. However, the linear/branch ratio of the aminated products **34–36** remains exclusively linear when compared to **5** (*l*/*b* = 4:1). Attempting to repeat the reaction of **1b** and aniline **2a** with [RhCl­(COD)]_2_ improved the branched and linear products (*l/b* 1:4 → 1:12) of product **5**. We rationalized that the para-*O*-methoxy group could play a key role in reducing the *l/b* selectivity. A similar trend was also observed with the aliphatic carbon chain 1,1-disubstituted allylic compound **37** (Scheme 2).

Finally, our synthetic methodology offers an opportunity to synthesize targets potentially relevant to medicinal chemistry and to evaluate the effectiveness of *Z*-selective allylic amination. We successfully synthesized different classes of *Z*-trisubstituted alkenes bearing allylic tertiary amines, including fluoxetine derivatives (**38** and **39**), neuromodulators (**40** and **41**), naftifine analogs (**42** and **43**), and NMPEA, an hTAA1 agonist (**44**), in good yields (52–82%) and with excellent *Z*-selectivity (*E*/*Z* > 1:99).

### Mechanism

To assess the efficacy of our Rh-L complex in modulating the reactivity and selectivity of our amination, we conducted a series of control experiments. Our reaction is intentionally designed to produce the π-allyl-rhodium complex. However, due to the structure of our allylic starting material, the possibility of undergoing an S_N_2′ mechanism^54^ or alkene isomerization^55^ cannot be ruled out. The ability of transition metals to direct reactivity and selectivity via an S_N_2′ pathway^56,57^ has been reported. Key questions to address include (i) the determination of ionization or direct reaction with amine (S_N_2′) conditions, (ii) rhodium-directed product isomerization or π-allyl-rhodium complex of the 1,1-disubstituted allylic substrate, (iii) energetic preference of the π-allyl-rhodium complex in controlling both the regioselectivity (*branched vs linear*), and the stereoselectivity (*E vs Z*). Therefore, we conducted a series of experiments to probe the reaction pathway (see Supporting Information page S23). To determine whether the reaction proceeds via an S_N_2′ pathway, we set up our standard reaction in the absence of [RhCl­(COD)]_2_ and found that there was no reaction in the absence of the catalyst (see Supporting Information page S23A). To determine if rhodium functions as a Lewis acid to activate the trichloroacetimidate group, we investigated the reaction of tertiary fluoromethyl trichloroacetimidate **1c** with aniline **2a** in the presence of 5 mol % of AgOTf as a Lewis acid^44,58^ (see Supporting Information page S23B). The starting material **1c** remained unreacted, and we observed no trace of the amination product. This result indicates that the rhodium catalyst does not behave as a Lewis acid. Next, we subjected the trisubstituted alkene product **9** (*E*/*Z* = 1:6), synthesized from [RhCl­(NBD)]_2_, to the standard optimized conditions using [RhCl­(COD)]_2_ as the catalyst to explore a potential isomerization pathway. After 6 h, there was no change in the selectivity of compound **9** (see Supporting Information page S23C), indicating that *E/Z* isomerization in the product was unlikely.

Next, we analyzed the role of the π-allylrhodium complex in determining the reactivity and selectivity of the reaction. We utilized Density Functional Theory (DFT) calculations to investigate the mechanism for the formation of trisubstituted olefin products that contain allylic tertiary amines. The computed profiles are reported in Figure 2. The starting point is **R-E2**, which is a square-pyramidal complex of rhodium­(I) (Rh^I^). The *R*-isomer of the 1,1-disubstituted fluoromethyl allylic trichloroacetimidates starting material, **1c**, is bound to Rh^I^ in a bidentate fashion, consistent with the previously reported computational structure reported by our group.^36,37,46^ This bidentate binding of **1c**, involving coordination to the rhodium center through both the alkene π-bond (*η* ^*2*^ *-*binding) and the heteroatom of the trichloroacetimidate group (κ^*1*^ interaction), leads to two distinct penta-coordinated complexes, denoted **R-E1** and **R-E2** (Figure 2). These, in turn, follow two separate free-energy pathways that give rise to the *Z*- and *E*-isomers of the linear product, respectively. In both pathways, the first step involves cleavage of the C–O bond. This step proceeds through two closely related transition states, **TS1** (from **R-E1**) and **TS1′** (from **R-E2**), and results in the formation of two π-allylrhodium intermediates with different relative stereochemistry, the *anti*-intermediate **R-I1** and the *syn*-intermediate **R-I2**. The calculated kinetic barrier (ΔG^⧺^) for C–O bond cleavage is 14.67 kcal mol^–1^ for **TS1**, leading to **R-I1**, and 15.30 kcal mol^–1^ for **TS1′**, leading to **R-I2**. The *anti*-intermediate **R-I1** is located at −2.20 kcal/mol relative to **R-E2**, while the *syn*-intermediate **R-I2** is at −4.84 kcal mol (Figure 2). These energies indicate that the *syn*-intermediate **R-I2** is thermodynamically more stable than the *anti*-intermediate **R-I1** by 2.64 kcal mol^–1^, and consequently, the more populous intermediate is in the reaction system.

**Figure 2:**
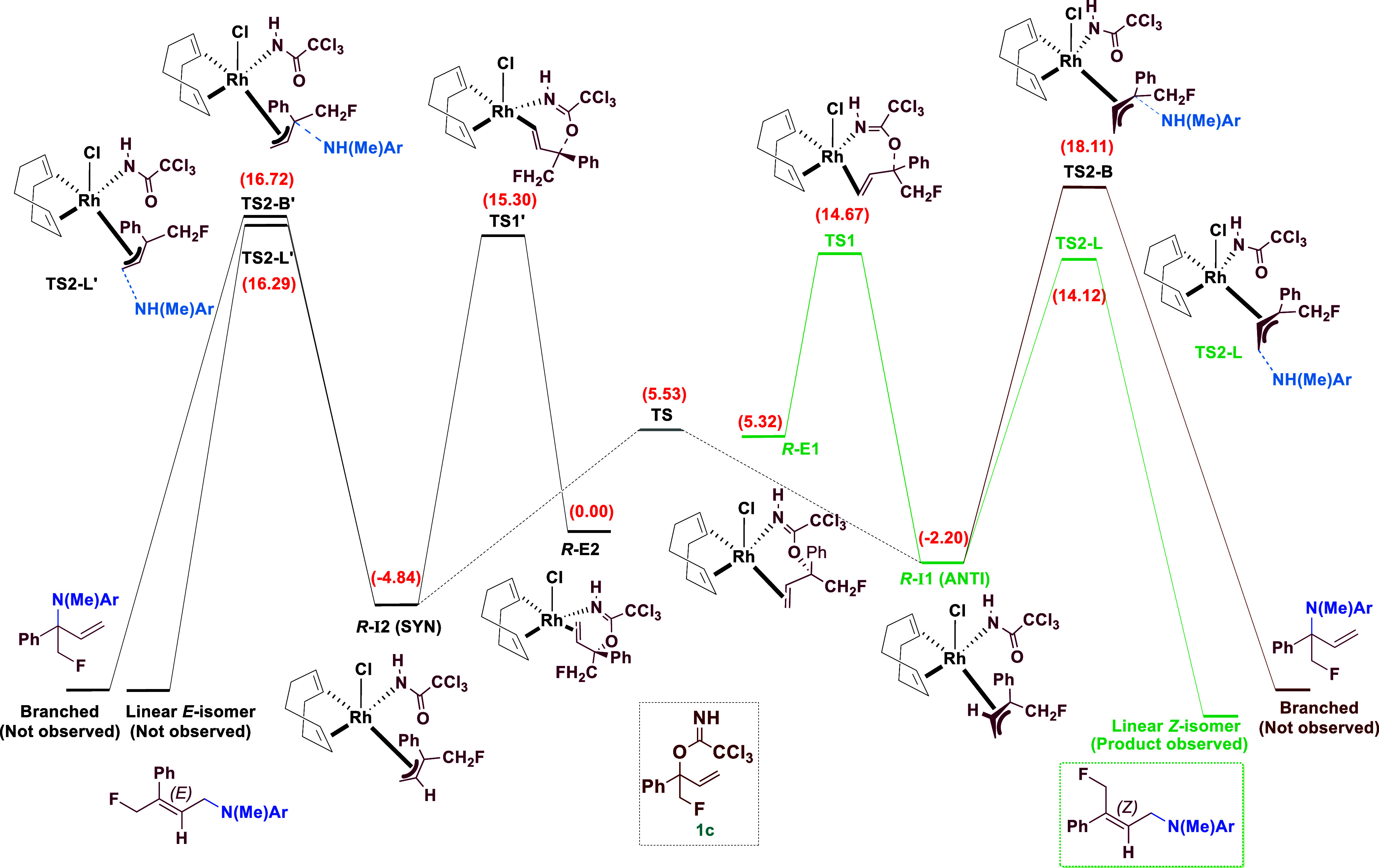
Computed free energy profile diagram for the formation of trisubstituted olefins bearing tertiary amines. Relative free energy changes (ΔG) are in kcal mol^–1^ and are computed with the Gaussian 16 program package^59^ at the PBE1PBE-D3­(BJ)^60^/6-311+G­(d,p)^61^ level of theory using toluene with SMD implicit solvation.^62^

Nucleophilic attack by amine on the *syn-*intermediate **R-I2** can lead to the *E*-isomer of the linear product or to a branched product via transition states **TS2-L′** or **TS2-B′**, respectively (Figure 2). The activation barriers for these steps are 16.29 kcal mol^–1^ for **TS2-L′**, leading to the linear *E*-product, and 16.72 kcal mol^–1^ for **TS2-B′**, leading to the branched product. From *anti*-intermediate **R-I1**, nucleophilic attack by the amine proceeds through two competing transition states, **TS2-L** and **TS2-B**, leading to the linear and branched products, respectively. The calculated activation free energies for these steps are 14.12 kcal mol^–1^ for **TS2-L** for the formation of the linear *Z*-product and 18.11 kcal mol^–1^ for **TS2-B** for the formation of the branched product. Notably, the barrier for the isomerization from the *syn* to the *anti* π-allyl intermediate (ΔG^⧺^ = 5.53 kcal mol^–1^) is much lower than the barriers for amination of the *syn* and *anti-*intermediates (Figure 2), indicating that *syn-to-anti* interconversion is faster than nucleophilic attack to form the products. We hypothesize that this rapid equilibration is enabled by the relatively labile Rh/COD coordination environment, which allows facile *syn-to-anti* interconversion. This observation is consistent with a Curtin–Hammett–type scenario in which the two π-allyl intermediates (**R-I1** and **R-I2**) may coexist in rapid pre-equilibrium, but the product distribution is governed by the relative free energies of the transition states for nucleophilic attack by the amine. **TS2-L** is therefore ∼4.0 kcal mol^–1^ lower in energy than **TS2-B**. This significantly lower activation barrier for **TS2-L** indicates a strong kinetic preference for the formation of the linear *Z*-product from **R-I1**. The pathway from the *syn-*intermediate **R-I2** to *anti-*intermediate **R-I1** and nucleophilic addition via **TS2-L** to the *Z*-isomer of the linear product is the minimum energy pathway. Our DFT calculations are in full agreement with the experimental results, as the linear *Z*-product is predominantly observed under these conditions.

The lower activation barrier for **TS2-L** relative to **TS2-B** originates from stabilizing noncovalent interactions. The optimized 3D geometries of the two transition states, **TS2-L** and **TS2-B**, are shown in Figure 3. **TS2-L** (ΔG^⧺^ = 14.12 kcal mol^–1^) is stabilized by a hydrogen bonding interaction between the incoming nucleophile and the substrate. Our calculations have identified an N–H···F hydrogen bond between the NH moiety of the incoming amine nucleophile and the F atom of the substrate. The distance between the H and F atoms is found to be 2.70 Å (Figure 3). In contrast, the transition state for the branched product, **TS2-B** (ΔG^⧺^ = 18.11 kcal mol^–1^), lacks this stabilizing hydrogen bonding interaction. The corresponding distance between the H and F atoms is found to be 3.72 Å; no other transition state with a shorter H···F distance was found.

**Figure 3:**
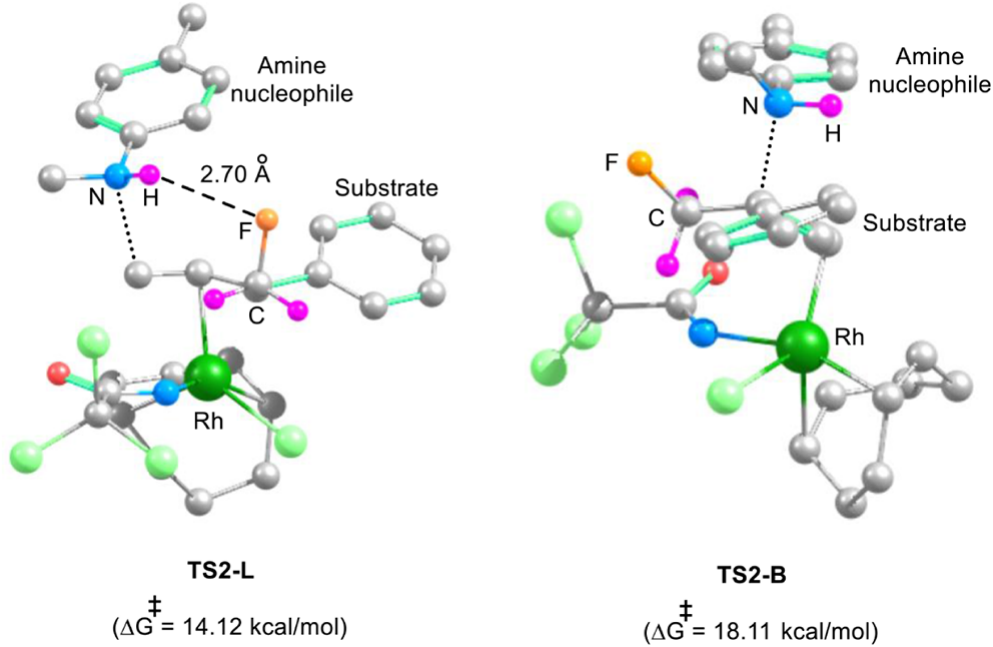
Optimized transition state structures of **TS2-L** and **TS2-B**.

The lower barrier for the linear product can be attributed to the NH···F hydrogen bond in the transition state. Similarly, the higher-energy *syn*-derived transition states **TS2-L′** and **TS2-B′** are less stabilized than **TS2-L**, because the lowest-energy conformation of **TS2-L′** does not exhibit an NH···F hydrogen-bonding interaction, whereas **TS2-B′** remains sterically crowded despite showing some NH···F interaction. In particular, the optimized structure of **TS2-B′** shows a closer phenyl–phenyl proximity than **TS2-L**, leading to a more congested transition-state geometry (Figure S4).

To further probe the role of hydrogen bonding, an additional computational model was designed in which the fluorine atom in the CH_2_F functional group of the substrate was replaced with a hydrogen atom, yielding a CH_3_-substituted analog. The two transition states were recalculated using this CH_3_-substituted substrate. The new transition states are called **TS2-L**_**Me**_ and **TS2-B**_**Me**_ (Figure S1). Analysis of these optimized transition-state structures confirms that **TS2-L**_**Me**_ lacks any hydrogen bonding interactions with the incoming nucleophile. In the original CH_2_F-containing system, the free-energy difference between **TS2-L** and **TS2-B** is 3.99 kcal mol^–1^ (Figure 3). **TS2-L** is the lower-energy transition state and is therefore favored. In contrast, in the CH_3_-substituted model, **TS2-L**_**Me**_ becomes 1.18 kcal mol^–1^ higher in energy than **TS2-B**_**Me**_. This inversion in relative transition-state energies reflects the loss of hydrogen-bonding stabilization in **TS2-L**_**Me**_ upon removal of the fluorine atom. This is strongly validated by experimental findings, where the reaction of methyl-substituted substrate **1a** with 4-methoxy-*N*-methylaniline **2a** yields the branched product **4** with a high branched-to-linear ratio of 30:1 (Scheme 1A).

## Conclusion

We have developed a strategy for the transformation of the challenging 1,1-disubstituted fluoromethyl π-allylrhodium intermediate in the presence of a secondary amine to provide access to the *Z*-trisubstituted alkene bearing an allylic tertiary amine in good yield and selectivity. The key insight for our design is to control the isomerization of the reactive π-allyl intermediate and the nucleophile’s kinetic preference for the *anti*-π-allyl intermediate. Computational DFT calculations show the thermodynamically favored *syn*-π-allyl equilibrates toward the more reactive *anti*-π-allyl, with equilibration between the two intermediates faster than amination, thereby establishing a Curtin-Hammett process via the kinetic dynamic resolution of our product. Our investigation also highlighted the crucial role of NH···F hydrogen-bonding interactions between the amine nucleophile and the fluoromethyl group in stabilizing the transition state and determining the high *Z*-selectivity. This interaction is not present in the analogous methyl-substituted substrate, thereby accounting for the switch in regioselectivity. The current study is particularly important as it provides a regioselective and stereocontrolled route to *Z*-trisubstituted alkenes, structural motifs that are difficult to access yet frequently found in biologically active molecules. An exploration of substrate scope with various classes of amines underscores the synthetic utility and potential pharmaceutical relevance of this *Z*-selective transformation. Finally, we are currently expanding this method to include other nucleophiles such as alcohols, carboxylic acids, and thiols. The results will be reported in due course.

## Supplementary Material



## Notes

The general synthesis of the key intermediates, amination products, control experimental procedure, and general procedure of the study, DFT coordinates, and ^1^H, ^13^C, and ^19^F NMR spectra of the synthetic compounds (PDF)
