# Supplementary material for: Z‑Selective Synthesis of Trisubstituted Alkenes Bearing Allylic Tertiary Amines via Rhodium-Catalyzed Allylic Amination of 1,1-Disubstituted Fluoromethyl Trichloroacetimidates
Source: JACS Au. 2026 Jul 3;6(7):4096–106. doi: 10.1021/jacsau.6c00604 (PMC13417294; doi:10.1021/jacsau.6c00604)
Supplement: Supplementary file 1 [file au6c00604_si_001.pdf]

Supporting Information

Z-Selective Synthesis of Trisubstituted Alkenes Bearing Allylic Tertiary Amine via Rhodium-Catalyzed Allylic Amination of 1,1-Disubstituted Fluoromethyl Trichloroacetimidates

Felix O. Chukwu,[‡] *Neha Rani,*[‡] *Rollanda Tella, H. Bernhard Schlegel,* Hien M. Nguyen**

Department of Chemistry, Wayne State University, Detroit, Michigan 48202, United States.

Email: hbs@chem.wayne.edu (H.B. Schlegel) and hmnguyen@wayne.edu (H. M. Nguyen)

Table of Contents

1. **General Information ……………………………………………………………………..S2**
2. **Experimental Procedures ……………………………………………………………….S3-S6**
3. **Analytical Data of All Compounds …………………………………………………….S7-S22**
4. **Control Studies and Proposed Mechanism………………………………………………….S23**
5. **Computational Data …………………………………………………………………......S24-S52**
6. **References…………………………………………………………………………………..S53**
7. **NMR Spectral Data of all Compounds ………………………………………………........S54-S123**

1. General Information

Methods and Reagents

All reactions were performed in oven-dried Schlenk flasks fitted with glass stoppers under positive argon pressure. Organic solutions were concentrated by rotary evaporation below 40 °C at 25 torr. Analytical thin-layer chromatography (TLC) and nuclear magnetic resonance spectroscopy (NMR) were routinely used to monitor the progress of the reactions. TLC was performed using pre-coated glass plates with 230-400 mesh silica gel impregnated with a fluorescent indicator (250 nm). Visualization was accomplished using UV light, potassium permanganate, or phosphomolybdic acid. Flash chromatography was performed on silica gel flash chromatography columns or a Teledyne Isco CombiFlash Rf system utilizing normal phase pre-column cartridges and gold high-performance columns. Purifications were performed using ethyl acetate/n-hexane eluting with a gradient method starting at 0:100 (ethyl acetate: n-hexane) and ending at 40:60 (ethyl acetate:n-hexane). All allylic trichloroacetimidates and tertiary amines were concentrated into 20 mL scintillation vials and stored under argon in a fridge at 0 °C until further use or characterization. Allylic trichloroacetimidates and allylic tertiary amines were checked by ^{1}H NMR after six months, showing little to no degradation. Dry solvents were obtained from a SG Waters solvent system utilizing activated alumina columns under an argon pressure or purchased from Sigma-Aldrich in sealed bottles. The rhodium catalysts and diene ligands were handled and transferred to Schlenk flasks within a glove box under an argon atmosphere. All chemicals and reagents were obtained from commercial vendors and used without further purification.

Instrumentation

All proton (^{1}H) nuclear magnetic resonance spectra were recorded on a 500 MHz spectrometer. All carbon (^{13}C) nuclear magnetic resonance spectra were recorded on a 126 MHz NMR spectrometer. All fluorine (^{19}F) nuclear magnetic resonance spectra were recorded on a 471 MHz NMR spectrometer and were fully decoupled by broad-band proton decoupling. Chemical shifts are expressed in parts per million (δ scale) and are referenced to residual $CHCl_3$ (^{1}H: δ 7.26 ppm, ^{13}C: δ 77.23 ppm) in the NMR solvent. Data are presented as follows: chemical shift, multiplicity (s = singlet, d = doublet, t = triplet, q = quartet, m = multiplet, and bs = broad singlet), integration, and coupling constant in hertz (Hz). High resolution TOF mass spectrometry utilizing electrospray ionization (ESI) in positive mode to confirm the identity of the compounds

2. Experimental Procedures

2.1 General Procedure for the Synthesis of Tertiary Allylic Alcohol

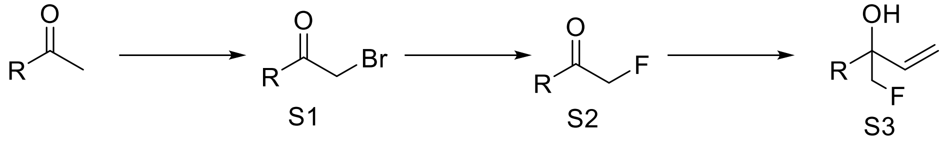

S1: At room temperature, *N*-bromosuccinimide (1.2 equiv. 29.0 mmol) was added dropwise to a solution of ketone (1.0 equiv. 24.0 mmol) and toluene-4-sulfonic acid (1.2 equiv. 29 mmol) in acetonitrile (0.5 equiv.), and the reaction mixture was stirred at 80 °C for 24 h. After completion of the reaction, the reaction was quenched with water and extracted with DCM. The combined organic layers were washed twice with saturated NaCl solution, dried over anhydrous Na_2SO_4, and the solvent was removed under vacuum. The crude product S1 was isolated for the next step without further purification.

S2: To a solution of the crude α-bromo-ketone S1 (17.10 mmol, 1.00 equiv.) in a 500 ml round-bottom flask (RBF) was added acetonitrile (ACN) (200 mL; 0.1 equiv.), 18-crown-6 (0.2 equiv.), and KF (37.69 mmol, 2.2 equiv.). The resulting mixture was stirred overnight at 89 °C. The reaction was allowed to cool, then quenched by the addition of 200 mL of water/ice. The resulting solution was extracted with 3x100 mL of ethyl acetate, and the organic layers were combined, washed with brine, and dried over anhydrous Na_2SO_4. Concentration under vacuum afforded the crude compound as a white solid. The crude compound was then purified by flash column chromatography a using 5–25% EtOAc in hexanes to afford the alcohol **S2** as a yellow oil (60-90% yield).

S3: To a solution of ketone **S3** (9.90 mmol, 1 equiv.) in 33 mL THF was added vinylmagnesium bromide (15 mL, 14.85 mmol, 1.5 equiv., 1M solution in THF) at -78 °C. The reaction mixture was brought to room temperature gradually. The reaction was monitored by TLC and was found to be complete in 5 hours. The reaction mixture was quenched by saturated ammonium chloride solution (10 mL), and the reaction mixture was transferred to a separatory funnel containing 20 mL THF and extracted with ethyl acetate (3 × 50 mL). The separated organic layer was washed with brine solution and dried over Na_2SO_4. The residue obtained was purified by flash chromatography on silica using 5-20% ethyl acetate/hexanes to obtain allylic alcohol **S4** as a brown oil (60-73% yield).

2.2 General Procedure for the Synthesis of Tertiary Allylic Trichloroacetimidate

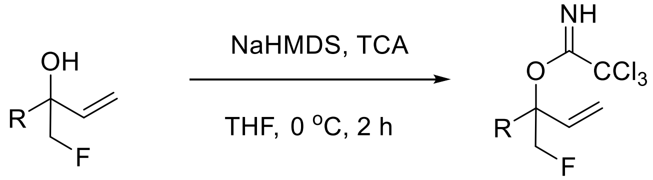

A 25 mL oven-dried Schlenk flask was charged with allylic alcohol (400 mg, 1.90 mmol, 1.0 equiv) in dry THF (5 mL). The suspension was cooled to 0 °C, and NaHMDS (0.76 mL, 0.76 mmol, 0.4 equiv., 1M in THF) in THF was added dropwise. The resulting mixture was allowed to stir for 30 min. Trichloroacetonitrile (8.55 mmol, 3.0 equiv.) and THF (5 mL) were added to a separate oven-dried Schlenk flask and cooled to 0 °C. The alcohol/sodium alkoxide solution was then transferred slowly to the trichloroacetonitrile solution *via* cannula. The reaction mixture was stirred at 0 °C for 1.5 h, concentrated

in vacuo, and loaded directly onto a column containing alumina pre-equilibrated with 1% triethylamine in hexanes. The crude product was then purified by silica gel flash column chromatography (1% ethyl acetate/hexanes + 1% triethylamine) to provide the tertiary allylic trichloroacetimidate substrate as a bright red oil (85-90% yield).

Note: Tertiary allylic trichloroacetimidates exist as mixtures of rotamers. Minor peaks in the ^{1}H and ^{13}C NMR spectra represent minor rotamers. We have reported a Variable Temperature NMR experiment on tertiary trichloroacetimidates as evidence.[1]

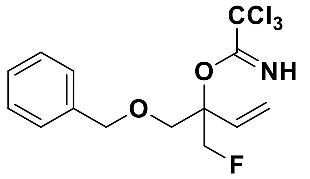

1b

We have previously reported the synthesis and characterization of this compound.[2]

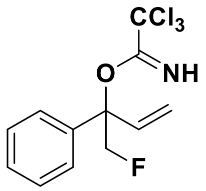

1c

^{1}H NMR (500 MHz, $CDCl_3$) δ 8.41 (s, 1H), 7.49 – 7.44 (m, 2H), 7.42 – 7.37 (m, 2H), 7.37 – 7.32 (m, 2H), 6.45 (ddd, J = 17.6, 11.1, 1.2 Hz, 1H), 5.57 – 5.48 (m, 2H), 5.04 (ddd, J = 54.6, 47.1, 9.6 Hz, 2H).
^{13}C NMR (126 MHz, $CDCl_3$) δ 158.83, 138.12 (d, J = 2.3 Hz), 135.35 (d, J = 2.9 Hz), 128.55, 128.39 (d, J = 6.2 Hz), 126.45, 125.97 (d, J = 1.2 Hz), 118.56 (d, J = 2.1 Hz), 92.01, 85.97 (d, J = 17.9 Hz), 84.69 (d, J = 182.5 Hz).
^{19}F NMR (471 MHz, $CDCl_3$) δ -225.18.
HRMS (ESI-TOF) m/z: $[M + Na]^+$ Calc. for for $C_{12}H_{11}Cl_3FNONa$ 331.9782; found 331.9785.

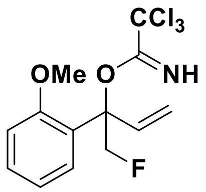

1d

^{1}H NMR (500 MHz, $CDCl_3$) δ 8.42 (s, 1H), 7.54 (dd, J = 7.8, 1.7 Hz, 1H), 7.03 – 6.86 (m, 4H), 6.43 (dt, J = 17.4, 10.8 Hz, 1H), 5.40 (dd, J = 17.5, 0.8 Hz, 1H), 5.34 (dd, J = 9.7, 3.2 Hz, 1H), 5.29 (d, J = 11.0 Hz, 1H), 5.23 (d, J = 2.8 Hz, 1H), 3.81 (s, 3H).

^{13}C NMR (126 MHz, $CDCl_3$) δ 158.82, 155.94, 136.19 (d, *J* = 3.5 Hz), 129.77, 129.62, 128.02, 120.88 (d, *J* = 6.0 Hz), 116.26 (d, *J* = 2.1 Hz), 111.77, 110.80, 86.51, 83.84 – 81.19 (m), 55.45, 46.26.
^{19}F NMR (471 MHz, $CDCl_3$) δ -226.27.
HRMS (ESI-TOF) m/z: $[M + Na]^+$ Calc. for for $C_{13}H_{13}Cl_3FNO_2Na$ 361.9888; found 361.9882

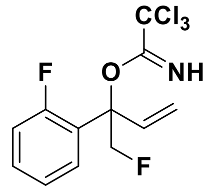

1e

^{1}H NMR (500 MHz, $CDCl_3$) δ 8.31 (s, 1H), 7.40 (td, *J* = 7.9, 1.8 Hz, 1H), 7.21 – 7.14 (m, 1H), 7.02 (td, *J* = 7.7, 1.2 Hz, 1H), 6.91 (ddd, *J* = 12.0, 8.2, 1.2 Hz, 1H), 6.26 (ddd, *J* = 17.6, 11.0, 1.8 Hz, 1H), 5.35 – 5.24 (m, 2H), 5.18 – 4.96 (m, 3H).
^{13}C NMR (126 MHz, $CDCl_3$) δ 160.50, 158.82, 158.53, 135.19 – 135.03 (m), 130.32, 130.25, 128.79 – 128.64 (m), 125.53 (dd, *J* = 11.2, 2.9 Hz), 124.30 (d, *J* = 3.4 Hz), 118.00, 116.61, 116.42, 91.89, 85.09 (dd, *J* = 18.3, 4.1 Hz), 82.89 (dd, *J* = 179.4, 7.4 Hz).
^{19}F NMR (471 MHz, $CDCl_3$) δ -111.29 (d, *J* = 4.4 Hz), -226.77 (d, *J* = 4.4 Hz).
HRMS (ESI-TOF) m/z: $[M + Na]^+$ Calc. for $C_{12}H_{10}Cl_3F_2NONa$ 349.9688; found 349.9684

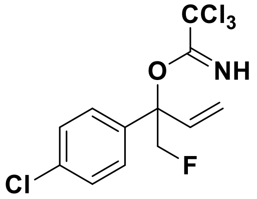

1f

^{1}H NMR (500 MHz, $CDCl_3$) δ 8.43 (s, 1H), 7.44 – 7.39 (m, 2H), 7.37 – 7.32 (m, 2H), 6.41 (ddd, *J* = 17.6, 11.1, 1.3 Hz, 1H), 5.56 – 5.47 (m, 2H), 5.11 (dd, *J* = 47.3, 9.6 Hz, 1H), 4.92 (dd, *J* = 46.8, 9.6 Hz, 1H).
^{13}C NMR (126 MHz, $CDCl_3$) δ 158.52, 136.54 (d, *J* = 2.1 Hz), 134.75 (d, *J* = 3.0 Hz), 134.15, 128.50, 127.33 (d, *J* = 1.3 Hz), 118.74 (d, *J* = 2.0 Hz), 91.58, 84.25 (d, *J* = 182.6 Hz), 67.87.
^{19}F NMR (471 MHz, $CDCl_3$) δ -225.35.
HRMS (ESI-TOF) m/z: $[M + Na]^+$ Calc. for $C_{12}H_{10}Cl_4FNONa$ 365.9393; found 365.9393

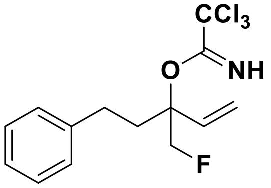

1g

^{1}H NMR (500 MHz, $CDCl_3$) δ 8.47 (s, 1H), 7.32 – 7.27 (m, 2H), 7.21 – 7.17 (m, 3H), 6.03 (ddd, J = 17.6, 11.2, 1.2 Hz, 1H), 5.51 – 5.38 (m, 2H), 4.98 – 4.88 (m, 1H), 4.88 – 4.79 (m, 1H), 2.74 (dd, J = 9.7, 7.5 Hz, 2H), 2.63 – 2.54 (m, 1H), 2.18 (dddd, J = 15.3, 13.7, 7.4, 3.7 Hz, 1H).
^{13}C NMR (126 MHz, $CDCl_3$) δ 157.24, 139.06, 133.36 (d, J = 3.6 Hz), 126.00, 125.89, 123.57, 114.44 (d, J = 1.3 Hz), 89.50, 83.47 (d, J = 18.1 Hz), 80.74 (d, J = 178.6 Hz), 33.13 (d, J = 3.0 Hz), 26.69 (d, J = 1.6 Hz).
^{19}F NMR (471 MHz, $CDCl_3$) δ -229.00.
HRMS (ESI-TOF) m/z: $[M + Na]^+$ Calc. for $C_{14}H_{15}Cl_3FNONa$ 360.0095; found 360.0090

2.4 General Procedure for Asymmetric Amination of Racemic Tertiary Allylic Trichloroacetimidates with Chiral Rhodium Catalyst

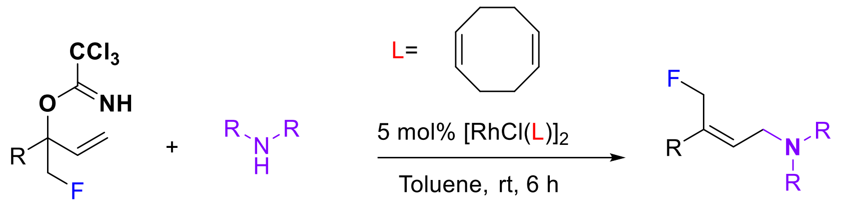

A 10 mL oven-dried Schlenk flask was charged with $[RhCl(COD)_2]_2$ (5 mol%) in a glove box. A separate 10 mL Schlenk flask was charged with allylic imidate (1.0 equiv), toluene (0.1 equiv.), and amine **2** (1.5 equiv.). The rhodium catalyst solution was then transferred to the flask containing the catalyst *via* a cannula. The reaction mixture was stirred at room temperature under an argon atmosphere. Reaction progress was monitored by TLC and both ^{1}H and ^{19}F NMR. After a 6 h reaction time, the crude reaction was purified by adsorbing directly onto a dry 5 g Teledyne Isco silica cartridge using vacuum followed by elution onto a pre-equilibrated 4 g silica flash column (0 - 40% ethyl acetate/hexanes) providing Z-tertiary trisubstituted allylic amine. The branched/linear (b/l > 99:1), and *E/Z* ratios of trisubstituted alkenes was determined by the crude ^{19}F NMR. The stereoselectivity of products was determined by 2D-ROESY.

3 Analytical Data of All Compounds

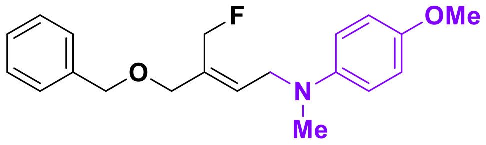

5

^{1}H NMR (500 MHz, $CDCl_3$) δ 7.39 – 7.33 (m, 5H), 7.30 (tdd, J = 9.6, 6.6, 2.1 Hz, 4H), 6.84 (q, J = 2.3 Hz, 2H), 6.75 – 6.72 (m, 2H), 5.82 (dp, J = 6.5, 3.1 Hz, 2H), 5.04 (d, J = 47.5 Hz, 2H), 4.55 (s, 2H), 4.16 (s, 2H), 3.98 (dd, J = 6.5, 4.0 Hz, 2H), 3.76 (s, 3H), 2.84 (s, 3H).

^{13}C NMR (126 MHz, $CDCl_3$) δ 138.14, 128.62 (d, J = 1.4 Hz), 128.55, 128.01, 127.97, 127.93, 127.89, 127.84, 127.81, 115.95, 114.88 (d, J = 1.8 Hz), 105.16, 84.99 (d, J = 166.0 Hz), 72.28, 65.07 (d, J = 1.6 Hz), 55.85, 51.29, 39.32 (d, J = 7.4 Hz).

^{19}F NMR (471 MHz, $CDCl_3$) δ -213.42, -215.77.

HRMS (ESI-TOF) m/z: $[M + H]^+$ Calc. for $C_{20}H_{25}FNO_2$ 330.4226; found 330.4226

Note: NMR contains a partially inseparable E-isomer in spectra.

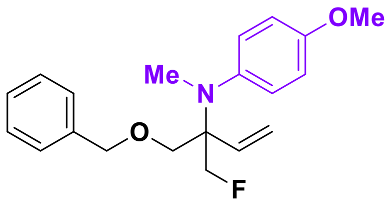

6

^{1}H NMR (500 MHz, CDCl$_3$) δ 7.36 – 7.28 (m, 5H), 7.14 (d, *J* = 8.4 Hz, 2H), 6.77 (d, *J* = 9.0 Hz, 2H), 5.83 (dd, *J* = 17.9, 11.2 Hz, 1H), 5.27 (d, *J* = 47.7 Hz, 2H), 4.64 (d, *J* = 5.7 Hz, 1H), 4.54 (d, *J* = 5.5 Hz, 1H), 4.52 (s, 2H), 3.78 (s, 3H), 3.56 (qd, *J* = 9.5, 2.3 Hz, 2H), 2.78 (s, 3H).

^{13}C NMR (126 MHz, CDCl$_3$) δ 156.93, 142.36, 138.30, 135.86, 128.99, 128.46, 127.73 (d, *J* = 3.1 Hz), 117.34, 115.09, 113.52, 83.57 (d, *J* = 176.1 Hz), 73.65, 70.58 (d, *J* = 4.5 Hz), 56.01, 55.50, 38.43.

^{19}F NMR (471 MHz, CDCl$_3$) δ -228.21.

HRMS (ESI-TOF) m/z: $[M + H]^+$ Calc. for $C_{20}H_{25}FNO_2$ 330.4226; found 330.4225

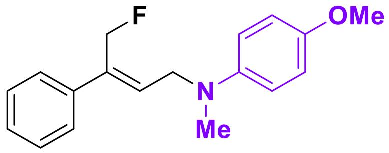

7

^{1}H NMR (500 MHz, CDCl$_3$) δ 7.23 – 7.17 (m, 2H), 7.17 – 7.02 (m, 4H), 6.68 – 6.57 (m, 4H), 5.90 (td, *J* = 6.4, 3.8 Hz, 1H), 5.14 (d, *J* = 47.8 Hz, 2H), 3.93 (dd, *J* = 6.5, 4.6 Hz, 2H), 3.57 (s, 3H), 2.70 (s, 3H).

^{13}C NMR (126 MHz, CDCl$_3$) δ 152.57, 144.42, 137.12 (d, *J* = 13.5 Hz), 132.58 (d, *J* = 7.7 Hz), 128.64, 127.77, 126.28, 115.95, 114.93, 79.69 (d, *J* = 164.6 Hz), 55.87, 52.07, 39.43.

^{19}F NMR (471 MHz, CDCl$_3$) δ -208.87.

HRMS (ESI-TOF) m/z: $[M + H]^+$ Calc. for $C_{18}H_{21}FNO$ 286.1601; found 286.1602

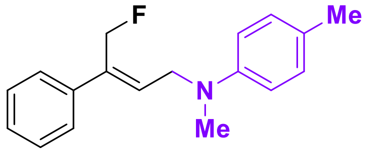

9

^{1}H NMR (500 MHz, CDCl$_3$) δ 7.34 (dq, *J* = 6.6, 1.2 Hz, 2H), 7.32 – 7.18 (m, 3H), 7.05 – 6.99 (m, 2H), 6.71 – 6.65 (m, 2H), 6.04 (td, *J* = 6.4, 3.8 Hz, 1H), 5.30 (d, *J* = 47.8 Hz, 2H), 4.13 (dd, *J* = 6.4, 4.6 Hz, 2H), 2.89 (s, 3H), 2.21 (s, 3H).

^{13}C NMR (126 MHz, CDCl$_3$) δ 147.53, 139.86 (d, *J* = 1.8 Hz), 137.04 (d, *J* = 13.6 Hz), 132.63 (d, *J* = 7.7 Hz), 129.94, 128.62, 127.76, 126.79, 126.27, 113.85, 79.69 (d, *J* = 164.7 Hz), 51.16 (d, *J* = 1.4 Hz), 38.74, 20.39.

^{19}F NMR (471 MHz, CDCl$_3$) δ -208.80.

HRMS (ESI-TOF) m/z: $[M + H]^+$ Calc. for $C_{18}H_{21}FN$ 270.1653; found 270.1652

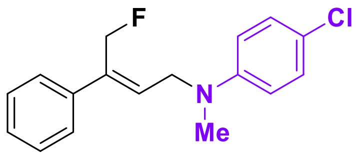

10

^{1}H NMR (500 MHz, $CDCl_3$) δ 7.33 (dt, *J* = 8.2, 1.2 Hz, 2H), 7.28 (ddd, *J* = 7.5, 6.6, 1.4 Hz, 2H), 7.25 – 7.20 (m, 1H), 7.15 – 7.10 (m, 2H), 6.66 – 6.60 (m, 2H), 5.97 (td, *J* = 6.4, 3.8 Hz, 1H), 5.29 (d, *J* = 47.8 Hz, 2H), 4.15 (dd, *J* = 6.3, 4.6 Hz, 2H), 2.90 (s, 3H).

^{13}C NMR (126 MHz, $CDCl_3$) δ 147.91, 139.55 (d, *J* = 1.9 Hz), 137.36 (d, *J* = 13.6 Hz), 131.68 (d, *J* = 7.5 Hz), 129.07, 128.57, 127.80, 126.13, 122.04, 114.23, 79.61 (d, *J* = 165.3 Hz), 50.78 (d, *J* = 0.9 Hz), 38.50.

^{19}F NMR (471 MHz, $CDCl_3$) δ -208.93.

HRMS (ESI-TOF) m/z: $[M + H]^+$ Calc. for $C_{17}H_{18}ClFN$ 290.1106; found 290.1108

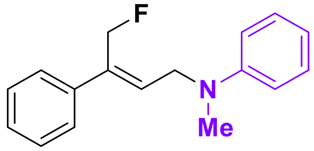

11

^{1}H NMR (500 MHz, $CDCl_3$) δ 7.46 – 7.36 (m, 2H), 7.36 – 7.31 (m, 2H), 7.31 – 7.26 (m, 3H), 6.84 – 6.73 (m, 3H), 6.09 (td, *J* = 6.3, 3.8 Hz, 1H), 5.37 (d, *J* = 47.8 Hz, 2H), 4.24 (dd, *J* = 6.3, 4.6 Hz, 2H), 2.99 (s, 3H).

^{13}C NMR (126 MHz, $CDCl_3$) δ 149.48, 139.81 (d, *J* = 1.8 Hz), 137.13 (d, *J* = 13.6 Hz), 132.50 (d, *J* = 7.6 Hz), 129.43, 128.65, 127.81, 126.27, 117.31, 113.28, 79.71 (d, *J* = 165.0 Hz), 50.80 (d, *J* = 1.2 Hz), 38.46.

^{19}F NMR (471 MHz, $CDCl_3$) δ -208.81.

HRMS (ESI-TOF) m/z: $[M + H]^+$ Calc. for $C_{17}H_{19}FN$ 256.1495; found 256.1494

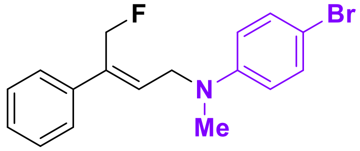

12

^{1}H NMR (500 MHz, $CDCl_3$) δ 7.38 – 7.12 (m, 8H), 6.58 (d, *J* = 9.0 Hz, 2H), 5.95 (td, *J* = 6.3, 3.8 Hz, 1H), 5.28 (d, *J* = 47.7 Hz, 2H), 4.13 (dd, *J* = 6.3, 4.5 Hz, 2H), 2.89 (s, 3H).

^{13}C NMR (126 MHz, $CDCl_3$) δ 148.36, 139.65 (d, *J* = 2.0 Hz), 137.53 (d, *J* = 13.6 Hz), 132.08, 128.70, 127.94, 126.25, 114.82, 79.75 (d, *J* = 165.4 Hz), 50.82, 38.57.

^{19}F NMR (471 MHz, $CDCl_3$) δ -208.93.

HRMS (ESI-TOF) m/z: $[M + H]^+$ Calc. for $C_{17}H_{18}BrFN$ 334.0600; found 332.0600.

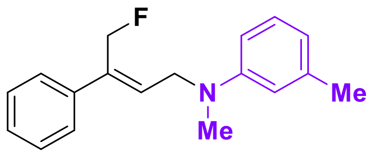

13

^{1}H NMR (500 MHz, $CDCl_3$) δ 7.36 – 7.31 (m, 2H), 7.29 – 7.24 (m, 2H), 7.24 – 7.19 (m, 1H), 7.11 – 7.05 (m, 1H), 6.58 – 6.50 (m, 3H), 6.02 (td, *J* = 6.4, 3.8 Hz, 1H), 5.30 (d, *J* = 47.8 Hz, 2H), 4.15 (dd, *J* = 6.4, 4.6 Hz, 2H), 2.90 (s, 3H), 2.26 (s, 3H).
^{13}C NMR (126 MHz, $CDCl_3$) δ 149.57, 139.84 (d, *J* = 1.8 Hz), 139.13, 137.03 (d, *J* = 13.6 Hz), 132.62 (d, *J* = 7.7 Hz), 129.28, 128.64, 127.79, 126.28, 118.29, 114.09, 110.51, 79.69 (d, *J* = 164.8 Hz), 50.82, 38.48, 22.05.
^{19}F NMR (471 MHz, $CDCl_3$) δ -208.74.
HRMS (ESI-TOF) m/z: $[M + H]^+$ Calc. for $C_{18}H_{21}FN$ 270.1653; found 270.1651

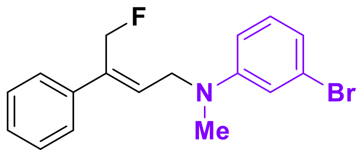

14

^{1}H NMR (500 MHz, $CDCl_3$) δ 7.43 – 7.27 (m, 6H), 7.09 (t, *J* = 8.1 Hz, 1H), 6.90 (t, *J* = 2.2 Hz, 1H), 6.86 (ddd, *J* = 7.8, 1.8, 0.8 Hz, 1H), 6.69 (ddd, *J* = 8.4, 2.5, 0.8 Hz, 1H), 6.02 (td, *J* = 6.4, 3.8 Hz, 1H), 5.36 (d, *J* = 47.7 Hz, 2H), 4.22 (dd, *J* = 6.4, 4.5 Hz, 2H), 2.98 (s, 3H).
^{13}C NMR (126 MHz, $CDCl_3$) δ 150.58, 139.66 (d, *J* = 2.0 Hz), 137.63 (d, *J* = 13.6 Hz), 131.52 (d, *J* = 7.4 Hz), 130.60, 128.70, 127.95, 126.29, 123.70, 119.88, 115.78, 111.55, 79.75 (d, *J* = 165.5 Hz), 50.57, 38.34.
^{19}F NMR (471 MHz, $CDCl_3$) δ -208.91.
HRMS (ESI-TOF) m/z: $[M + H]^+$ Calc. $C_{17}H_{18}BrFN$ 334.0600; found 334.0600

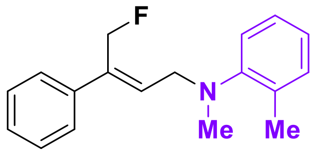

15

^{1}H NMR (500 MHz, $CDCl_3$) δ 7.36 – 7.31 (m, 2H), 7.31 – 7.24 (m, 2H), 7.24 – 7.14 (m, 1H), 7.10 (ddd, *J* = 15.4, 7.6, 1.7 Hz, 2H), 7.04 – 6.97 (m, 1H), 6.91 (td, *J* = 7.4, 1.3 Hz, 1H), 6.09 (td, *J* = 6.7, 3.6 Hz, 1H), 5.21 (d, *J* = 47.8 Hz, 2H), 3.71 (dd, *J* = 6.7, 4.4 Hz, 2H), 2.65 (s, 3H), 2.27 (s, 3H).
^{13}C NMR (126 MHz, $CDCl_3$) δ 151.62, 140.08, 137.12 (d, *J* = 13.7 Hz), 133.04, 131.35, 128.63, 127.72, 126.57, 126.35, 123.36, 120.06, 79.62 (d, *J* = 164.1 Hz), 53.98, 41.46, 18.45.
^{19}F NMR (471 MHz, $CDCl_3$) δ -208.90.
HRMS (ESI-TOF) m/z: $[M + H]^+$ Calc. for $C_{18}H_{21}FN$ 270.1653; found 270.1650.

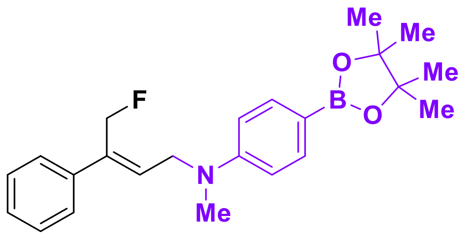

16

^{1}H NMR (500 MHz, $CDCl_3$) δ 7.66 – 7.60 (m, 2H), 7.30 (dt, J = 8.2, 1.3 Hz, 2H), 7.28 – 7.21 (m, 2H), 7.21 – 7.16 (m, 1H), 6.70 – 6.64 (m, 2H), 5.96 (td, J = 6.3, 3.8 Hz, 1H), 5.28 (d, J = 47.7 Hz, 2H), 4.20 (dd, J = 6.3, 4.5 Hz, 2H), 2.94 (s, 3H), 1.25 (s, 12H).
^{13}C NMR (126 MHz, $CDCl_3$) δ 151.46, 139.70 (d, J = 1.9 Hz), 137.33 (d, J = 13.6 Hz), 136.49, 131.96 (d, J = 7.5 Hz), 128.65, 127.86, 126.27, 111.86, 83.38, 79.74 (d, J = 165.2 Hz), 50.24, 38.18, 24.99.
^{19}F NMR (471 MHz, $CDCl_3$) δ -208.80.
HRMS (ESI-TOF) m/z: $[M + H]^+$ Calc. for $C_{23}H_{30}BFNO_2$ 382.2347; found 382.2348

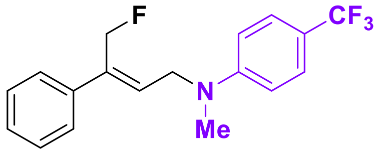

17

^{1}H NMR (500 MHz, $CDCl_3$) δ 7.50 – 7.43 (m, 2H), 7.41 – 7.27 (m, 5H), 6.80 – 6.74 (m, 2H), 6.01 (td, J = 6.3, 3.8 Hz, 1H), 5.32 (d, J = 47.7 Hz, 2H), 4.30 (dd, J = 6.3, 4.5 Hz, 2H), 3.06 (s, 3H).
^{13}C NMR (126 MHz, $CDCl_3$) δ 151.34 (d, J = 1.2 Hz), 139.55 (d, J = 2.1 Hz), 137.79 (d, J = 13.5 Hz), 131.26 (d, J = 7.3 Hz), 128.04, 126.72 (q, J = 3.8 Hz), 126.27, 118.40 (q, J = 32.7 Hz), 111.82, 79.83 (d, J = 165.7 Hz), 50.42, 38.37.
^{19}F NMR (471 MHz, $CDCl_3$) δ -209.03, -210.21.
HRMS (ESI-TOF) m/z: $[M + H]^+$ Calc. for $C_{18}H_{18}F_4N$ 324.1369; found 324.1366

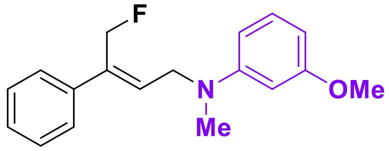

18

^{1}H NMR (500 MHz, $CDCl_3$) δ 7.33 (dt, J = 8.2, 1.1 Hz, 2H), 7.29 – 7.24 (m, 2H), 7.24 – 7.20 (m, 1H), 7.13 – 7.06 (m, 1H), 6.37 – 6.30 (m, 1H), 6.29 – 6.22 (m, 2H), 6.01 (td, J = 6.3, 3.8 Hz, 1H), 5.29 (d, J = 47.8 Hz, 2H), 4.16 (dd, J = 6.4, 4.6 Hz, 2H), 3.73 (s, 3H), 2.91 (s, 3H).

^{13}C NMR (126 MHz, $CDCl_3$) δ 160.98, 150.84, 139.79 (d, J = 1.8 Hz), 137.10 (d, J = 13.9 Hz), 132.44 (d, J = 7.7 Hz), 130.12, 127.82, 126.28, 106.27, 102.15, 99.77, 79.72 (d, J = 165.0 Hz), 55.29, 50.83, 38.54.
^{19}F NMR (471 MHz, $CDCl_3$) δ -208.79.

HRMS (ESI-TOF) m/z: $[M + H]^+$ Calc. for $C_{18}H_{21}FNO$ 286.1601; found 286.1601

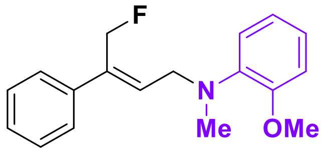

19

^{1}H NMR (500 MHz, $CDCl_3$) δ 7.36 – 7.32 (m, 2H), 7.32 – 7.23 (m, 2H), 7.23 – 7.17 (m, 1H), 6.98 – 6.89 (m, 2H), 6.89 – 6.78 (m, 2H), 6.12 (td, *J* = 6.9, 3.6 Hz, 1H), 5.22 (d, *J* = 47.9 Hz, 2H), 3.94 (dd, *J* = 6.9, 4.3 Hz, 2H), 3.83 (s, 3H), 2.78 (s, 3H).
^{13}C NMR (126 MHz, $CDCl_3$) δ 152.59, 140.71, 140.09 (d, *J* = 1.4 Hz), 137.14 (d, *J* = 13.6 Hz), 132.50 (d, *J* = 8.1 Hz), 128.57, 127.64, 126.32, 123.05, 120.97, 119.65, 111.33, 79.50 (d, *J* = 163.7 Hz), 55.54, 52.67 (d, *J* = 2.0 Hz), 40.16.
^{19}F NMR (471 MHz, $CDCl_3$) δ -208.66
HRMS (ESI-TOF) m/z: $[M + H]^+$ Calc. for $C_{18}H_{21}FNO$ 286.1601; found 286.1600

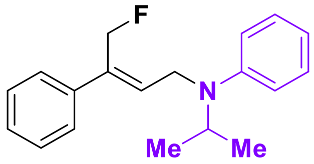

20

^{1}H NMR (500 MHz, $CDCl_3$) δ 7.41 – 7.20 (m, 8H), 6.79 (d, *J* = 8.2 Hz, 2H), 6.72 (t, *J* = 7.2 Hz, 1H), 6.05 (td, *J* = 5.5, 3.8 Hz, 1H), 5.40 (d, *J* = 47.8 Hz, 2H), 4.15 (dt, *J* = 8.5, 6.0 Hz, 3H), 1.24 (d, *J* = 6.6 Hz, 6H).
^{13}C NMR (126 MHz, $CDCl_3$) δ 148.82, 139.83, 136.56 (d, *J* = 7.6 Hz), 135.04 (d, *J* = 13.5 Hz), 129.44 (d, *J* = 7.9 Hz), 128.64, 127.65, 126.23, 116.87, 113.40, 79.89 (d, *J* = 164.8 Hz), 48.66, 42.26, 20.44.
^{19}F NMR (471 MHz, $CDCl_3$) δ -210.12, -213.04.
HRMS (ESI-TOF) m/z: $[M + H]^+$ Calc. for $C_{19}H_{23}FN$ 284.1808; found 284.1805.

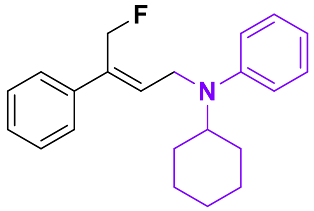

21

^{1}H NMR (500 MHz, $CDCl_3$) δ 7.33 – 7.29 (m, 2H), 7.27 – 7.21 (m, 2H), 7.19 – 7.09 (m, 3H), 6.65 (dd, *J* = 18.1, 1.1 Hz, 1H), 6.61 (dt, *J* = 7.3, 1.0 Hz, 1H), 5.95 (td, *J* = 5.5, 3.8 Hz, 1H), 5.31 (d, *J* = 47.8 Hz, 2H), 4.09 (t, *J* = 5.5 Hz, 2H), 3.57 (ddt, *J* = 11.4, 7.0, 3.4 Hz, 1H), 1.80 (dd, *J* = 23.3, 12.3 Hz, 4H), 1.67 – 1.56 (m, 1H), 1.41 – 1.24 (m, 4H), 1.08 (qt, *J* = 12.8, 3.8 Hz, 1H).

^{13}C NMR (126 MHz, CDCl$_3$) δ 148.72, 139.81 (d, J = 2.0 Hz), 136.73 (d, J = 7.6 Hz), 134.88 (d, J = 13.7 Hz), 129.46, 128.62, 127.62, 126.20, 116.66, 113.08, 79.85 (d, J = 164.8 Hz), 57.66, 43.19 (d, J = 1.2 Hz), 26.38, 26.03.
^{19}F NMR (471 MHz, CDCl$_3$) δ -210.03, -212.99.
HRMS (ESI-TOF) m/z: $[M + H]^+$ Calc. for $C_{22}H_{27}FN$ 324.2121; found 324.2120.

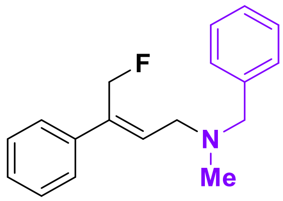

22

^{1}H NMR (500 MHz, CDCl$_3$) δ 7.36 (dt, J = 8.3, 1.2 Hz, 2H), 7.32 – 7.15 (m, 9H), 6.14 (td, J = 6.9, 3.6 Hz, 1H), 5.22 (d, J = 47.8 Hz, 2H), 3.50 (s, 2H), 3.23 (dd, J = 6.9, 4.4 Hz, 2H), 2.20 (s, 3H).
^{13}C NMR (126 MHz, CDCl$_3$) δ 140.12 (d, J = 1.3 Hz), 138.84, 137.34 (d, J = 13.8 Hz), 133.15, 129.21, 128.63, 128.47, 127.71, 127.30, 126.33, 79.64 (d, J = 163.7 Hz), 62.24, 54.84 (d, J = 2.2 Hz), 42.62.
^{19}F NMR (471 MHz, CDCl$_3$) δ -209.06.
HRMS (ESI-TOF) m/z: $[M + H]^+$ Calc. for $C_{18}H_{21}FN$ 270.1652; found 270.1654

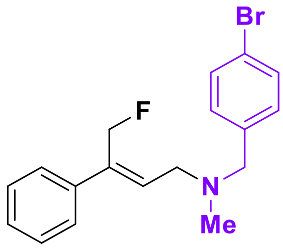

23

^{1}H NMR (500 MHz, CDCl$_3$) δ 7.41 – 7.33 (m, 4H), 7.31 – 7.12 (m, 6H), 6.11 (td, J = 6.9, 3.6 Hz, 1H), 5.21 (d, J = 47.8 Hz, 2H), 3.44 (s, 2H), 3.22 (dd, J = 6.9, 4.4 Hz, 2H), 2.18 (s, 3H).
^{13}C NMR (126 MHz, CDCl$_3$) δ 140.02, 137.97, 137.53 (d, J = 13.5 Hz), 132.83, 131.58, 130.81, 128.66, 127.78, 126.31, 121.10, 79.62 (d, J = 164.0 Hz), 61.45, 54.87 (d, J = 2.1 Hz), 42.55.
^{19}F NMR (471 MHz, CDCl$_3$) δ -209.01.
HRMS (ESI-TOF) m/z: $[M + H]^+$ Calc. for $C_{18}H_{20}BrFN$ 348.0757; found 348.0752.

1 g scale reaction of substrate 4

A 50 mL oven-dried Schlenk flask was charged with $[RhCl(COD)_2]_2$ (79.38 mg, 5 mol%) in a glove box. A separate 50 mL Schlenk flask was charged with allylic imidate **1** (1 g, 3.22 mmol, 1.0 equiv.), toluene (32 mL), and amine **2** (966.35 mg, 4.83 mmol, 1.5 equiv.). The rhodium catalyst solution was then transferred to the flask containing the catalyst *via* a cannula. The reaction mixture was stirred at room temperature under an argon atmosphere. Reaction progress was monitored by TLC and both ^{1}H and ^{19}F NMR. After a 6 h reaction time, the crude reaction was purified by adsorbing directly onto a dry 5 g Teledyne Isco silica cartridge using vacuum followed by elution onto a pre-equilibrated 12 g silica flash

column (0 - 40% ethyl acetate/hexanes) providing Z-tertiary trisubstituted allylic amine **4** (720 mg, 64 %) as a pale-yellow oil. The branched/linear (*b/l* > 99:1) and *E/Z* ratios (*E/Z* > 1:99) of *Z*-trisubstituted alkene was determined by the crude ^{19}F NMR.

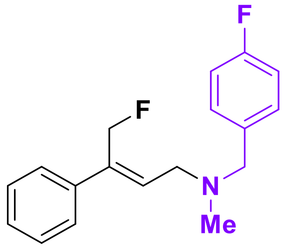

24

^{1}H NMR (500 MHz, $CDCl_3$) δ 7.44 (dq, *J* = 6.7, 1.2 Hz, 2H), 7.39 – 7.26 (m, 6H), 7.02 (t, *J* = 8.7 Hz, 2H), 6.20 (td, *J* = 6.9, 3.6 Hz, 1H), 5.29 (d, *J* = 47.8 Hz, 2H), 3.54 (s, 2H), 3.31 (s, 2H), 2.27 (s, 3H).
^{13}C NMR (126 MHz, $CDCl_3$) δ 162.25 (d, *J* = 245.3 Hz), 140.02, 130.71 (d, *J* = 7.9 Hz), 128.66, 127.80, 126.32, 115.22, 79.66 (d, *J* = 163.9 Hz), 61.33, 54.76, 42.41.
^{19}F NMR (471 MHz, $CDCl_3$) δ -115.76, -209.06.
HRMS (ESI-TOF) m/z: $[M + H]^+$ Calc. for $C_{18}H_{20}F_2N$ 288.1558; found 288.1554.

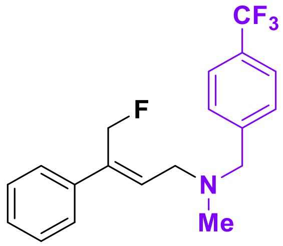

25

^{1}H NMR (500 MHz, $CDCl_3$) δ 7.49 (d, *J* = 8.0 Hz, 2H), 7.41 – 7.31 (m, 4H), 7.31 – 7.09 (m, 4H), 6.10 (td, *J* = 6.9, 3.6 Hz, 1H), 5.21 (d, *J* = 47.8 Hz, 2H), 3.52 (s, 2H), 3.23 (dd, *J* = 6.9, 4.4 Hz, 2H), 2.19 (s, 3H).
^{13}C NMR (126 MHz, $CDCl_3$) δ 143.29, 140.01 (d, *J* = 1.4 Hz), 137.61 (d, *J* = 13.6 Hz), 132.77 (d, *J* = 7.9 Hz), 129.24, 128.67, 127.81, 126.31, 125.41 (q, *J* = 3.8 Hz), 123.31, 79.61 (d, *J* = 164.0 Hz), 61.64, 55.05 (d, *J* = 2.1 Hz), 42.69.
^{19}F NMR (471 MHz, $CDCl_3$) δ -62.40, -208.99.
HRMS (ESI-TOF) m/z: $[M + H]^+$ Calc. for $C_{19}H_{20}F_4N$ 338.1526; found 338.1522.

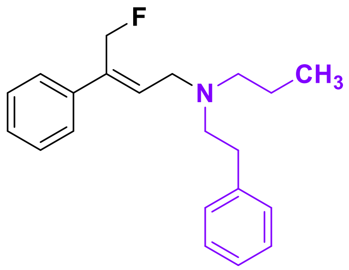

26

^{1}H NMR (500 MHz, CDCl$_3$) δ 7.42 (dd, *J* = 7.4, 1.4 Hz, 2H), 7.35 (dd, *J* = 8.4, 6.8 Hz, 2H), 7.31 – 7.26 (m, 3H), 7.22 – 7.17 (m, 3H), 6.17 (td, *J* = 6.7, 3.7 Hz, 1H), 5.30 (d, *J* = 47.9 Hz, 2H), 3.44 (t, *J* = 5.8 Hz, 2H), 2.78 (s, 4H), 2.53 (t, *J* = 7.6 Hz, 2H), 1.54 (h, *J* = 7.3 Hz, 2H), 0.91 (t, *J* = 7.3 Hz, 3H).
^{13}C NMR (126 MHz, CDCl$_3$) δ 140.74, 140.21, 133.82, 128.91, 128.63, 128.50, 127.63, 126.28, 126.10, 79.68 (d, *J* = 163.7 Hz), 56.24 (d, *J* = 18.5 Hz), 51.84, 33.80, 31.07, 20.61, 12.08.
^{19}F NMR (471 MHz, CDCl$_3$) δ -208.41.
HRMS (ESI-TOF) m/z: $[M + H]^+$ Calc. for $C_{21}H_{27}FN$ 312.2121; found 312.2125

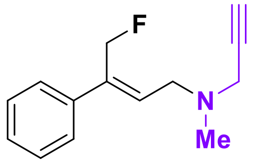

27

^{1}H NMR (500 MHz, CDCl$_3$) δ 7.45 (dt, *J* = 8.2, 1.2 Hz, 2H), 7.39 – 7.32 (m, 2H), 7.32 – 7.26 (m, 1H), 6.13 (td, *J* = 7.1, 3.5 Hz, 1H), 5.35 (d, *J* = 47.8 Hz, 2H), 3.39 (d, *J* = 2.4 Hz, 2H), 3.35 (dd, *J* = 7.1, 4.3 Hz, 2H), 2.39 (s, 3H), 2.28 (t, *J* = 2.4 Hz, 1H).
^{13}C NMR (126 MHz, CDCl$_3$) δ 139.95 (d, *J* = 1.3 Hz), 138.04 (d, *J* = 13.6 Hz), 132.19 (d, *J* = 7.9 Hz), 128.66, 127.82, 126.33, 79.57 (d, *J* = 163.6 Hz), 78.46, 73.68, 53.14 (d, *J* = 2.2 Hz), 45.38, 42.00.
^{19}F NMR (471 MHz, CDCl$_3$) δ -208.77.
HRMS (ESI-TOF) m/z: $[M + H]^+$ Calc. for $C_{14}H_{17}FN$ 218.1339; found 218.1338.

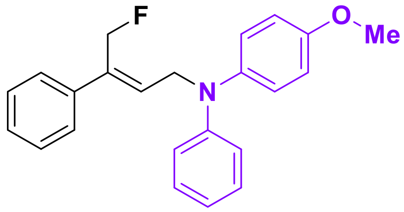

28

^{1}H NMR (500 MHz, CDCl$_3$) δ 7.39 – 7.29 (m, 4H), 7.29 – 7.26 (m, 1H), 7.23 – 7.15 (m, 2H), 7.14 – 7.09 (m, 2H), 6.92 – 6.88 (m, 2H), 6.83 – 6.76 (m, 3H), 6.23 (td, *J* = 6.2, 3.6 Hz, 1H), 5.23 (d, *J* = 47.8 Hz, 2H), 4.57 (dd, *J* = 6.2, 4.7 Hz, 2H), 3.82 (s, 3H).

^{13}C NMR (126 MHz, CDCl$_3$) δ 156.90, 148.74, 140.59, 139.79 (d, J = 1.9 Hz), 136.74 (d, J = 13.7 Hz), 132.72 (d, J = 7.5 Hz), 129.28, 129.10, 128.64, 127.80, 127.48, 126.32, 118.75, 116.04, 115.13, 79.79 (d, J = 165.1 Hz), 55.65, 50.57.
^{19}F NMR (471 MHz, CDCl$_3$) δ -209.76.
HRMS (ESI-TOF) m/z: $[M + H]^+$ Calc. $C_{23}H_{23}FNO$ 348.1757; found 348.1756

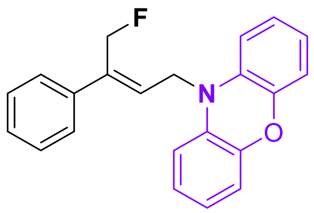

29

^{1}H NMR (500 MHz, CDCl$_3$) δ 7.44 – 7.28 (m, 5H), 6.83 (ddd, J = 7.9, 5.4, 3.6 Hz, 2H), 6.74 – 6.70 (m, 4H), 6.56 – 6.49 (m, 2H), 6.02 (td, J = 5.5, 3.7 Hz, 1H), 5.47 (d, J = 47.6 Hz, 2H), 4.52 (t, J = 5.3 Hz, 2H).
^{13}C NMR (126 MHz, CDCl$_3$) δ 145.64, 139.08 (d, J = 2.6 Hz), 138.51 (d, J = 13.8 Hz), 133.77, 131.13 (d, J = 6.8 Hz), 128.75, 128.14, 126.23, 123.97, 121.48, 115.58, 111.81, 80.14 (d, J = 166.5 Hz), 42.77.
^{19}F NMR (471 MHz, CDCl$_3$) δ -210.10, -211.04.
HRMS (ESI-TOF) m/z: $[M + H]^+$ Calc. for $C_{22}H_{19}FNO$ 332.1444; found 332.1448

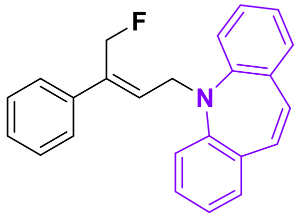

30

^{1}H NMR (500 MHz, CDCl$_3$) δ 7.31 – 7.22 (m, 7H), 7.10 (dd, J = 7.5, 1.7 Hz, 2H), 7.02 (ddt, J = 8.6, 7.4, 1.8 Hz, 4H), 6.78 (s, 2H), 6.01 (td, J = 5.9, 3.6 Hz, 1H), 5.41 (d, J = 47.8 Hz, 2H), 4.66 (dd, J = 5.9, 4.6 Hz, 2H).
^{13}C NMR (126 MHz, CDCl$_3$) δ 150.43, 139.86 (d, J = 1.8 Hz), 136.75 (d, J = 13.7 Hz), 133.90, 132.49 (d, J = 7.5 Hz), 132.28, 129.41, 129.01, 128.51, 127.65, 126.41, 123.83, 120.58, 80.25 (d, J = 164.7 Hz), 48.85 (d, J = 1.2 Hz).
^{19}F NMR (471 MHz, CDCl$_3$) δ -210.30.
HRMS (ESI-TOF) m/z: $[M + H]^+$ Calc. for $C_{24}H_{21}FN$ 342.1652; found 342.1651.

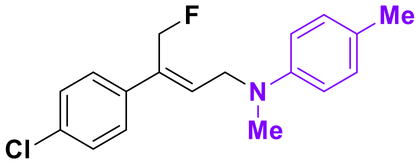

31

^{1}H NMR (500 MHz, $CDCl_3$) δ 7.31 (q, *J* = 8.7 Hz, 5H), 7.10 – 7.04 (m, 2H), 6.76 – 6.69 (m, 2H), 6.08 (td, *J* = 6.3, 3.6 Hz, 1H), 5.32 (d, *J* = 47.8 Hz, 2H), 4.17 (dd, *J* = 6.3, 4.6 Hz, 2H), 2.94 (s, 3H), 2.27 (s, 3H).

^{13}C NMR (126 MHz, $CDCl_3$) δ 147.68, 138.49 (d, *J* = 1.6 Hz), 136.28 (d, *J* = 13.9 Hz), 133.95, 133.39 (d, *J* = 7.6 Hz), 130.23, 129.03, 127.88, 127.22, 114.12, 79.64 (d, *J* = 165.0 Hz), 51.42, 39.08, 20.65.

^{19}F NMR (471 MHz, $CDCl_3$) δ -209.26.

HRMS (ESI-TOF) m/z: $[M + H]^+$ Calc. for $C_{18}H_{20}ClFN$ 304.1262; found 304.1260

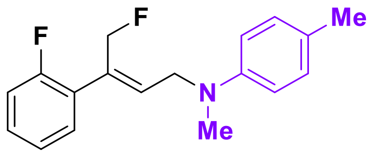

32

^{1}H NMR (500 MHz, $CDCl_3$) δ 7.27 (dd, *J* = 8.4, 6.5 Hz, 1H), 7.25 – 7.20 (m, 1H), 7.11 – 7.00 (m, 4H), 6.73 (d, *J* = 8.4 Hz, 2H), 5.95 (td, *J* = 6.3, 3.3 Hz, 1H), 5.31 (d, *J* = 47.5 Hz, 2H), 4.18 (dd, *J* = 6.3, 4.2 Hz, 2H), 2.95 (s, 3H), 2.26 (s, 3H).

^{13}C NMR (126 MHz, $CDCl_3$) δ 159.91 (d, *J* = 246.8 Hz), 147.47, 135.07, 133.72, 130.47 (d, *J* = 3.9 Hz), 129.93, 129.47 (d, *J* = 8.4 Hz), 128.51 (d, *J* = 27.2 Hz), 127.89 (d, *J* = 2.8 Hz), 126.85, 124.35 (d, *J* = 3.6 Hz), 115.77 (d, *J* = 22.4 Hz), 113.89, 80.15 (d, *J* = 170.1 Hz), 51.02, 38.75, 20.39.

^{19}F NMR (471 MHz, $CDCl_3$) δ -115.75, -210.45.

HRMS (ESI-TOF) m/z: $[M + H]^+$ Calc. for $C_{18}H_{20}F_2N$ 288.1558; found 288.1555

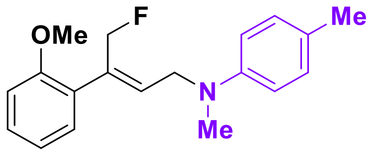

33

^{1}H NMR (500 MHz, $CDCl_3$) δ 7.25 (d, *J* = 7.1 Hz, 1H), 7.15 (dd, *J* = 7.5, 1.8 Hz, 1H), 7.10 – 7.04 (m, 2H), 6.93 – 6.83 (m, 2H), 6.74 (d, *J* = 8.1 Hz, 2H), 5.82 (td, *J* = 6.3, 3.6 Hz, 1H), 5.31 (d, *J* = 47.7 Hz, 2H), 4.17 (dd, *J* = 6.3, 4.3 Hz, 2H), 3.82 (s, 3H), 2.95 (s, 3H), 2.26 (s, 3H).

^{13}C NMR (126 MHz, $CDCl_3$) δ 156.75 (d, *J* = 23.9 Hz), 147.65, 137.11 (d, *J* = 15.0 Hz), 133.24, 130.92, 130.55, 129.86, 129.73, 129.15, 126.54, 120.91, 113.82, 110.73, 80.64 (d, *J* = 164.2 Hz), 55.58, 51.04, 38.68, 20.39.

^{19}F NMR (471 MHz, $CDCl_3$) δ -209.36, -210.50.

HRMS (ESI-TOF) m/z: $[M + H]^+$ Calc. for $C_{19}H_{23}FNO$ 300.1757; found 300.1754

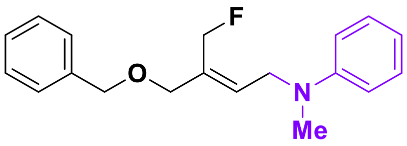

34

^{1}H NMR (500 MHz, CDCl$_3$) δ 7.37 (d, J = 3.3 Hz, 5H), 7.25 – 7.17 (m, 2H), 6.74 (s, 3H), 5.81 (d, J = 5.2 Hz, 1H), 4.87 (dd, J = 47.4, 1.2 Hz, 2H), 4.56 (s, 2H), 4.17 (s, 2H), 4.03 (t, J = 5.6 Hz, 2H), 2.90 (s, 3H).
^{13}C NMR (126 MHz, CDCl$_3$) δ 149.39, 138.08 (d, J = 6.6 Hz), 131.48 (d, J = 32.7 Hz), 128.66, 128.00, 127.89 (d, J = 5.3 Hz), 117.21, 113.19, 84.98 (d, J = 166.1 Hz), 72.97, 72.37, 65.09 (d, J = 1.6 Hz), 50.03, 38.38.
^{19}F NMR (471 MHz, CDCl$_3$) δ -213.42, -213.43.
HRMS (ESI-TOF) m/z: $[M + H]^+$ Calc. for $C_{19}H_{23}FNO$ 300.1757; found 300.1756
Note: NMR contains a minor partially inseparable E-isomer

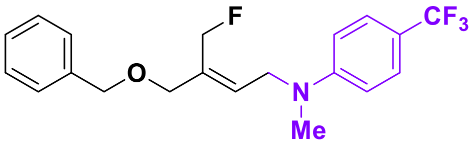

35

^{1}H NMR (500 MHz, CDCl$_3$) δ 7.46 – 7.40 (m, 2H), 7.39 (d, J = 7.9 Hz, 5H), 6.69 (d, J = 8.7 Hz, 2H), 5.74 (tdt, J = 6.2, 4.1, 1.0 Hz, 1H), 4.87 (dq, J = 47.3, 1.1 Hz, 2H), 4.57 (s, 2H), 4.17 (s, 2H), 4.08 (t, J = 5.5 Hz, 2H), 2.96 (s, 3H).
^{13}C NMR (126 MHz, CDCl$_3$) δ 151.27, 137.90, 135.30 (d, J = 14.4 Hz), 129.94 (d, J = 10.4 Hz), 128.70, 128.11, 127.95, 126.66 (q, J = 3.8 Hz), 111.74, 84.83 (d, J = 166.9 Hz), 73.15, 65.11 (d, J = 1.8 Hz), 49.66, 38.28.
^{19}F NMR (471 MHz, CDCl$_3$) δ -60.99, -214.10.
HRMS (ESI-TOF) m/z: $[M + H]^+$ Calc. for $C_{20}H_{22}F_4NO$ 368.1631; found 368.1633

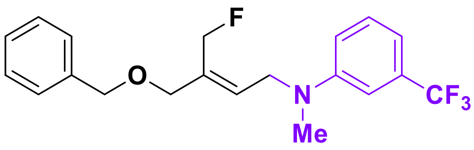

36

^{1}H NMR (500 MHz, CDCl$_3$) δ 7.39 – 7.34 (m, 4H), 6.95 (dd, J = 7.9, 1.3 Hz, 1H), 6.89 (t, J = 2.1 Hz, 1H), 6.84 (dd, J = 8.4, 2.6 Hz, 1H), 5.75 (dd, J = 5.1, 1.1 Hz, 1H), 4.87 (dt, J = 47.3, 1.1 Hz, 2H), 4.57 (s, 2H), 4.17 (s, 2H), 4.06 (t, J = 5.5 Hz, 2H), 2.94 (s, 3H).
^{13}C NMR (126 MHz, CDCl$_3$) δ 149.17, 137.79, 135.22 (d, J = 14.2 Hz), 131.55 (d, J = 31.5 Hz), 129.62, 128.54, 128.41, 127.92, 127.79, 127.59, 115.69, 113.32, 109.00, 84.69 (d, J = 166.8 Hz), 72.93, 64.96 (d, J = 1.9 Hz), 49.73, 38.19.
^{19}F NMR (471 MHz, CDCl$_3$) δ -62.76, -214.17.

HRMS (ESI-TOF) m/z: $[M + H]^+$ Calc. for $C_{20}H_{22}F_4NO$ 368.1631; found 368.1630

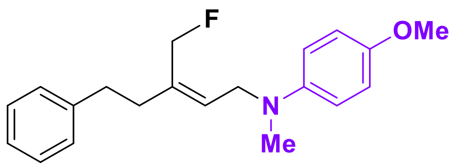

37

^{1}H NMR (500 MHz, CDCl$_3$) δ 7.33 – 7.28 (m, 2H), 7.25 – 7.17 (m, 3H), 6.83 – 6.79 (m, 2H), 6.67 – 6.63 (m, 2H), 5.61 – 5.55 (m, 1H), 4.78 (dq, J = 47.6, 1.0 Hz, 2H), 3.76 (s, 3H), 3.64 (t, J = 5.7 Hz, 2H), 2.78 (dd, J = 8.6, 6.9 Hz, 2H), 2.73 (s, 3H), 2.51 (dd, J = 8.6, 6.9 Hz, 2H).
^{13}C NMR (126 MHz, CDCl$_3$) δ 152.31 (d, J = 11.3 Hz), 144.45, 141.50, 136.69 (d, J = 14.3 Hz), 128.75, 128.63, 126.30, 115.67, 114.85, 86.80 (d, J = 165.7 Hz), 55.88, 51.16, 39.21, 34.54 (d, J = 1.2 Hz), 30.34 (d, J = 1.4 Hz).
^{19}F NMR (471 MHz, CDCl$_3$) δ -210.92, -214.84.
HRMS (ESI-TOF) m/z: $[M + H]^+$ Calc. for $C_{20}H_{25}FNO$ 314.1914; found 314.1911.

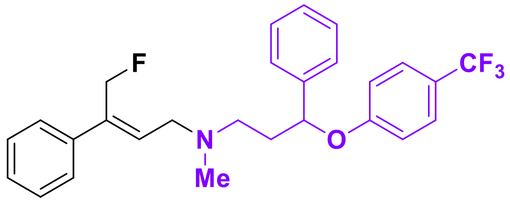

38

^{1}H NMR (500 MHz, CDCl$_3$) δ 7.40 – 7.21 (m, 9H), 7.09 (dd, J = 7.4, 1.8 Hz, 1H), 6.93 – 6.83 (m, 4H), 5.80 (td, J = 6.8, 3.5 Hz, 1H), 5.32 (dd, J = 8.3, 4.8 Hz, 1H), 5.22 (d, J = 47.7 Hz, 2H), 3.79 (s, 3H), 3.28 – 3.22 (m, 2H), 2.64 (dt, J = 12.4, 7.2 Hz, 1H), 2.49 (ddd, J = 12.7, 7.6, 5.4 Hz, 1H), 2.30 (s, 3H), 2.23 – 2.16 (m, 1H), 2.04 – 1.97 (m, 1H).
^{13}C NMR (126 MHz, CDCl$_3$) δ 160.79, 141.31, 139.99, 128.92, 128.62, 127.97, 127.74, 126.88 (q, J = 3.8 Hz), 126.24, 126.01, 115.89, 79.65 (d, J = 164.0 Hz), 78.27, 55.59 (d, J = 2.1 Hz), 53.32, 42.51, 36.70.
^{19}F NMR (471 MHz, CDCl$_3$) δ -61.54, -208.82.
HRMS (ESI-TOF) m/z: $[M + H]^+$ Calc. for $C_{27}H_{28}F_4NO$ 458.2101; found 458.2105

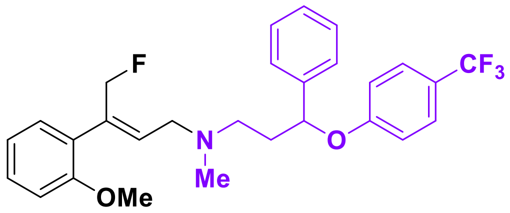

39

^{1}H NMR (500 MHz, CDCl$_3$) δ 7.40 – 7.21 (m, 9H), 7.09 (dd, *J* = 7.4, 1.8 Hz, 1H), 6.93 – 6.83 (m, 4H), 5.80 (td, *J* = 6.8, 3.5 Hz, 1H), 5.32 (dd, *J* = 8.3, 4.8 Hz, 1H), 5.22 (d, *J* = 47.7 Hz, 2H), 3.79 (s, 3H), 3.28 – 3.22 (m, 2H), 2.64 (dt, *J* = 12.4, 7.2 Hz, 1H), 2.49 (ddd, *J* = 12.7, 7.6, 5.4 Hz, 1H), 2.30 (s, 3H), 2.23 – 2.16 (m, 1H), 2.04 – 1.97 (m, 1H).
^{13}C NMR (126 MHz, CDCl$_3$) δ 160.84, 156.68, 141.39, 137.14 (d, *J* = 14.8 Hz), 133.84 (d, *J* = 6.7 Hz), 130.51, 130.14, 129.09, 128.89, 127.92, 126.84 (q, *J* = 3.8 Hz), 126.03, 120.91, 115.91, 110.71, 80.48 (d, *J* = 163.2 Hz), 78.47, 55.54, 55.37, 53.38, 42.53, 36.75.
^{19}F NMR (471 MHz, CDCl$_3$) δ -61.54, -209.51.
HRMS (ESI-TOF) m/z: $[M + H]^+$ Calc. for $C_{28}H_{30}F_4NO_2$ 488.2206; found 488.2201.

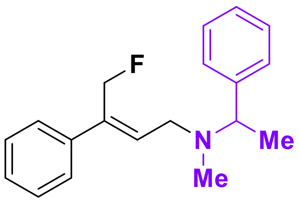

40

^{1}H NMR (500 MHz, CDCl$_3$) δ 7.35 (dt, *J* = 8.3, 1.2 Hz, 2H), 7.29 – 7.24 (m, 6H), 7.22 – 7.19 (m, 1H), 7.19 – 7.15 (m, 2H), 6.10 (ddd, *J* = 7.3, 6.4, 3.6 Hz, 1H), 5.21 – 5.15 (m, 1H), 5.11 – 5.05 (m, 1H), 3.53 (q, *J* = 6.7 Hz, 1H), 3.22 (ddd, *J* = 14.6, 6.3, 5.0 Hz, 1H), 3.10 (ddd, *J* = 14.6, 7.4, 4.3 Hz, 1H), 2.19 (s, 3H), 1.33 (d, *J* = 6.7 Hz, 3H).
^{1}H NMR (500 MHz, CDCl$_3$) δ 7.35 (dt, *J* = 8.3, 1.2 Hz, 2H), 7.29 – 7.24 (m, 6H), 7.22 – 7.19 (m, 1H), 7.19 – 7.15 (m, 2H), 6.10 (ddd, *J* = 7.3, 6.4, 3.6 Hz, 1H), 5.13 (ddd, *J* = 47.8 Hz, 2H), 3.53 (q, *J* = 6.7 Hz, 1H), 3.22 (ddd, *J* = 14.6, 6.3, 5.0 Hz, 1H), 3.10 (ddd, *J* = 14.6, 7.4, 4.3 Hz, 1H), 2.19 (s, 3H), 1.33 (d, *J* = 6.7 Hz, 3H).
^{13}C NMR (126 MHz, CDCl$_3$) δ 143.86, 140.22 (d, *J* = 1.3 Hz), 136.79 (d, *J* = 13.6 Hz), 133.79 (d, *J* = 8.1 Hz), 128.60, 128.45, 127.78, 127.61, 127.16, 126.29, 79.58 (d, *J* = 163.4 Hz), 63.65, 52.35 (d, *J* = 2.3 Hz), 39.12, 19.29.
^{19}F NMR (471 MHz, CDCl$_3$) δ -208.80.
HRMS (ESI-TOF) m/z: $[M + H]^+$ Calc. for $C_{19}H_{23}FN$ 284.1808; found 284.1806

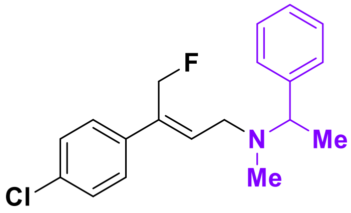

41

^{1}H NMR (500 MHz, CDCl$_3$) δ 7.37 – 7.27 (m, 8H), 7.25 (d, *J* = 4.5 Hz, 1H), 6.14 (td, *J* = 6.7, 3.3 Hz, 1H), 5.17 (dd, *J* = 47.8, 2.9 Hz, 2H), 3.60 (q, *J* = 6.6 Hz, 1H), 3.28 (dt, *J* = 11.5, 5.8 Hz, 1H), 3.20 – 3.11 (m, 1H), 2.26 (s, 3H), 1.41 (d, *J* = 6.8 Hz, 3H).
^{13}C NMR (126 MHz, CDCl$_3$) δ 143.78, 138.59, 134.32 (d, *J* = 8.1 Hz), 133.51, 128.74, 127.76, 127.63, 127.21, 79.32 (d, *J* = 163.5 Hz), 63.77, 52.34, 39.17, 19.27.
^{19}F NMR (471 MHz, CDCl$_3$) δ -209.33.
HRMS (ESI-TOF) m/z: [M + H]$^+$ Calc. for $C_{19}H_{22}ClFN$ 318.1419; 318.1414.

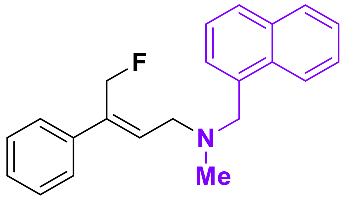

42

^{1}H NMR (500 MHz, CDCl$_3$) δ 8.23 (dd, *J* = 8.5, 1.4 Hz, 1H), 7.77 (dd, *J* = 7.9, 1.6 Hz, 1H), 7.70 (d, *J* = 8.0 Hz, 1H), 7.49 – 7.13 (m, 10H), 6.16 (td, *J* = 6.9, 3.5 Hz, 1H), 5.21 (d, *J* = 47.8 Hz, 2H), 3.89 (s, 2H), 3.29 (dd, *J* = 6.9, 4.4 Hz, 2H), 2.24 (s, 3H).
^{13}C NMR (126 MHz, CDCl$_3$) δ 140.14 (d, *J* = 1.3 Hz), 137.39 (d, *J* = 13.6 Hz), 134.72, 134.06, 133.14 (d, *J* = 8.1 Hz), 132.62, 128.61, 128.26, 127.67 (d, *J* = 1.3 Hz), 126.32, 126.02, 125.79, 125.27, 124.78, 79.61 (d, *J* = 163.6 Hz), 60.67, 55.19 (d, *J* = 2.3 Hz), 42.88.
^{19}F NMR (471 MHz, CDCl$_3$) δ -209.01.
HRMS (ESI-TOF) m/z: [M + H]$^+$ Calc. for $C_{22}H_{23}FN$ 320.1808; found 320.1800

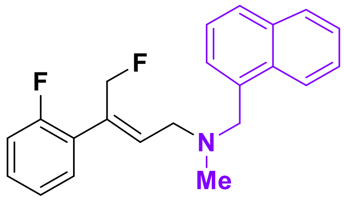

43

^{1}H NMR (500 MHz, CDCl$_3$) δ 8.31 (d, *J* = 8.3 Hz, 1H), 7.85 (dd, *J* = 7.8, 1.6 Hz, 1H), 7.79 (d, *J* = 8.0 Hz, 1H), 7.55 – 7.48 (m, 2H), 7.43 (dt, *J* = 14.9, 7.0 Hz, 1H), 7.28 (td, *J* = 7.3, 3.2 Hz, 2H), 7.11 (td, *J* = 7.6, 1.2 Hz, 1H), 7.05 (ddd, *J* = 11.1, 7.9, 1.2 Hz, 1H), 6.12 (td, *J* = 6.9, 2.9 Hz, 1H), 5.25 (d, *J* = 47.5 Hz, 2H), 3.98 (s, 2H), 3.43 – 3.27 (m, 2H), 2.33 (s, 3H).
^{13}C NMR (126 MHz, CDCl$_3$) δ 160.01 (d, *J* = 246.8 Hz), 135.11 (d, *J* = 106.1 Hz), 132.63, 130.47 (d, *J* = 3.9 Hz), 129.36 (d, *J* = 8.4 Hz), 128.59, 128.28, 127.71, 126.03, 125.81, 125.28, 124.81, 124.33 (d, *J* = 3.5 Hz), 115.89, 115.71, 80.80 – 79.23 (m), 60.60, 54.90, 42.82.
^{19}F NMR (471 MHz, CDCl$_3$) δ -115.82, -210.94.
HRMS (ESI-TOF) m/z: [M + H]$^+$ Calc. for $C_{22}H_{22}F_2N$ 338.1714; found 338.1711.

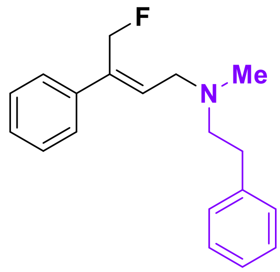

44

^{1}H NMR (500 MHz, $CDCl_3$) δ 7.42 – 7.31 (m, 2H), 7.31 – 7.06 (m, 9H), 6.08 (td, *J* = 6.9, 3.7 Hz, 1H), 5.21 (d, *J* = 47.8 Hz, 2H), 3.28 (dd, *J* = 6.9, 4.4 Hz, 2H), 2.75 (dd, *J* = 9.8, 6.1 Hz, 2H), 2.63 (dd, *J* = 9.7, 5.8 Hz, 2H), 2.30 (s, 3H).
^{13}C NMR (126 MHz, $CDCl_3$) δ 140.00 (d, *J* = 1.5 Hz), 137.49 (d, *J* = 13.6 Hz), 132.48, 128.84, 128.64, 128.57, 127.77, 126.28 (d, *J* = 6.3 Hz), 79.67 (d, *J* = 164.0 Hz), 59.36, 55.17 (d, *J* = 1.9 Hz), 42.38, 34.02.
^{19}F NMR (471 MHz, $CDCl_3$) δ -208.84.
HRMS (ESI-TOF) m/z: $[M + H]^+$ Calc. for $C_{19}H_{23}FN$ 284.1802; found 284.1804.

4. Control studies

(A)

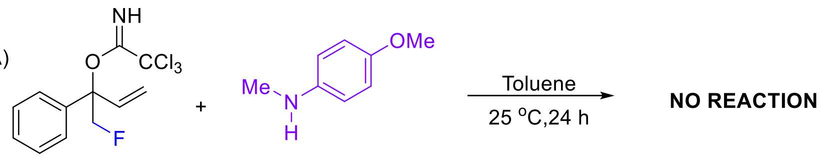

1c **2a**

(B)

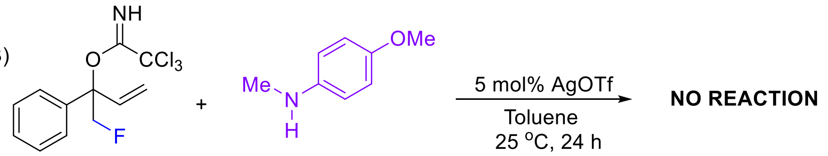

1c **2a**

(C)

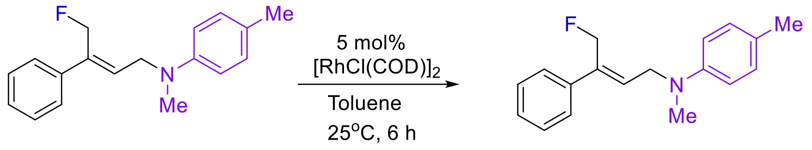

9 (*E*/*Z* = 1:6)
synthesized from $[RhCl(NBD)]_2$

9 (*E*/*Z* = 1:6)
monitored by ^{19}F and ^{1}H NMR

Proposed Mechanism

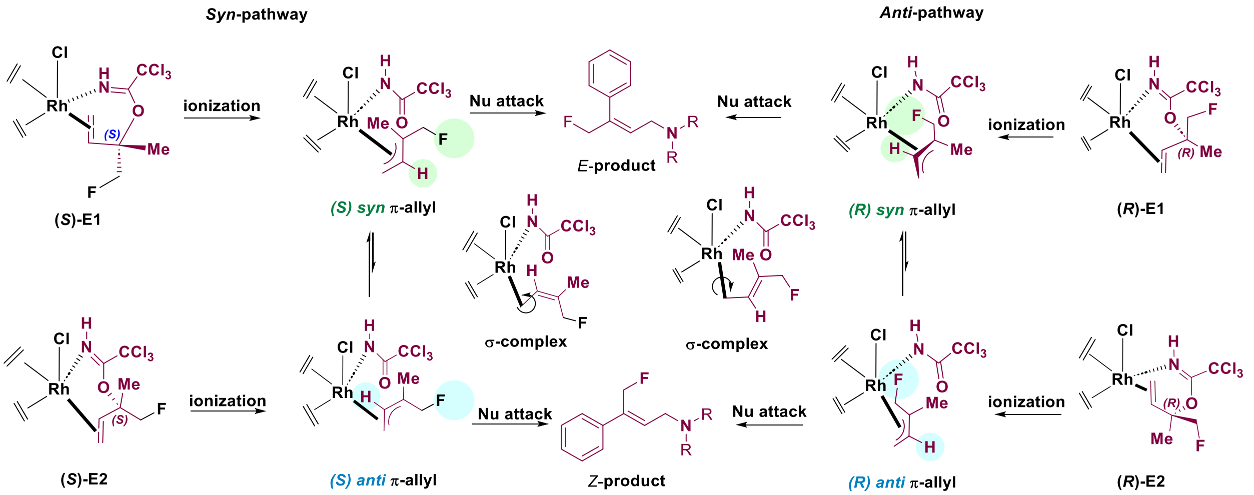

5. Computational Methods

Density functional theory (DFT) calculations were carried out with the Gaussian 16 series of programs[3] using the PBE1PBE functional[4] and the Grimme-D3(BJ)[5] empirical corrections for dispersion. 6-31G(d,p) basis set for H, C, N, O Cl and the Stuttgart–Dresden (SDD) basis set[6] with pseudo potential for rhodium were utilized for geometry optimizations and vibrational frequency calculations. Solvation effects in toluene were incorporated using the implicit SMD solvation model[7] and included during structure optimization. Harmonic vibrational frequency calculations confirmed all the optimized structures as minima, and the converged wave functions were tested for SCF stability. The zero-point energy, thermal corrections, and entropic contributions were included in the calculation of the free energies. The standard states of 1 M concentration were used for all the reactants and products. A TS keyword was used that requests optimization to a transition state rather than a local minimum, using the Berny algorithm. All the transition state structures had only one imaginary frequency. Distances in structural figures are shown in Å and energies are in kcal/mol.

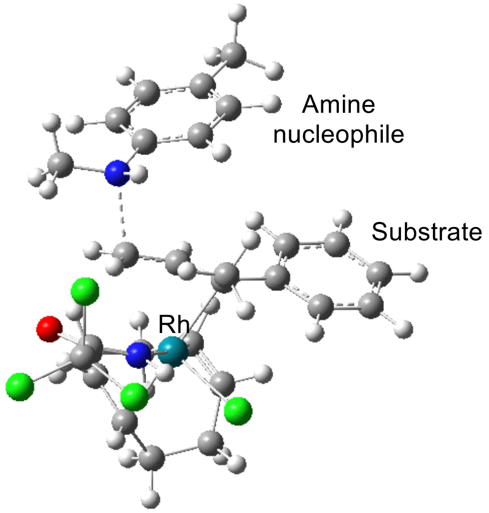

TS2-L with CH_3

(1.18 kcal/mol)

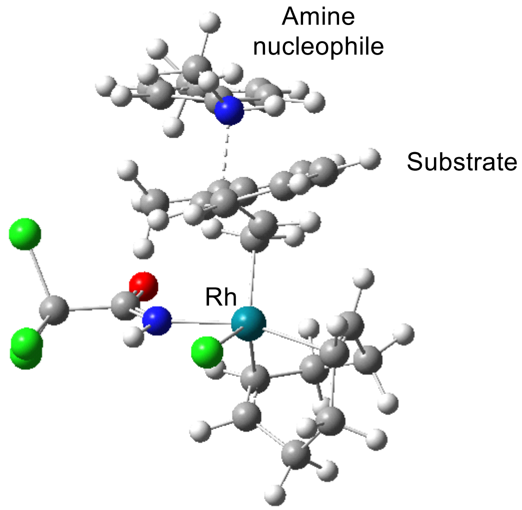

TS2-B with CH_3

(0.00 kcal/mol)

Figure S1. DFT-optimized transition-state structures **TS2-L** and **TS2-B** with CH_3 substitution in place of CH_2F. Free energy analysis supports selective formation of the branched product in the absence of the F atom.

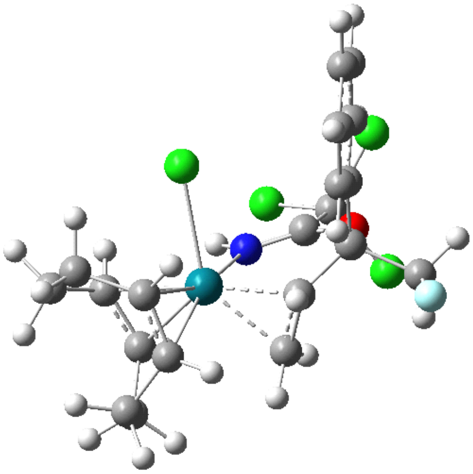

R-E1
(5.32)

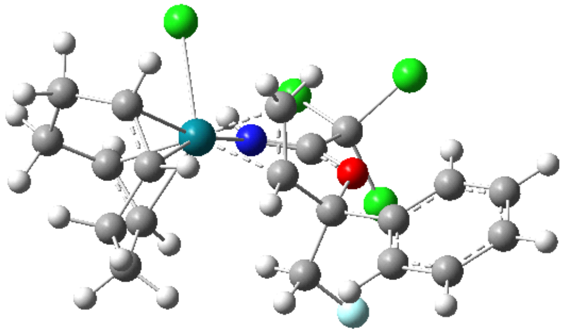

R-E2
(0.00)

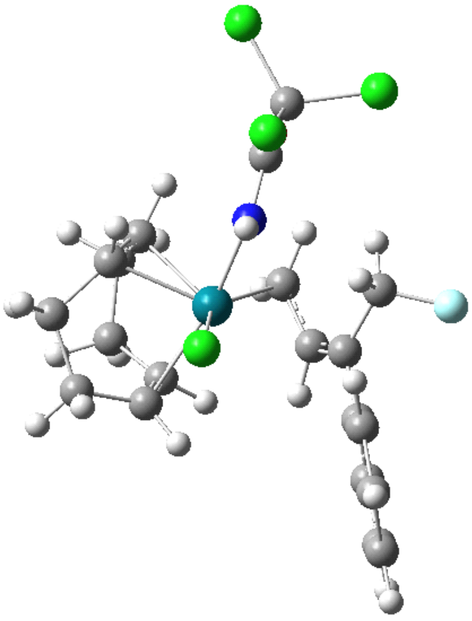

R-I1 (anti)
(-2.20)

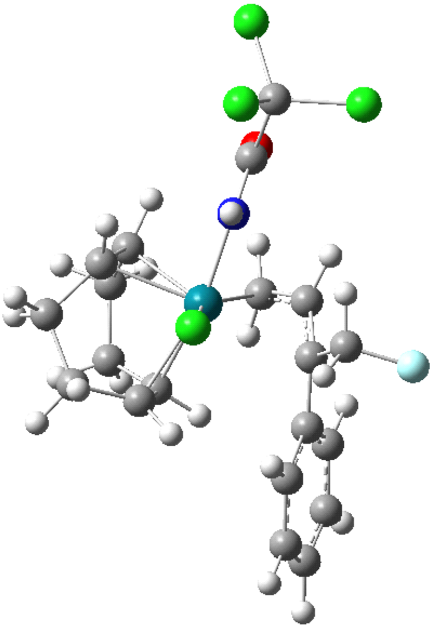

R-I2 (syn)
(-4.84)

Figure S2. DFT-optimized structures **R-E1**, **R-E2**, **R-I1(anti)** and **R-I2(syn)**. Relative free energy changes (ΔG) are in kcal mol^{-1}.

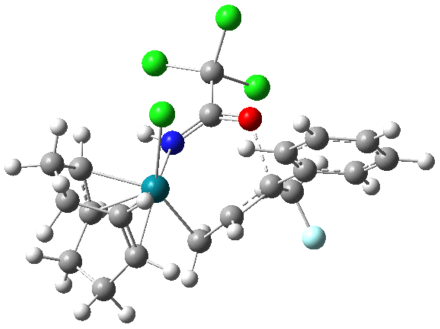

TS1
(14.67)

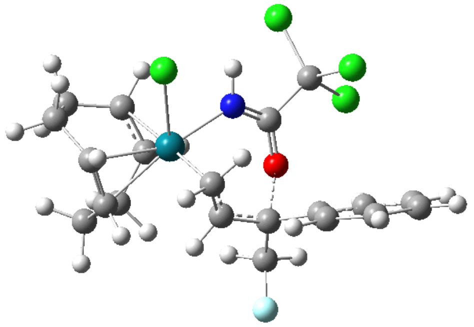

TS1'
(15.30)

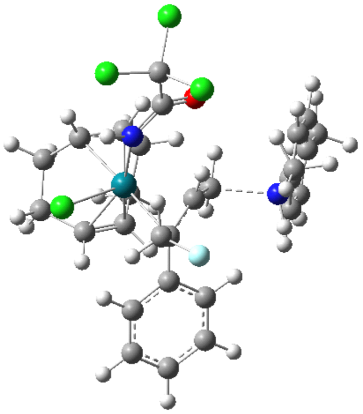

TS2-L'
(16.29)

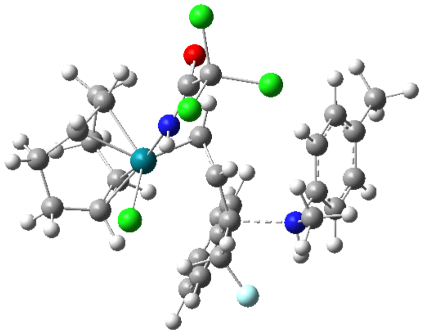

TS2-B'
(16.72)

Figure S3. DFT-optimized transition-state structures **TS1**, **TS1'**, **TS2-L'** and **TS2-B'**. Relative free energy changes (ΔG) are in kcal mol^{-1}.

TS2-B'

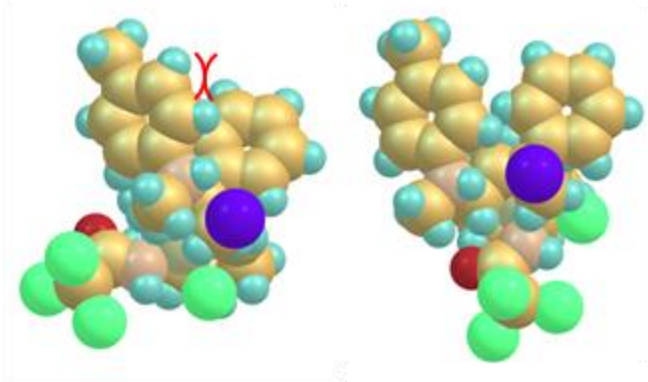

TS2-L

Figure S4. CPK space-filling model of the optimized geometries of **TS2-B'** and **TS2-L**.

Cartesian coordinates of DFT-calculated structures:

R-E1

Gibbs free energy: -1854646.41 kcal/mol

C	-1.725301000	0.775367000	-0.603214000
C	-0.360107000	1.371138000	-0.989738000
C	0.316343000	-1.557325000	-0.432402000
C	1.663076000	-0.882843000	-0.145910000
C	-1.663464000	-0.226308000	0.521808000
C	0.615048000	1.467110000	0.169723000
C	-0.733731000	-1.252710000	0.621135000
C	1.526813000	0.466750000	0.518775000
H	-2.197992000	0.297138000	-1.475111000
H	-2.385965000	1.581832000	-0.279283000
H	-0.522195000	2.376491000	-1.390417000
H	0.100295000	0.795257000	-1.800612000
H	0.458225000	-2.642058000	-0.484815000
H	-0.066469000	-1.256446000	-1.412772000
H	2.251854000	-0.792175000	-1.071727000
H	2.245690000	-1.522123000	0.526458000
H	0.820766000	2.476489000	0.522977000
H	-0.993812000	-2.077701000	1.281690000
H	2.410313000	0.770452000	1.083117000
H	-2.563038000	-0.295946000	1.131433000
Rh	-0.122833000	0.396385000	1.966047000
C	-0.633577000	-0.791983000	3.629243000
C	0.621166000	-1.226638000	3.110720000
N	0.984920000	1.539646000	3.234838000
Cl	-1.751553000	2.309223000	2.262290000
H	-1.503968000	-1.401945000	3.399820000
C	-0.776382000	-0.144821000	4.982808000
H	0.693458000	-2.168137000	2.573961000
H	1.546019000	-0.954133000	3.619808000

C	1.006559000	1.539177000	4.505927000
H	1.604254000	2.224114000	2.811024000
C	-0.498413000	-1.177889000	6.096568000
O	0.289804000	0.807479000	5.320056000
C	-2.126681000	0.519371000	5.200819000
C	2.011877000	2.377309000	5.328597000
H	0.514961000	-1.575924000	5.982494000
H	-0.628893000	-0.725202000	7.084313000
F	-1.390677000	-2.235200000	5.983438000
C	-3.298684000	-0.173209000	4.878796000
C	-2.233653000	1.785841000	5.776114000
Cl	2.918495000	3.506731000	4.298033000
Cl	3.154700000	1.227109000	6.077532000
Cl	1.168811000	3.296499000	6.588234000
C	-4.546357000	0.399313000	5.110776000
H	-3.251489000	-1.168615000	4.450262000
C	-3.481823000	2.359911000	6.004136000
H	-1.341573000	2.343284000	6.032553000
C	-4.644695000	1.671339000	5.670413000
H	-5.444361000	-0.153117000	4.847380000
H	-3.540056000	3.356043000	6.434438000
H	-5.618507000	2.121850000	5.841848000

R-E2

Gibbs free energy: -1854651.73 kcal/mol

Cl	0.075264000	-1.427087000	2.717111000
C	1.054250000	1.429389000	-0.221594000
C	1.405240000	1.182475000	1.122957000
H	1.836787000	1.430795000	-0.976461000
H	2.444630000	0.987887000	1.377751000
H	0.812794000	1.594001000	1.937105000
C	-0.046173000	2.397334000	-0.618944000
C	-0.677360000	1.983618000	-1.942173000
H	-1.269441000	1.075094000	-1.816488000

H	0.100379000	1.805608000	-2.689912000
O	-1.108978000	2.493935000	0.363342000
C	-1.754634000	1.442477000	0.814321000
N	-1.365491000	0.235366000	0.735056000
H	-1.978945000	-0.434040000	1.194358000
C	-3.068330000	1.889197000	1.482154000
Cl	-3.956777000	0.510292000	2.155939000
Cl	-2.668547000	3.026971000	2.790835000
Cl	-4.068222000	2.695336000	0.251300000
F	-1.520683000	2.978265000	-2.419857000
C	0.551961000	3.799204000	-0.686782000
C	1.458767000	4.104035000	-1.707311000
C	0.265088000	4.768283000	0.276169000
C	2.064738000	5.355622000	-1.766122000
H	1.701746000	3.366442000	-2.467644000
C	0.874913000	6.020657000	0.216471000
H	-0.437013000	4.550676000	1.072906000
C	1.775629000	6.320427000	-0.802145000
H	2.763363000	5.576052000	-2.568607000
H	0.639329000	6.764258000	0.972996000
H	2.248092000	7.297745000	-0.846781000
Rh	0.491884000	-0.572449000	0.346575000
C	-0.316404000	-1.522079000	-1.456589000
C	2.359785000	-1.113467000	-0.676137000
C	-0.367070000	-2.443262000	-0.400940000
C	2.254739000	-1.834697000	0.511502000
C	0.709176000	-1.500978000	-2.564410000
H	-1.251472000	-1.015281000	-1.696135000
C	2.138505000	-1.709612000	-2.055627000
H	2.986441000	-0.224101000	-0.643303000
C	0.586322000	-3.613922000	-0.236992000
H	-1.321128000	-2.546370000	0.114382000
C	1.826105000	-3.277230000	0.608461000
H	2.778085000	-1.449892000	1.385421000

H	0.456749000	-2.248797000	-3.332296000
H	0.643937000	-0.524923000	-3.058231000
H	2.847364000	-1.254139000	-2.755411000
H	2.387205000	-2.775309000	-2.026784000
H	0.047915000	-4.435643000	0.244874000
H	0.877370000	-3.983860000	-1.226748000
H	2.674124000	-3.921071000	0.328464000
H	1.604257000	-3.476088000	1.659056000

R-I1 (Anti)

Gibbs free energy: -1854653.93 kcal/mol

Cl	-0.176825000	-0.476653000	2.838606000
C	-1.492708000	-1.502019000	-0.741091000
C	-0.564291000	-0.753863000	-1.532097000
H	-2.526414000	-1.170353000	-0.749036000
H	-0.997450000	-0.083942000	-2.270163000
H	0.395629000	-1.166957000	-1.828935000
C	-1.183462000	-2.470555000	0.203231000
O	2.532444000	-0.707180000	-1.581413000
C	2.534935000	-0.986393000	-0.381011000
N	1.559736000	-0.849127000	0.495098000
H	1.780285000	-1.095462000	1.453542000
C	3.860254000	-1.671171000	0.144392000
Cl	5.252730000	-0.719987000	-0.414141000
Cl	3.914051000	-3.307302000	-0.570462000
Cl	3.964048000	-1.829717000	1.925860000
C	-2.211515000	-2.949117000	1.145124000
C	-1.836602000	-3.436375000	2.407474000
C	-3.577112000	-2.931345000	0.813351000
C	-2.798219000	-3.865142000	3.316345000
H	-0.791827000	-3.451221000	2.695399000
C	-4.535107000	-3.370734000	1.718985000
H	-3.894655000	-2.608654000	-0.174048000
C	-4.149358000	-3.832873000	2.978135000

H	-2.487830000	-4.223621000	4.293642000
H	-5.584453000	-3.364904000	1.437234000
H	-4.898504000	-4.175499000	3.686664000
C	0.101187000	-3.257116000	0.170289000
H	0.735762000	-2.977682000	-0.668825000
H	0.667646000	-3.188179000	1.101395000
F	-0.246198000	-4.604180000	-0.010249000
Rh	-0.277419000	0.049302000	0.425065000
C	-2.041904000	1.286270000	1.184987000
C	0.740444000	1.877170000	0.879407000
C	-2.077737000	1.391962000	-0.188595000
C	0.714305000	1.722850000	-0.521913000
C	-1.476022000	2.349462000	2.090922000
H	-2.663233000	0.530072000	1.661647000
C	-0.096775000	2.877458000	1.654779000
H	1.641455000	1.533028000	1.382509000
C	-1.621412000	2.610163000	-0.962328000
H	-2.741112000	0.718834000	-0.722857000
C	-0.176260000	2.495780000	-1.456815000
H	1.597739000	1.297455000	-0.993026000
H	-2.199434000	3.176127000	2.147461000
H	-1.396520000	1.924197000	3.092098000
H	0.462022000	3.165262000	2.549475000
H	-0.200007000	3.786799000	1.051857000
H	-2.290189000	2.746251000	-1.818134000
H	-1.747879000	3.497218000	-0.334140000
H	0.259141000	3.493130000	-1.617911000
H	-0.154254000	1.999077000	-2.431252000

R-I2 (Syn)

Gibbs free energy: -1854656.57 kcal/mol

Cl	0.220255000	-0.527471000	2.965130000
C	-0.426593000	-2.062871000	-0.627863000
C	-0.551137000	-0.935970000	-1.477672000
H	0.511788000	-2.607110000	-0.680229000

H	0.252694000	-0.790471000	-2.192088000
H	-1.520935000	-0.547665000	-1.763412000
C	-1.240089000	-2.323355000	0.486664000
C	-0.681189000	-3.288701000	1.499619000
H	-0.962432000	-3.026097000	2.520696000
H	0.407909000	-3.350950000	1.433664000
O	2.480910000	-1.035070000	-1.644600000
C	2.659086000	-1.197555000	-0.437188000
N	1.811891000	-0.968934000	0.546273000
H	2.132181000	-1.142844000	1.492052000
C	4.034402000	-1.857475000	-0.023689000
Cl	4.503784000	-1.558412000	1.677764000
Cl	5.323334000	-1.251613000	-1.077481000
Cl	3.836642000	-3.621127000	-0.261437000
C	-2.709662000	-2.139640000	0.513830000
C	-3.390630000	-1.931180000	1.722850000
C	-3.461449000	-2.257666000	-0.666011000
C	-4.775329000	-1.797976000	1.744345000
H	-2.830325000	-1.857666000	2.651027000
C	-4.847201000	-2.126823000	-0.642567000
H	-2.956590000	-2.482329000	-1.601078000
C	-5.508887000	-1.887699000	0.561299000
H	-5.283197000	-1.627158000	2.689609000
H	-5.411838000	-2.228950000	-1.565343000
H	-6.590692000	-1.789247000	0.580446000
F	-1.199243000	-4.568494000	1.233828000
Rh	-0.083015000	-0.207946000	0.539641000
C	-1.831043000	1.046288000	1.427922000
C	0.860654000	1.758998000	0.786753000
C	-2.030250000	0.995910000	0.069428000
C	0.721633000	1.504665000	-0.581729000
C	-1.237859000	2.206984000	2.178309000
H	-2.344641000	0.306592000	2.034764000
C	0.031664000	2.782671000	1.536997000

H	1.808948000	1.483627000	1.241975000
C	-1.737284000	2.145215000	-0.874325000
H	-2.717273000	0.231411000	-0.282012000
C	-0.319703000	2.136768000	-1.468592000
H	1.573914000	1.077703000	-1.106602000
H	-2.010940000	2.985074000	2.265411000
H	-1.011577000	1.868126000	3.190298000
H	0.655016000	3.219432000	2.322460000
H	-0.215706000	3.601299000	0.852067000
H	-2.465888000	2.115463000	-1.690599000
H	-1.922341000	3.083287000	-0.340275000
H	-0.002097000	3.162228000	-1.708250000
H	-0.324788000	1.595982000	-2.419108000

TS

Gibbs free energy: -1854646.20 kcal/mol

C	2.121959000	-2.655811000	-0.159595000
C	-0.276572000	-1.702726000	-1.469043000
C	1.258485000	-3.156201000	0.789660000
C	-1.111816000	-1.961123000	-0.397243000
H	1.573827000	-3.084870000	1.830538000
Rh	0.679456000	-1.002692000	0.467538000
Cl	2.090890000	-0.349664000	2.215124000
C	2.112635000	1.547149000	-0.384947000
C	1.864236000	0.140676000	-0.822564000
H	1.638145000	1.899090000	0.521193000
H	2.805827000	-0.407309000	-0.887720000
H	1.355123000	0.114876000	-1.786335000
C	2.873642000	2.418895000	-1.075521000
C	3.529741000	2.020972000	-2.363504000
H	4.404940000	2.638930000	-2.582708000
H	3.830923000	0.968579000	-2.356986000
O	-1.649443000	0.985482000	-1.064985000
C	-1.274428000	1.295417000	0.059389000

N	-0.416915000	0.636897000	0.832954000
H	-0.166591000	1.092327000	1.705230000
C	-1.830269000	2.640756000	0.682068000
Cl	-0.502291000	3.836669000	0.683196000
Cl	-2.387498000	2.380073000	2.367476000
Cl	-3.179157000	3.253954000	-0.278180000
C	3.044228000	3.812592000	-0.605917000
C	3.121809000	4.867686000	-1.527957000
C	3.113774000	4.118222000	0.762165000
C	3.243954000	6.186073000	-1.095205000
H	3.054012000	4.658844000	-2.592682000
C	3.231946000	5.436549000	1.193756000
H	3.093301000	3.312933000	1.492317000
C	3.295904000	6.477542000	0.267244000
H	3.288887000	6.988912000	-1.826582000
H	3.285576000	5.649795000	2.258280000
H	3.390353000	7.506209000	0.604518000
F	2.645729000	2.173522000	-3.456447000
H	-1.902361000	-1.233322000	-0.223576000
C	0.068962000	-4.043710000	0.503295000
C	2.004655000	-2.881013000	-1.644409000
C	-1.237685000	-3.246605000	0.373095000
C	0.578142000	-2.722047000	-2.196890000
H	0.255421000	-4.635292000	-0.396708000
H	-0.035660000	-4.756237000	1.327412000
H	-1.617014000	-3.002079000	1.373131000
H	-2.019135000	-3.863175000	-0.095572000
H	0.054790000	-3.682916000	-2.194094000
H	0.642092000	-2.418530000	-3.246129000
H	2.668526000	-2.174694000	-2.151043000
H	2.394770000	-3.879803000	-1.886226000
H	-0.498737000	-0.776118000	-1.992857000
H	3.077038000	-2.274882000	0.199554000

TS1

Gibbs free energy: -1854637.06 kcal/mol

Cl	-0.237191000	0.638717000	2.775302000
C	-0.964876000	-1.743073000	0.031888000
C	-0.746697000	-1.034608000	-1.198021000
H	-1.976642000	-1.811695000	0.422828000
H	-1.588718000	-0.892570000	-1.867217000
H	0.192295000	-1.179807000	-1.727380000
C	-0.083008000	-2.734778000	0.567940000
O	1.549078000	-1.953535000	1.441958000
C	2.067524000	-0.932805000	0.922880000
N	1.459121000	0.009877000	0.267525000
H	2.076754000	0.721710000	-0.103816000
C	3.623474000	-0.889838000	1.081767000
Cl	4.355213000	0.623685000	0.467925000
Cl	4.299744000	-2.258958000	0.153203000
Cl	4.023297000	-1.062850000	2.797347000
C	0.873404000	-3.420265000	-0.383162000
Rh	-0.534773000	0.432232000	0.294172000
C	-2.478004000	1.423654000	0.877703000
C	0.044885000	2.496145000	0.129724000
C	-2.669815000	0.789560000	-0.334273000
C	-0.248251000	2.003324000	-1.158956000
C	-2.308146000	2.921972000	1.014175000
H	-2.741676000	0.879245000	1.779618000
C	-0.845565000	3.383252000	0.961634000
H	1.092799000	2.541510000	0.426242000
C	-2.698799000	1.482783000	-1.675823000
H	-3.139089000	-0.192945000	-0.308299000
C	-1.501218000	2.410754000	-1.918306000
H	0.596083000	1.698089000	-1.776607000
H	-2.897419000	3.409454000	0.228840000

H	-2.745136000	3.233006000	1.967634000
H	-0.439382000	3.375805000	1.975728000
H	-0.782277000	4.419369000	0.595580000
H	-2.727899000	0.717634000	-2.457184000
H	-3.644433000	2.037041000	-1.767726000
H	-1.753125000	3.441595000	-1.643732000
H	-1.275666000	2.432171000	-2.989102000
C	-0.550831000	-3.494328000	1.738508000
C	-1.144153000	-2.810726000	2.811224000
C	-0.431019000	-4.890669000	1.794429000
C	-1.610678000	-3.514695000	3.914410000
H	-1.177159000	-1.723833000	2.795707000
C	-0.920485000	-5.591578000	2.892915000
H	0.011035000	-5.439980000	0.968904000
C	-1.507836000	-4.906361000	3.955569000
H	-2.045032000	-2.973541000	4.750495000
H	-0.838930000	-6.674626000	2.919163000
H	-1.877665000	-5.454914000	4.817825000
H	1.564380000	-4.085266000	0.134998000
H	1.433795000	-2.697407000	-0.977823000
F	0.113525000	-4.191880000	-1.274623000

TS1'

Gibbs free energy: -1854636.43 kcal/mol

Cl	-0.845838000	-1.586579000	3.084283000
C	1.461516000	0.967270000	0.713865000
C	1.282450000	0.536781000	2.063420000
H	2.303293000	0.571832000	0.148982000
H	2.115456000	0.056894000	2.574975000
H	0.594166000	1.064502000	2.717094000
C	0.916312000	2.131592000	0.078092000
O	-0.961193000	1.789223000	-0.733387000
C	-1.746056000	1.262872000	0.099019000
N	-1.451990000	0.296447000	0.909729000

H	-2.155473000	0.015490000	1.586904000
C	-3.175914000	1.890213000	0.206830000
Cl	-4.415670000	0.612707000	0.340791000
Cl	-3.531829000	2.888055000	-1.213676000
Cl	-3.214219000	2.908565000	1.673836000
C	1.365789000	2.322898000	-1.354747000
H	0.779544000	3.078343000	-1.877125000
H	1.340067000	1.377601000	-1.899985000
F	2.695218000	2.763829000	-1.320168000
C	0.572392000	3.355491000	0.827904000
C	-0.252976000	4.347967000	0.263774000
C	1.169737000	3.617458000	2.073695000
C	-0.503248000	5.532998000	0.939116000
H	-0.739745000	4.161686000	-0.687023000
C	0.922285000	4.811492000	2.744670000
H	1.868377000	2.906159000	2.497594000
C	0.078902000	5.768187000	2.186302000
H	-1.163705000	6.272979000	0.496160000
H	1.400385000	4.994739000	3.702692000
H	-0.118309000	6.697366000	2.714032000
Rh	0.201926000	-0.869045000	0.941953000
C	1.467182000	-2.646642000	1.416442000
C	-0.882096000	-2.421035000	-0.208458000
C	-0.290669000	-1.516450000	-1.096291000
C	2.099030000	-1.952380000	0.400445000
C	0.625563000	-3.884556000	1.239068000
H	1.792609000	-2.453738000	2.436986000
C	-0.352958000	-3.817414000	0.055964000
H	-1.925681000	-2.248998000	0.048509000
C	0.975733000	-1.764499000	-1.882355000
H	-0.923930000	-0.727502000	-1.498998000
C	2.107692000	-2.385952000	-1.053811000
H	2.901916000	-1.283004000	0.703740000
H	1.293820000	-4.753621000	1.139585000

H	0.059161000	-4.022633000	2.162799000
H	-1.199338000	-4.478655000	0.266146000
H	0.112866000	-4.204428000	-0.857264000
H	0.756618000	-2.391887000	-2.760025000
H	1.313127000	-0.801216000	-2.278933000
H	3.071289000	-2.110337000	-1.495148000
H	2.060348000	-3.479039000	-1.093759000

TS2-L

Gibbs free energy: -2084179.76 kcal/mol

Cl	-0.507347000	-2.160114000	1.958218000
C	0.443835000	1.506739000	0.067685000
C	-0.644592000	2.354375000	0.293706000
H	0.870085000	1.556101000	-0.926976000
H	-1.143695000	2.426953000	1.245360000
H	-1.157620000	2.792979000	-0.554400000
C	1.056558000	0.665222000	1.049786000
C	0.911891000	1.015574000	2.502953000
H	1.130090000	0.169235000	3.155773000
H	-0.068037000	1.419134000	2.752007000
O	-3.424791000	1.997718000	1.254318000
C	-2.980271000	1.184553000	2.069880000
N	-1.966971000	0.353143000	1.936875000
H	-1.820334000	-0.283220000	2.713399000
C	-3.632408000	1.257225000	3.508972000
Cl	-5.382813000	1.495227000	3.357038000
Cl	-2.899542000	2.684465000	4.316877000
Cl	-3.341176000	-0.180155000	4.529952000
N	0.217055000	4.370269000	0.734090000
C	1.187483000	4.629023000	-0.246162000
C	0.889000000	5.329831000	-1.419851000
C	2.470019000	4.085981000	-0.087528000
C	1.862590000	5.481710000	-2.405544000
C	3.428329000	4.250403000	-1.080643000

C	3.147590000	4.950427000	-2.260627000
H	-0.095836000	5.760652000	-1.572069000
H	2.708839000	3.530932000	0.817189000
H	1.611943000	6.032829000	-3.309487000
H	4.418091000	3.823372000	-0.932211000
F	1.853337000	2.033848000	2.815808000
C	2.340994000	-0.017004000	0.705551000
C	2.753582000	-1.152694000	1.420821000
C	3.181994000	0.458550000	-0.312174000
C	3.945927000	-1.799907000	1.112077000
H	2.110287000	-1.556796000	2.195533000
C	4.377116000	-0.188649000	-0.620442000
H	2.923534000	1.363266000	-0.854615000
C	4.764304000	-1.324535000	0.087128000
H	4.232702000	-2.685380000	1.673305000
H	5.011145000	0.206503000	-1.410203000
H	5.696134000	-1.830098000	-0.151469000
C	4.201584000	5.155189000	-3.311860000
H	3.757937000	5.309520000	-4.300379000
H	4.815757000	6.036370000	-3.087413000
H	4.877969000	4.296553000	-3.374049000
H	0.642178000	4.137396000	1.625500000
C	-0.929825000	5.251882000	0.873294000
H	-1.593391000	5.154419000	0.009090000
H	-1.496353000	4.952397000	1.757157000
H	-0.633309000	6.303812000	0.974322000
Rh	-0.820483000	-0.301920000	0.343555000
C	-2.638066000	-1.291738000	-0.428836000
C	0.102314000	-1.915504000	-0.972066000
C	0.100230000	-0.756283000	-1.718847000
C	-2.566167000	-0.008995000	-0.978074000
C	-2.355728000	-2.570488000	-1.195816000
H	-3.243407000	-1.401448000	0.468434000
C	-0.894378000	-3.035398000	-1.111544000

H	1.016508000	-2.160763000	-0.435574000
C	-0.877452000	-0.458526000	-2.836619000
H	1.037204000	-0.209088000	-1.739167000
C	-2.105118000	0.332220000	-2.372862000
H	-3.135599000	0.775054000	-0.482260000
H	-2.997028000	-3.361219000	-0.795022000
H	-2.661270000	-2.433611000	-2.239242000
H	-0.624289000	-3.622682000	-2.002455000
H	-0.772986000	-3.693174000	-0.249186000
H	-0.355163000	0.107914000	-3.614808000
H	-1.180043000	-1.402841000	-3.300642000
H	-2.938208000	0.196979000	-3.079472000
H	-1.868743000	1.402053000	-2.388194000

TS2-B

Gibbs free energy: -2084175.77 kcal/mol

C	2.173451000	-0.008710000	0.125949000
C	1.698034000	0.016322000	-1.217273000
H	2.819719000	-0.841469000	0.392392000
H	1.300859000	0.920426000	-1.667358000
H	2.162962000	-0.653681000	-1.936745000
C	2.291913000	1.093656000	1.067111000
C	1.248738000	2.170521000	0.991685000
H	0.334825000	1.800073000	1.463634000
H	1.038166000	2.453678000	-0.038783000
N	3.867455000	2.057887000	0.430369000
H	4.470861000	1.238392000	0.408482000
C	2.760660000	0.702157000	2.432804000
C	2.110402000	1.159750000	3.584762000
C	3.862860000	-0.154308000	2.585692000
C	2.543214000	0.763292000	4.847856000
H	1.247791000	1.809842000	3.506971000
C	4.303078000	-0.539910000	3.846628000
H	4.395538000	-0.527621000	1.712936000

C	3.640065000	-0.082793000	4.985737000
H	2.011264000	1.115827000	5.726837000
H	5.160074000	-1.201258000	3.939500000
H	3.975639000	-0.388339000	5.972929000
F	1.650544000	3.326104000	1.670468000
C	4.457954000	3.024830000	1.375456000
H	5.490832000	3.235013000	1.080103000
H	3.882077000	3.945976000	1.379043000
H	4.441405000	2.595668000	2.378129000
C	3.711126000	2.520325000	-0.916367000
C	4.187975000	1.733171000	-1.963503000
C	3.023838000	3.703767000	-1.197755000
C	3.967449000	2.118408000	-3.282720000
H	4.727204000	0.813212000	-1.748228000
C	2.805811000	4.071747000	-2.518953000
H	2.637862000	4.324099000	-0.395136000
C	3.261733000	3.285461000	-3.585170000
H	4.340216000	1.491492000	-4.088872000
H	2.250898000	4.983114000	-2.727386000
C	2.966564000	3.676246000	-5.003369000
H	3.145798000	4.743518000	-5.169930000
H	3.578763000	3.111311000	-5.712055000
H	1.913671000	3.481489000	-5.240445000
Rh	0.160346000	-0.946946000	-0.124407000
Cl	-0.662905000	-0.651838000	2.273315000
N	-0.959436000	0.764656000	-0.362131000
C	-0.898779000	-1.675877000	-1.845069000
C	-1.600178000	-2.034598000	-0.672440000
C	1.336515000	-2.804450000	-0.581901000
C	0.747647000	-2.925050000	0.667448000
C	-1.181444000	1.566750000	-1.380064000
H	-1.521082000	0.926188000	0.466198000
C	-0.050001000	-2.600264000	-2.680729000
H	-1.249848000	-0.797836000	-2.383084000

C	-1.640890000	-3.433988000	-0.080766000
H	-2.428592000	-1.386259000	-0.392531000
C	0.846756000	-3.505251000	-1.833608000
H	2.375371000	-2.482329000	-0.611061000
C	-0.520795000	-3.695751000	0.942385000
H	1.346541000	-2.679709000	1.543446000
O	-0.665333000	1.556595000	-2.501579000
C	-2.180331000	2.769579000	-1.125595000
H	-0.687715000	-3.196391000	-3.351771000
H	0.573389000	-1.974884000	-3.329449000
H	-2.605153000	-3.570819000	0.418387000
H	-1.613090000	-4.172711000	-0.890128000
H	1.715162000	-3.821822000	-2.421839000
H	0.321695000	-4.423026000	-1.552194000
H	-0.285846000	-4.770129000	0.993803000
H	-0.869510000	-3.395144000	1.932872000
Cl	-1.180187000	4.252304000	-0.983758000
Cl	-3.172479000	2.624353000	0.358235000
Cl	-3.274977000	2.924550000	-2.516843000

TS2-L'

Gibbs free energy: -2084177.59 kcal/mol

Cl	-1.588811000	-1.754181000	1.789716000
C	1.016048000	1.071066000	0.409626000
C	1.708096000	1.009737000	-0.827160000
H	0.308235000	1.889990000	0.467909000
H	1.177519000	1.427998000	-1.675563000
H	2.466558000	0.265946000	-1.032567000
C	1.100544000	0.252880000	1.563117000
C	0.260710000	0.723688000	2.720487000
H	0.022249000	-0.074931000	3.422511000
H	-0.660785000	1.203128000	2.384969000
O	-1.072279000	2.352843000	-1.364450000
C	-1.858820000	1.825044000	-0.568056000

N	-1.708337000	0.692460000	0.082246000
H	-2.461456000	0.400919000	0.694880000
C	-3.144538000	2.675010000	-0.236060000
Cl	-4.433094000	1.770878000	0.612997000
Cl	-3.818569000	3.326700000	-1.745618000
Cl	-2.608533000	4.022194000	0.815608000
N	3.006395000	2.563594000	-0.975975000
C	3.530910000	2.460213000	-2.289037000
C	4.644944000	1.641254000	-2.506364000
C	2.892679000	3.051863000	-3.379701000
C	5.117677000	1.432822000	-3.795327000
C	3.380145000	2.831212000	-4.667353000
C	4.495120000	2.023621000	-4.903314000
H	5.142130000	1.167864000	-1.661481000
H	2.018012000	3.678729000	-3.238478000
H	5.988540000	0.798491000	-3.944936000
H	2.873199000	3.301029000	-5.506775000
F	0.997850000	1.698682000	3.439294000
C	2.285714000	-0.552263000	1.961328000
C	2.129800000	-1.672909000	2.792460000
C	3.586951000	-0.188078000	1.588602000
C	3.229906000	-2.420946000	3.199255000
H	1.129505000	-1.967300000	3.101345000
C	4.690772000	-0.935728000	1.993191000
H	3.738892000	0.709976000	1.000161000
C	4.516374000	-2.061537000	2.795432000
H	3.081000000	-3.289993000	3.834581000
H	5.689682000	-0.627804000	1.694502000
H	5.375126000	-2.645253000	3.115253000
C	5.029085000	1.807156000	-6.290390000
H	5.227996000	0.746935000	-6.480595000
H	4.325444000	2.160680000	-7.049341000
H	5.973567000	2.345142000	-6.436792000
C	2.243189000	3.756891000	-0.610913000

H	1.270196000	3.748704000	-1.109422000
H	2.067888000	3.734737000	0.466312000
H	2.785943000	4.671409000	-0.872523000
H	3.730050000	2.376108000	-0.288388000
Rh	-0.214995000	-0.705928000	-0.028581000
C	-0.600972000	-0.747719000	-2.185687000
C	-1.435566000	-1.678611000	-1.548154000
C	1.503455000	-2.014240000	-0.764309000
C	0.656709000	-2.781933000	0.011542000
C	0.614198000	-1.125249000	-2.999146000
H	-1.011604000	0.234722000	-2.408680000
C	-1.228968000	-3.178014000	-1.652439000
H	-2.441695000	-1.350543000	-1.296401000
C	1.567974000	-2.090260000	-2.279209000
H	2.387102000	-1.636130000	-0.257506000
C	-0.378481000	-3.744527000	-0.509178000
H	0.925811000	-2.915794000	1.055026000
H	0.286949000	-1.557895000	-3.956968000
H	1.151267000	-0.208213000	-3.258330000
H	-2.206683000	-3.669472000	-1.644162000
H	-0.779707000	-3.411215000	-2.625129000
H	2.596562000	-1.882611000	-2.594176000
H	1.366270000	-3.124930000	-2.578627000
H	0.130637000	-4.669286000	-0.821685000
H	-1.028954000	-4.005204000	0.328362000

TS2-B'

Gibbs free energy: -2084177.16 kcal/mol

Cl	-2.277744000	1.291141000	2.215171000
C	0.933883000	1.529675000	0.540657000
C	0.932459000	0.771040000	-0.665195000
H	0.504844000	2.526974000	0.470100000
H	0.721187000	1.288104000	-1.598006000
H	1.563420000	-0.103326000	-0.774352000

C	1.694080000	1.339446000	1.766337000
C	0.914485000	1.836660000	2.962544000
H	0.207537000	1.073705000	3.296229000
H	0.331310000	2.725572000	2.721038000
O	-0.823642000	2.263716000	-2.757032000
C	-1.426414000	2.693383000	-1.768280000
N	-1.614786000	2.119166000	-0.601419000
H	-2.163526000	2.635890000	0.076604000
C	-1.961312000	4.177685000	-1.912459000
Cl	-3.193247000	4.651353000	-0.704502000
Cl	-2.642884000	4.415527000	-3.532791000
Cl	-0.531007000	5.250549000	-1.705750000
N	3.046091000	2.712239000	1.756438000
H	3.481361000	2.528349000	2.659633000
C	4.013277000	2.536698000	0.716635000
C	5.272019000	2.025363000	1.029270000
C	3.683196000	2.825080000	-0.608491000
C	6.196634000	1.797171000	0.015566000
C	4.615539000	2.581816000	-1.611608000
C	5.884773000	2.065350000	-1.321911000
H	5.521068000	1.788978000	2.061263000
H	2.704784000	3.217165000	-0.864080000
H	7.174518000	1.395899000	0.269721000
H	4.346495000	2.797097000	-2.642855000
C	2.503832000	0.101211000	1.986338000
C	2.415332000	-0.605819000	3.196949000
C	3.350802000	-0.407452000	0.986306000
C	3.118247000	-1.792174000	3.385999000
H	1.784714000	-0.245982000	4.002066000
C	4.061915000	-1.587705000	1.181748000
H	3.459302000	0.118039000	0.046028000
C	3.943139000	-2.289825000	2.379053000
H	3.016930000	-2.328205000	4.325405000
H	4.707689000	-1.957235000	0.390038000

H	4.489948000	-3.216919000	2.527837000
F	1.766574000	2.150476000	4.037001000
C	6.890405000	1.841024000	-2.414006000
H	6.409943000	1.492282000	-3.333415000
H	7.415723000	2.773435000	-2.655039000
H	7.644664000	1.105121000	-2.120455000
C	2.447012000	4.060530000	1.781935000
H	1.722938000	4.164516000	0.972279000
H	1.953551000	4.221160000	2.739297000
H	3.232273000	4.809952000	1.651860000
Rh	-0.943574000	0.366438000	0.237057000
C	-0.934130000	-1.426568000	1.565442000
C	-2.681446000	-0.647606000	-0.532890000
C	-0.023591000	-1.669284000	0.554077000
C	-1.665107000	-0.653600000	-1.509365000
C	-2.340895000	-1.966869000	1.619653000
H	-0.544092000	-1.048265000	2.509103000
C	-3.093240000	-1.859681000	0.283794000
H	-3.427914000	0.140087000	-0.617841000
C	-0.273258000	-2.584113000	-0.628710000
H	1.017271000	-1.473193000	0.787903000
C	-0.791185000	-1.831913000	-1.859221000
H	-1.716187000	0.107129000	-2.286215000
H	-2.309373000	-3.011404000	1.966438000
H	-2.878324000	-1.392382000	2.377564000
H	-4.165997000	-1.793454000	0.490915000
H	-2.956593000	-2.766786000	-0.315902000
H	0.664118000	-3.093562000	-0.877925000
H	-0.974168000	-3.370353000	-0.330223000
H	-1.330039000	-2.514579000	-2.534346000
H	0.059667000	-1.446454000	-2.431792000

TS2-L with CH_3

Gibbs free energy: -2021930.47 kcal/mol

Cl	-0.318468000	-2.138043000	1.915546000
C	0.375412000	1.567153000	0.026038000
C	-0.727785000	2.413012000	0.245484000
H	0.779029000	1.609568000	-0.977827000
H	-1.257505000	2.446423000	1.183509000
H	-1.276805000	2.770785000	-0.619141000
C	1.046375000	0.776962000	1.001692000
C	0.940205000	1.092249000	2.475686000
H	0.856680000	0.182054000	3.076399000
H	0.073636000	1.711883000	2.709852000
O	-3.466737000	1.902458000	1.373046000
C	-2.903000000	1.136187000	2.163207000
N	-1.885346000	0.332658000	1.950119000
H	-1.618501000	-0.254109000	2.733088000
C	-3.392500000	1.259725000	3.661589000
Cl	-5.161296000	1.382830000	3.703921000
Cl	-2.671760000	2.784152000	4.292813000
Cl	-2.889550000	-0.080483000	4.730002000
N	0.001285000	4.371297000	0.596905000
C	1.099761000	4.594018000	-0.259622000
C	0.951874000	5.187715000	-1.516291000
C	2.355040000	4.104461000	0.118747000
C	2.050599000	5.290976000	-2.367132000
C	3.441024000	4.217818000	-0.741010000
C	3.312931000	4.812715000	-2.001583000
H	-0.010893000	5.571041000	-1.839439000
H	2.477931000	3.626569000	1.088270000
H	1.918404000	5.758360000	-3.340436000
H	4.405824000	3.827391000	-0.425431000
C	2.318950000	0.102282000	0.608292000
C	2.827523000	-0.948553000	1.390249000
C	3.070068000	0.504552000	-0.508768000
C	4.013989000	-1.590602000	1.048841000

H	2.261993000	-1.294527000	2.247957000
C	4.260453000	-0.135301000	-0.848411000
H	2.749403000	1.351607000	-1.107825000
C	4.737181000	-1.192066000	-0.075402000
H	4.370767000	-2.412642000	1.663966000
H	4.820278000	0.203775000	-1.716598000
H	5.664204000	-1.693576000	-0.340082000
C	4.498232000	4.960936000	-2.912544000
H	5.050251000	5.883092000	-2.691660000
H	5.198738000	4.127833000	-2.796448000
H	4.194151000	5.005740000	-3.962776000
H	0.311996000	4.250530000	1.555662000
C	-1.160044000	5.248318000	0.521948000
H	-1.686126000	5.099657000	-0.424721000
H	-1.847649000	4.984006000	1.327681000
H	-0.880346000	6.304668000	0.612198000
Rh	-0.825755000	-0.295999000	0.291721000
C	-2.619291000	-1.360585000	-0.361549000
C	0.101715000	-1.845731000	-1.059667000
C	-0.005611000	-0.688127000	-1.804430000
C	-2.641707000	-0.073233000	-0.915720000
C	-2.326584000	-2.626098000	-1.147754000
H	-3.172562000	-1.498499000	0.565113000
C	-0.840916000	-3.017780000	-1.150470000
H	1.056637000	-2.044120000	-0.576950000
C	-1.054974000	-0.450715000	-2.871779000
H	0.897453000	-0.091532000	-1.881069000
C	-2.297734000	0.273331000	-2.343232000
H	-3.225676000	0.680897000	-0.391188000
H	-2.903576000	-3.446787000	-0.710591000
H	-2.700391000	-2.506219000	-2.171275000
H	-0.592882000	-3.591012000	-2.057079000
H	-0.638163000	-3.667331000	-0.296996000
H	-0.605530000	0.138141000	-3.678681000

H	-1.329569000	-1.412612000	-3.316994000
H	-3.163644000	0.076331000	-2.994211000
H	-2.128415000	1.355354000	-2.387681000
H	1.848127000	1.618666000	2.800401000

TS2-B with CH_3

Gibbs free energy: -2021931.66 kcal/mol

C	2.142926000	0.047928000	0.095541000
C	1.645616000	0.115551000	-1.234116000
H	2.823019000	-0.773769000	0.308804000
H	1.221397000	1.029644000	-1.636810000
H	2.100986000	-0.518806000	-1.990983000
C	2.266869000	1.135938000	1.066592000
C	1.239700000	2.224831000	1.065647000
H	0.332286000	1.819560000	1.520705000
H	0.989492000	2.534865000	0.052834000
N	3.808051000	2.088645000	0.474859000
H	4.486591000	1.330874000	0.525737000
C	2.751730000	0.688967000	2.419122000
C	2.134508000	1.141841000	3.590341000
C	3.839293000	-0.190413000	2.543177000
C	2.587301000	0.730310000	4.842039000
H	1.271424000	1.794520000	3.535115000
C	4.300344000	-0.593556000	3.792042000
H	4.343767000	-0.574947000	1.658719000
C	3.673247000	-0.133902000	4.949907000
H	2.077386000	1.081025000	5.734864000
H	5.145663000	-1.272918000	3.860065000
H	4.025153000	-0.454024000	5.926713000
C	4.233551000	3.138005000	1.416609000
H	5.215108000	3.521775000	1.123423000
H	3.518505000	3.959602000	1.416945000
H	4.287597000	2.712013000	2.419418000
C	3.708000000	2.496550000	-0.897278000

C	4.264701000	1.684970000	-1.883590000
C	3.003239000	3.644758000	-1.262631000
C	4.104901000	2.010640000	-3.227259000
H	4.816570000	0.790613000	-1.602659000
C	2.843283000	3.952643000	-2.607568000
H	2.559299000	4.288424000	-0.510904000
C	3.379960000	3.140487000	-3.614602000
H	4.540505000	1.365022000	-3.985732000
H	2.273038000	4.836664000	-2.881721000
C	3.151807000	3.467714000	-5.061127000
H	3.326341000	4.529710000	-5.263018000
H	3.805945000	2.883252000	-5.714272000
H	2.114728000	3.249063000	-5.342545000
Rh	0.153033000	-0.924147000	-0.138501000
Cl	-0.623153000	-0.691371000	2.296513000
N	-1.000438000	0.770741000	-0.310613000
C	-0.957223000	-1.644202000	-1.833570000
C	-1.605760000	-2.041310000	-0.644186000
C	1.350287000	-2.737754000	-0.676514000
C	0.809928000	-2.896833000	0.591460000
C	-1.254210000	1.591901000	-1.302593000
H	-1.500527000	0.939381000	0.554466000
C	-0.118415000	-2.531926000	-2.718008000
H	-1.344683000	-0.762484000	-2.339573000
C	-1.590748000	-3.452590000	-0.080052000
H	-2.436338000	-1.417398000	-0.319126000
C	0.832095000	-3.428831000	-1.922531000
H	2.380505000	-2.392764000	-0.736852000
C	-0.427969000	-3.705337000	0.896698000
H	1.436080000	-2.657990000	1.450046000
O	-0.806808000	1.583550000	-2.454284000
C	-2.222712000	2.806192000	-0.993172000
H	-0.765364000	-3.132123000	-3.376840000
H	0.464850000	-1.878779000	-3.376703000

H	-2.532625000	-3.624019000	0.450492000
H	-1.574043000	-4.175096000	-0.904334000
H	1.685464000	-3.712263000	-2.548732000
H	0.341248000	-4.365053000	-1.639435000
H	-0.165689000	-4.774588000	0.919518000
H	-0.747824000	-3.430722000	1.904436000
Cl	-1.244339000	4.306156000	-1.106333000
Cl	-2.995823000	2.773872000	0.623195000
Cl	-3.505848000	2.844871000	-2.225638000
H	1.552837000	3.093598000	1.649254000

----

6. References

1. M. K. Arachchi, R. N. Schaugaard, H. B. Schlegel and H. M. Nguyen, *J. Am. Chem. Soc.*, 2023, **145**, 19642–19654.
2. F. O. Chukwu, M. K. Arachchi and H. M. Nguyen, *Org. Lett.*, 2025, **27**, 594–599.
3. M. J. Frisch, G. W. Trucks, H. B. Schlegel, G. E. Scuseria, M. A. Robb, J. R. Cheeseman, G. Scalmani, V. Barone, G. A. Petersson, H. Nakatsuji, X. Li, M. Caricato, A. V. Marenich, J. Bloino, B. G. Janesko, R. Gomperts, B. Mennucci, H. P. Hratchian, J. V. Ortiz, A. F. Izmaylov, J. L. Sonnenberg, D. Williams-Young, F. Ding, F. Lipparini, F. Egidi, J. Goings, B. Peng, A. Petrone, T. Henderson, D. Ranasinghe, V. G. Zakrzewski, J. Gao, N. Rega, G. Zheng, W. Liang, M. Hada, M. Ehara, K. Toyota, R. Fukuda, J. Hasegawa, M. Ishida, T. Nakajima, Y. Honda, O. Kitao, H. Nakai, T. Vreven, K. Throssell, J. A. Montgomery, Jr., J. E. Peralta, F. Ogliaro, M. J. Bearpark, J. J. Heyd, E. N. Brothers, K. N. Kudin, V. N. Staroverov, T. A. Keith, R. Kobayashi, J. Normand, K. Raghavachari, A. P. Rendell, J. C. Burant, S. S. Iyengar, J. Tomasi, M. Cossi, J. M. Millam, M. Klene, C. Adamo, R. Cammi, J. W. Ochterski, R. L. Martin, K. Morokuma, O. Farkas, J. B. Foresman, and D. J. Fox, Gaussian 16, Revision B.01, Gaussian, Inc., Wallingford CT, 2016.
4. a) Perdew, J. P.; Burke, K.; Ernzerhof, M. Generalized Gradient Approximation Made Simple. *Phys. Rev. Lett.* **1996**, *77*, 3865–3868. https://doi.org/10.1103/PhysRevLett.77.3865. b) Adamo, C.; Barone, V. Toward Reliable Density Functional Methods without Adjustable Parameters: The PBE0 Model. *J. Chem. Phys.* **1999**, *110*, 6158–6170. https://doi.org/10.1063/1.478522.
5. Grimme, S.; Ehrlich, S.; Goerigk, L. Effect of the Damping Function in Dispersion Corrected Density Functional Theory. *J. Comput. Chem.* **2011**, *32*, 1456–1465. https://doi.org/10.1002/jcc.21759.
6. a) Hehre, W. J.; Ditchfield, R.; Pople, J. A. Self-Consistent Molecular Orbital Methods. XII. Further Extensions of Gaussian-Type Basis Sets for Use in Molecular Orbital Studies of Organic Molecules. *J. Chem. Phys.* **1972**, *56*, 2257–2261. https://doi.org/10.1063/1.1677527. b) Hariharan, P. C.; Pople, J. A. The Influence of Polarization Functions on Molecular Orbital Hydrogenation Energies. *Theor. Chim. Acta* **1973**, *28*, 213–222. https://doi.org/10.1007/BF00533485.
7. Andrae, D.; Häussermann, U.; Dolg, M.; Stoll, H.; Preuss, H. Energy-Adjusted Ab Initio Pseudopotentials for the Second and Third Row Transition Elements. *Theor. Chim. Acta* **1990**, *77*, 123–141. https://doi.org/10.1007/BF01114537.

7. NMR Spectra of All Compounds

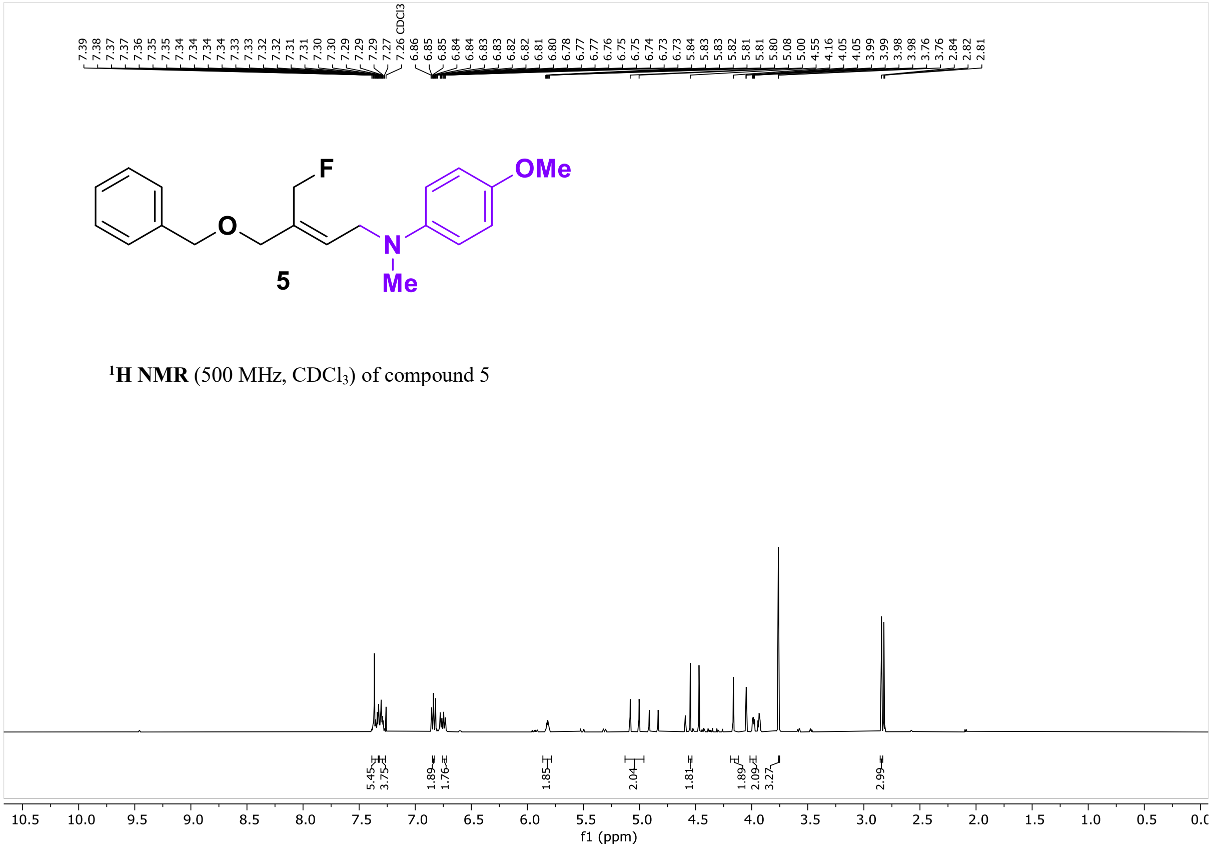

^{1}H NMR (500 MHz, $CDCl_3$) of compound 5

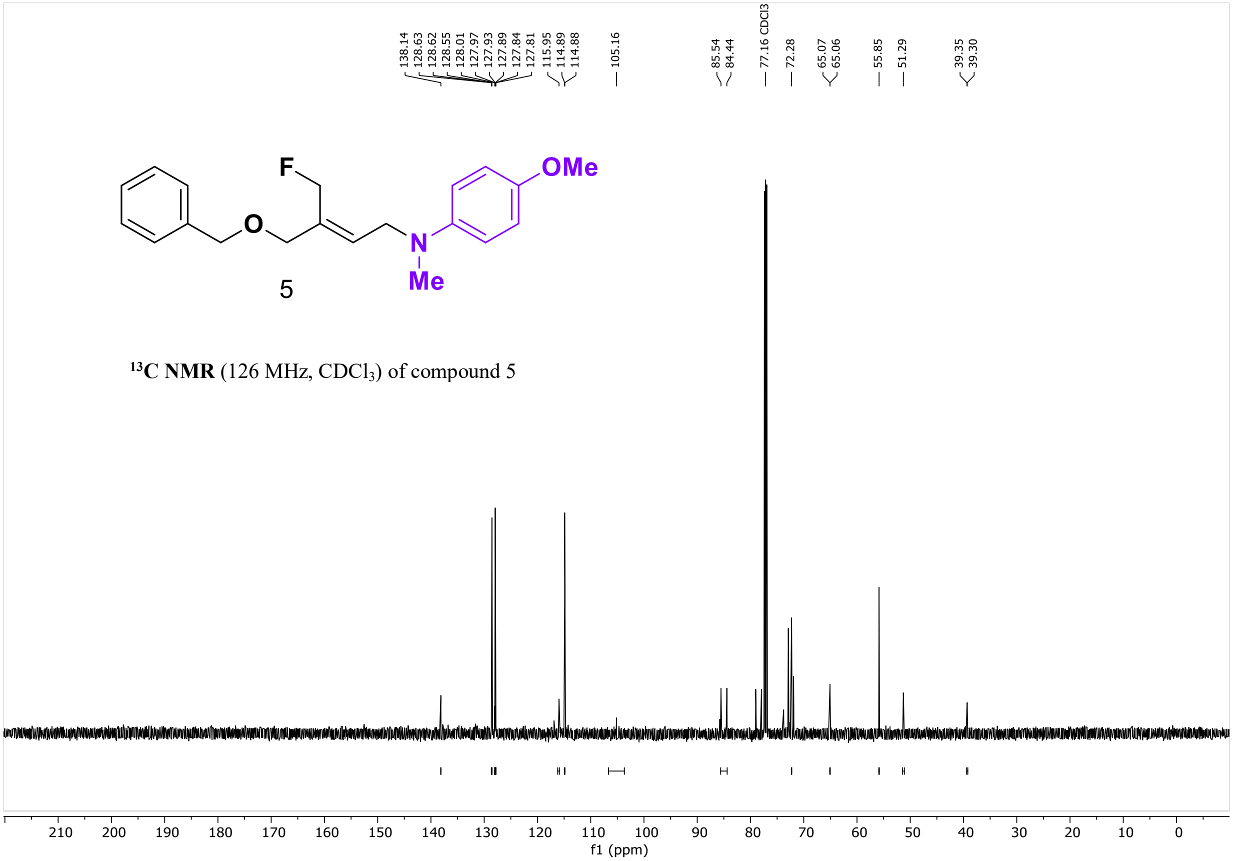

^{13}C NMR (126 MHz, $CDCl_3$) of compound 5

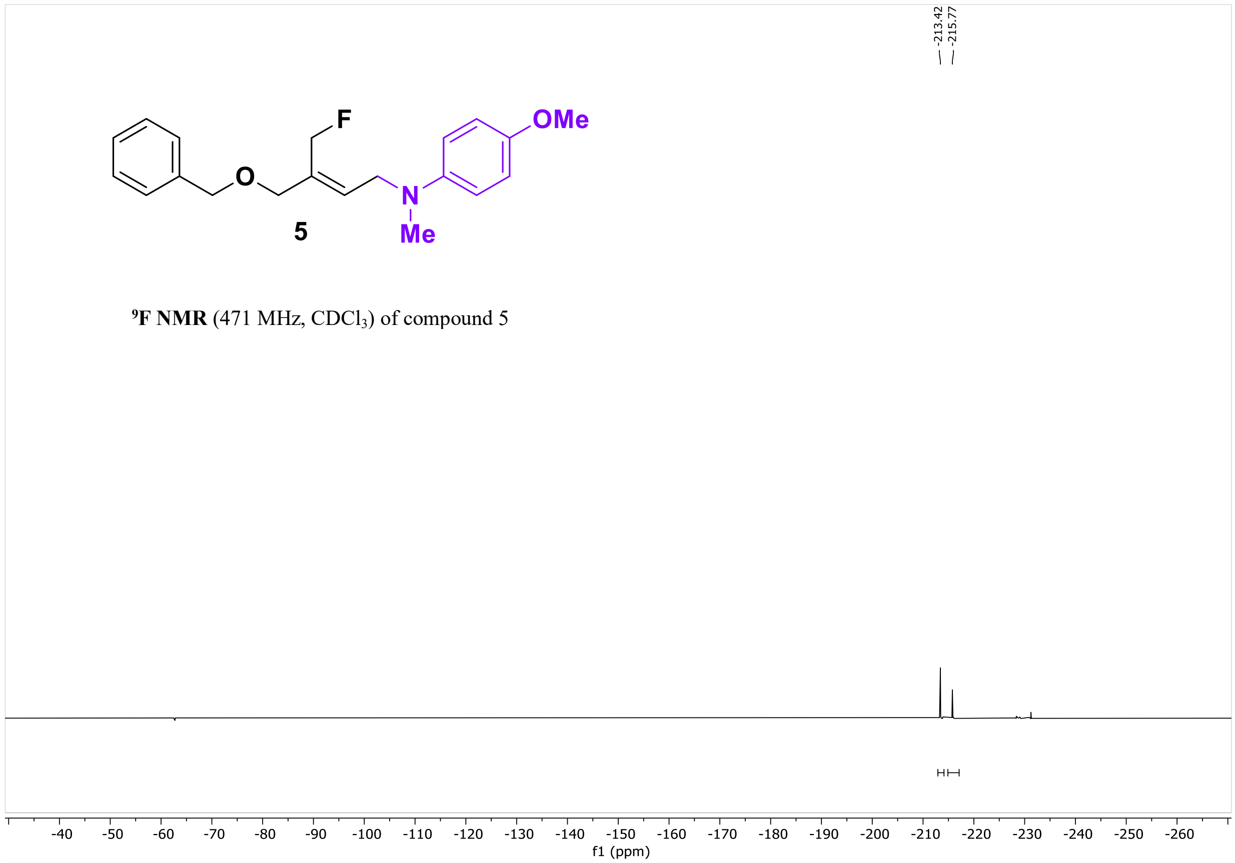

^{9}F NMR (471 MHz, $CDCl_3$) of compound 5

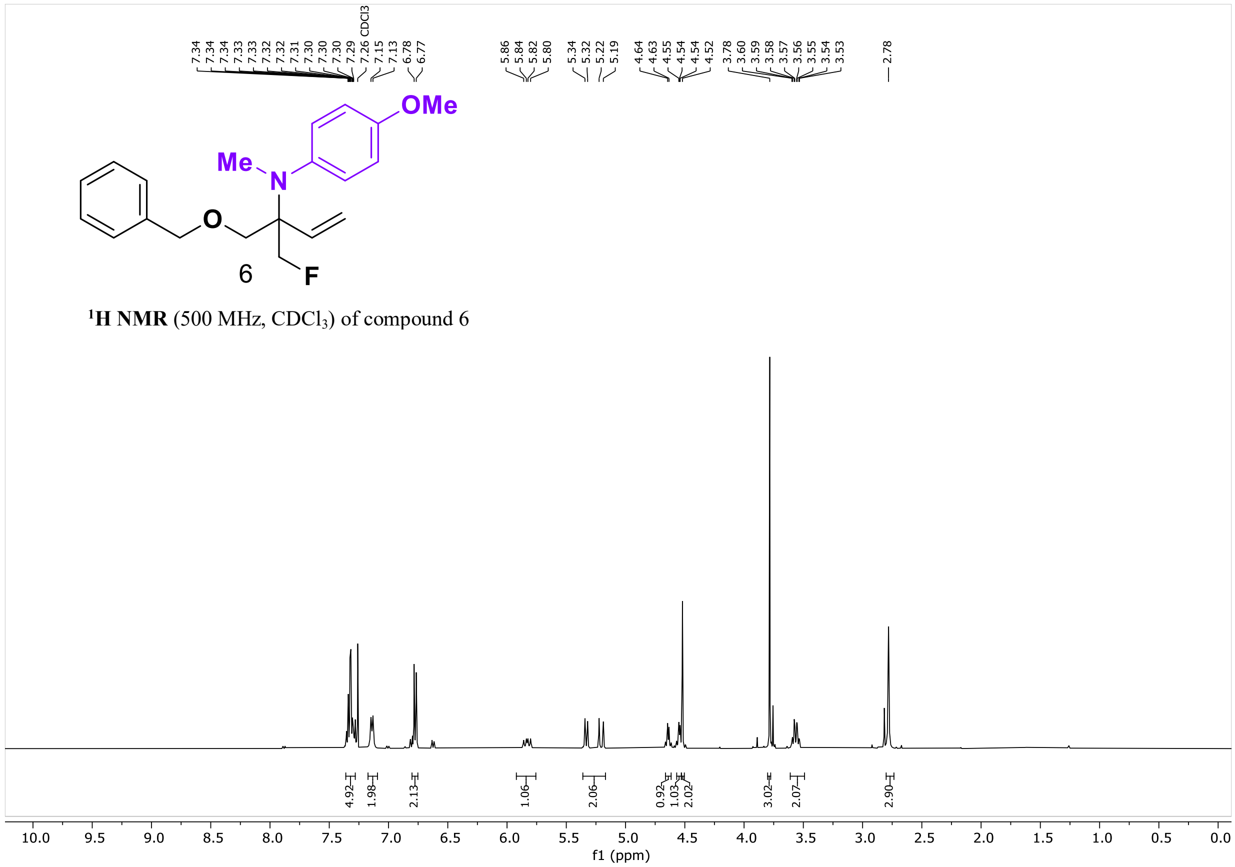

^{1}H NMR (500 MHz, $CDCl_3$) of compound 6

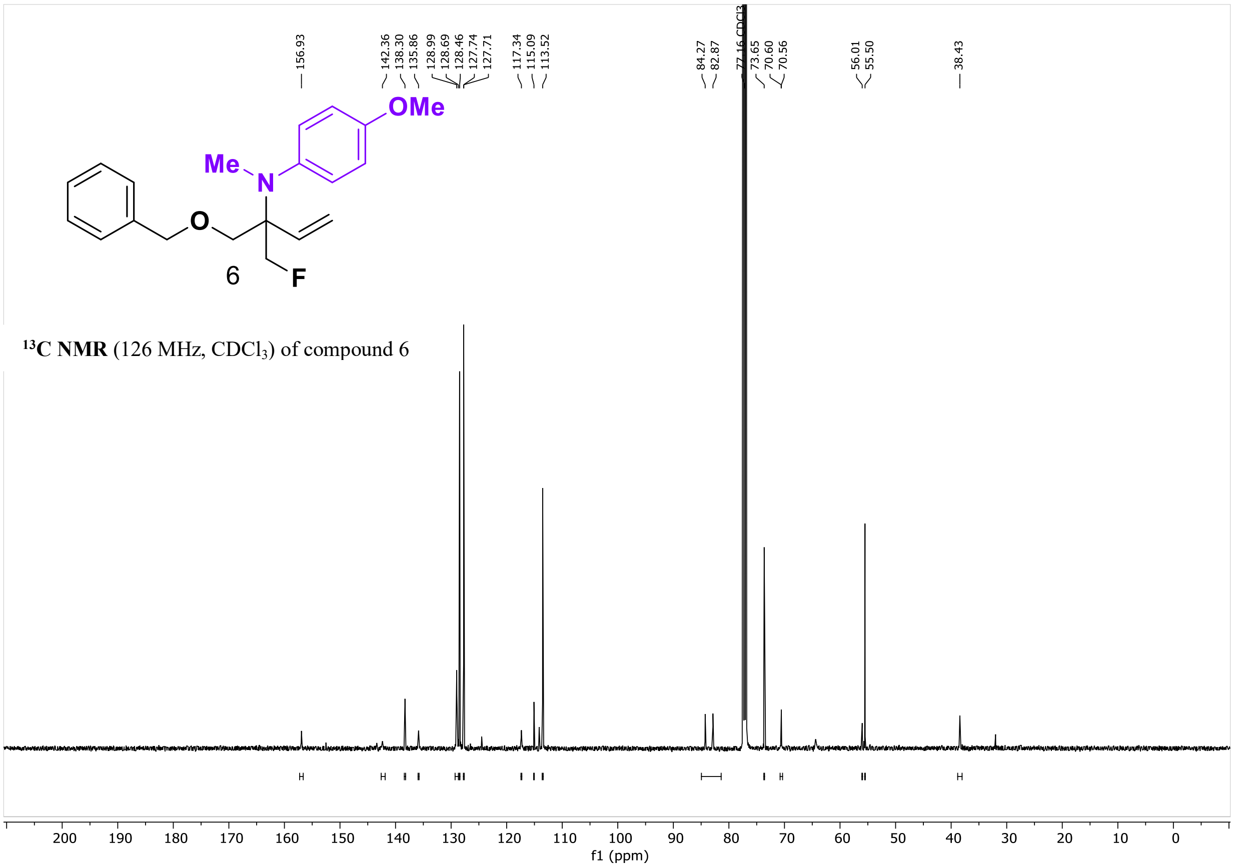

^{13}C NMR (126 MHz, $CDCl_3$) of compound 6

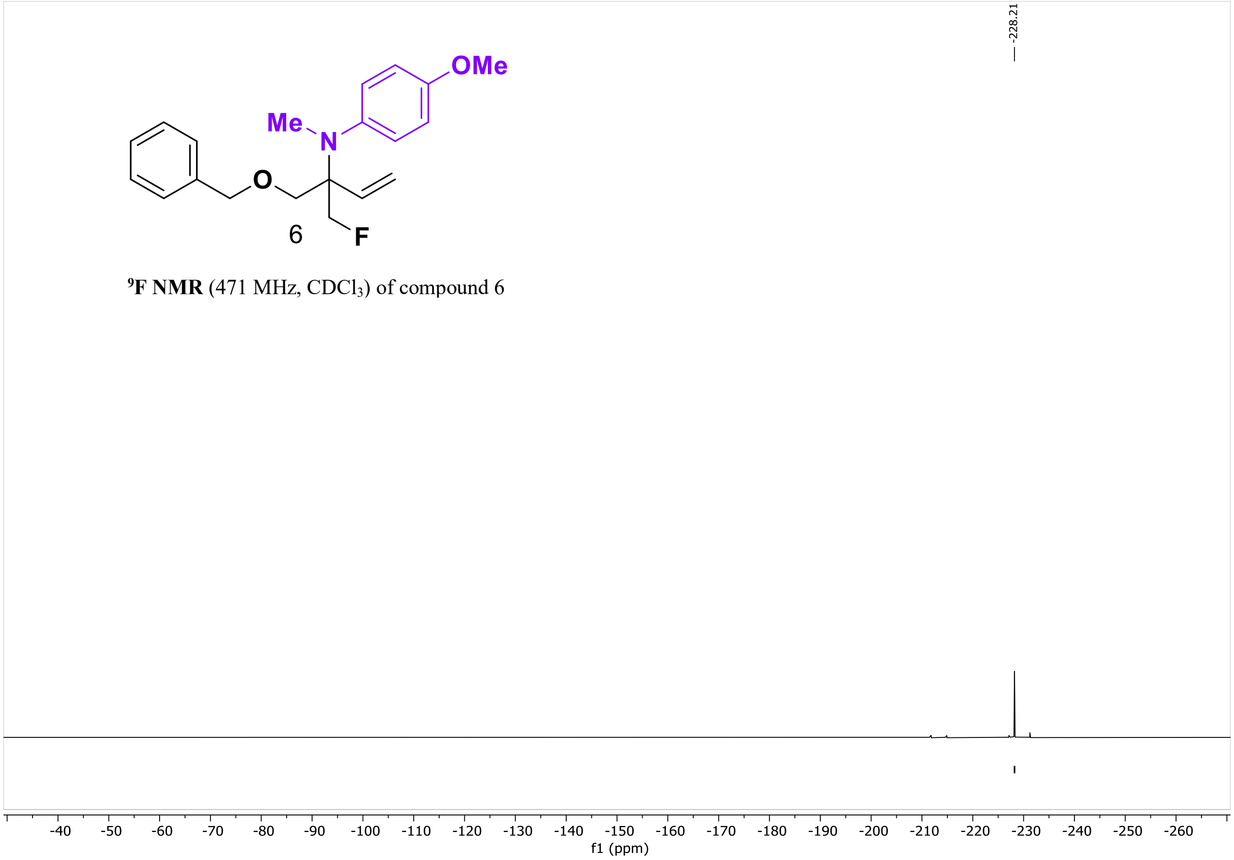

[9]F NMR (471 MHz, $CDCl_3$) of compound 6

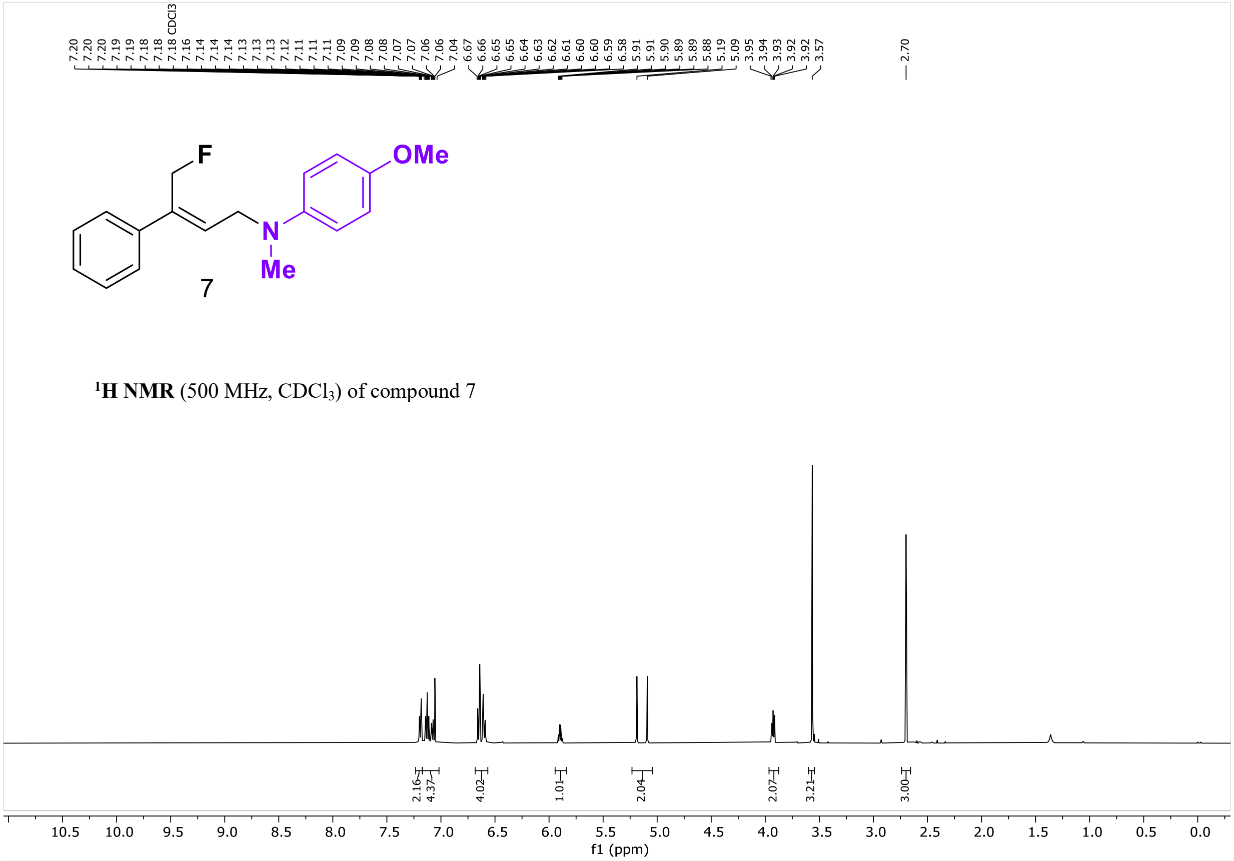

¹H NMR (500 MHz, $CDCl_3$) of compound 7

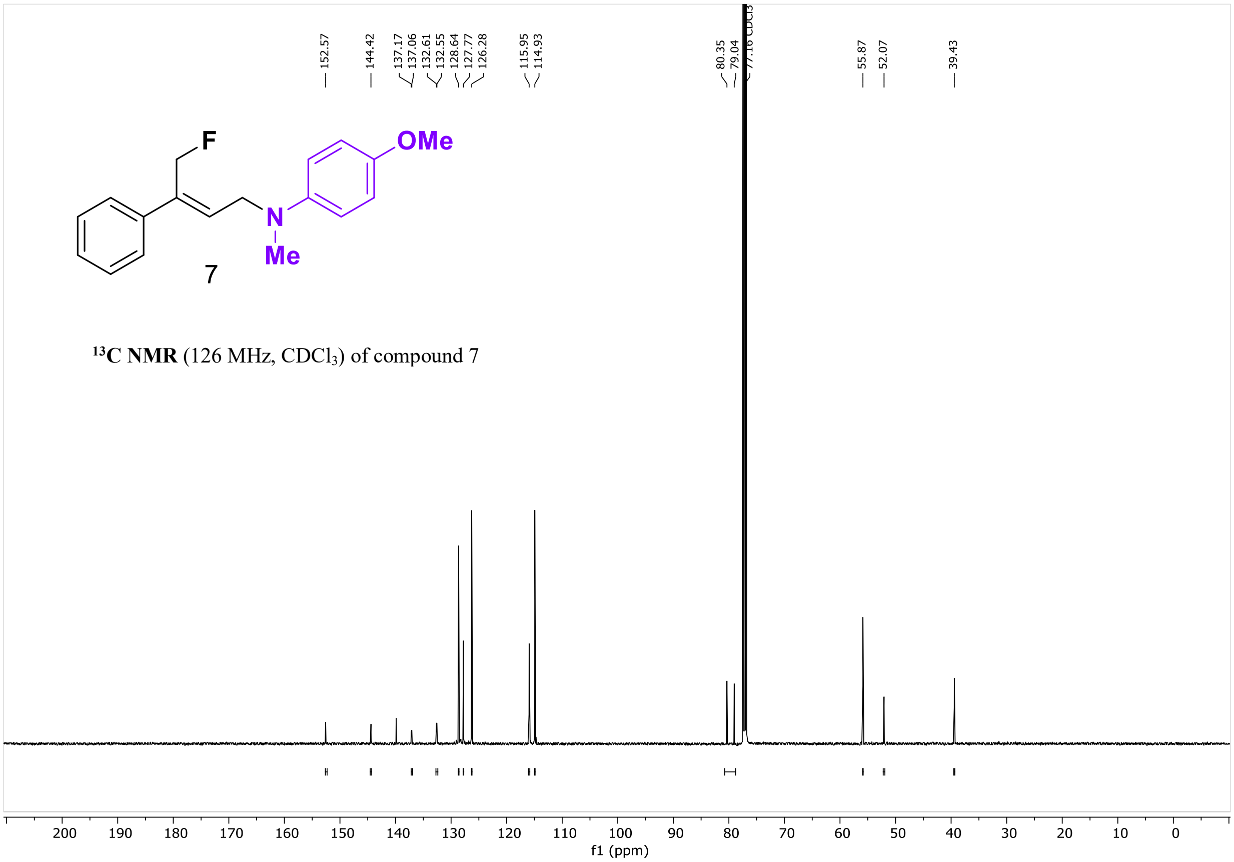

¹³C NMR (126 MHz, $CDCl_3$) of compound 7

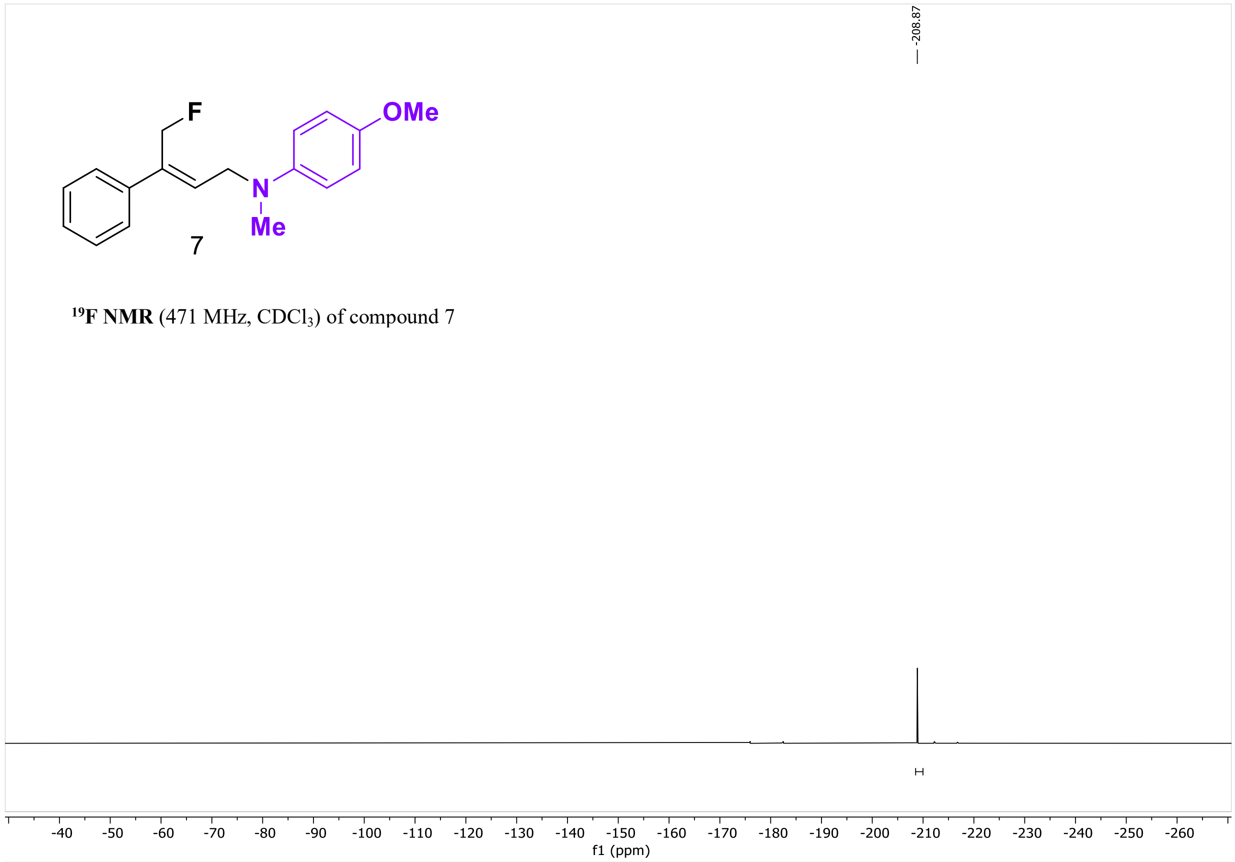

^{19}F NMR (471 MHz, $CDCl_3$) of compound 7

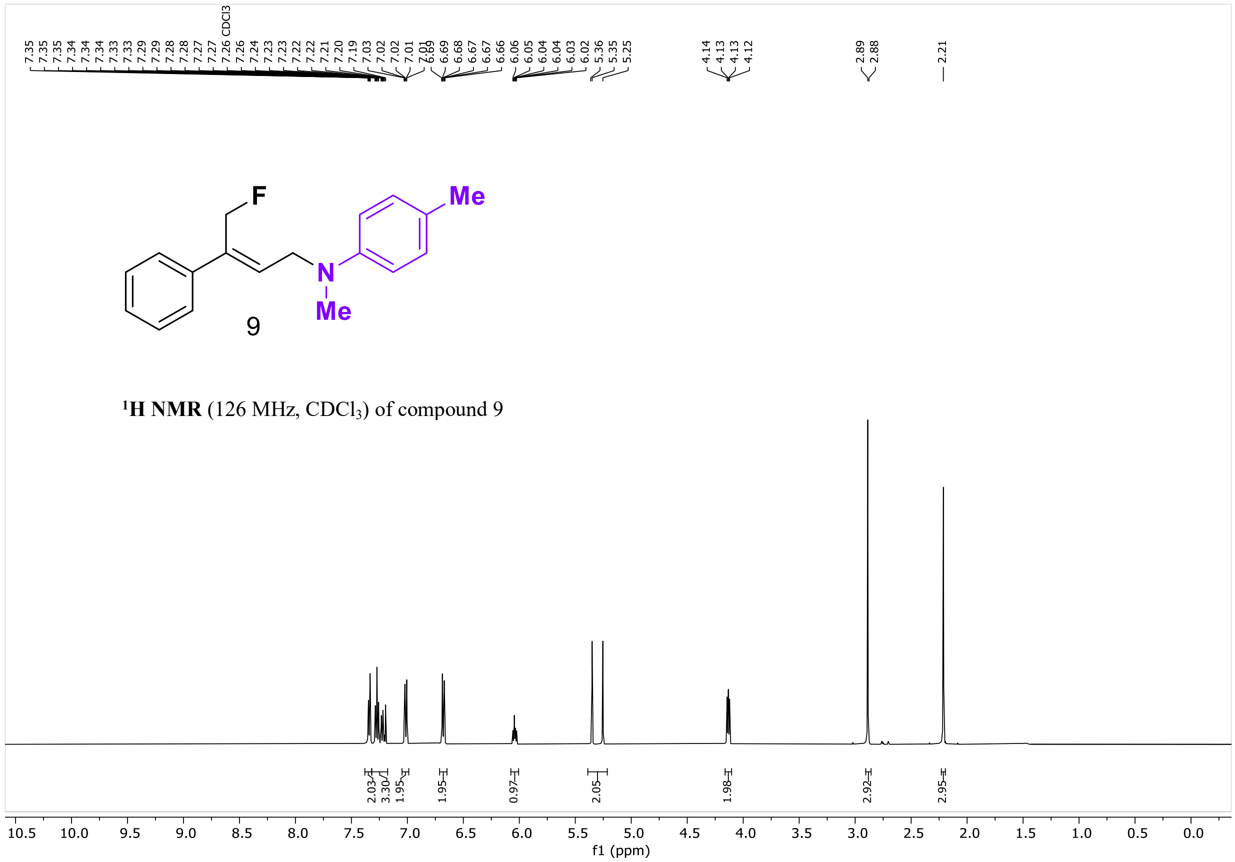

^{1}H NMR (126 MHz, $CDCl_3$) of compound 9

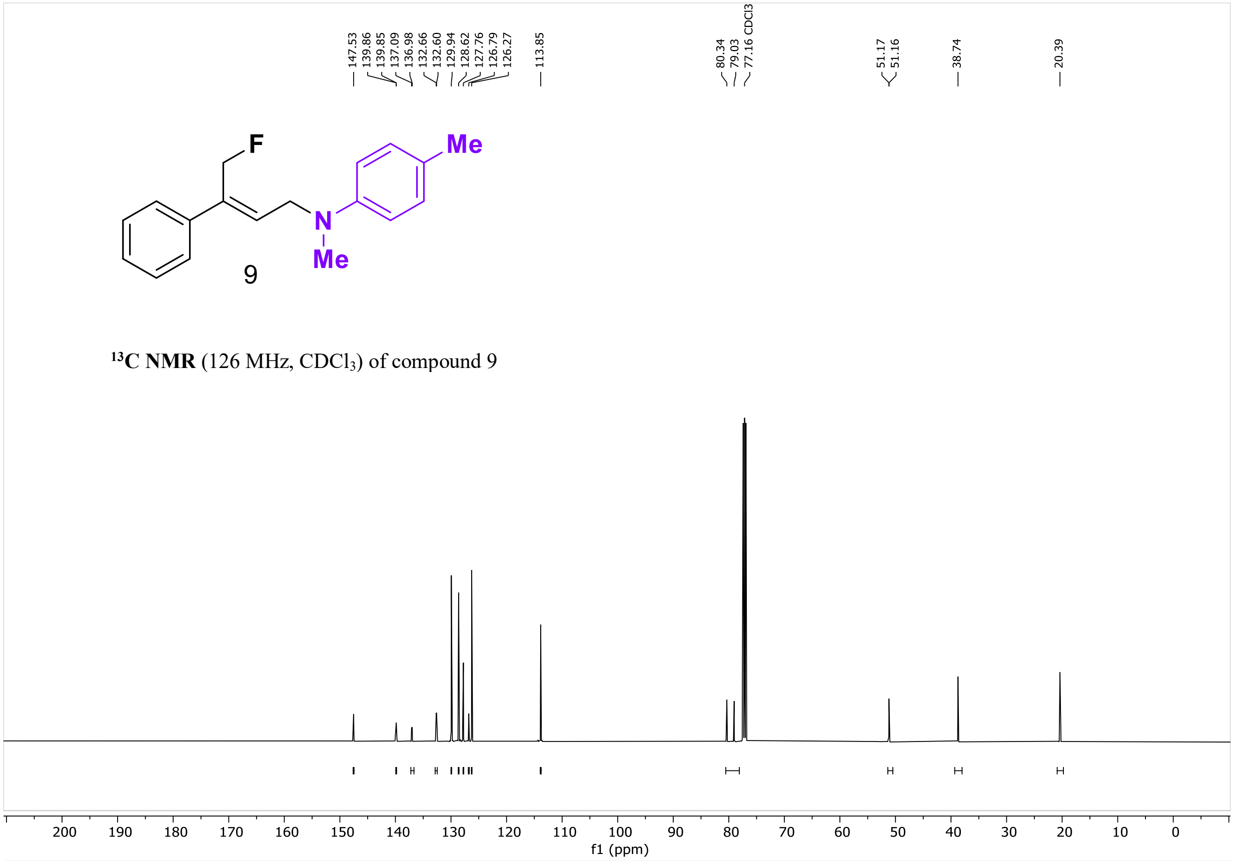

^{13}C NMR (126 MHz, $CDCl_3$) of compound 9

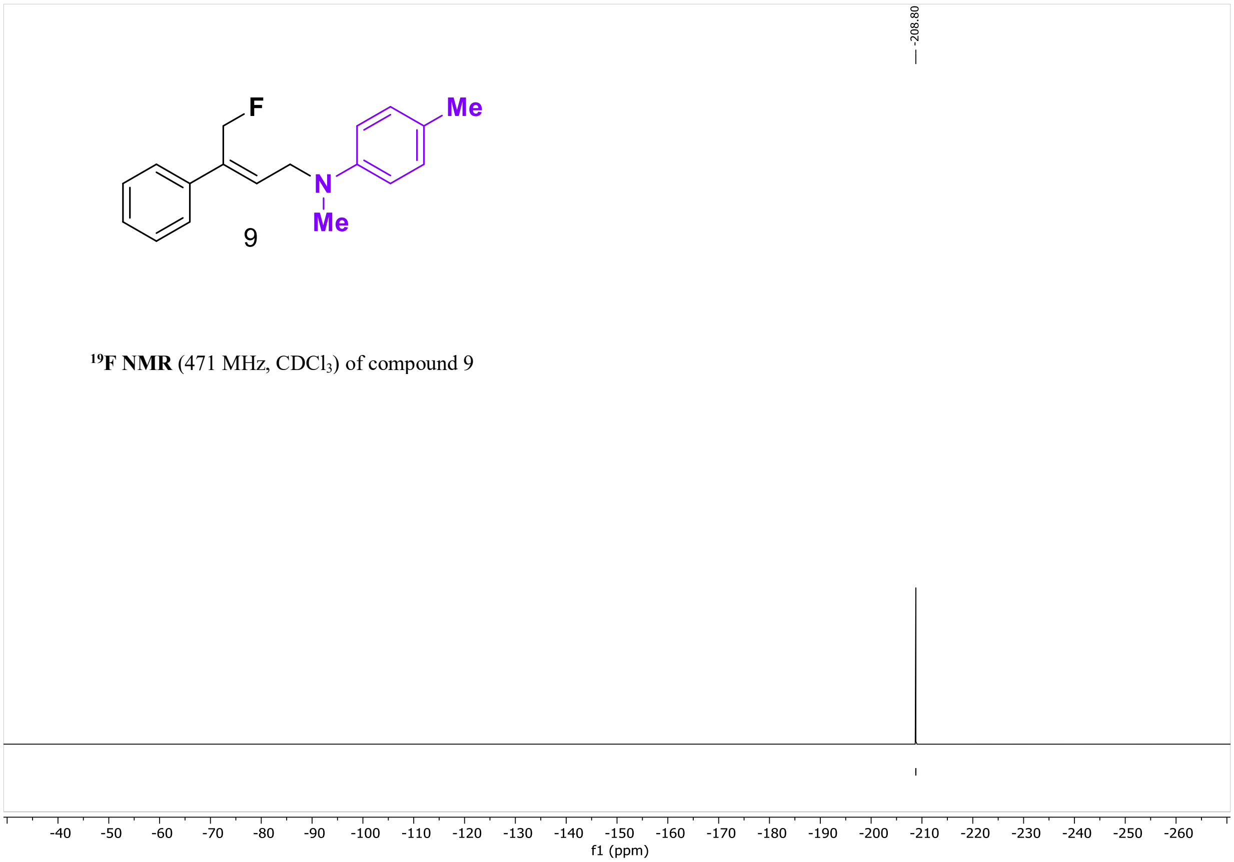

^{19}F NMR (471 MHz, $CDCl_3$) of compound 9

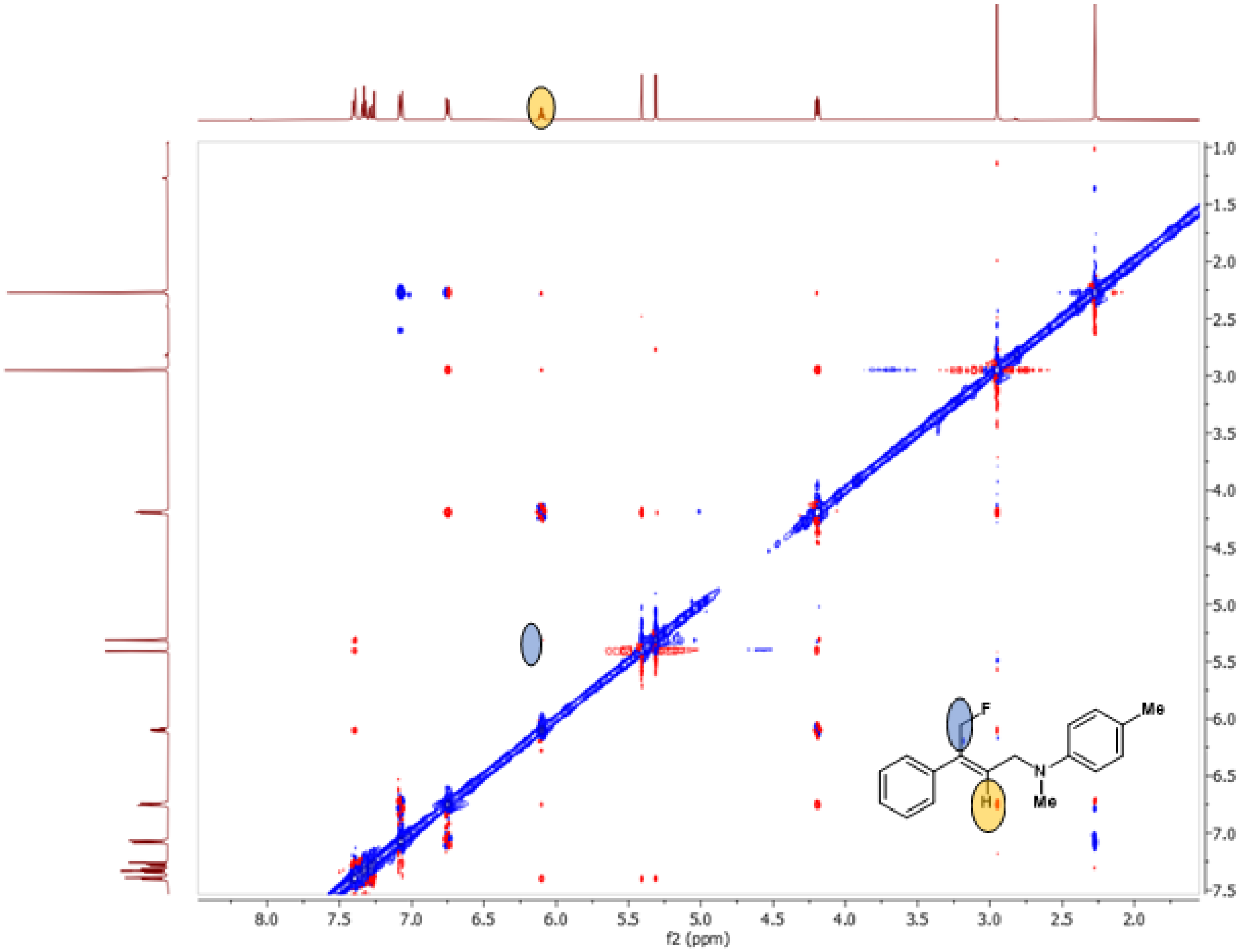

2D-ROESY NMR of compound 9 (Z-isomer)

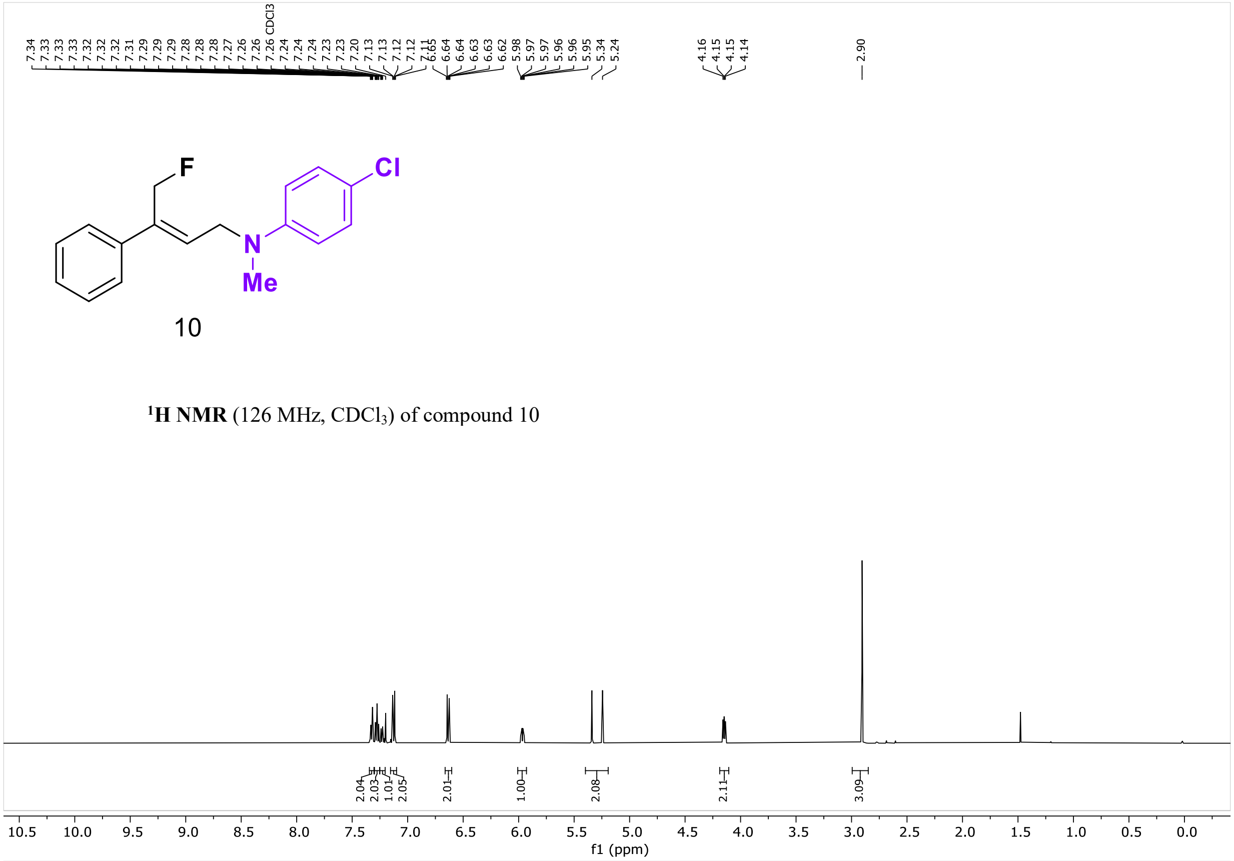

^{1}H NMR (126 MHz, $CDCl_3$) of compound 10

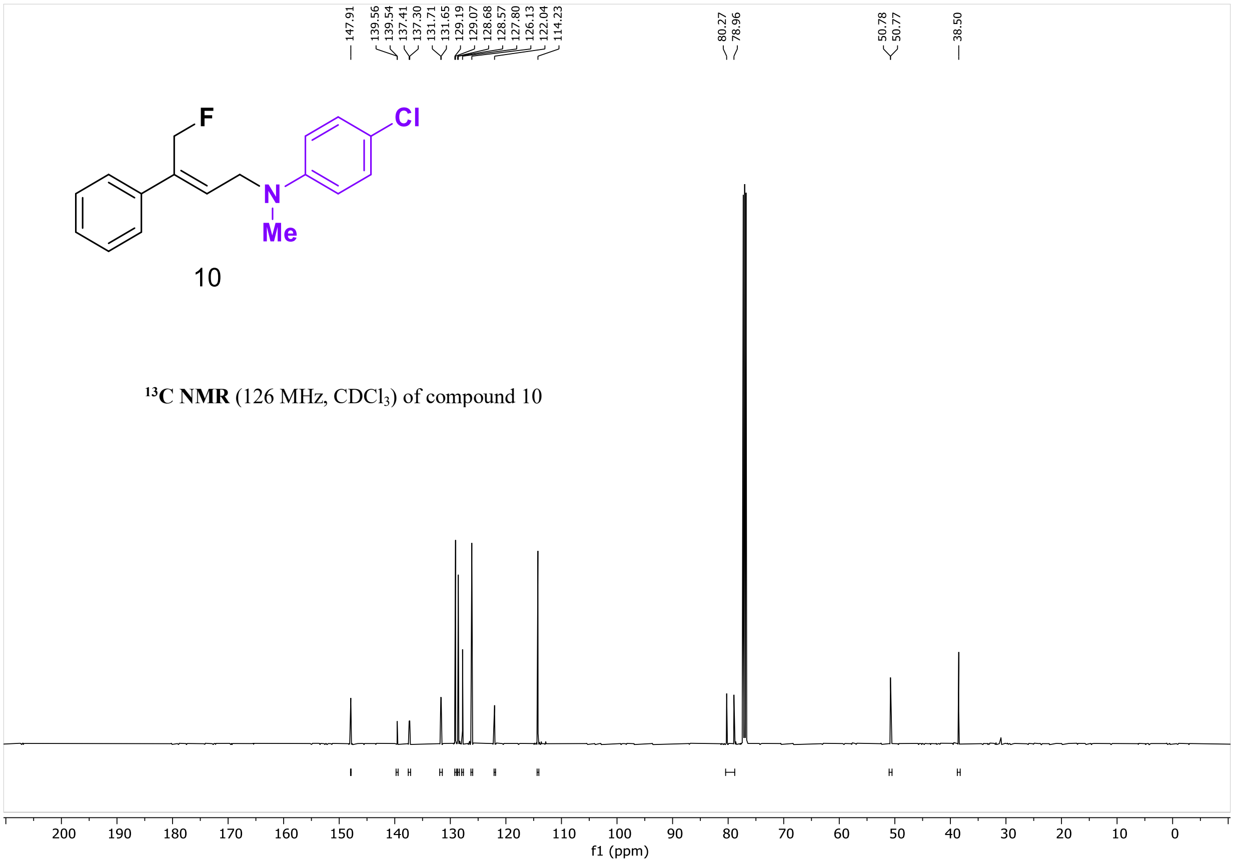

^{13}C NMR (126 MHz, $CDCl_3$) of compound 10

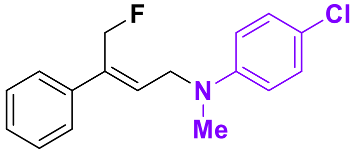

10

^{19}F NMR (471 MHz, $CDCl_3$) of compound 10

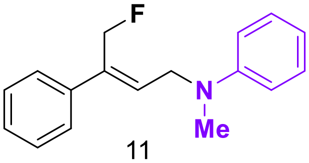

11

^{1}H NMR (126 MHz, $CDCl_3$) of compound 11

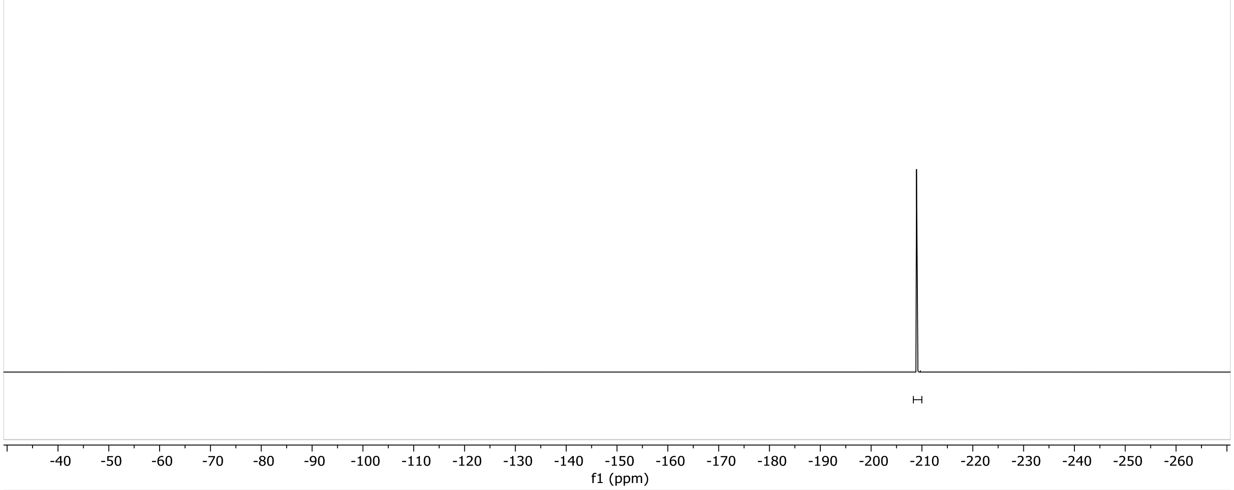

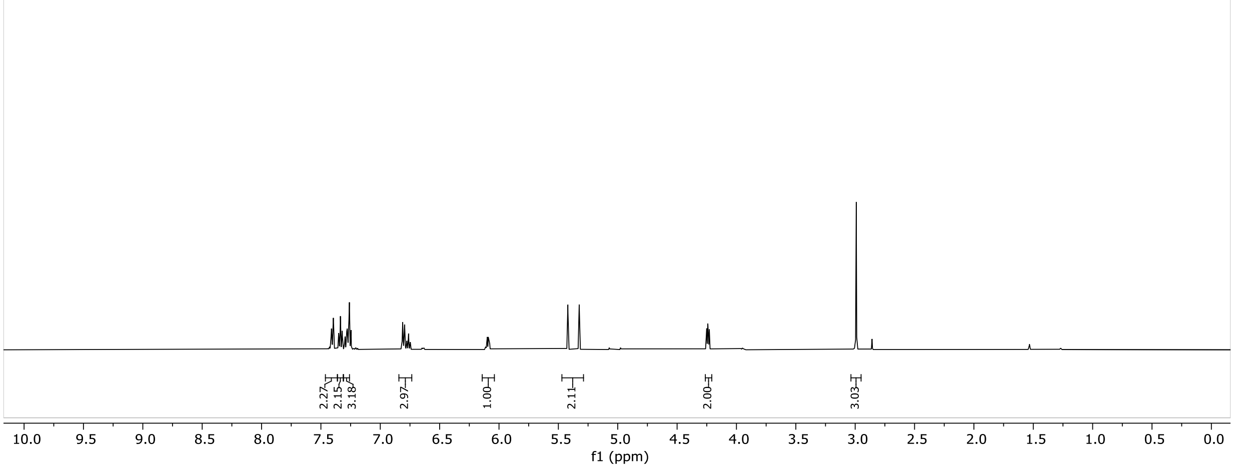

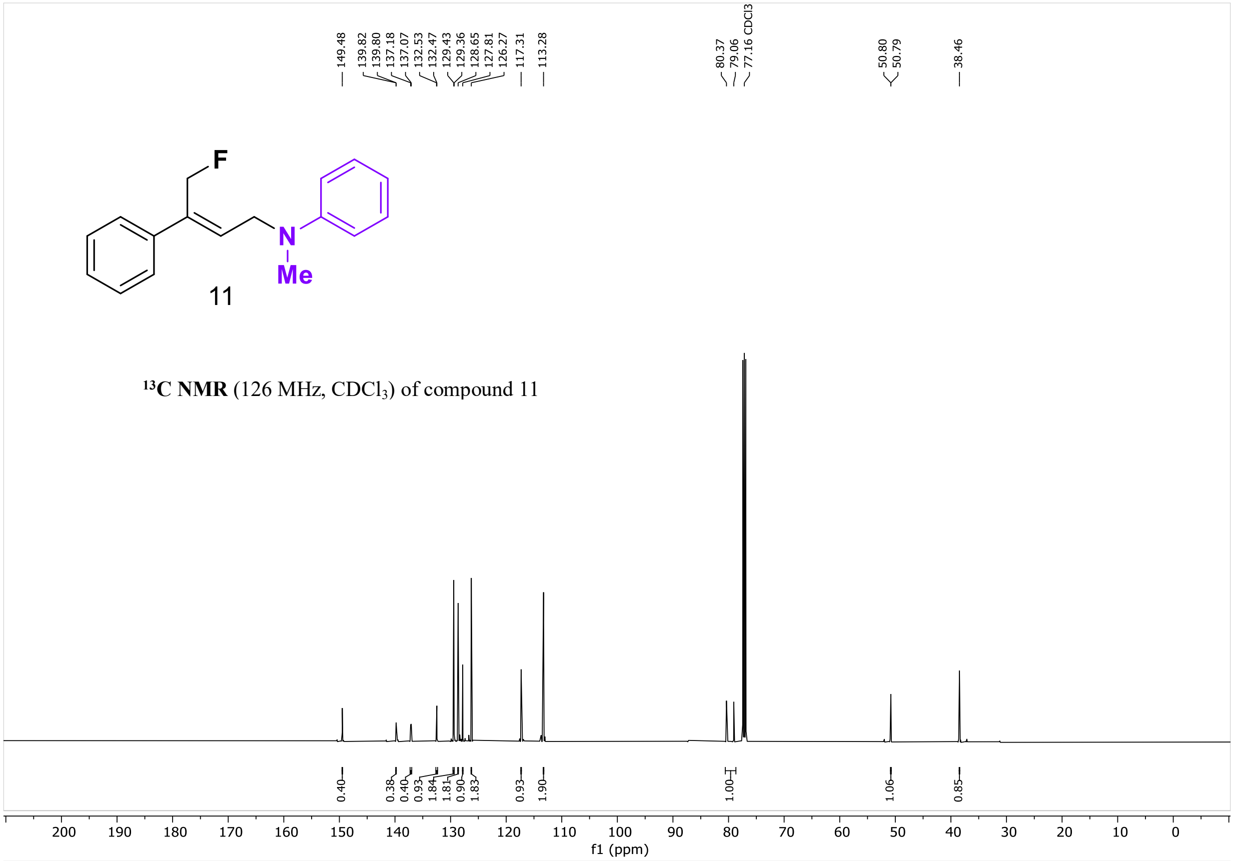

^{13}**C NMR** (126 MHz, $CDCl_3$) of compound 11

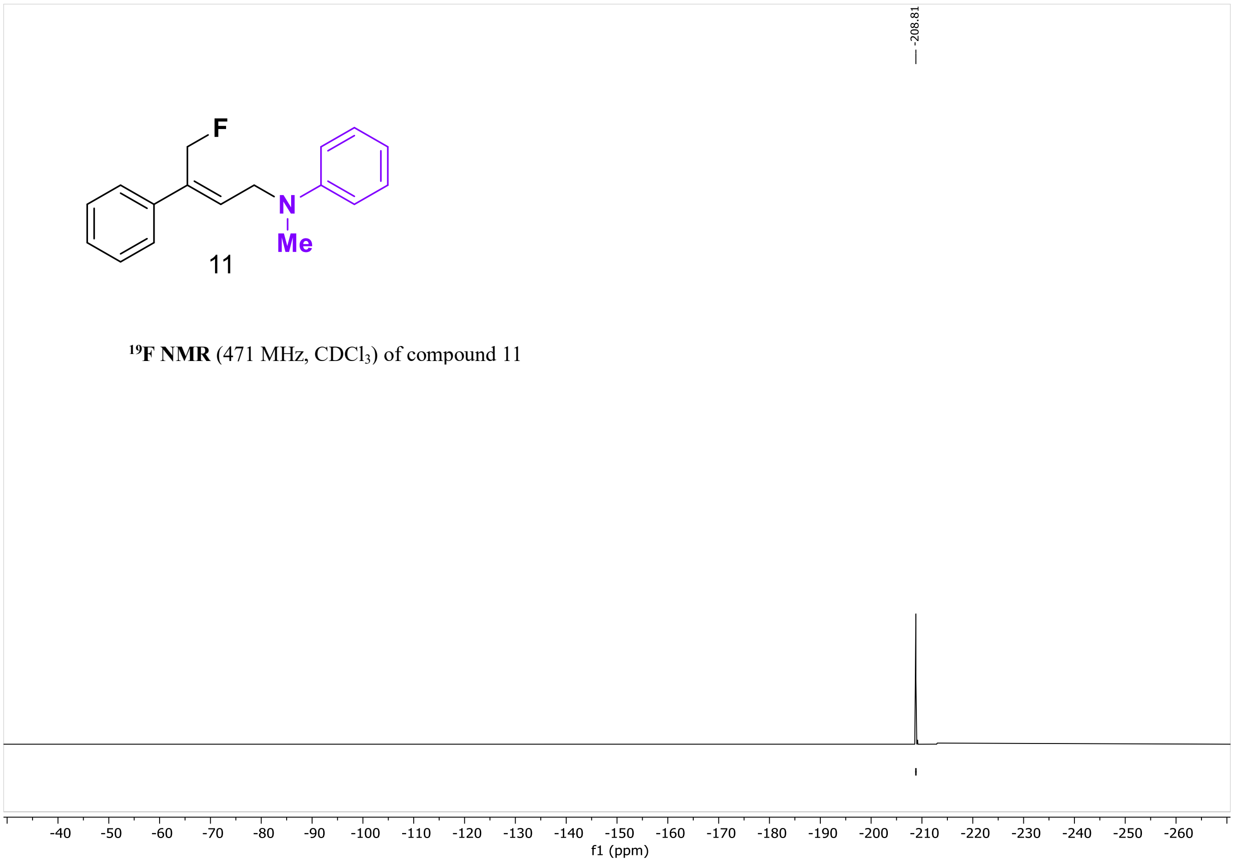

^{19}**F NMR** (471 MHz, $CDCl_3$) of compound 11

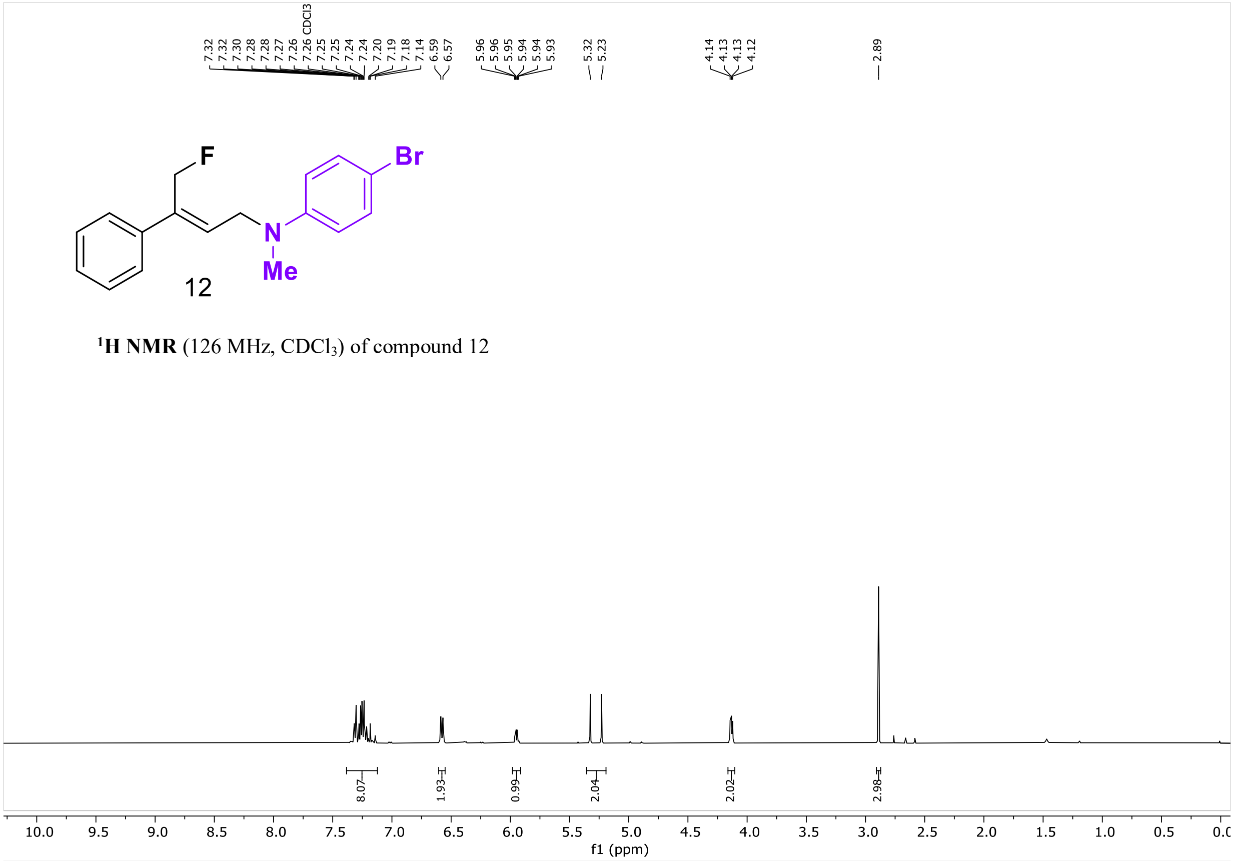

¹H NMR (126 MHz, $CDCl_3$) of compound 12

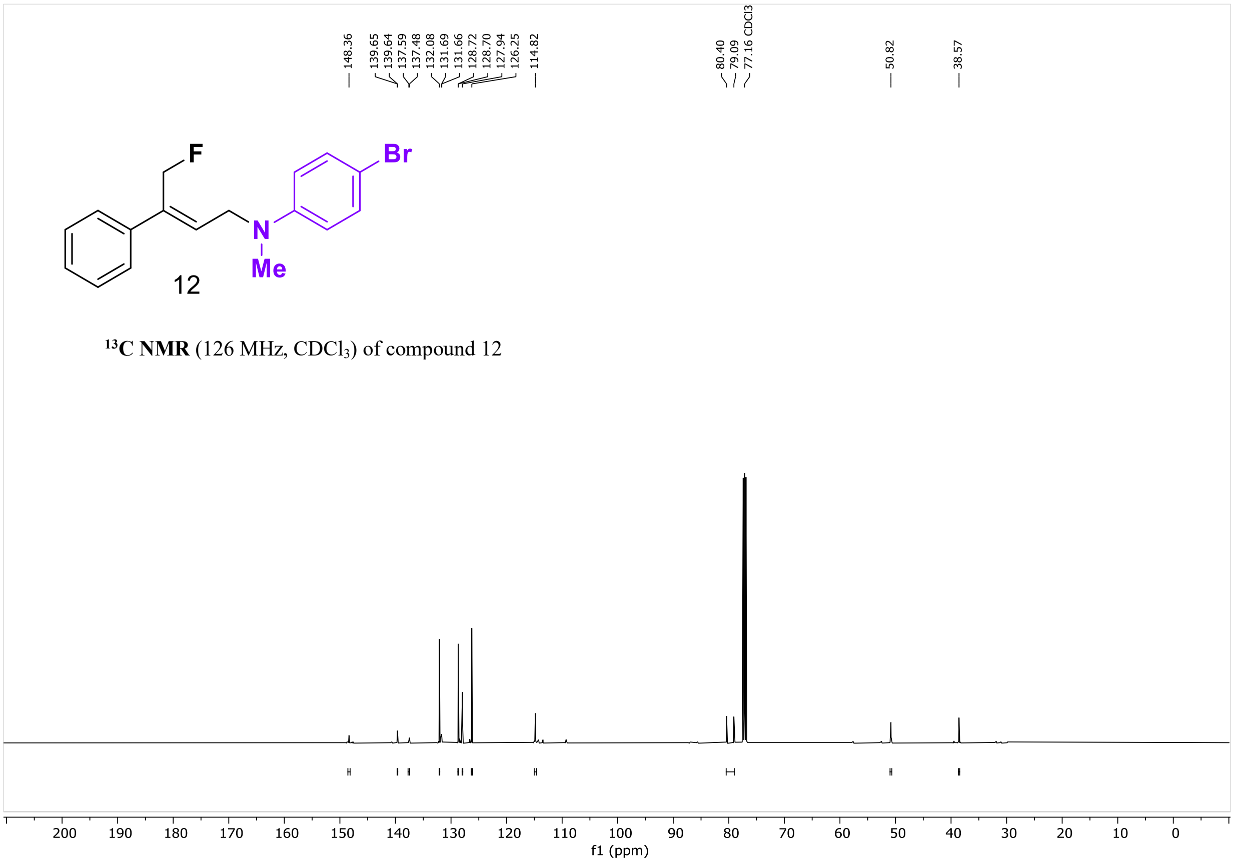

¹³C NMR (126 MHz, $CDCl_3$) of compound 12

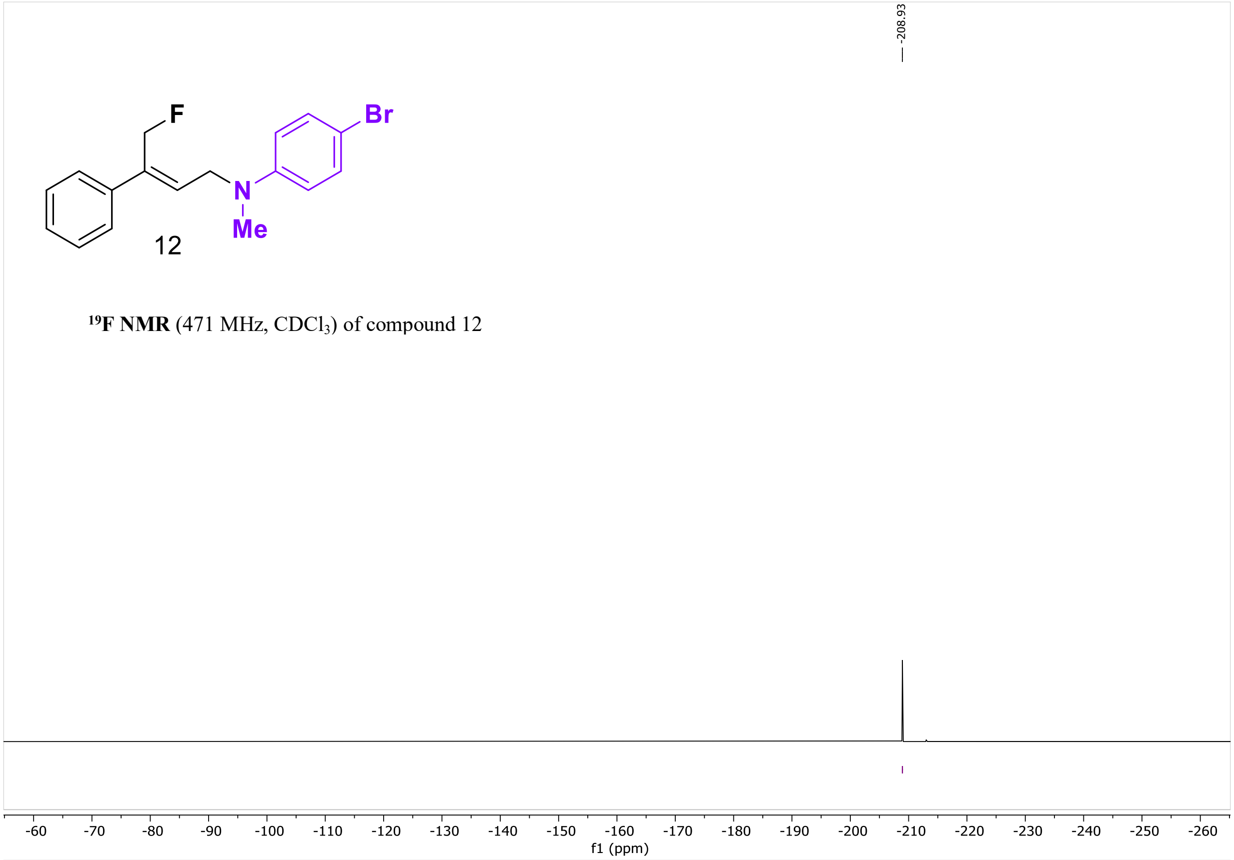

^{19}F NMR (471 MHz, $CDCl_3$) of compound 12

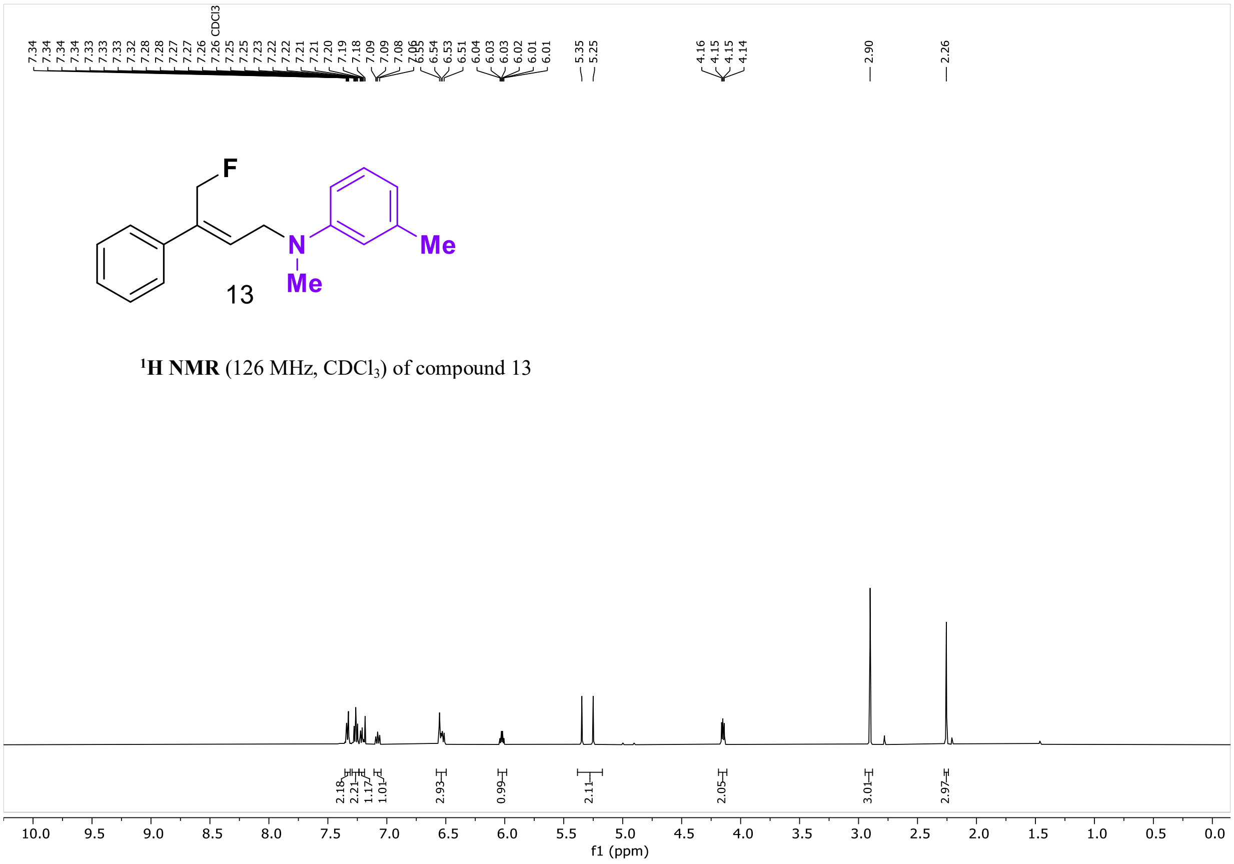

^{1}H NMR (126 MHz, $CDCl_3$) of compound 13

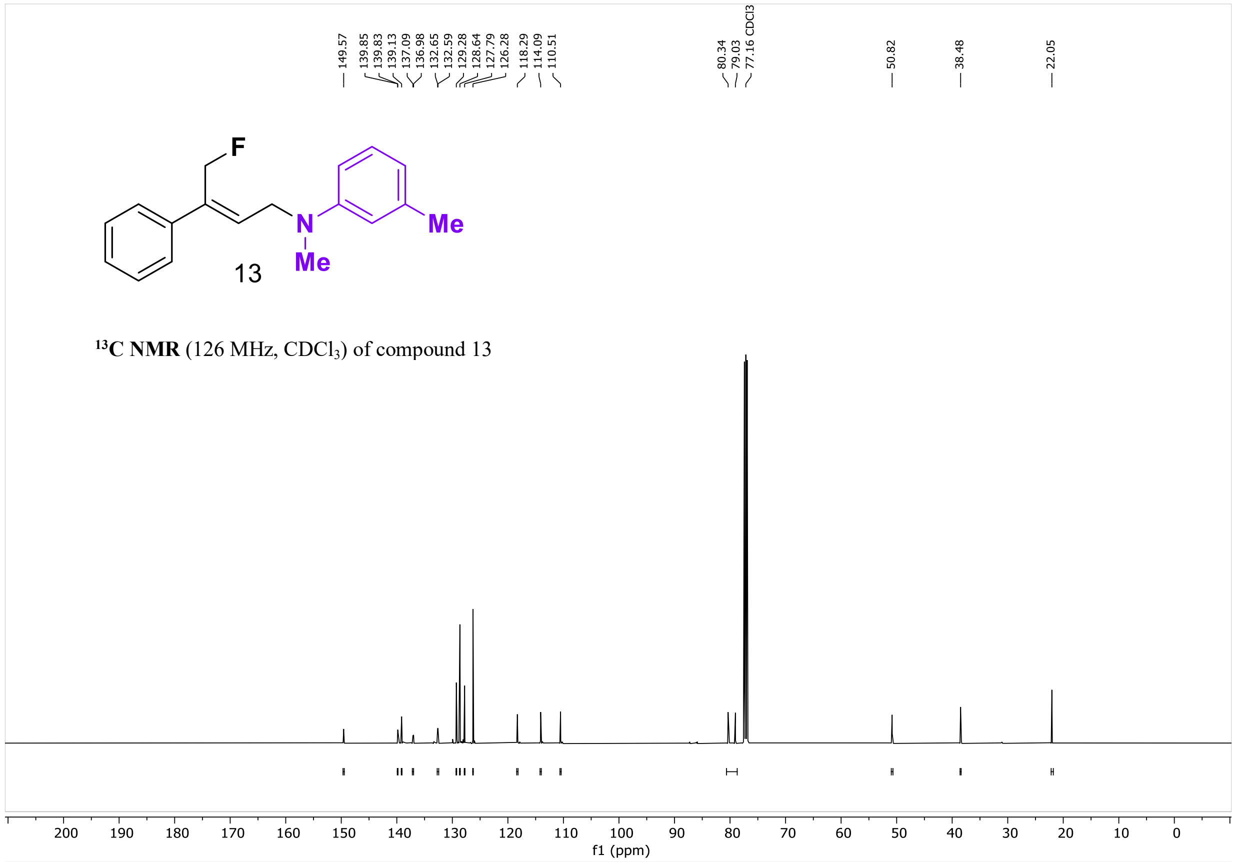

^{13}**C NMR** (126 MHz, $CDCl_3$) of compound 13

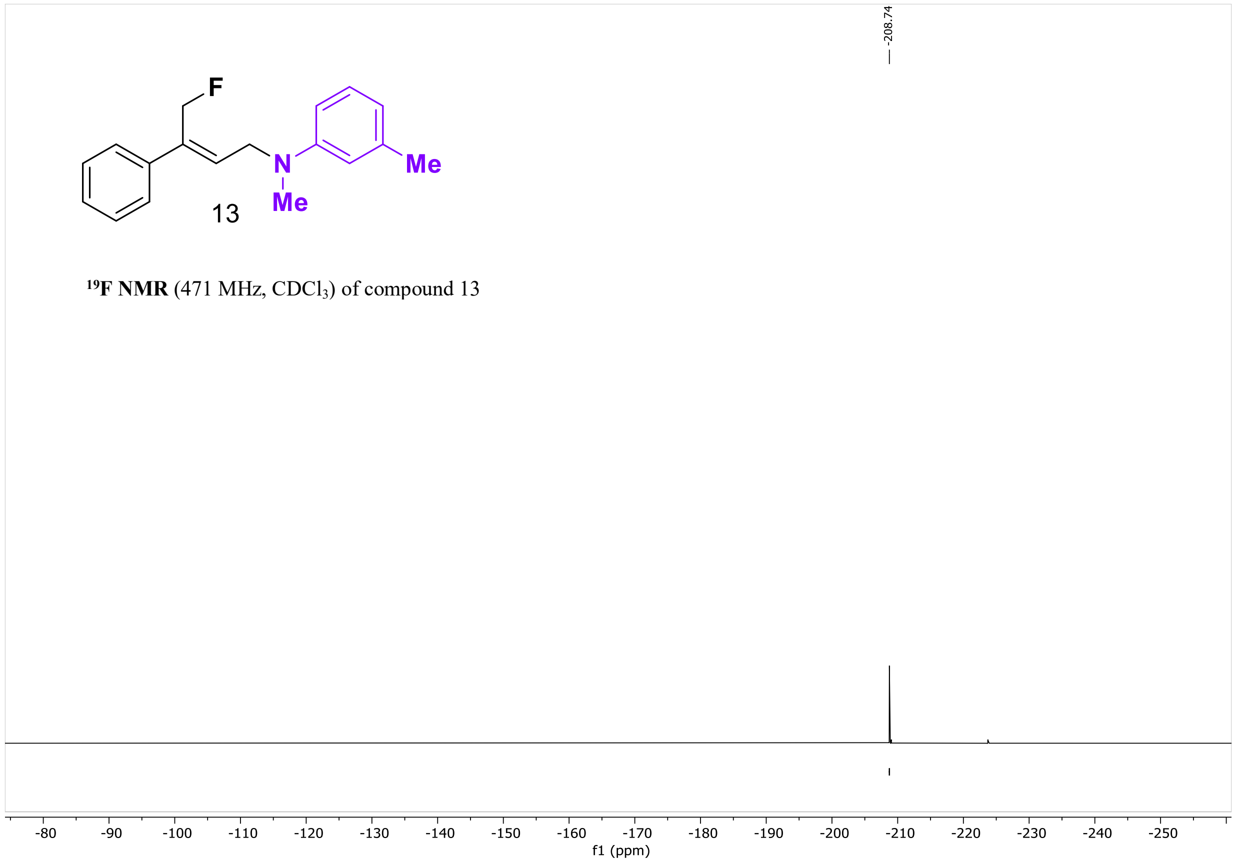

^{19}**F NMR** (471 MHz, $CDCl_3$) of compound 13

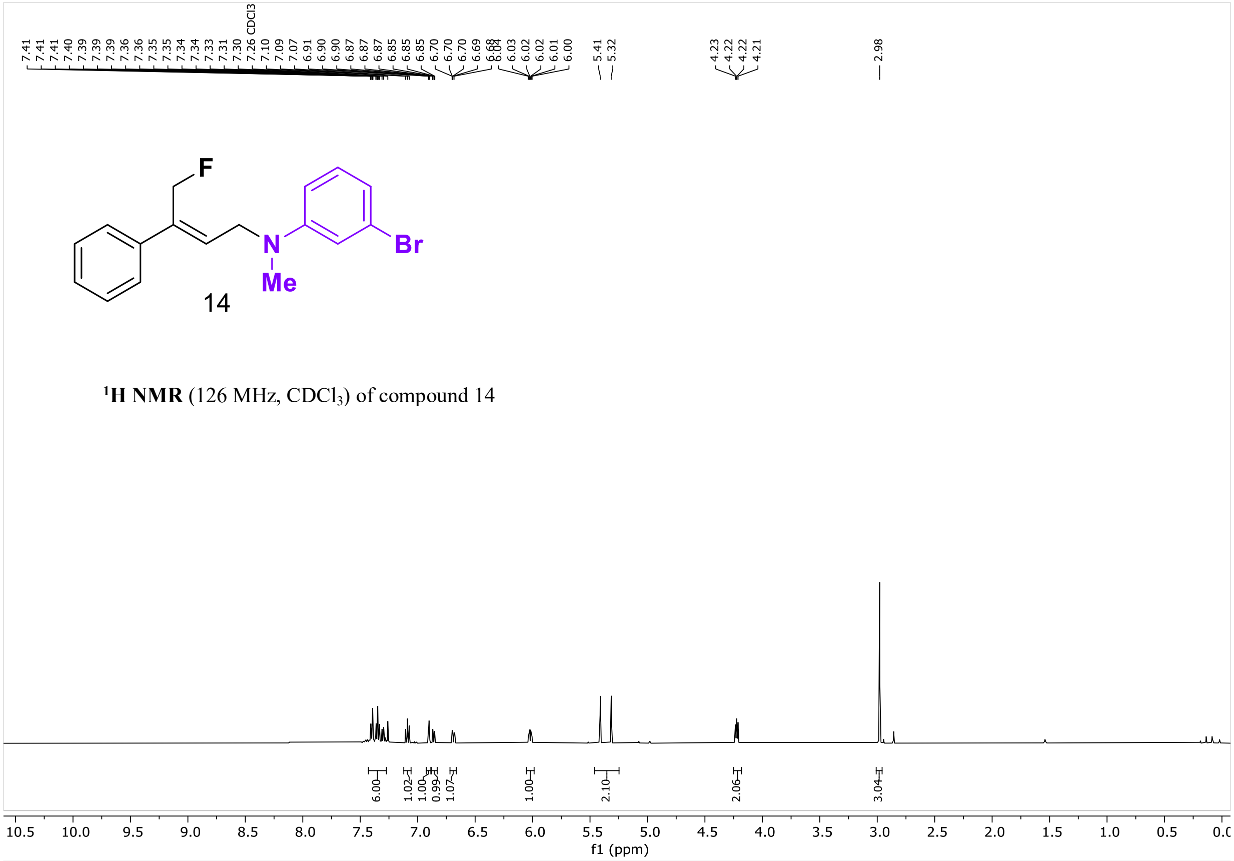

^{1}H NMR (126 MHz, $CDCl_3$) of compound 14

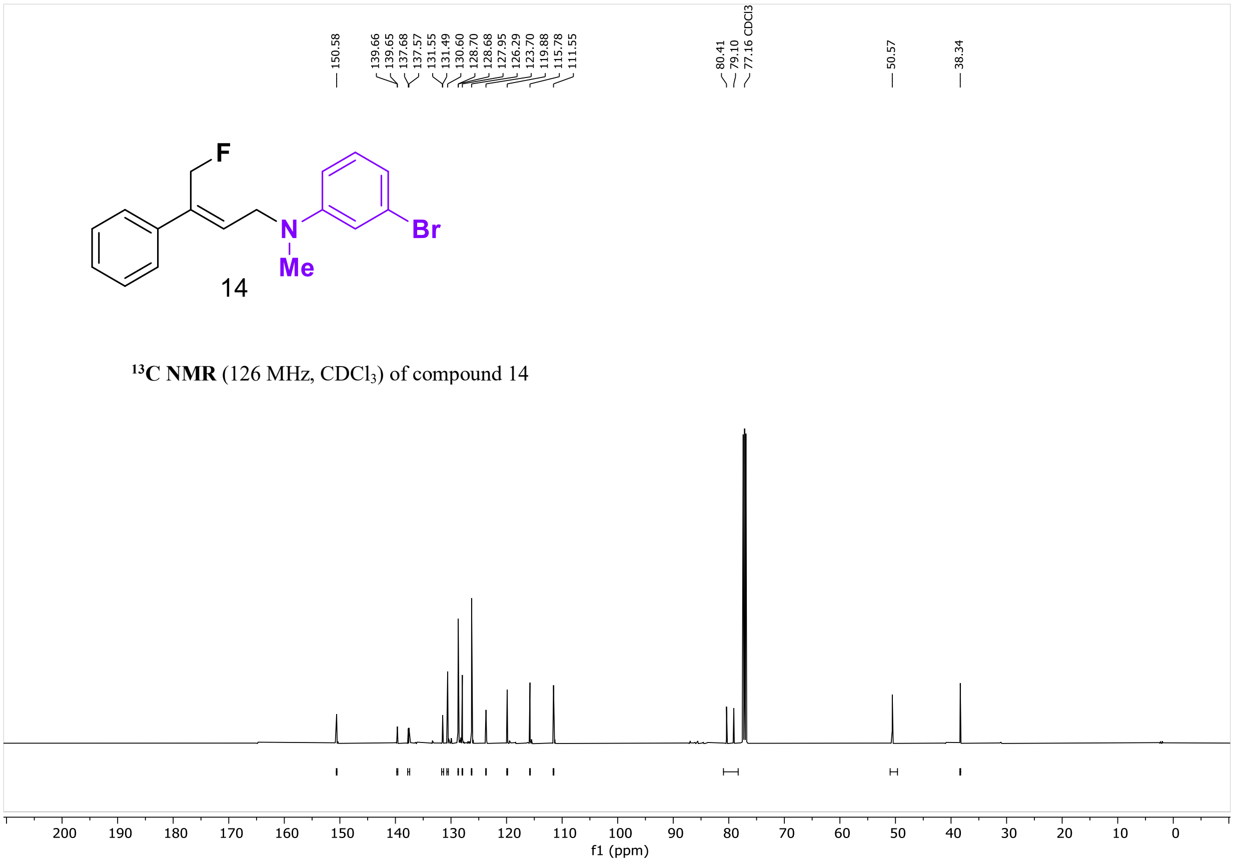

^{13}C NMR (126 MHz, $CDCl_3$) of compound 14

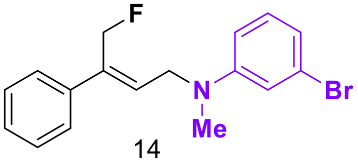

14

^{19}F NMR (471 MHz, $CDCl_3$) of compound 14

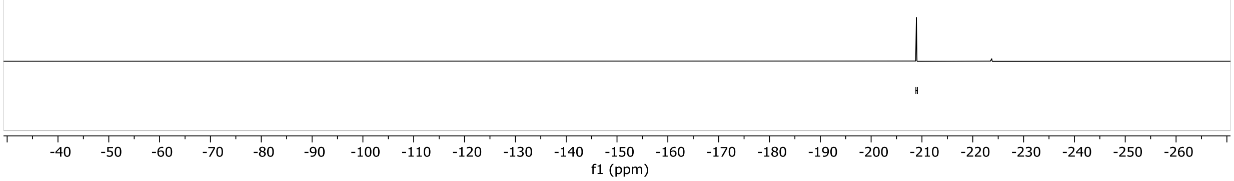

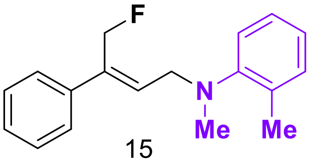

15

^{1}H NMR (126 MHz, $CDCl_3$) of compound 15

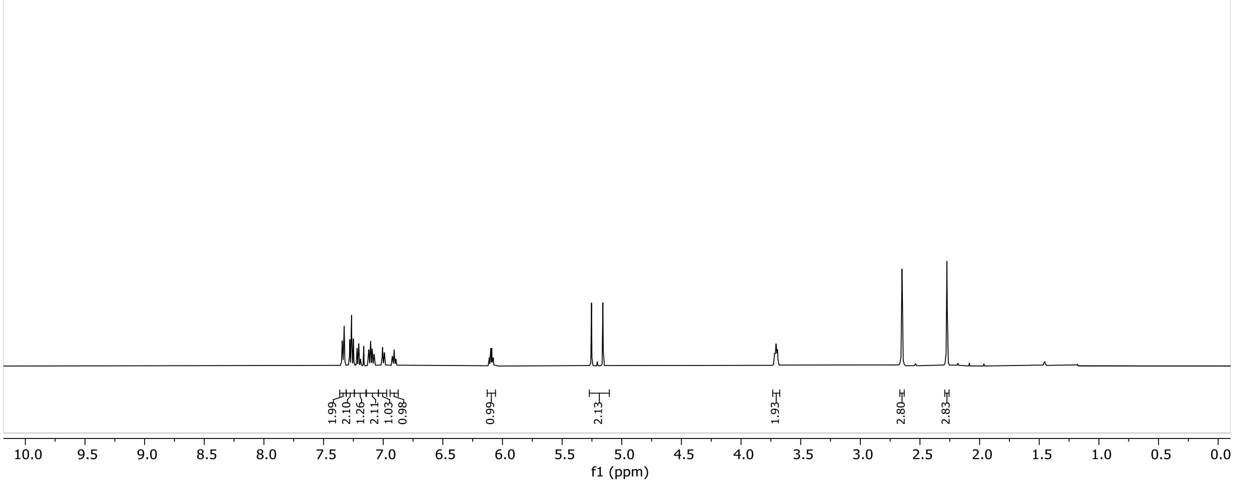

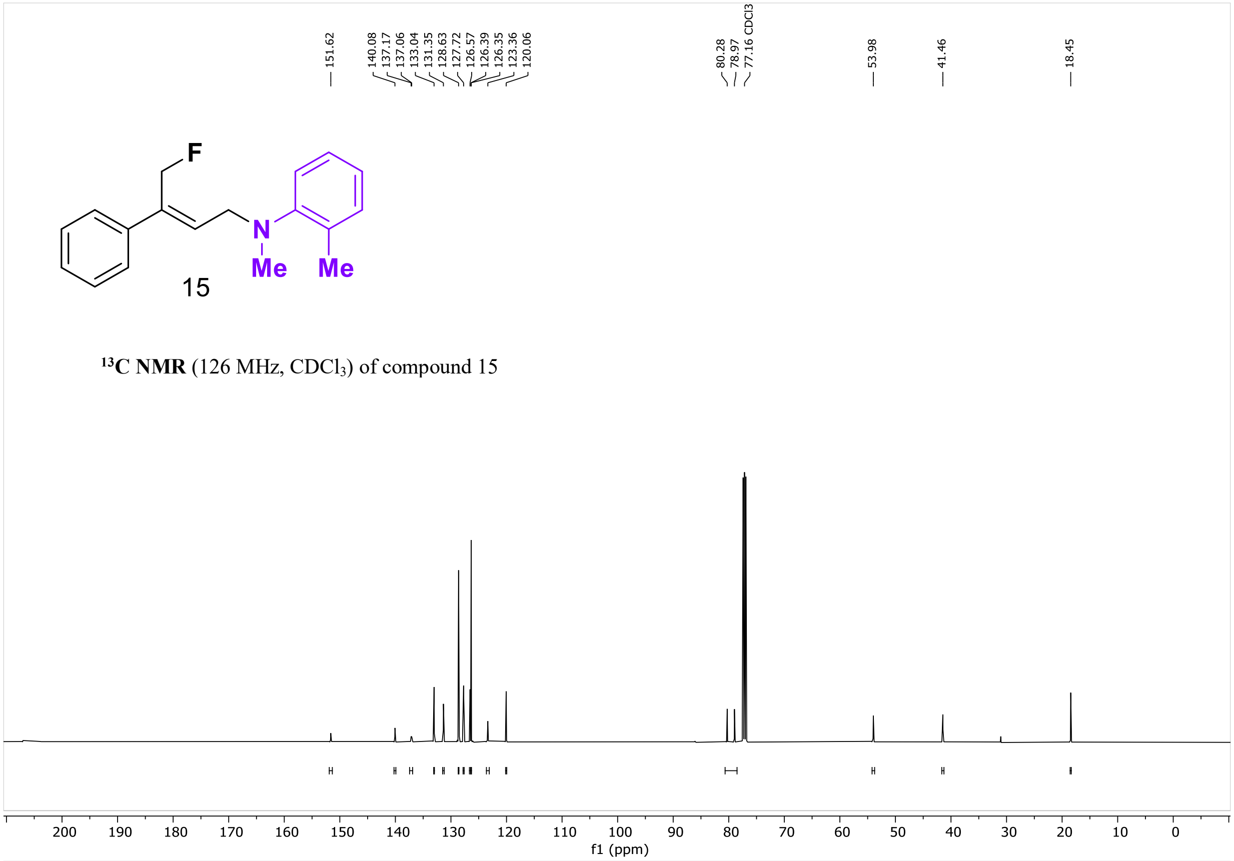

^{13}**C NMR** (126 MHz, $CDCl_3$) of compound 15

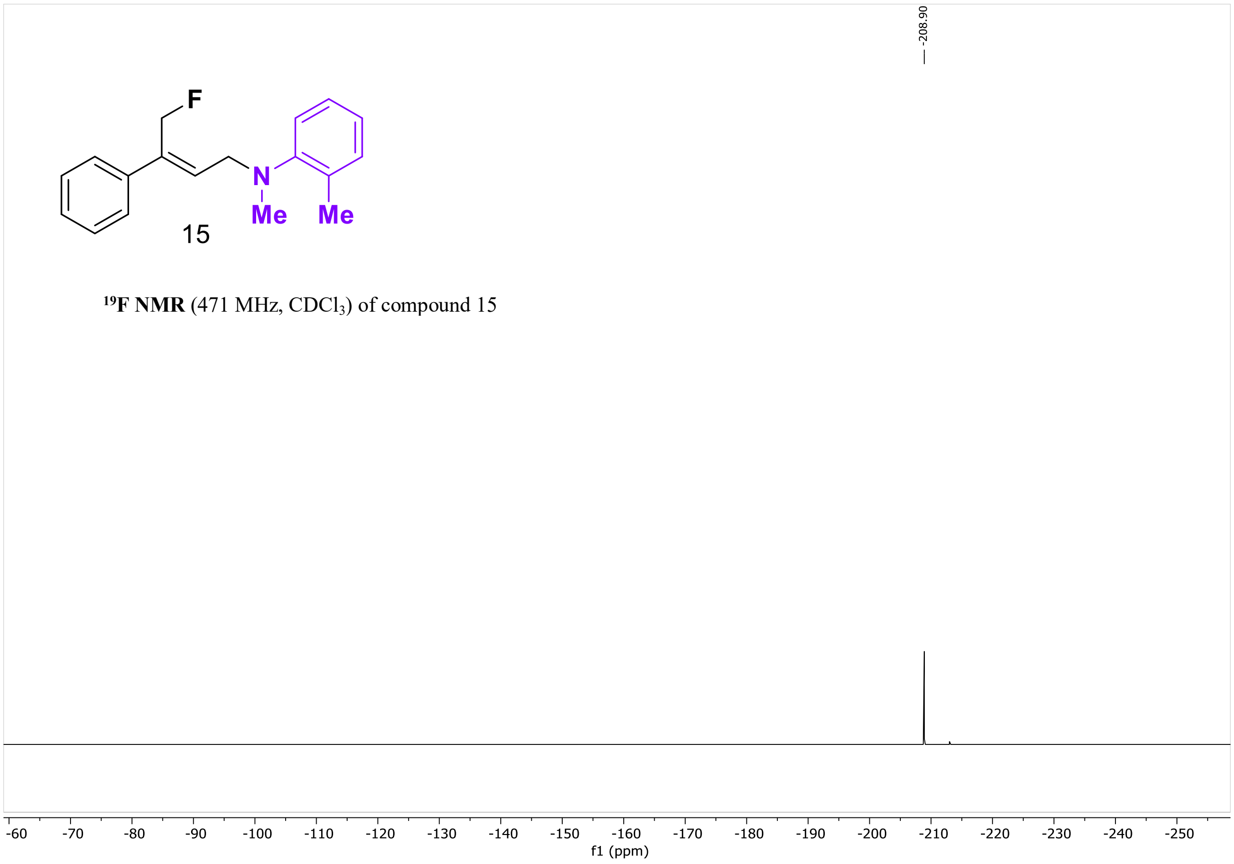

^{19}**F NMR** (471 MHz, $CDCl_3$) of compound 15

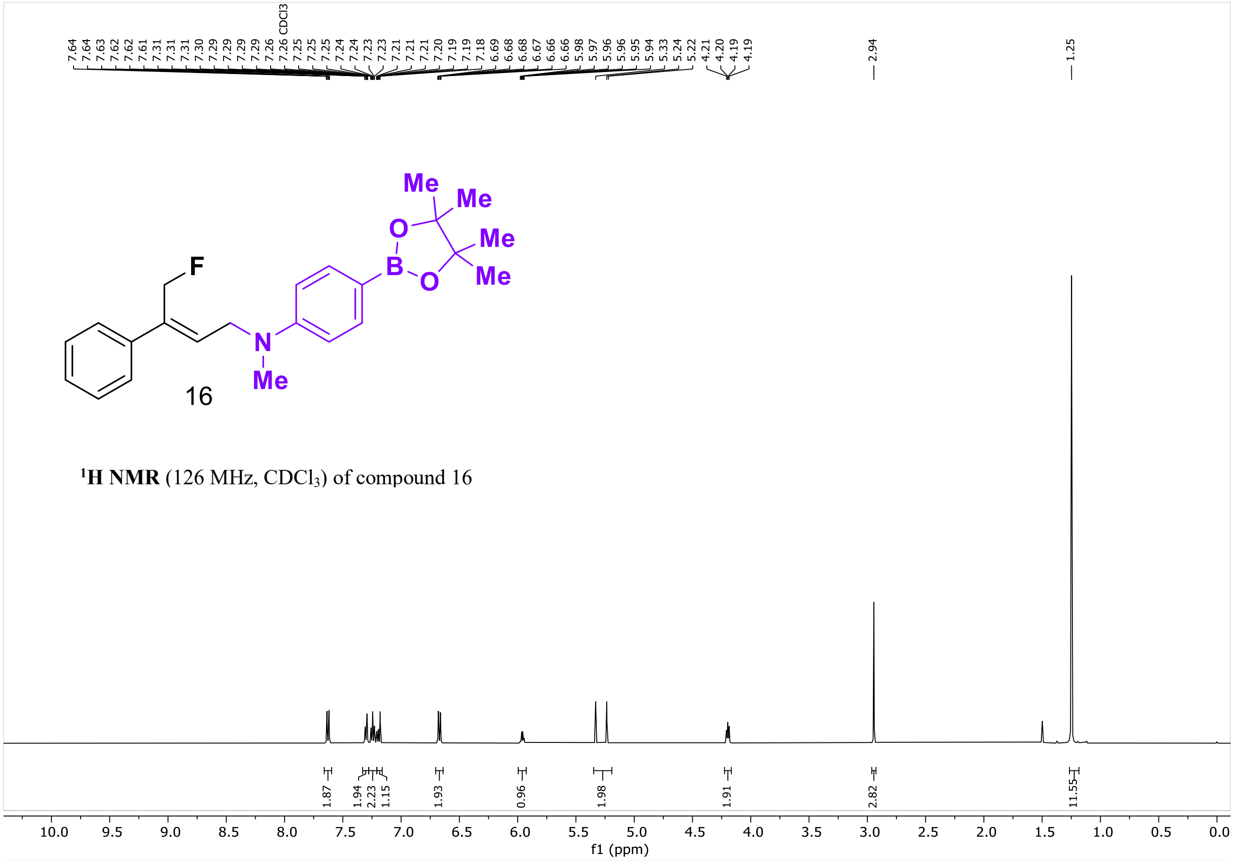

¹H NMR (126 MHz, $CDCl_3$) of compound 16

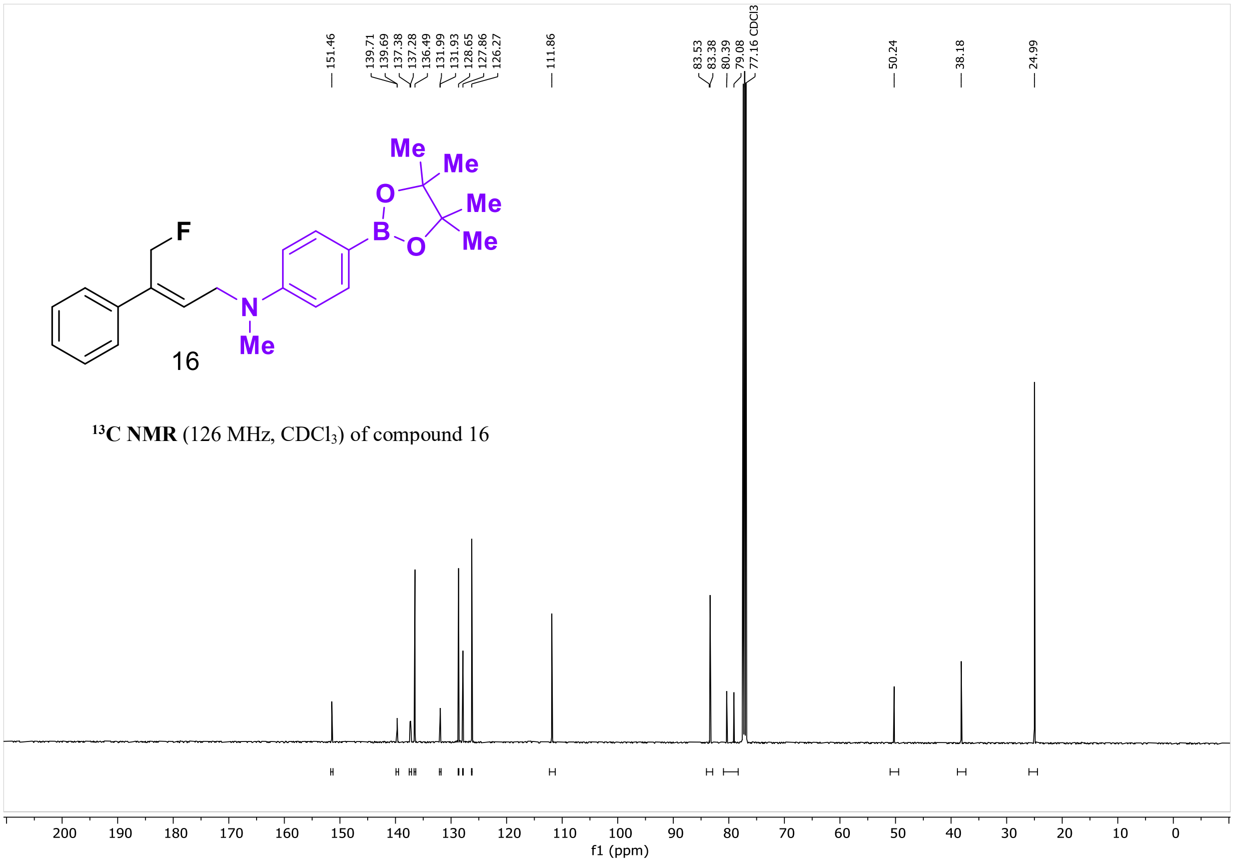

¹³C NMR (126 MHz, $CDCl_3$) of compound 16

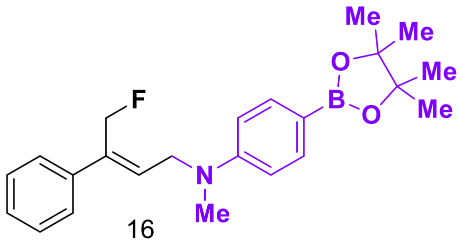

^{19}F NMR (471 MHz, $CDCl_3$) of compound 16

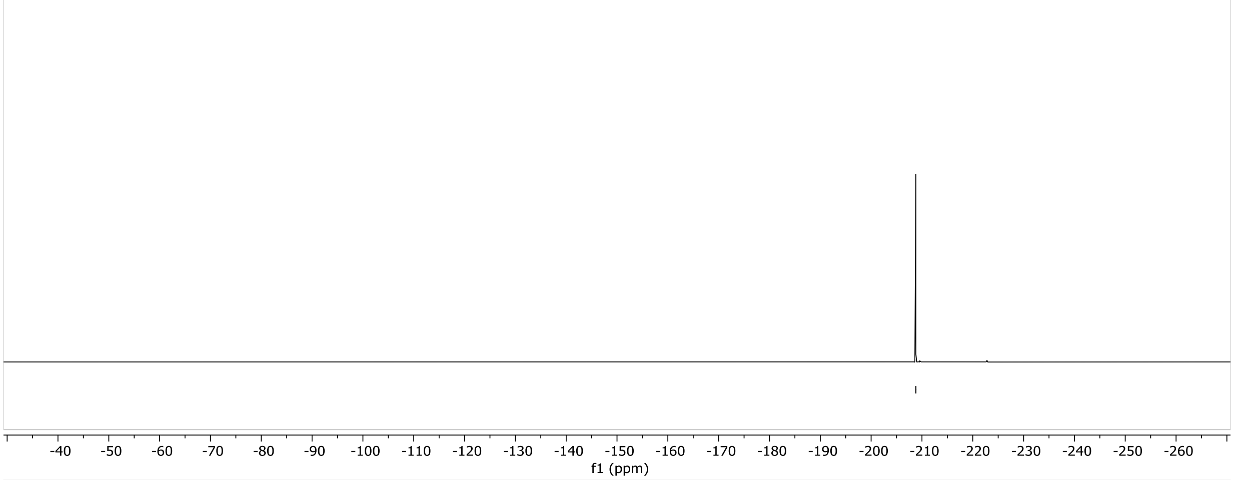

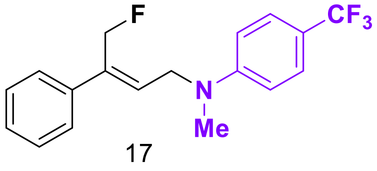

^{1}H NMR (126 MHz, $CDCl_3$) of compound 17

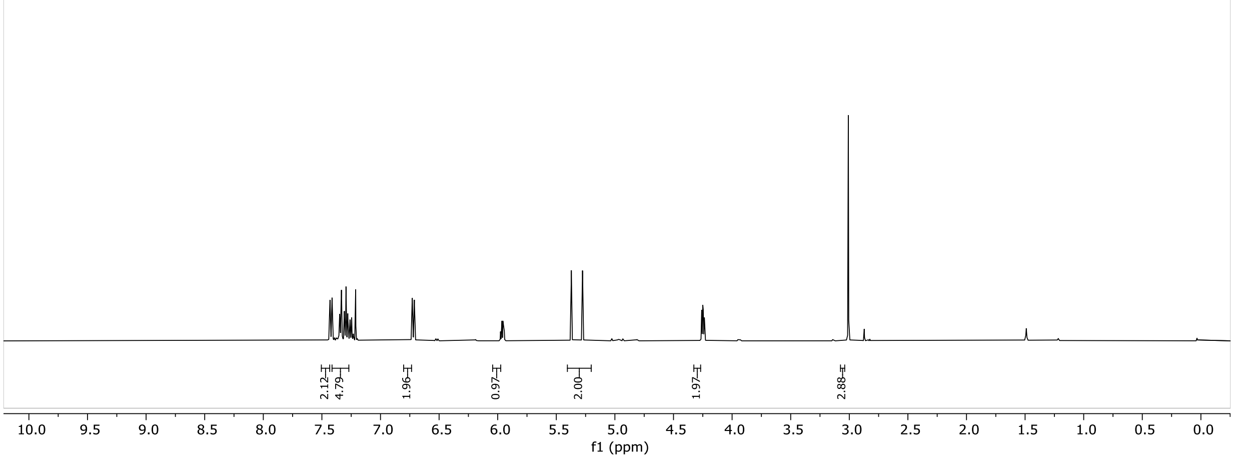

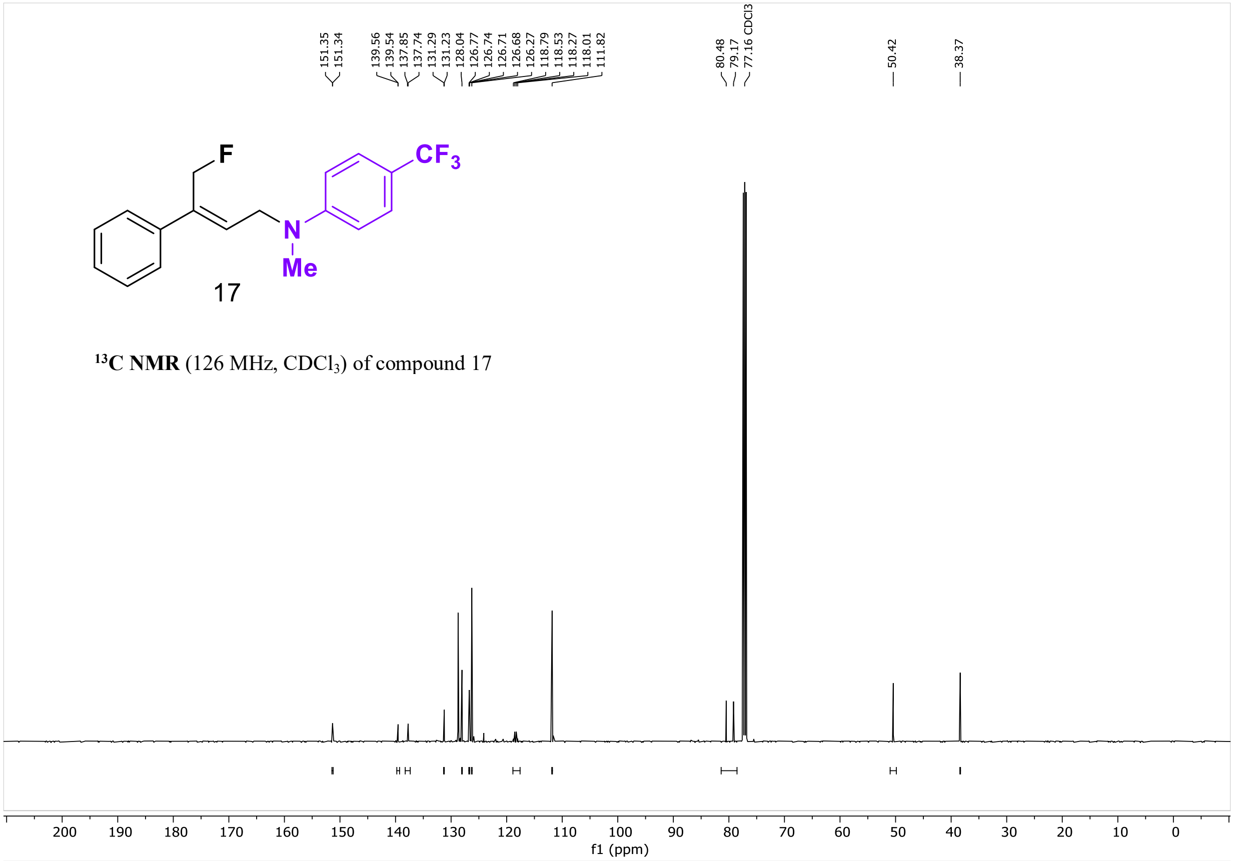

^{13}C **NMR** (126 MHz, $CDCl_3$) of compound 17

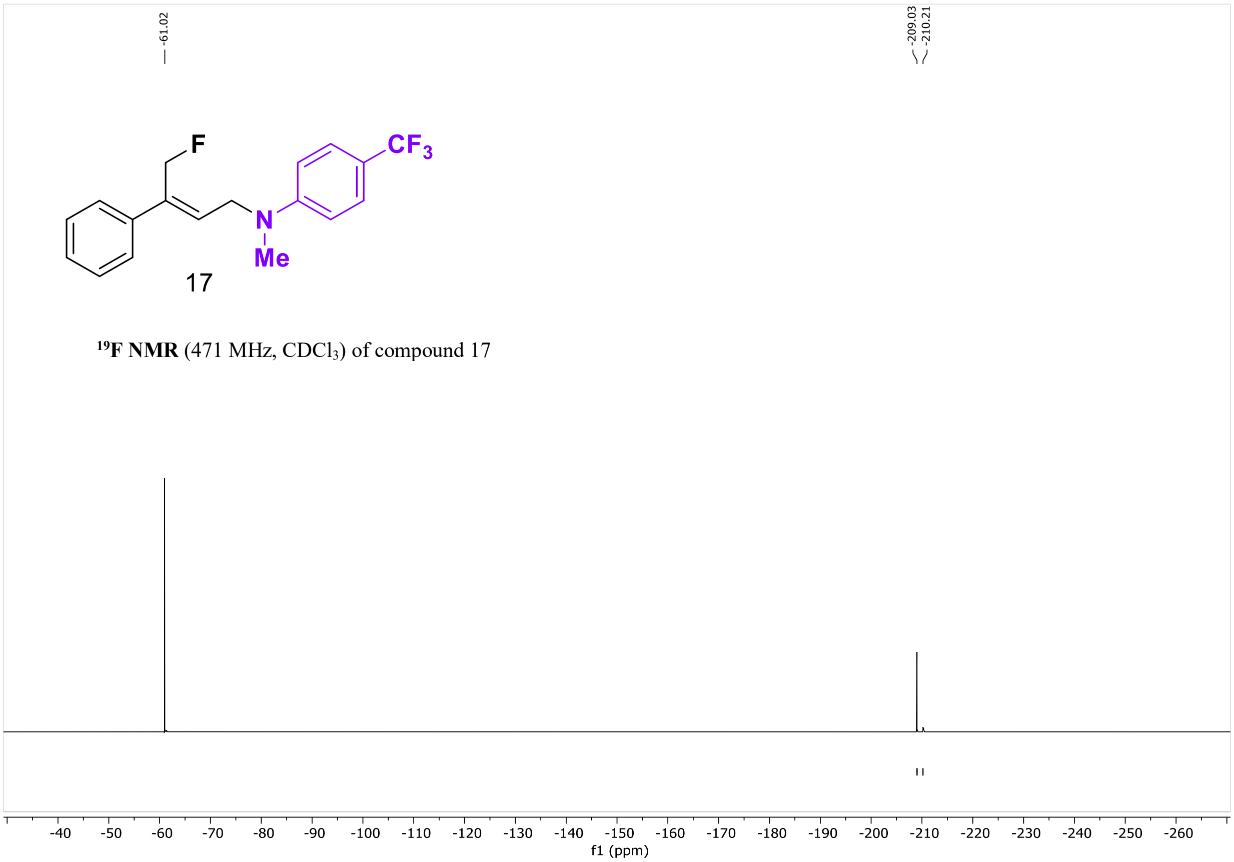

^{19}F **NMR** (471 MHz, $CDCl_3$) of compound 17

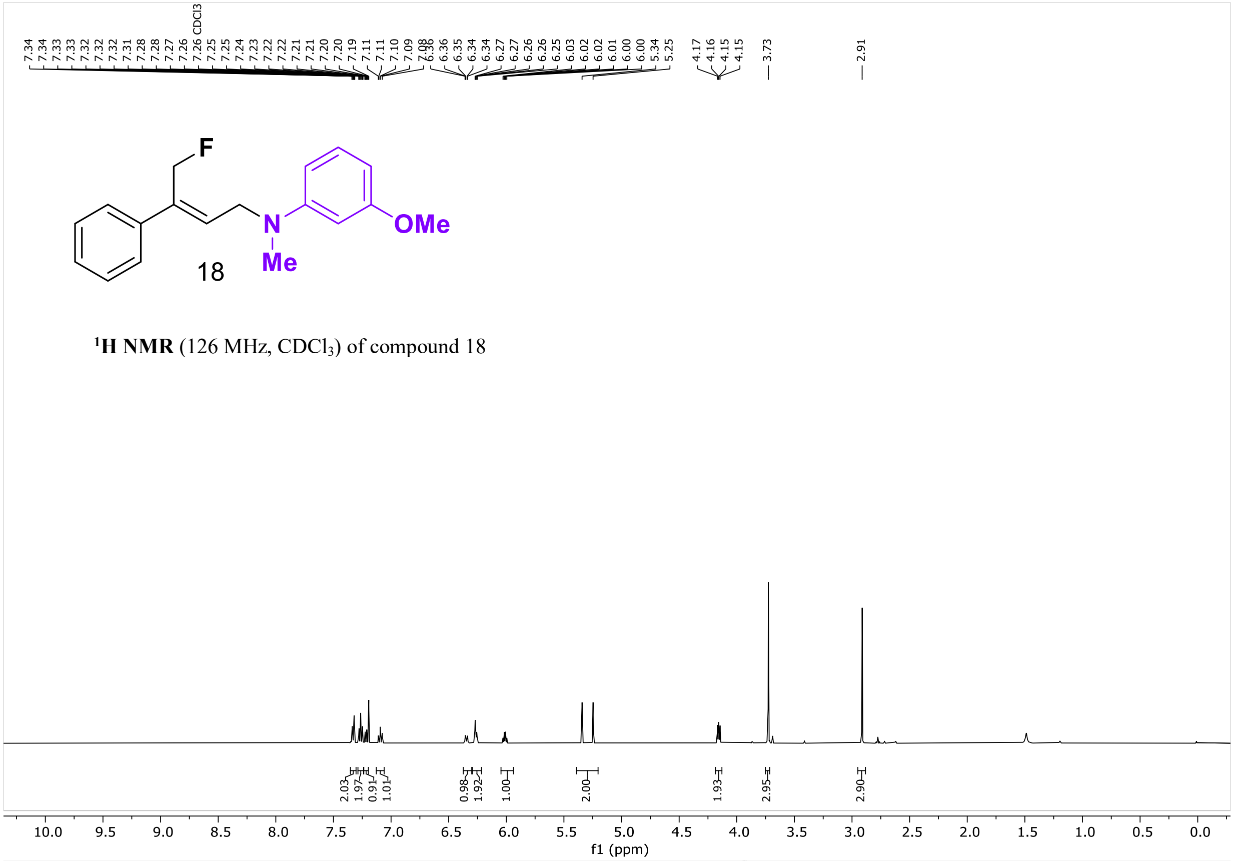

^{1}**H NMR** (126 MHz, $CDCl_3$) of compound 18

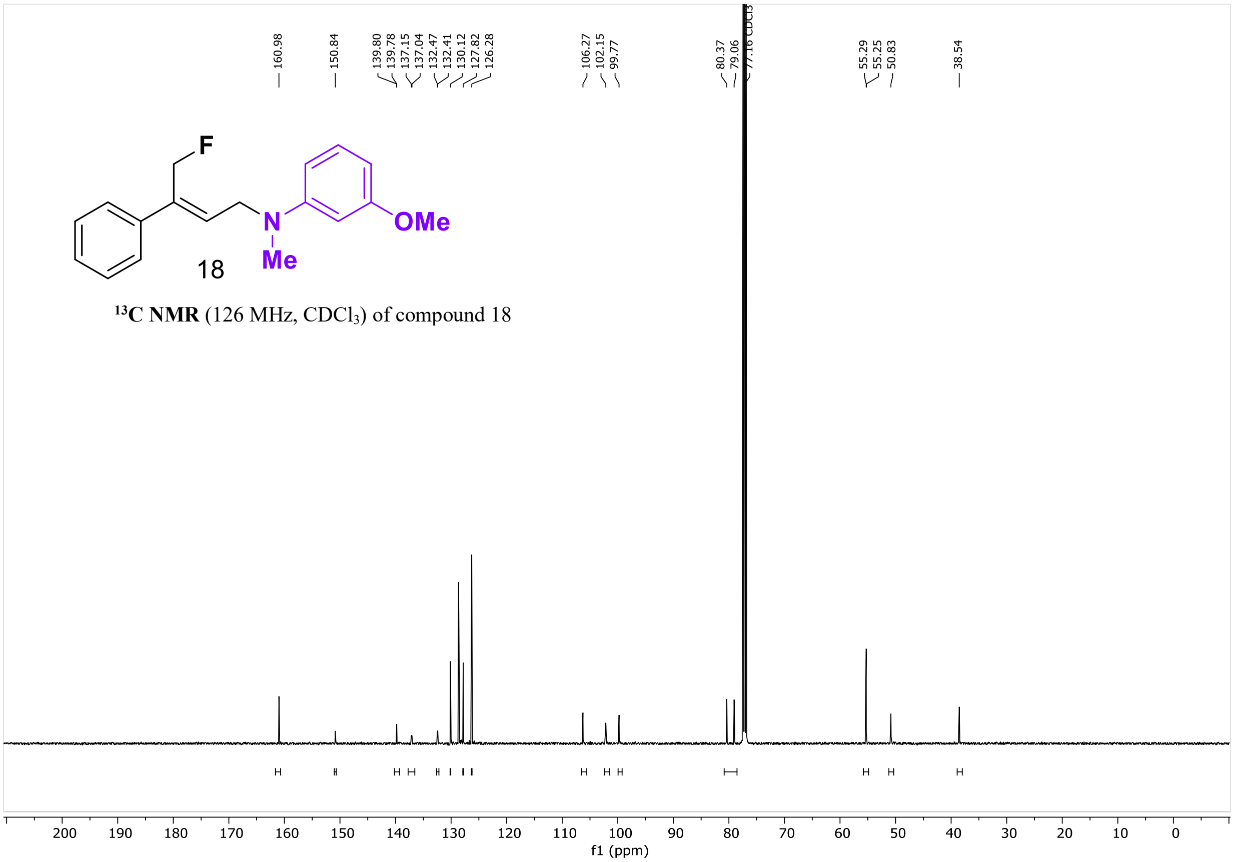

^{13}**C NMR** (126 MHz, $CDCl_3$) of compound 18

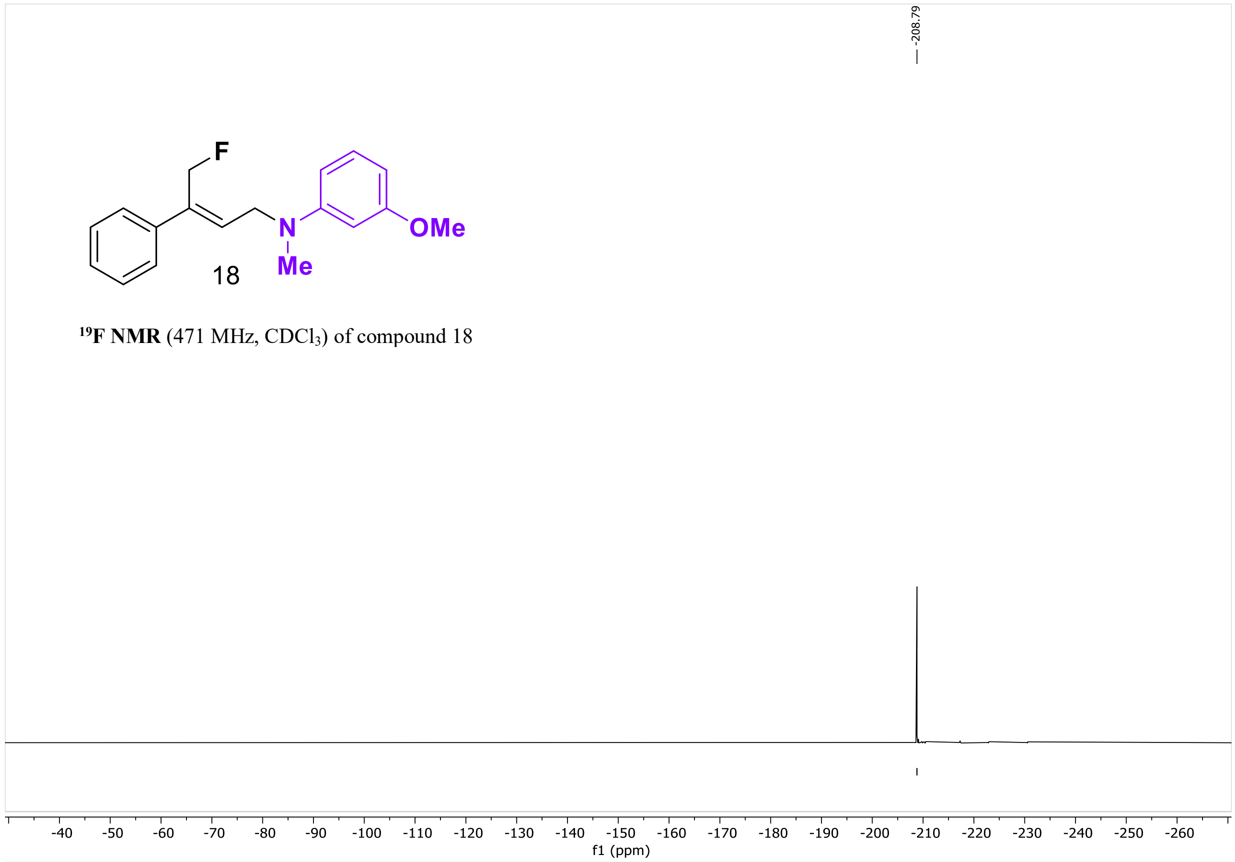

^{19}F NMR (471 MHz, $CDCl_3$) of compound 18

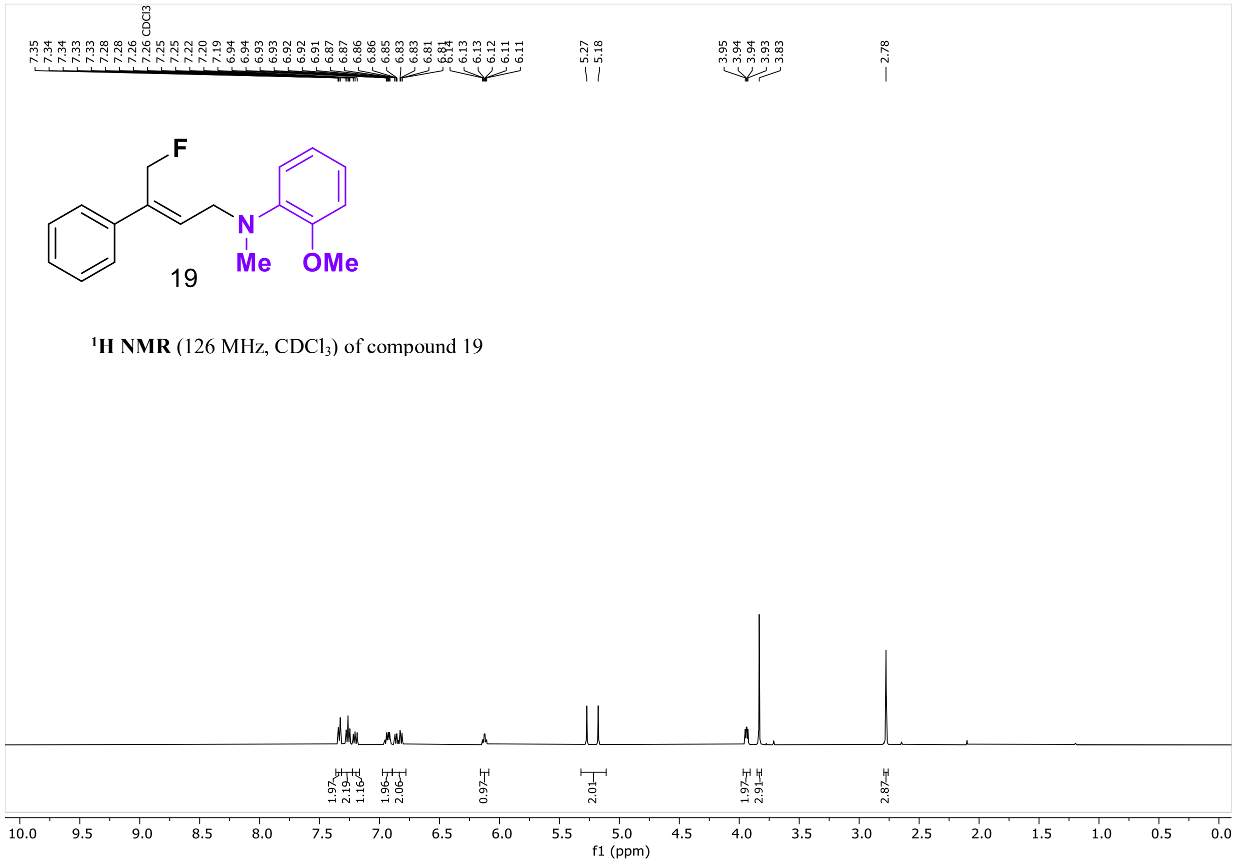

^{1}H NMR (126 MHz, $CDCl_3$) of compound 19

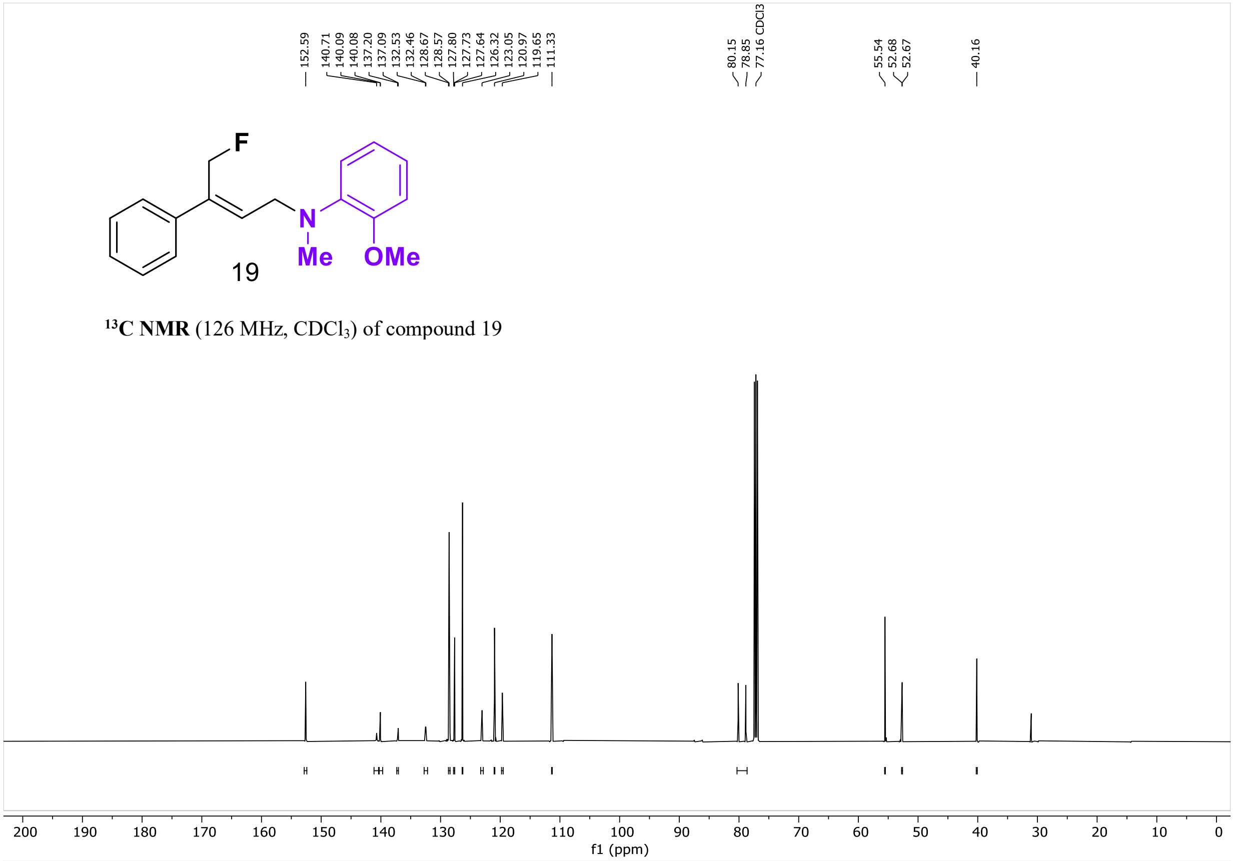

^{13}**C NMR** (126 MHz, $CDCl_3$) of compound 19

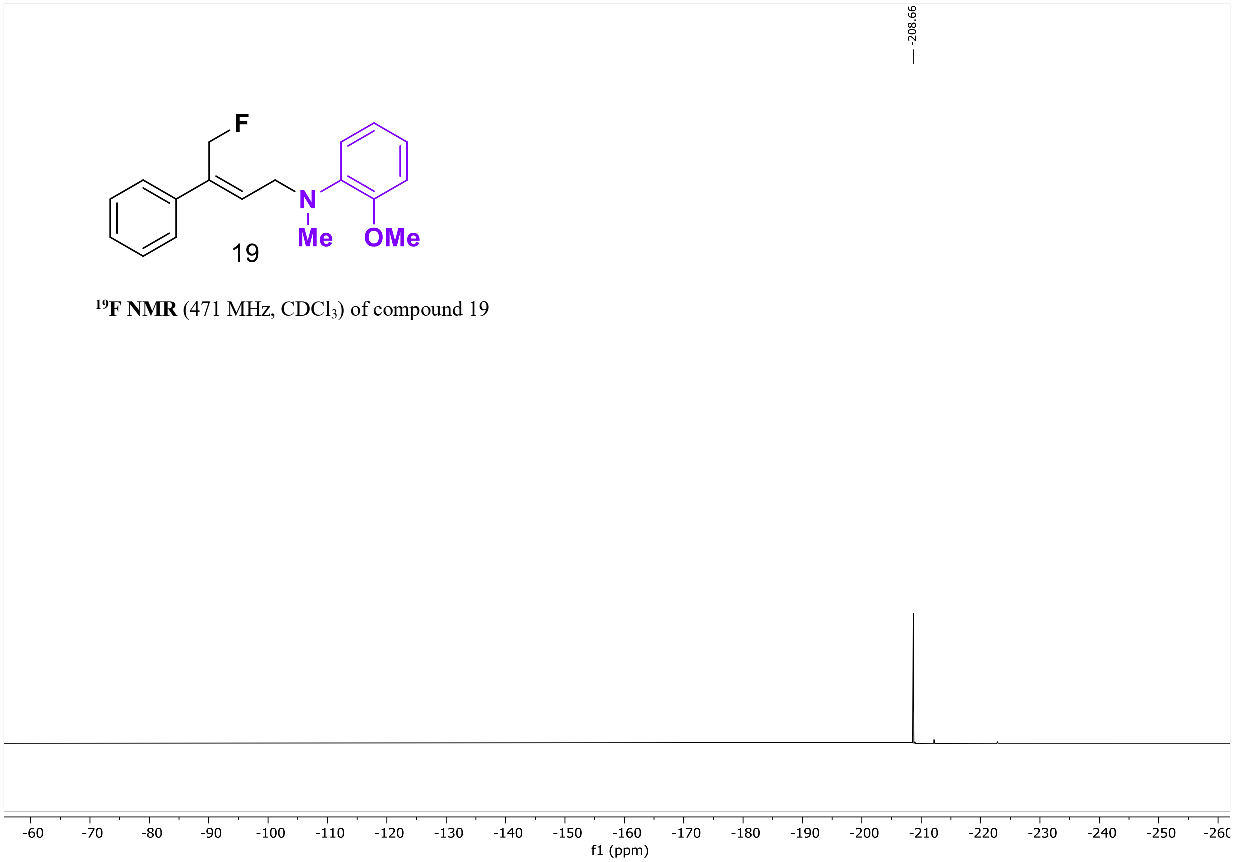

^{19}**F NMR** (471 MHz, $CDCl_3$) of compound 19

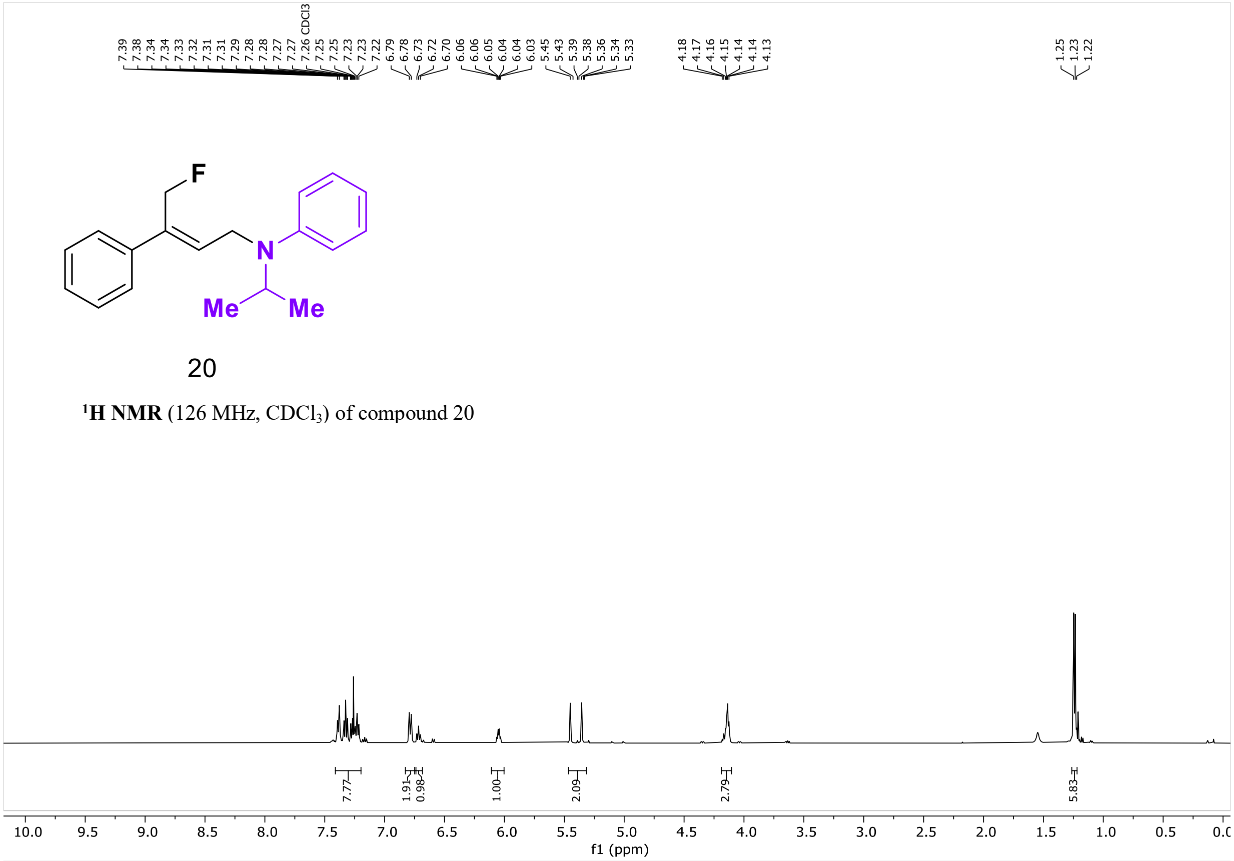

^{1}H NMR (126 MHz, $CDCl_3$) of compound 20

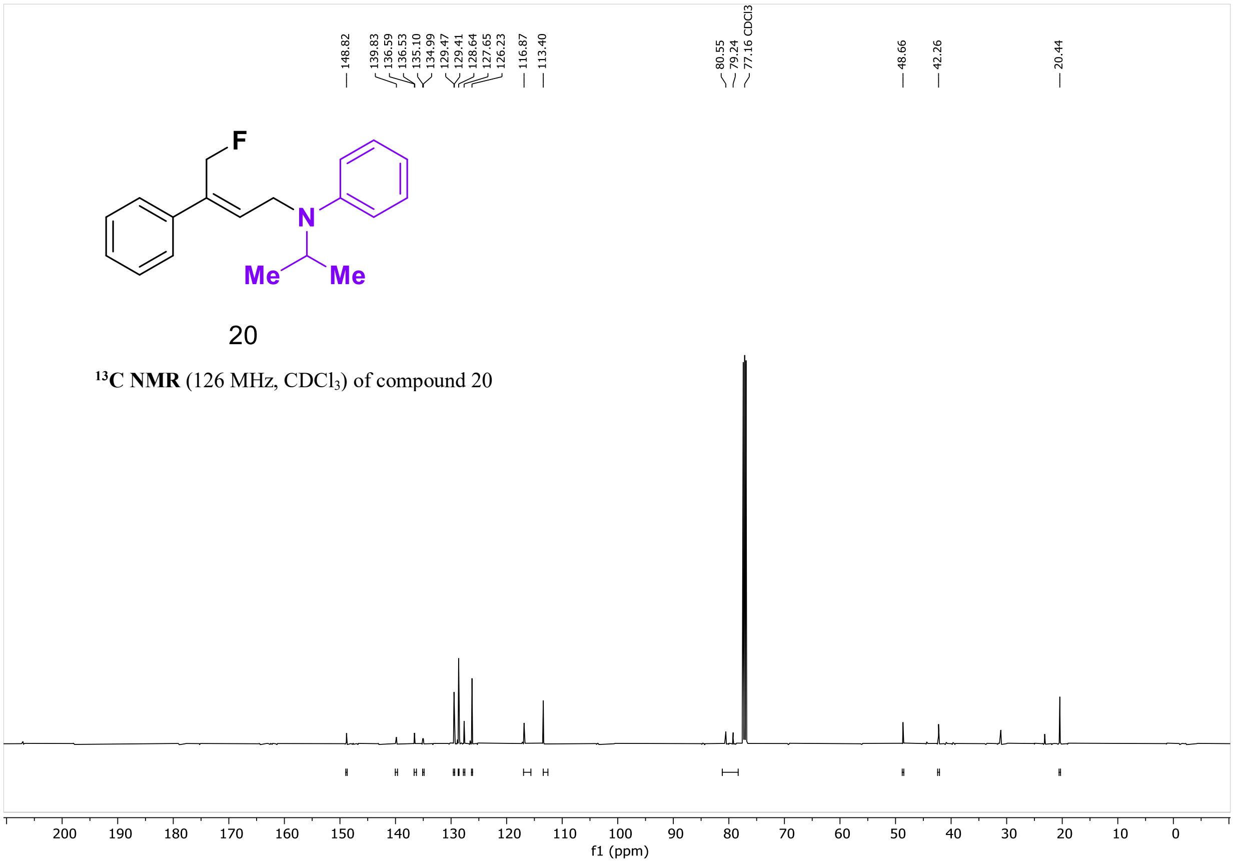

^{13}C NMR (126 MHz, $CDCl_3$) of compound 20

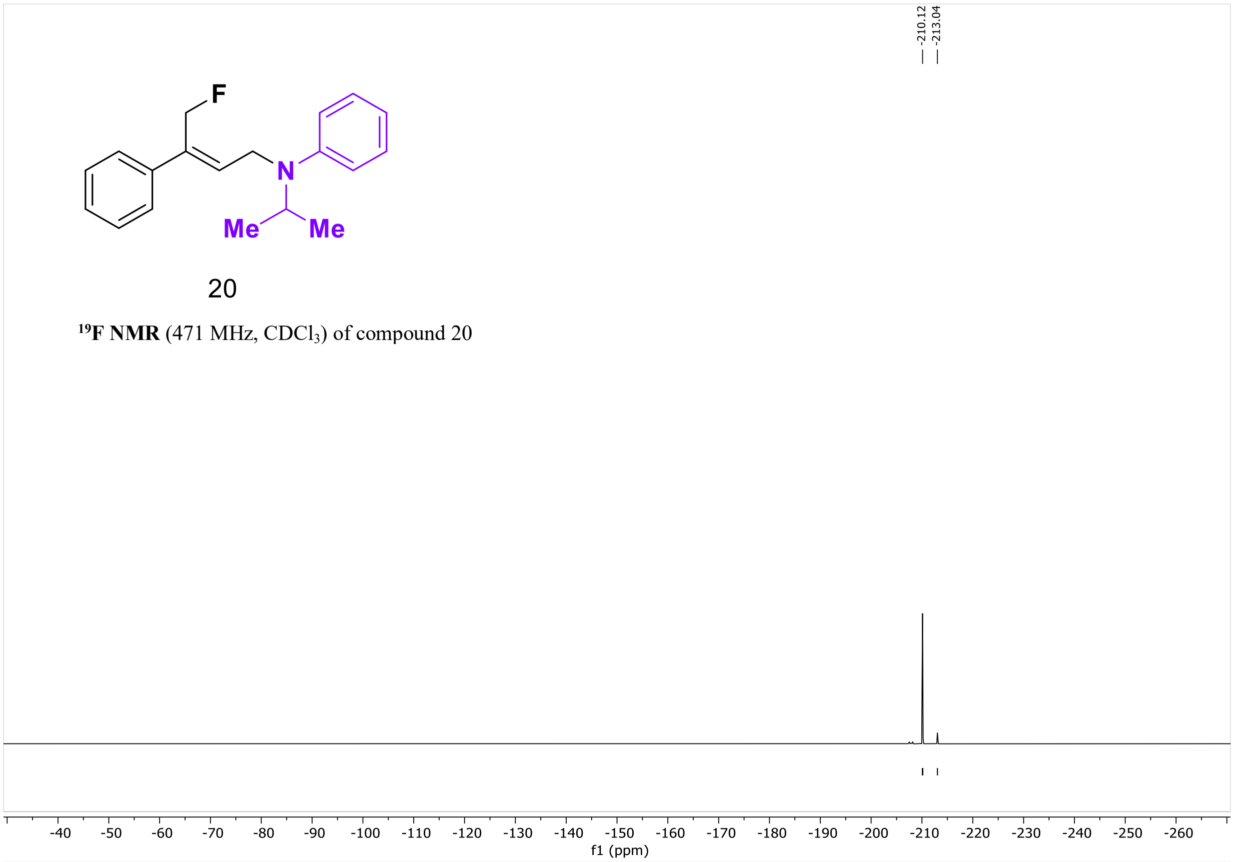

^{19}F NMR (471 MHz, $CDCl_3$) of compound 20

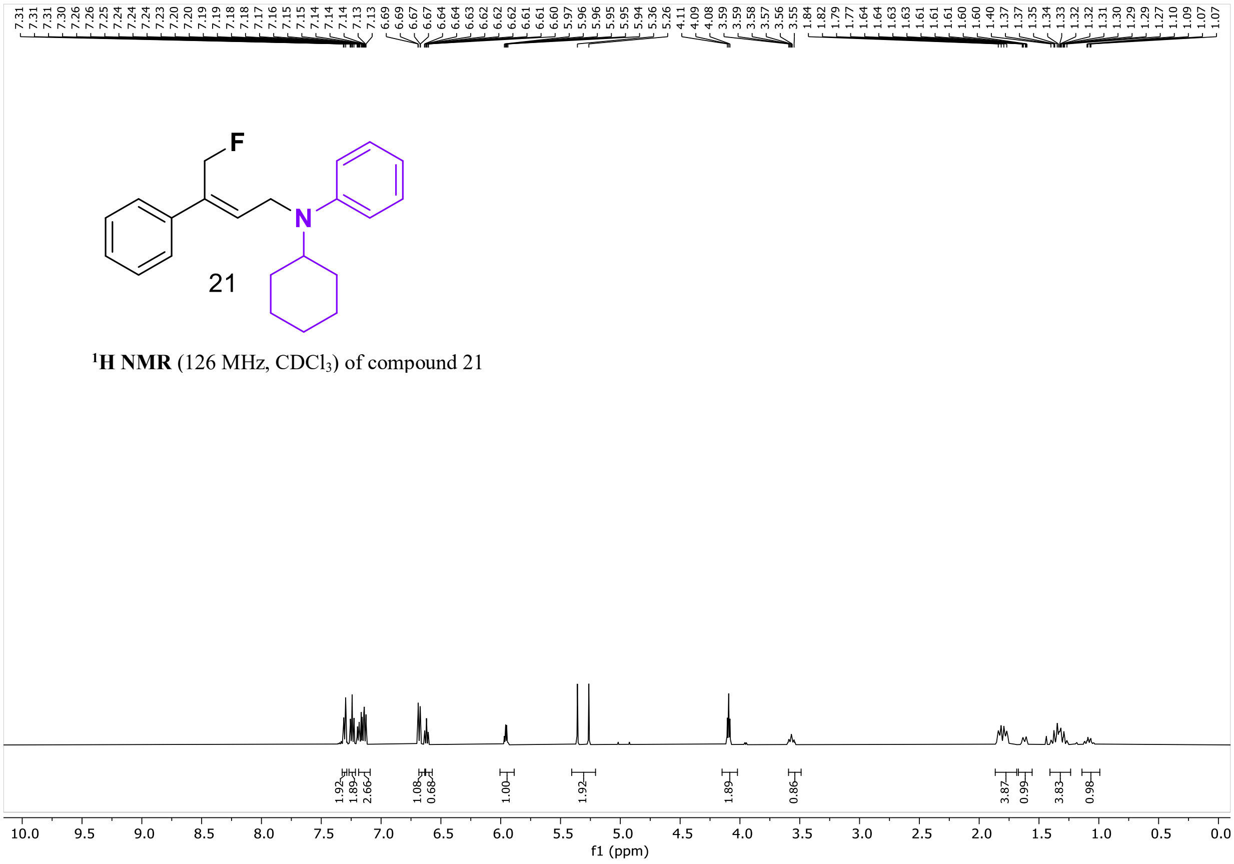

^{1}H NMR (126 MHz, $CDCl_3$) of compound 21

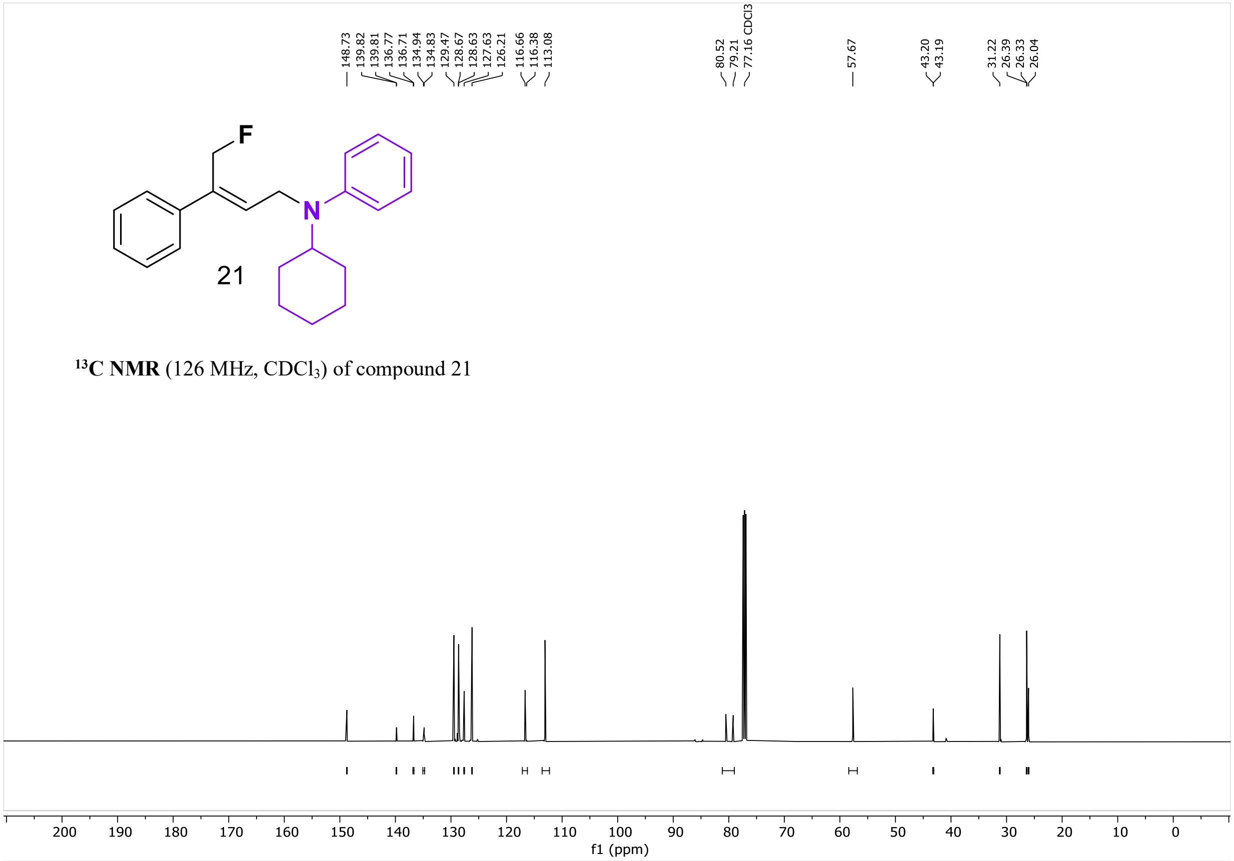

^{13}C NMR (126 MHz, $CDCl_3$) of compound 21

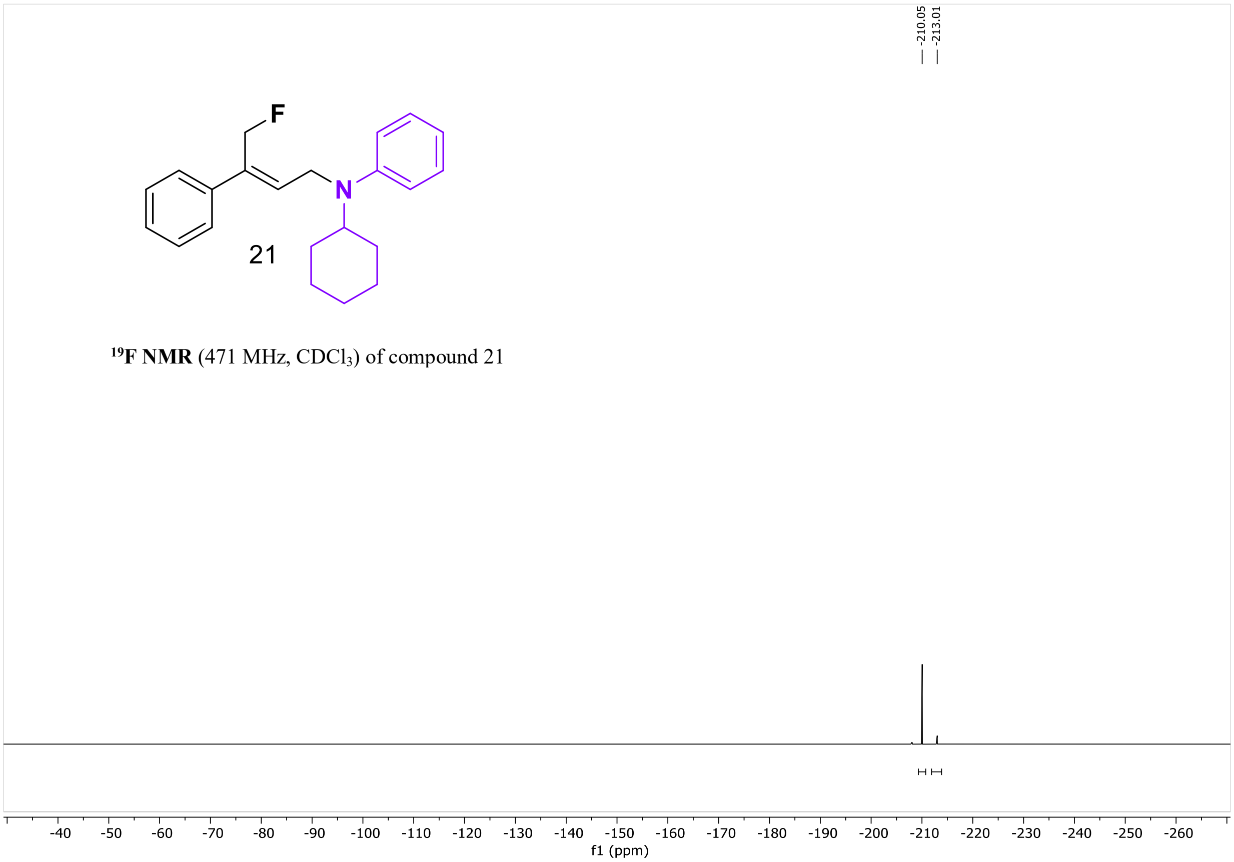

^{19}F NMR (471 MHz, $CDCl_3$) of compound 21

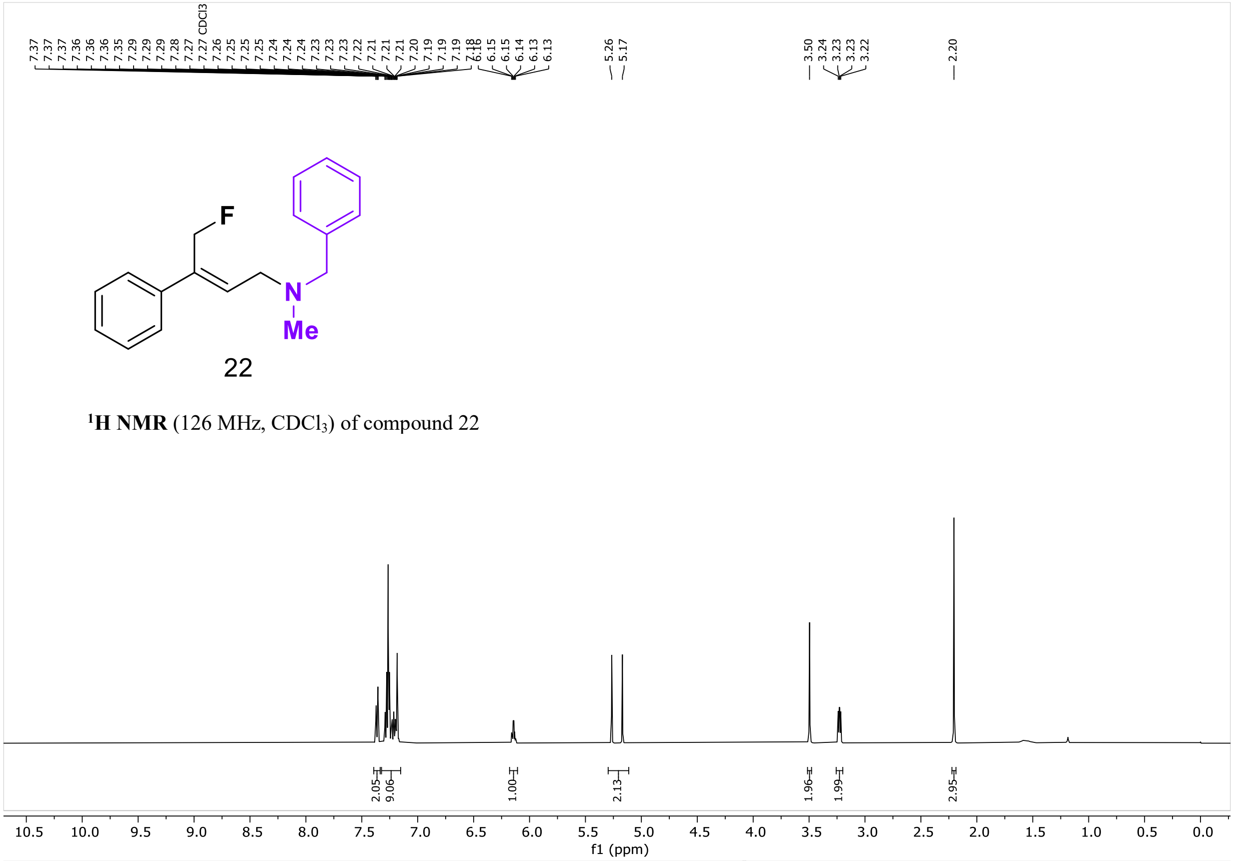

^{1}H NMR (126 MHz, $CDCl_3$) of compound 22

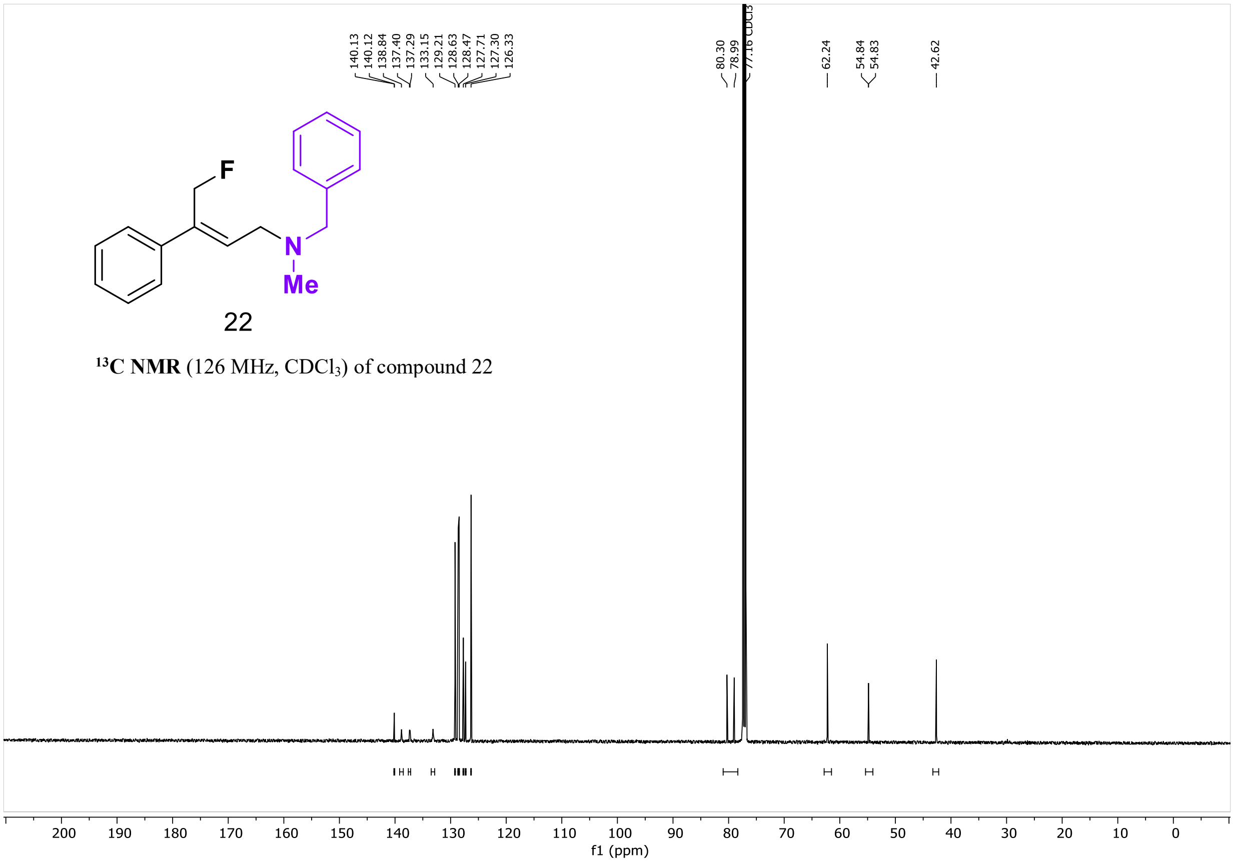

^{13}C NMR (126 MHz, $CDCl_3$) of compound 22

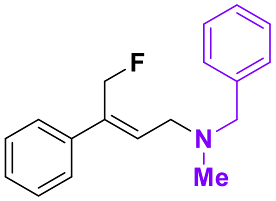

22

^{19}F NMR (471 MHz, $CDCl_3$) of compound 22

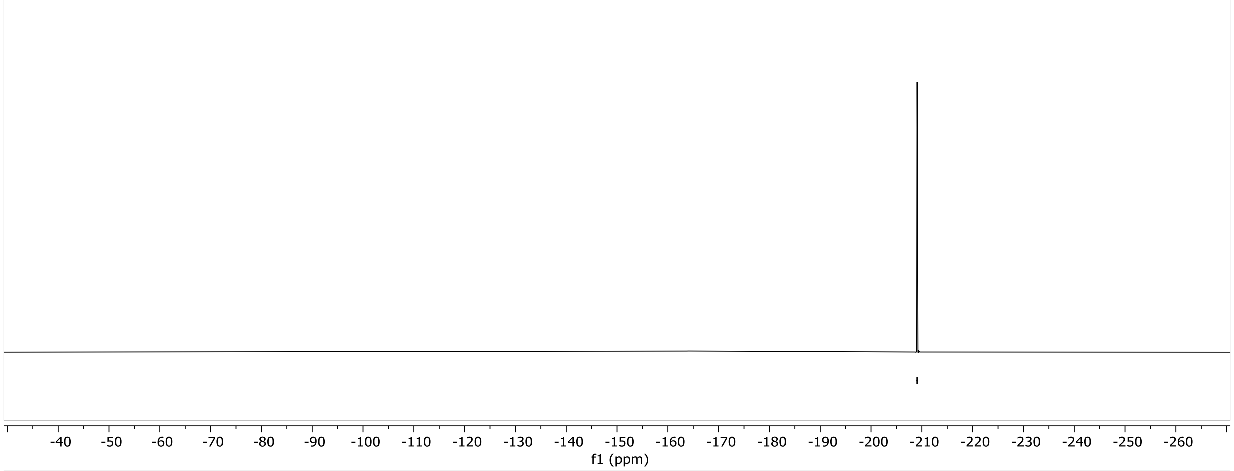

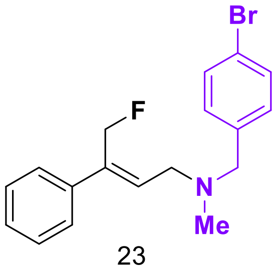

23

^{1}H NMR (126 MHz, $CDCl_3$) of compound 23

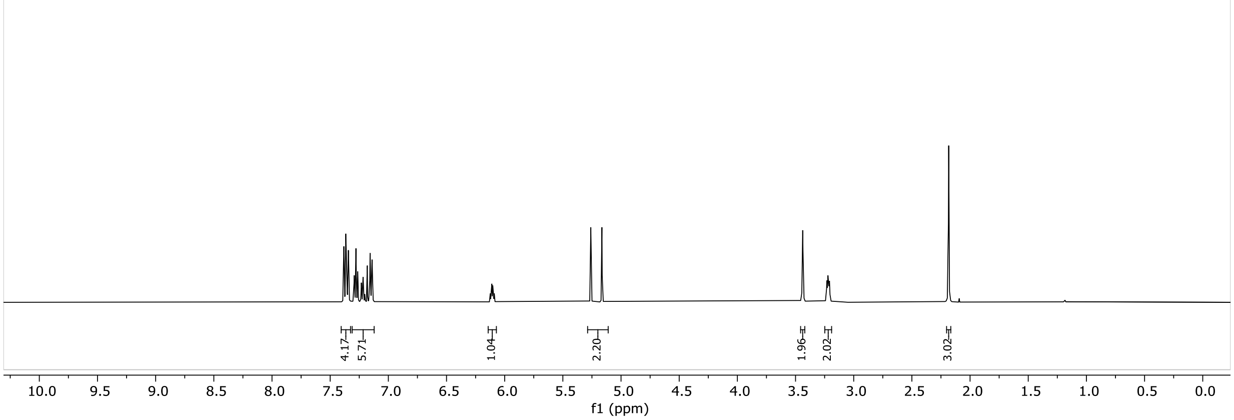

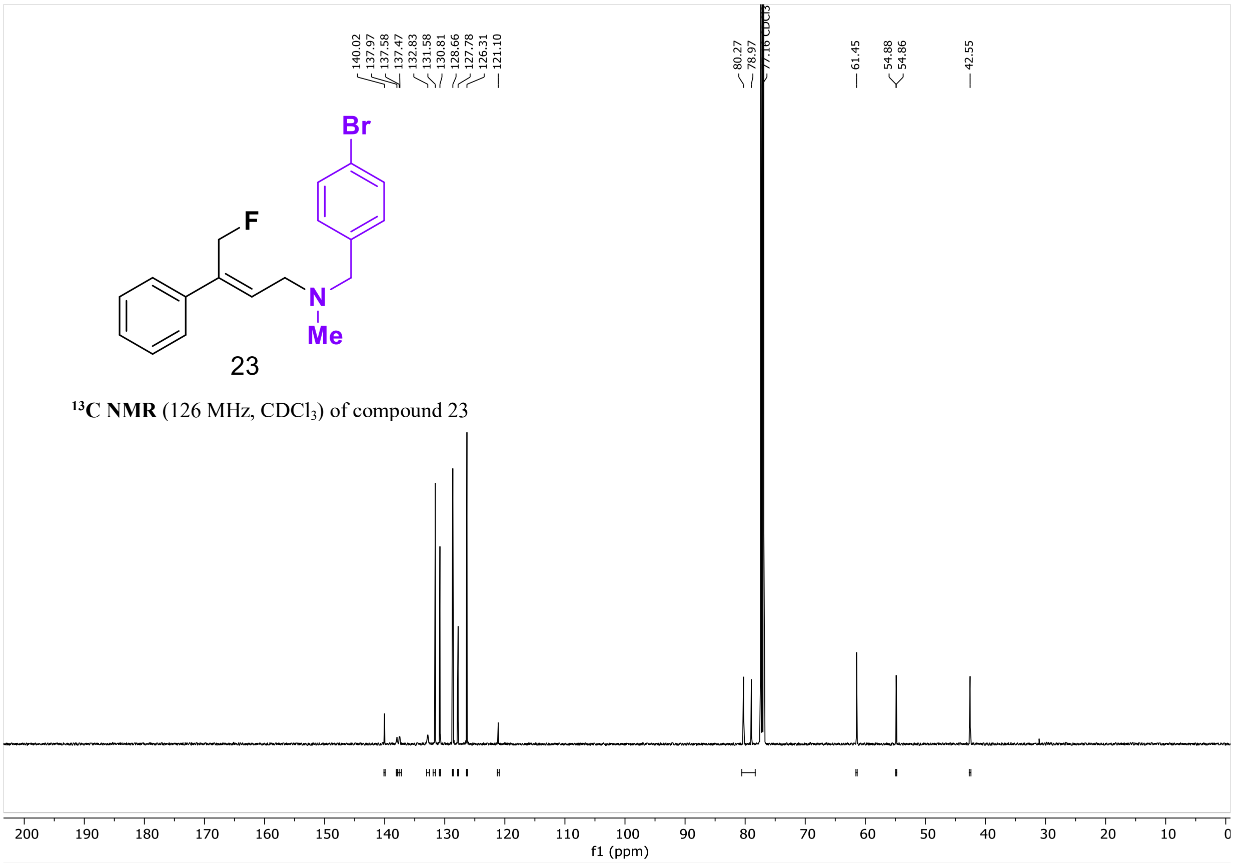

^{13}**C NMR** (126 MHz, $CDCl_3$) of compound 23

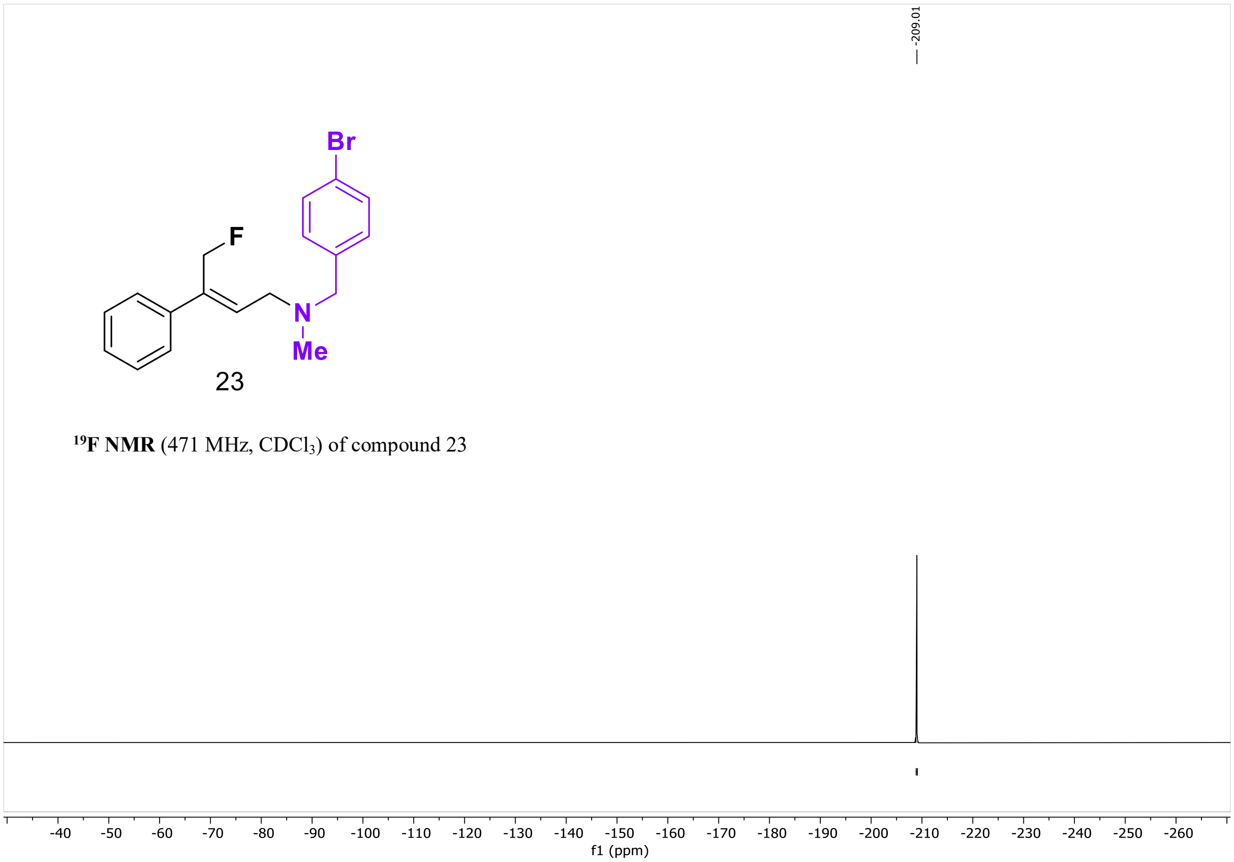

^{19}**F NMR** (471 MHz, $CDCl_3$) of compound 23

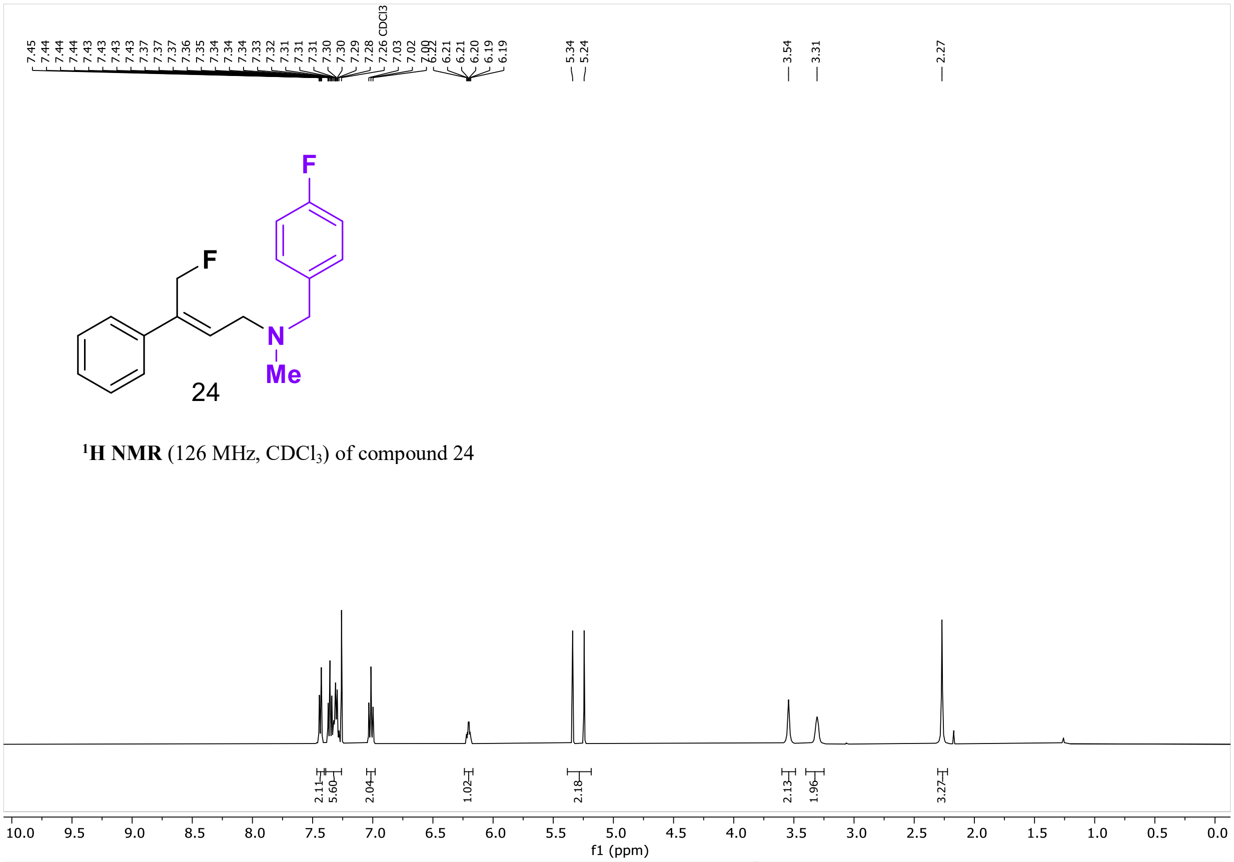

¹H NMR (126 MHz, $CDCl_3$) of compound 24

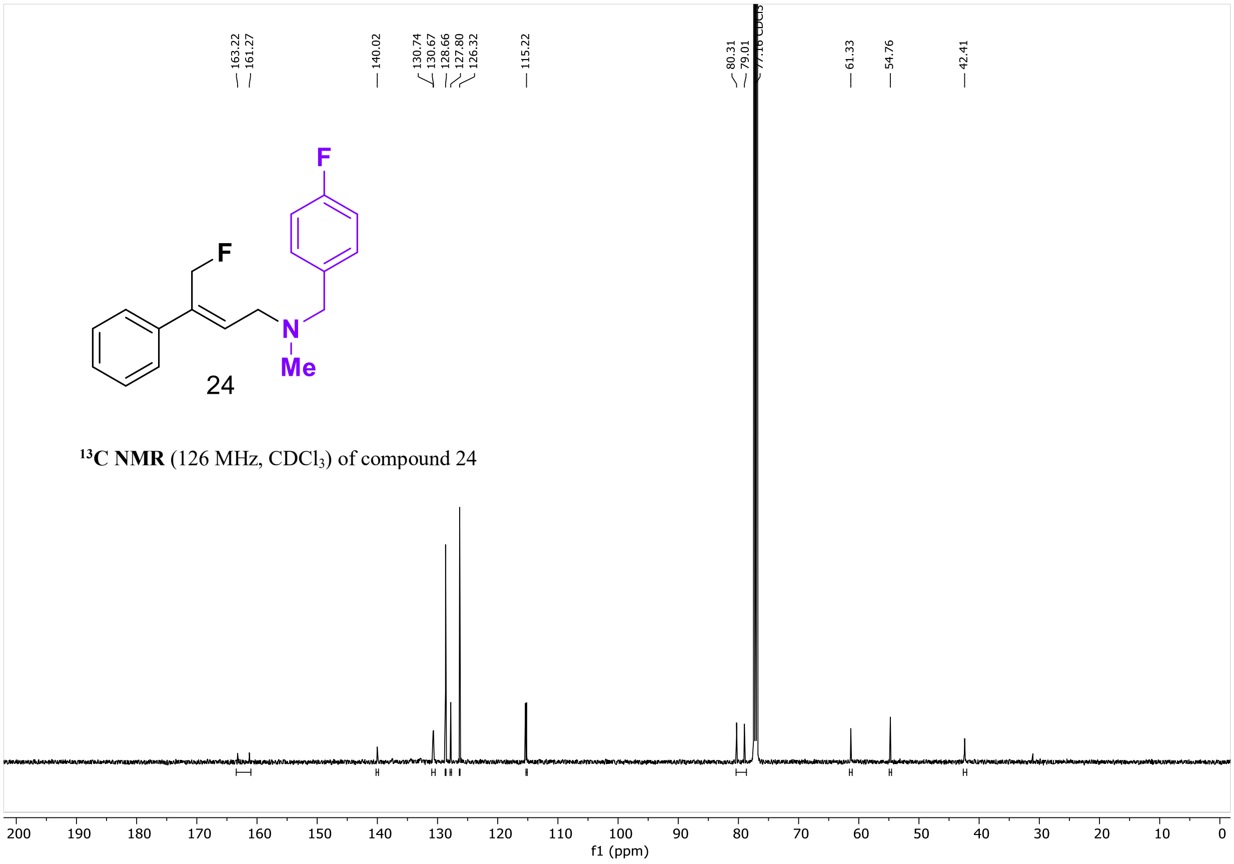

¹³C NMR (126 MHz, $CDCl_3$) of compound 24

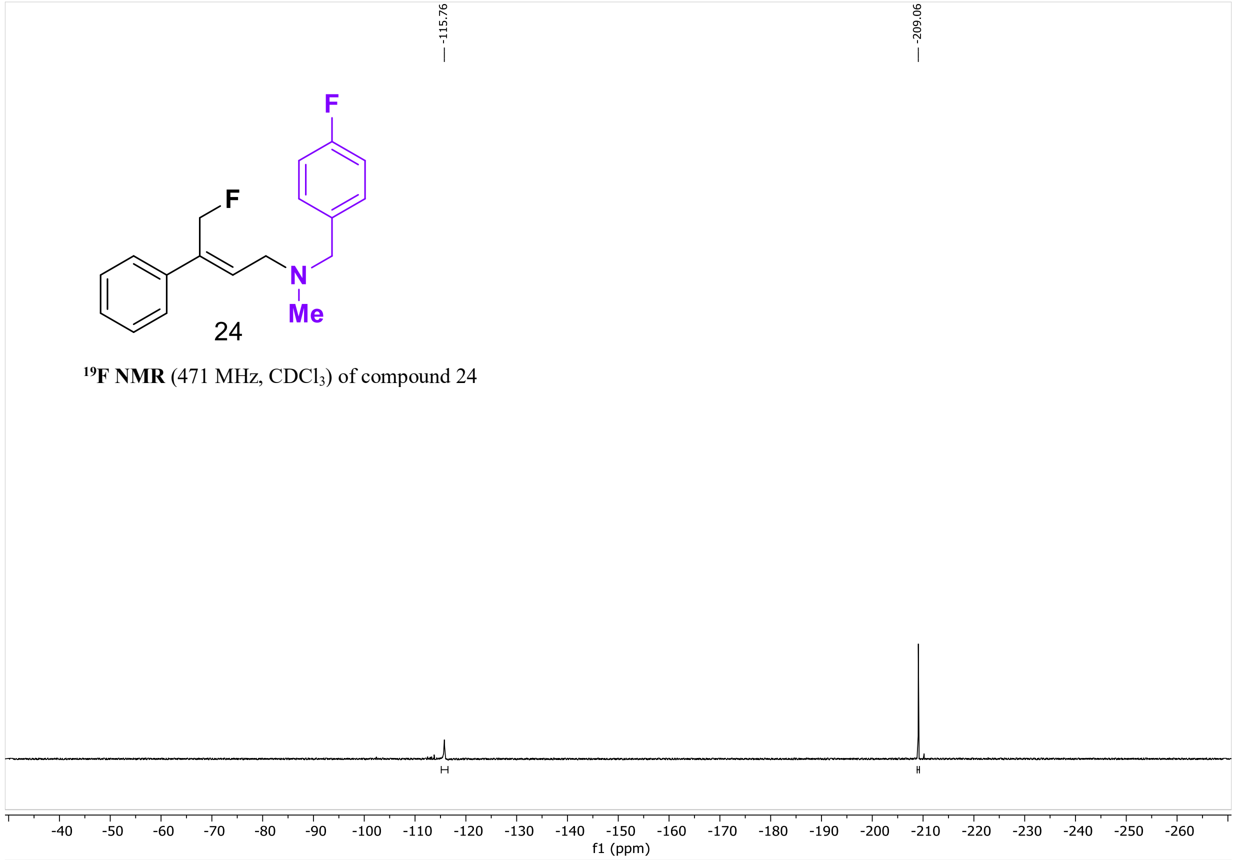

^{19}F NMR (471 MHz, $CDCl_3$) of compound 24

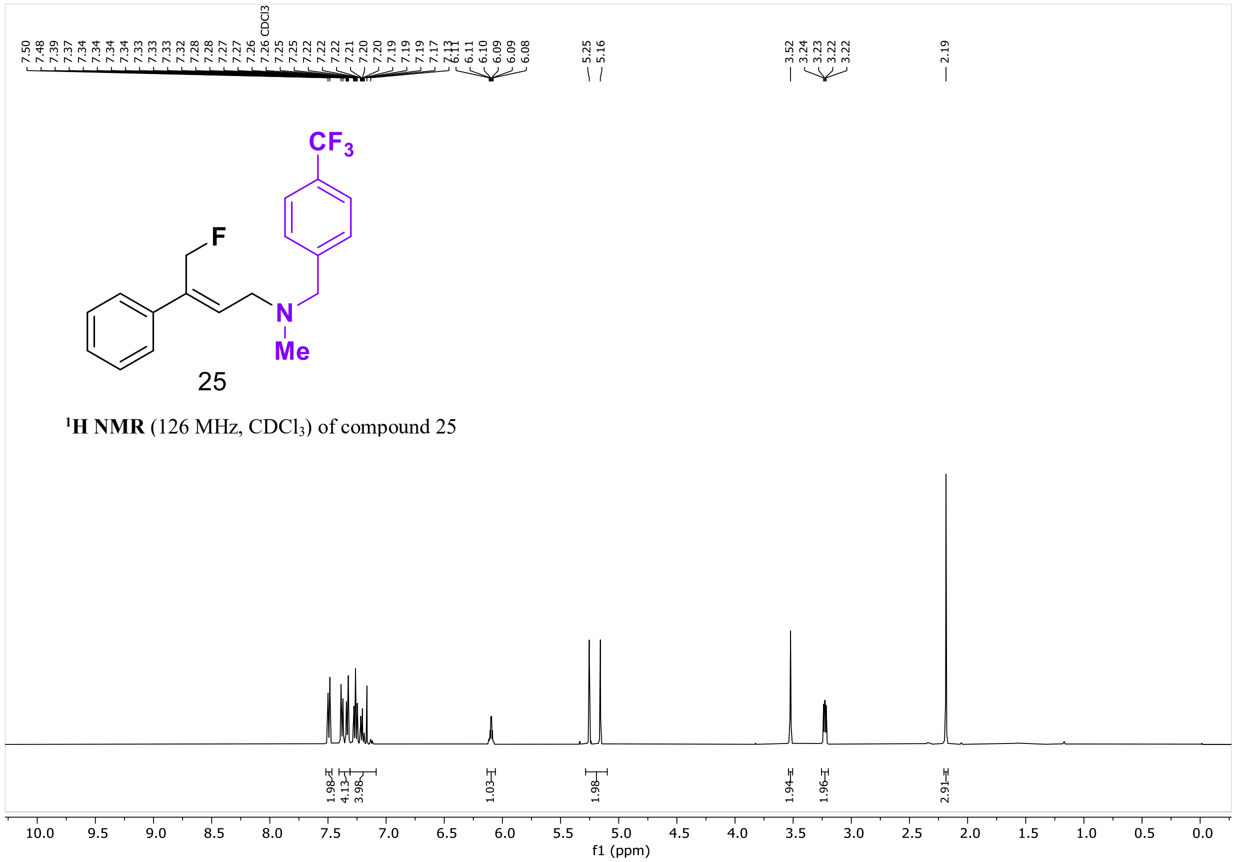

^{1}H NMR (126 MHz, $CDCl_3$) of compound 25

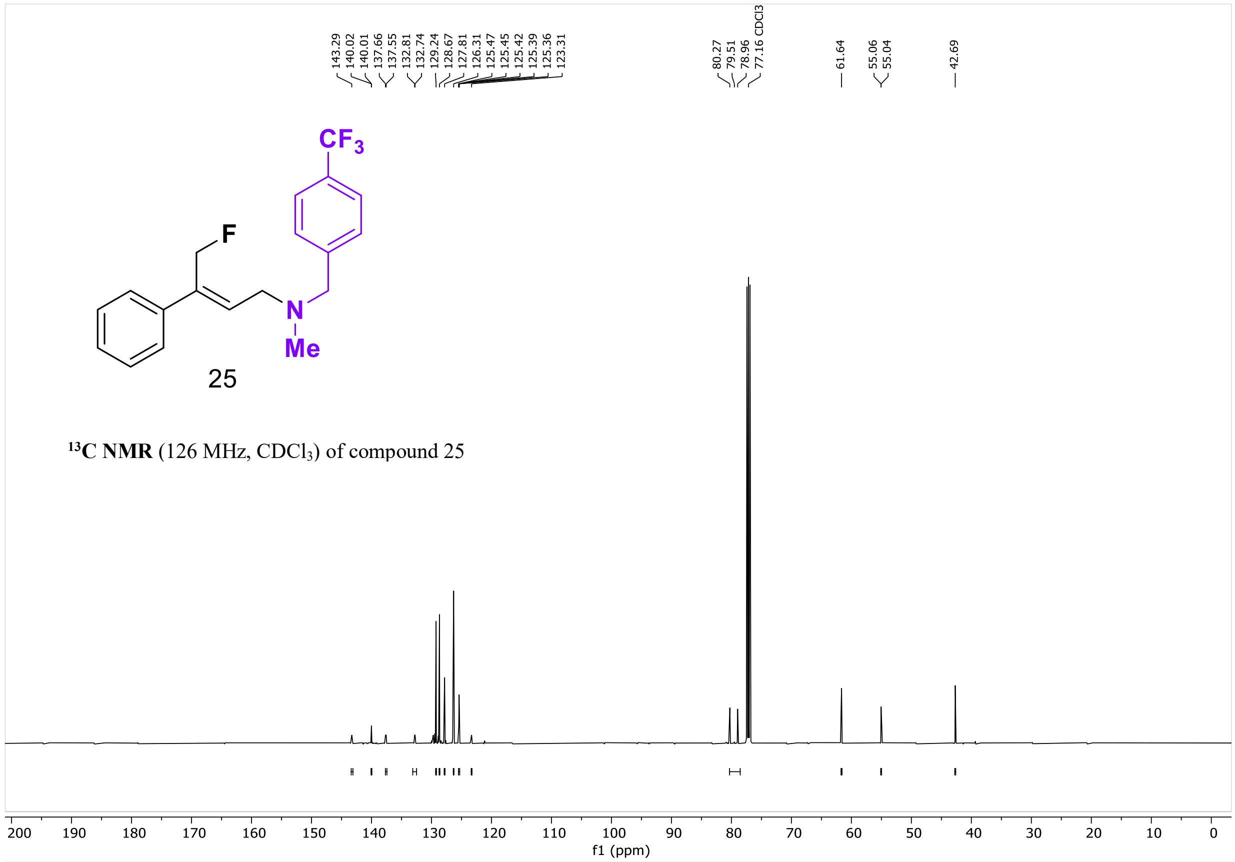

^{13}C NMR (126 MHz, $CDCl_3$) of compound 25

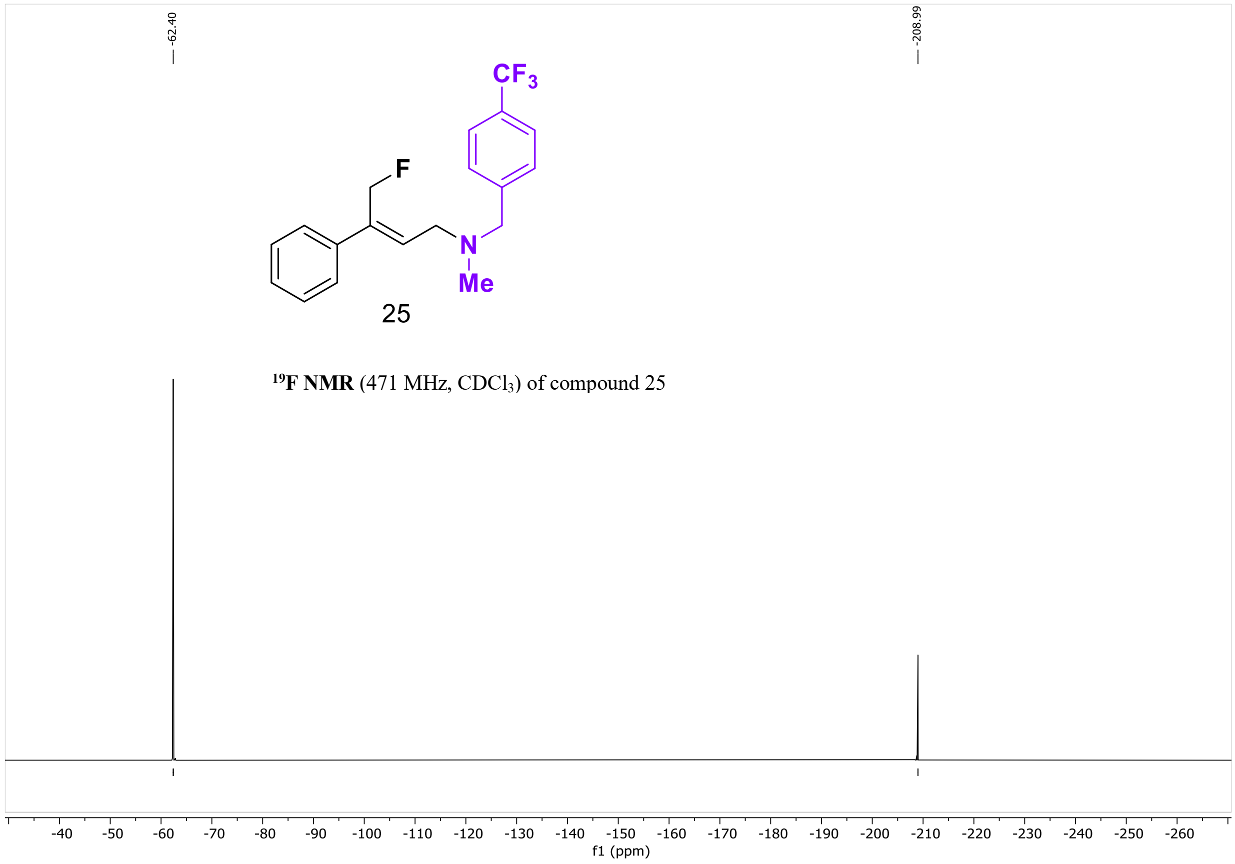

^{19}F NMR (471 MHz, $CDCl_3$) of compound 25

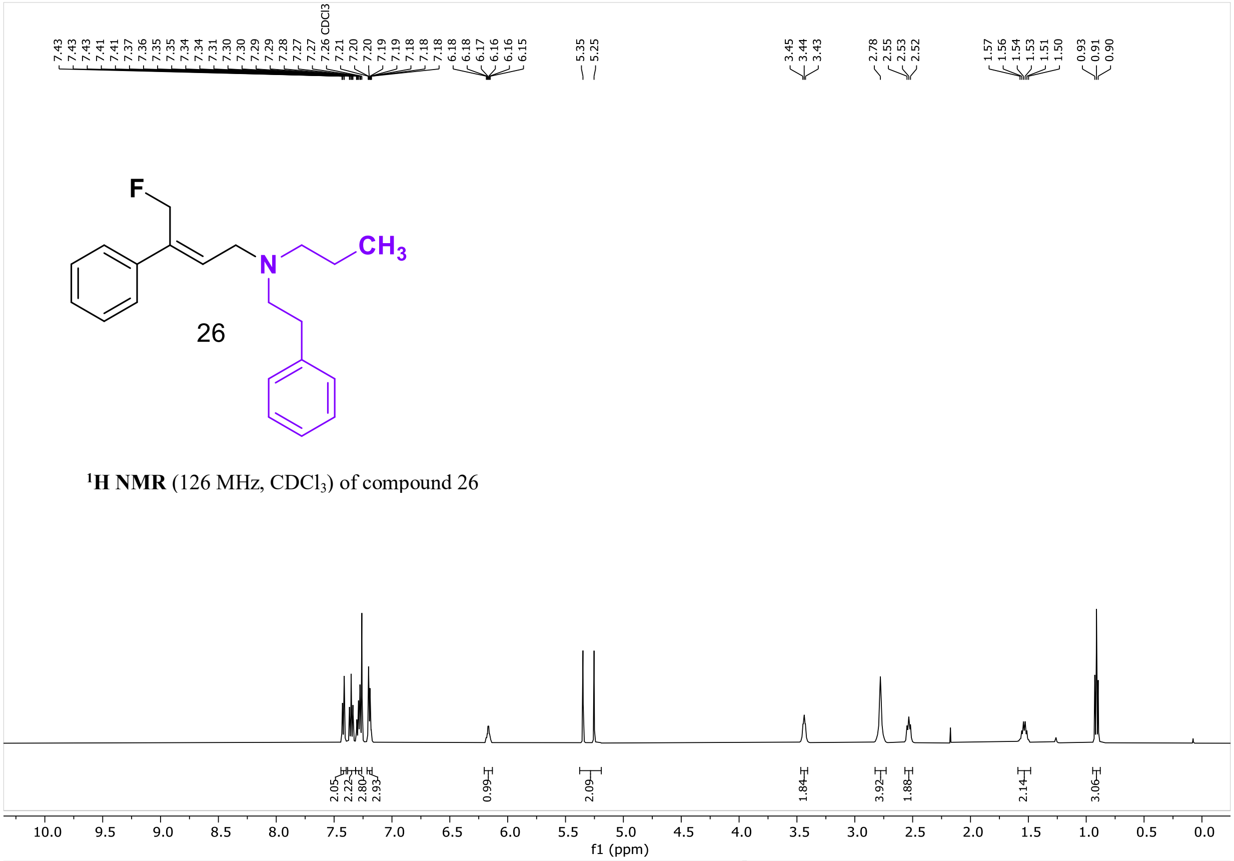

¹H NMR (126 MHz, $CDCl_3$) of compound 26

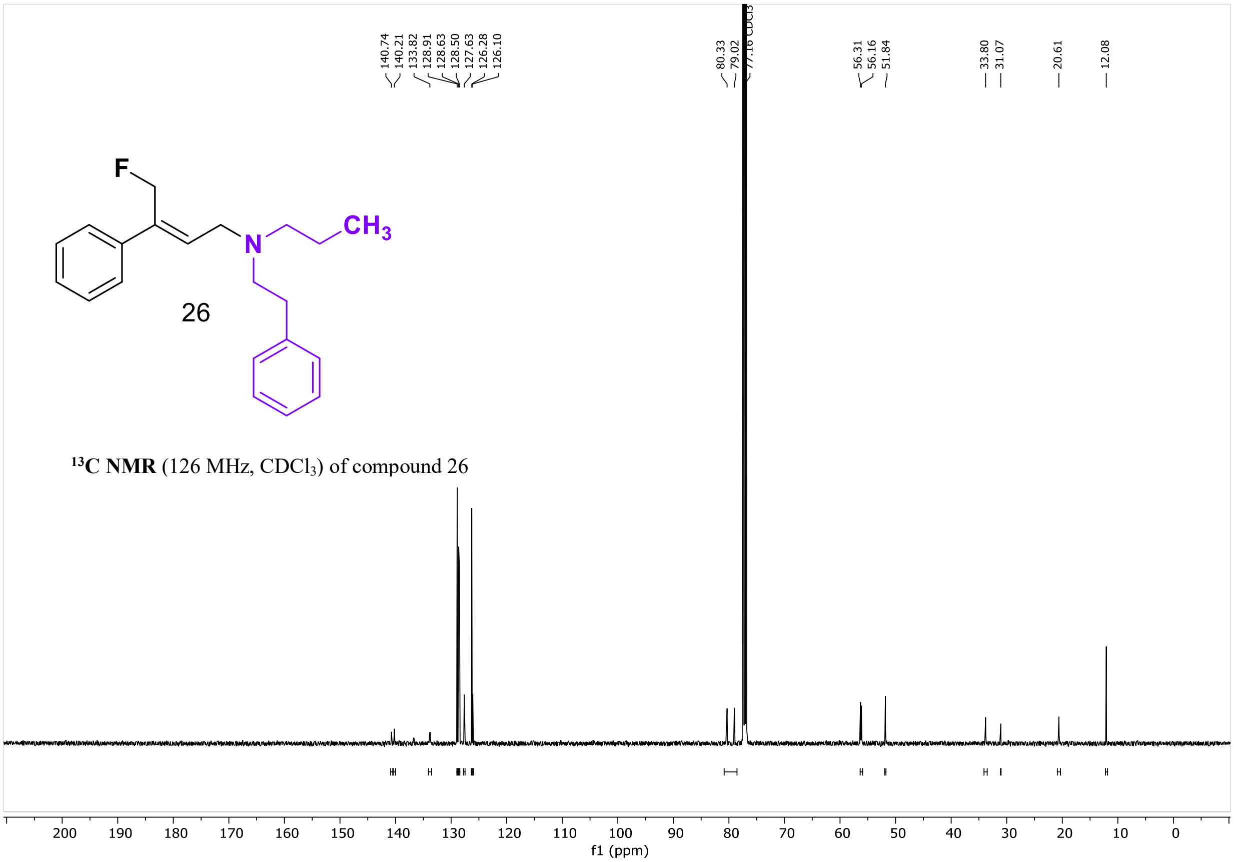

¹³C NMR (126 MHz, $CDCl_3$) of compound 26

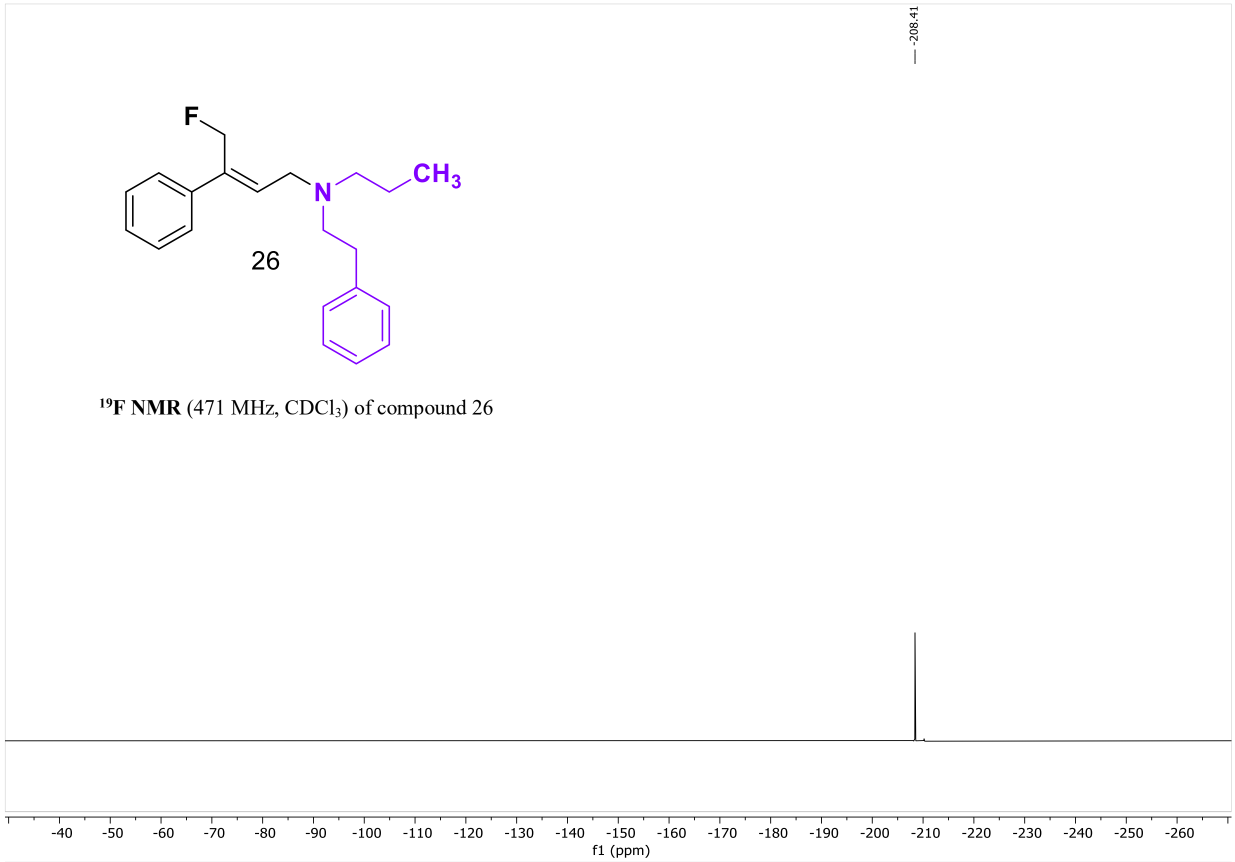

^{19}F NMR (471 MHz, $CDCl_3$) of compound 26

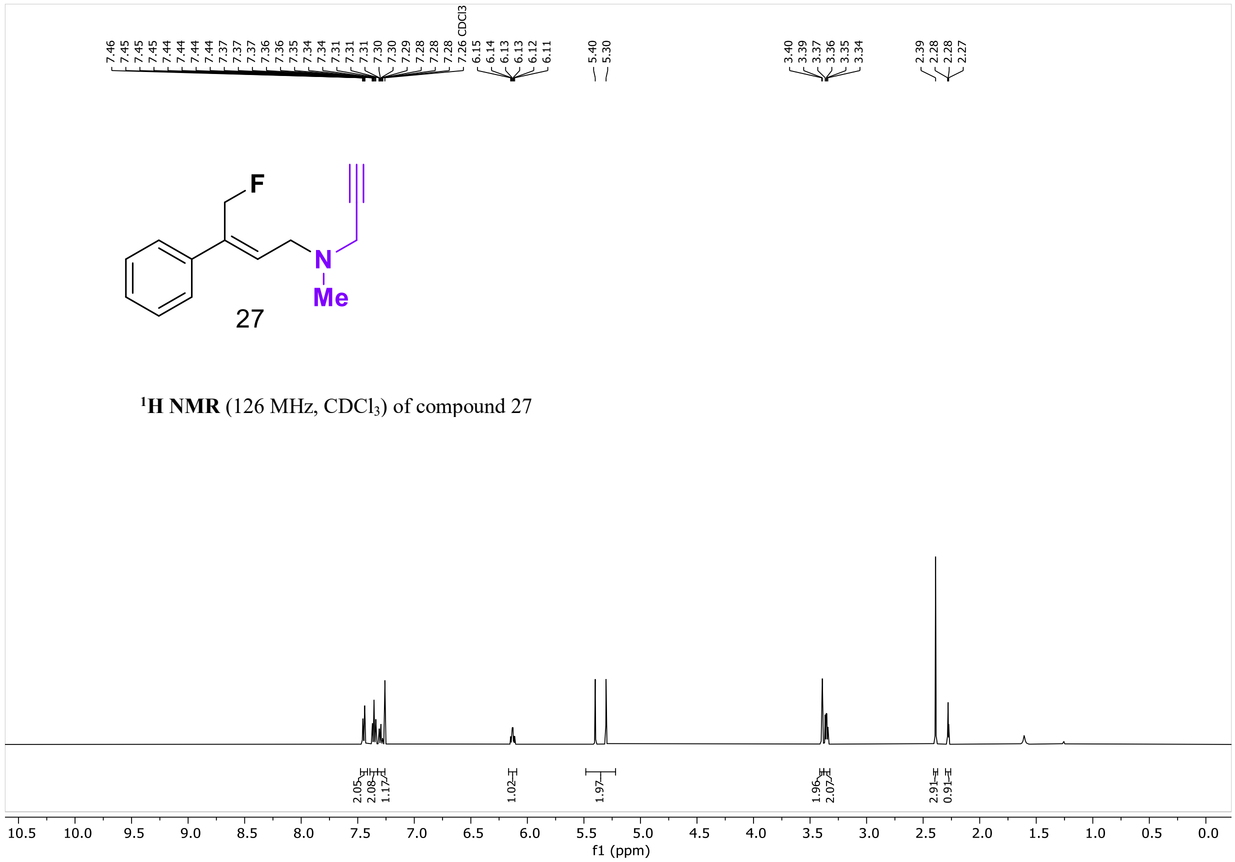

^{1}H NMR (126 MHz, $CDCl_3$) of compound 27

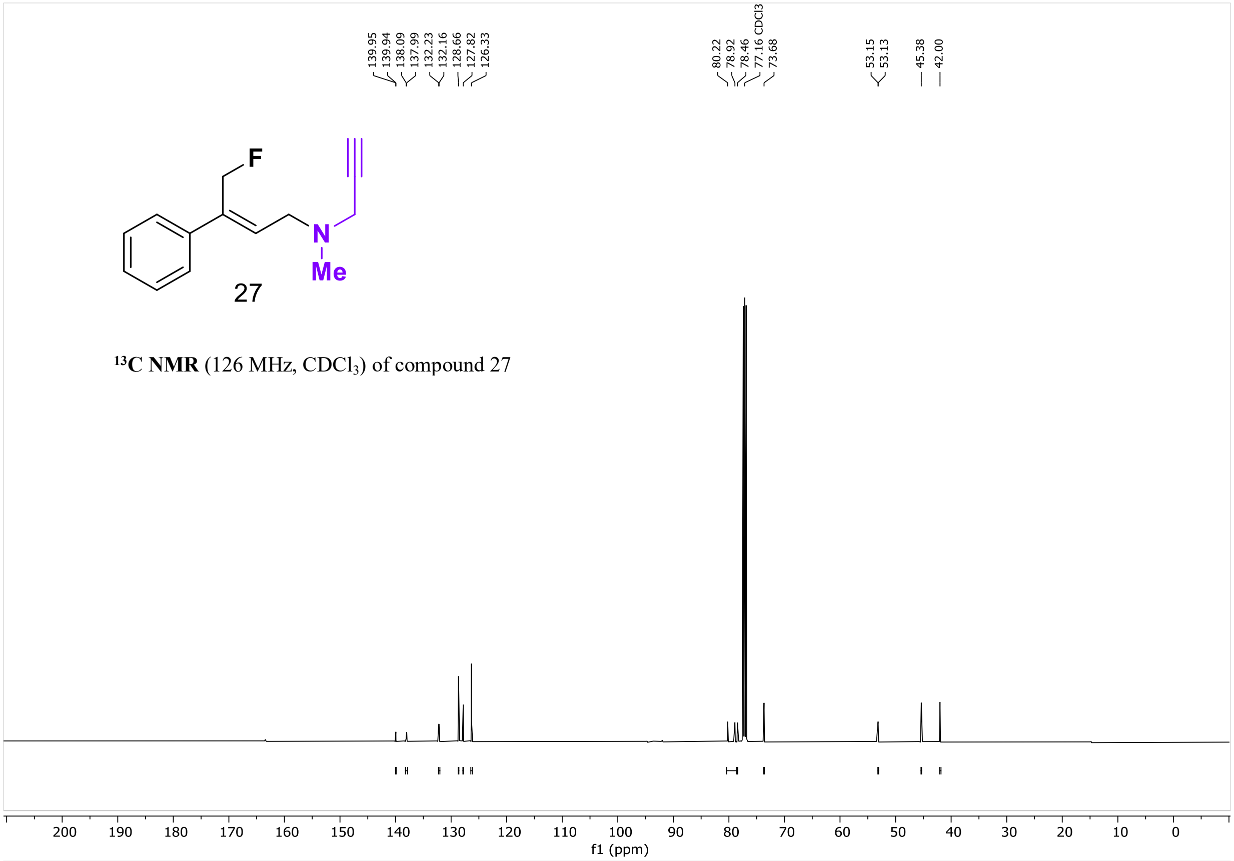

^{13}C NMR (126 MHz, $CDCl_3$) of compound 27

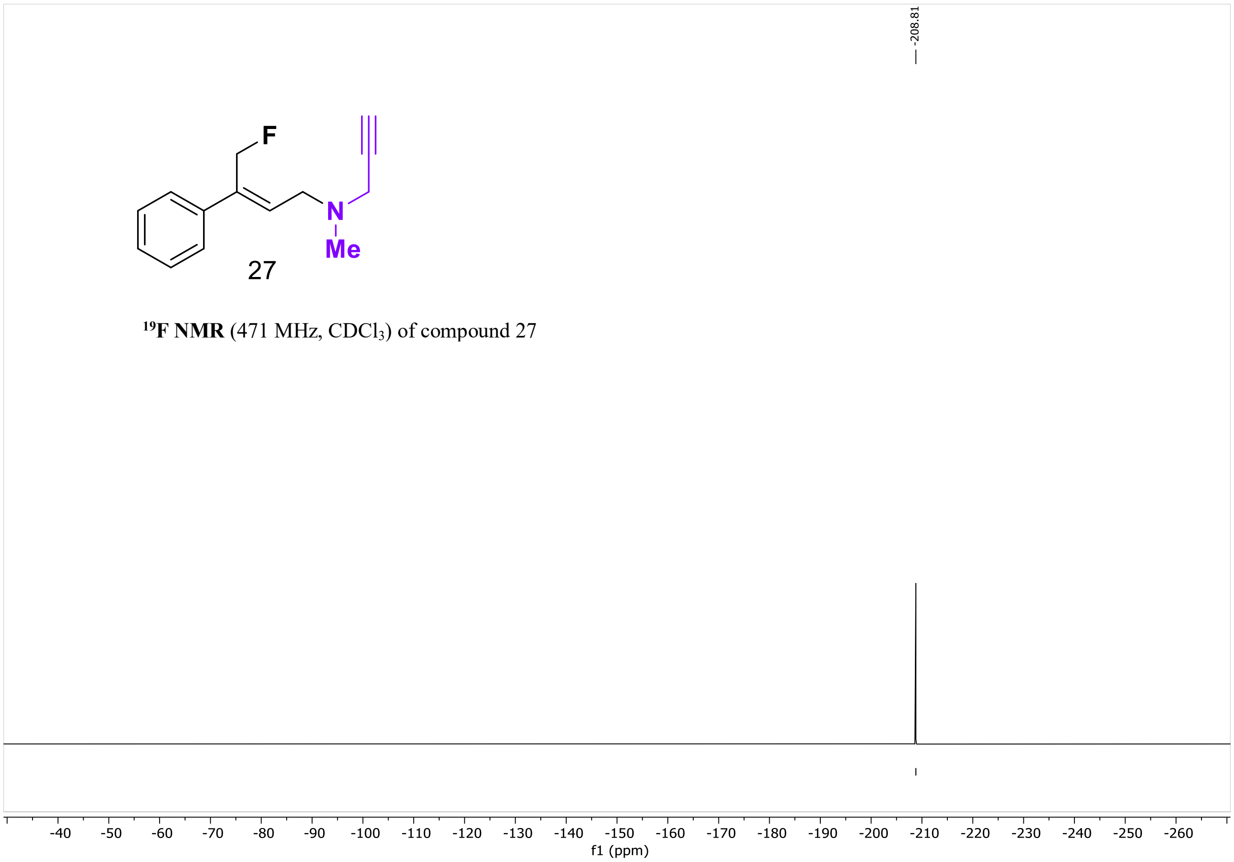

^{19}F NMR (471 MHz, $CDCl_3$) of compound 27

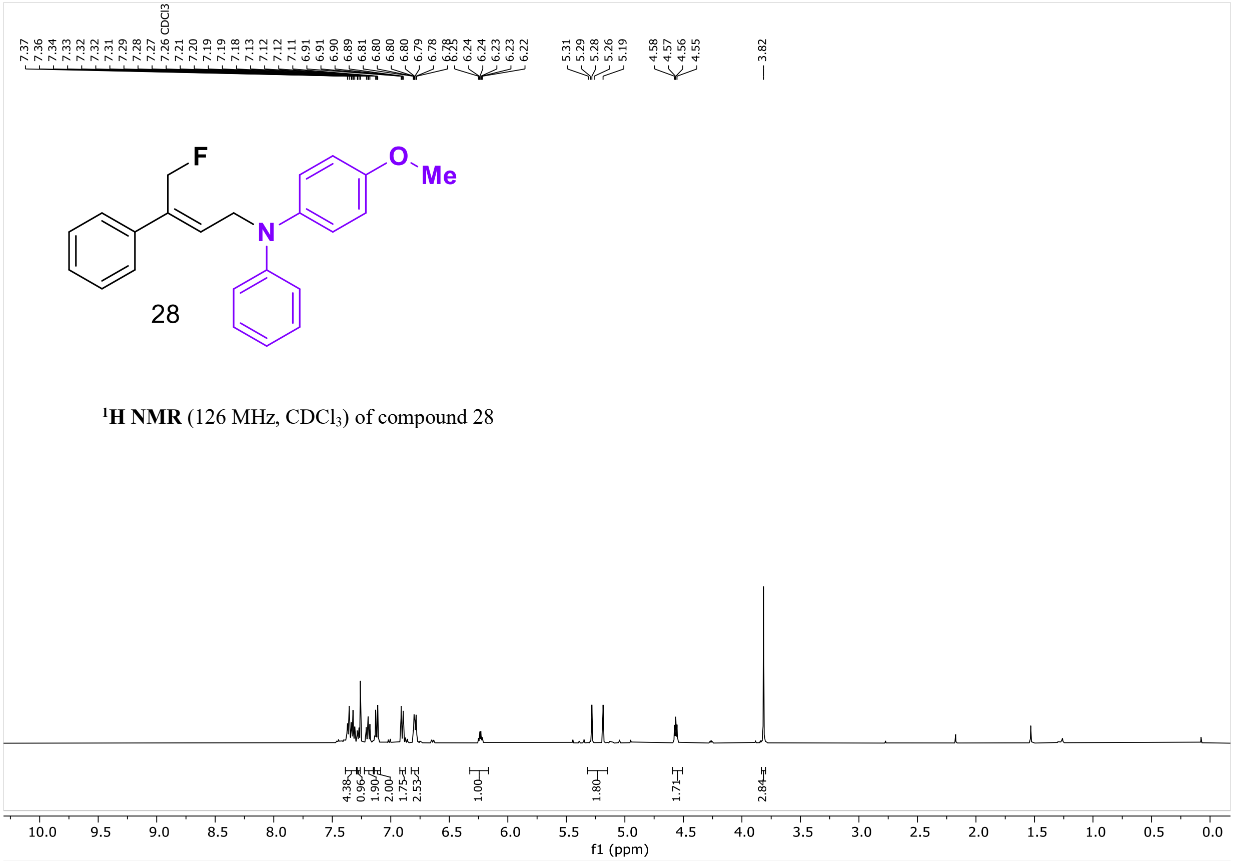

^{1}H NMR (126 MHz, $CDCl_3$) of compound 28

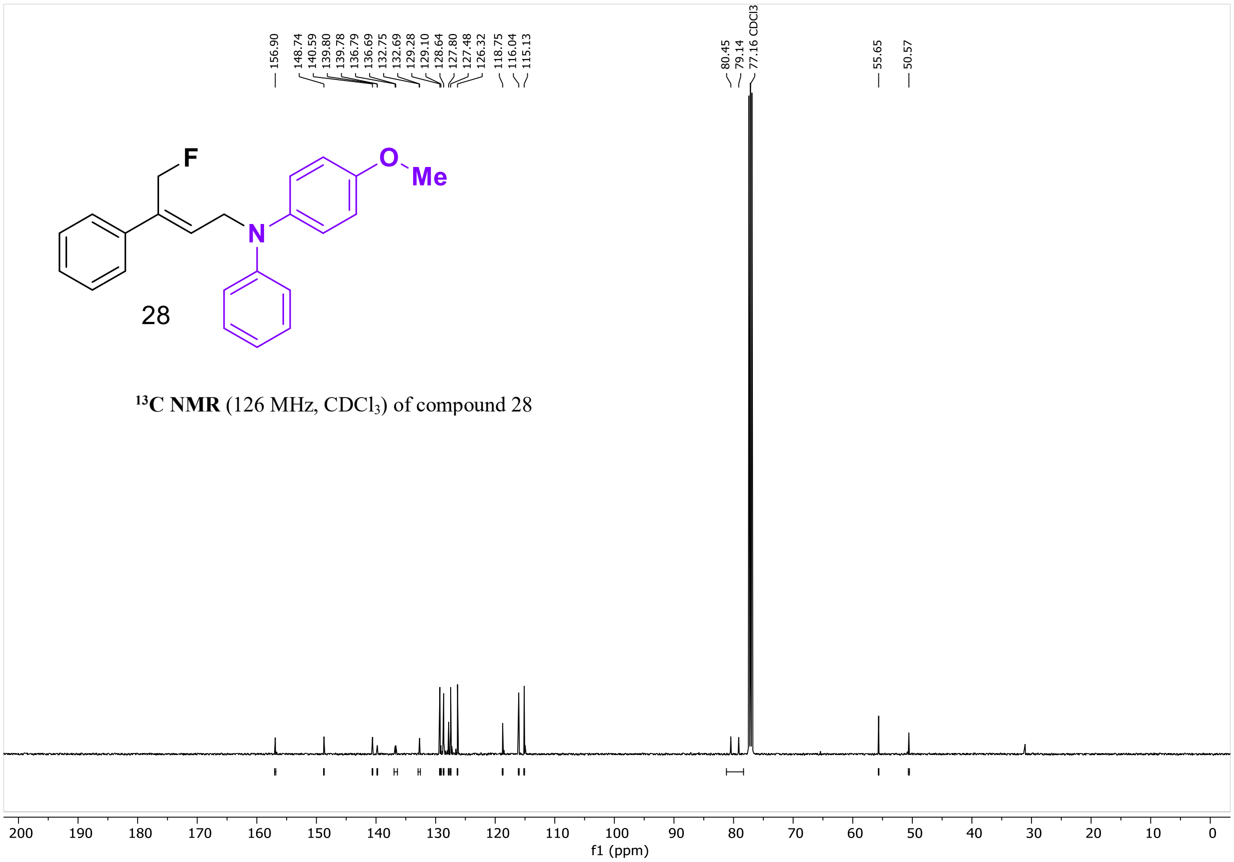

^{13}C NMR (126 MHz, $CDCl_3$) of compound 28

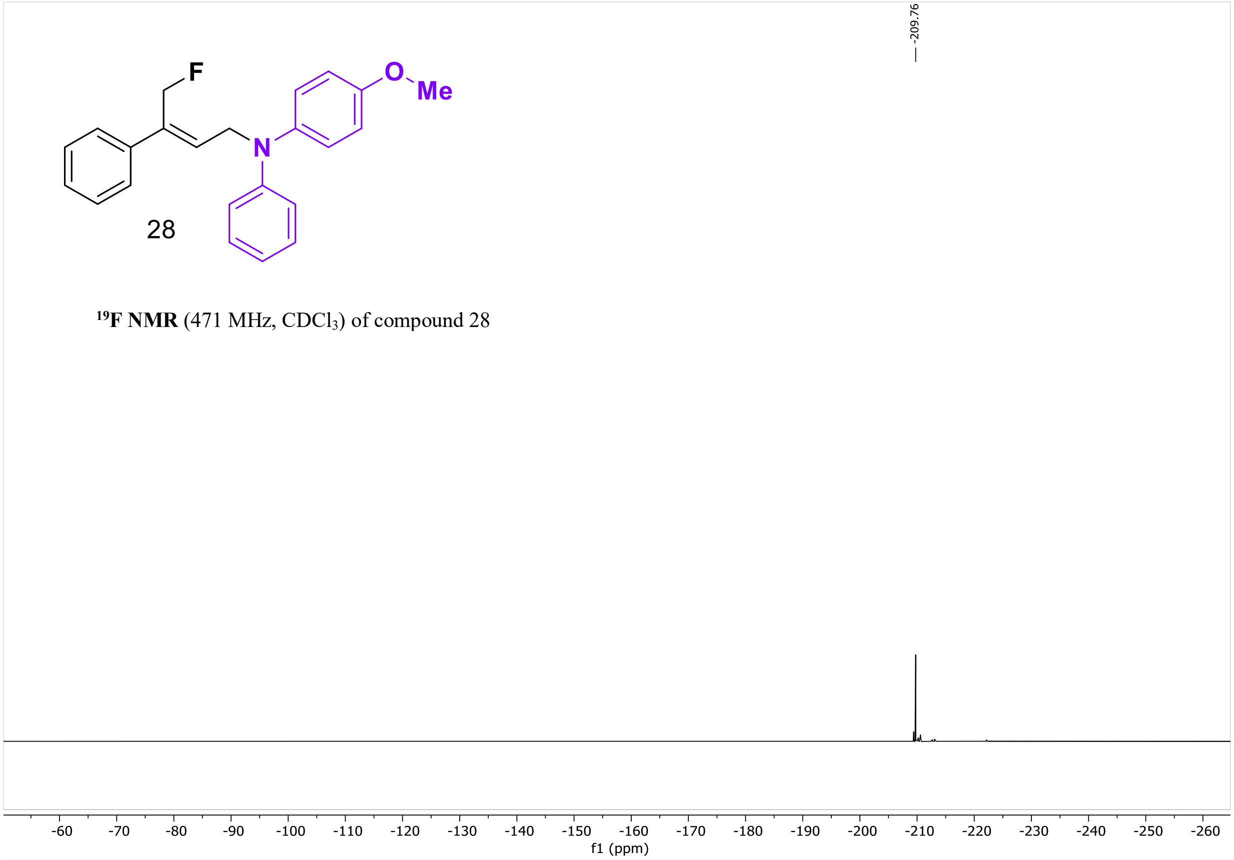

28

^{19}F NMR (471 MHz, $CDCl_3$) of compound 28

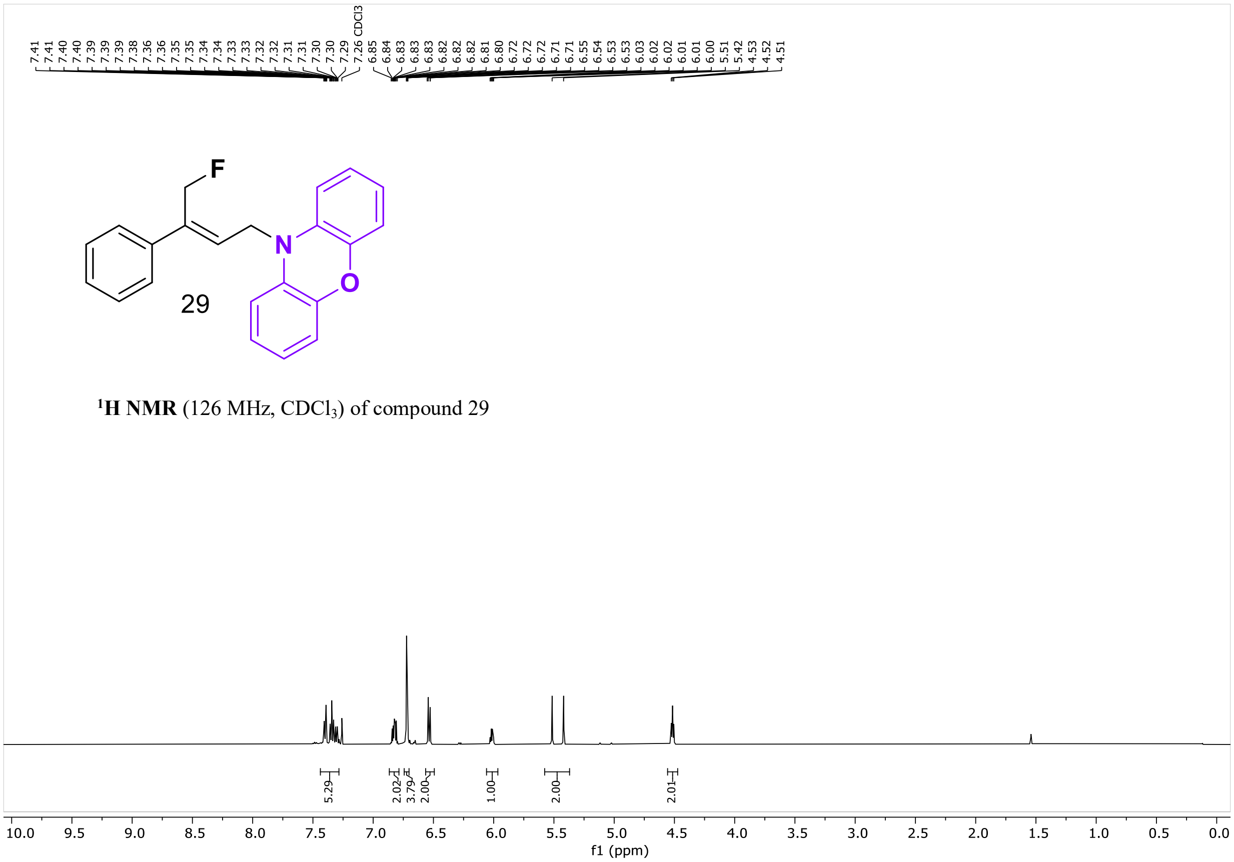

29

^{1}H NMR (126 MHz, $CDCl_3$) of compound 29

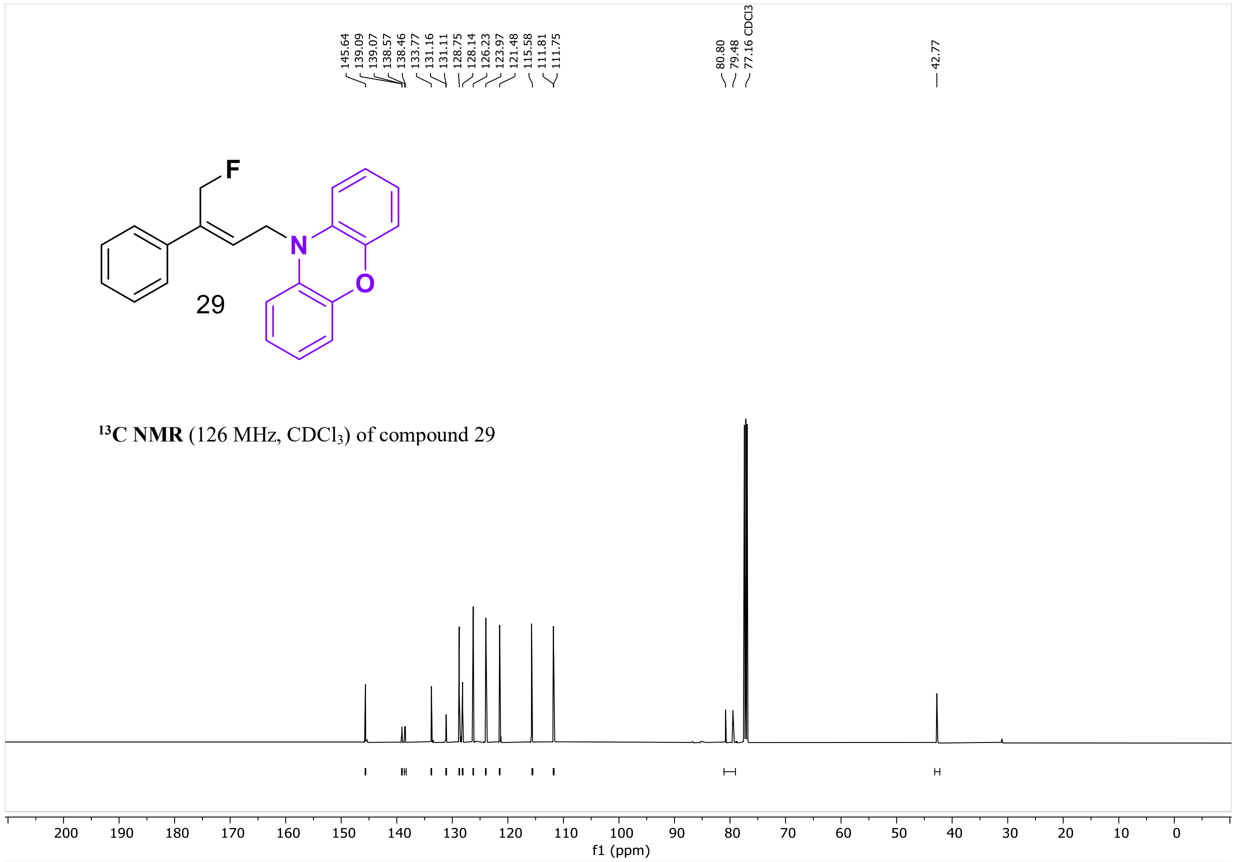

^{13}C NMR (126 MHz, $CDCl_3$) of compound 29

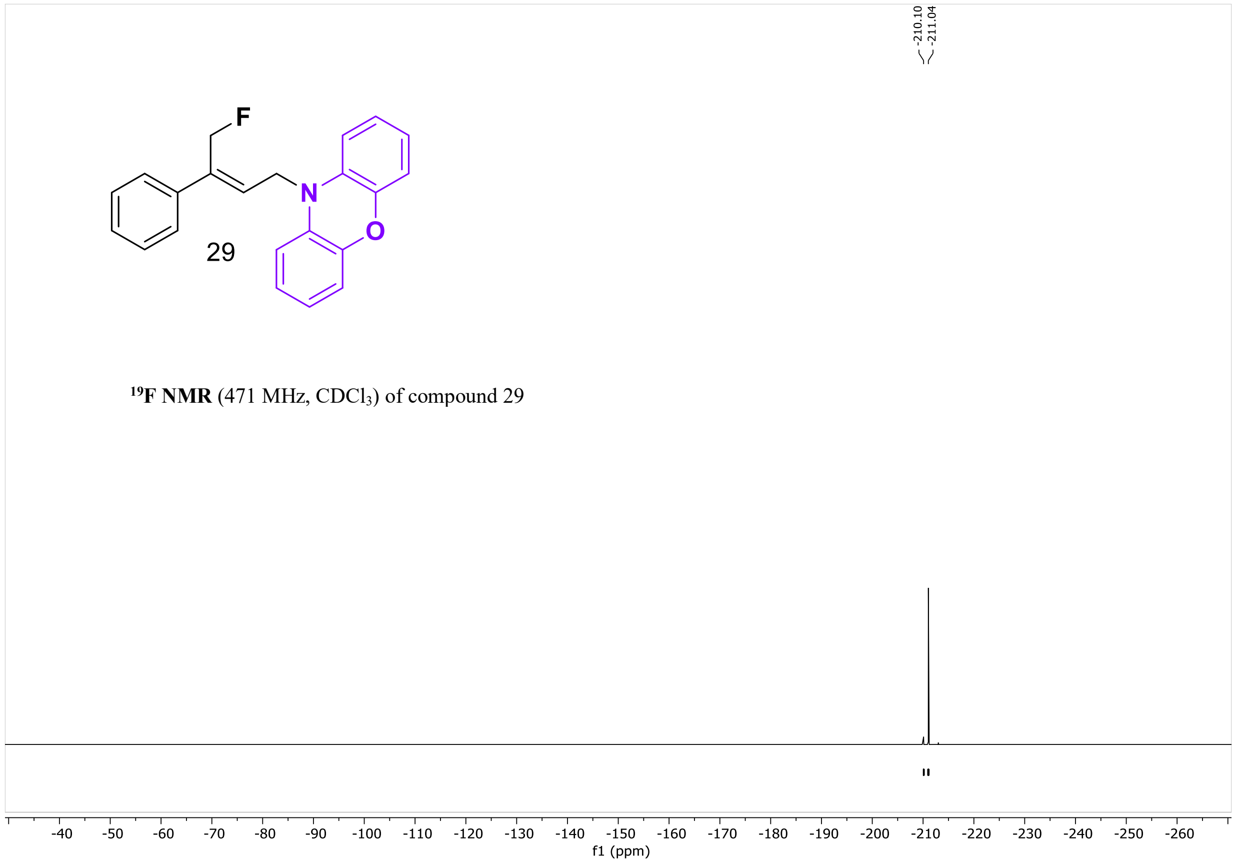

^{19}F NMR (471 MHz, $CDCl_3$) of compound 29

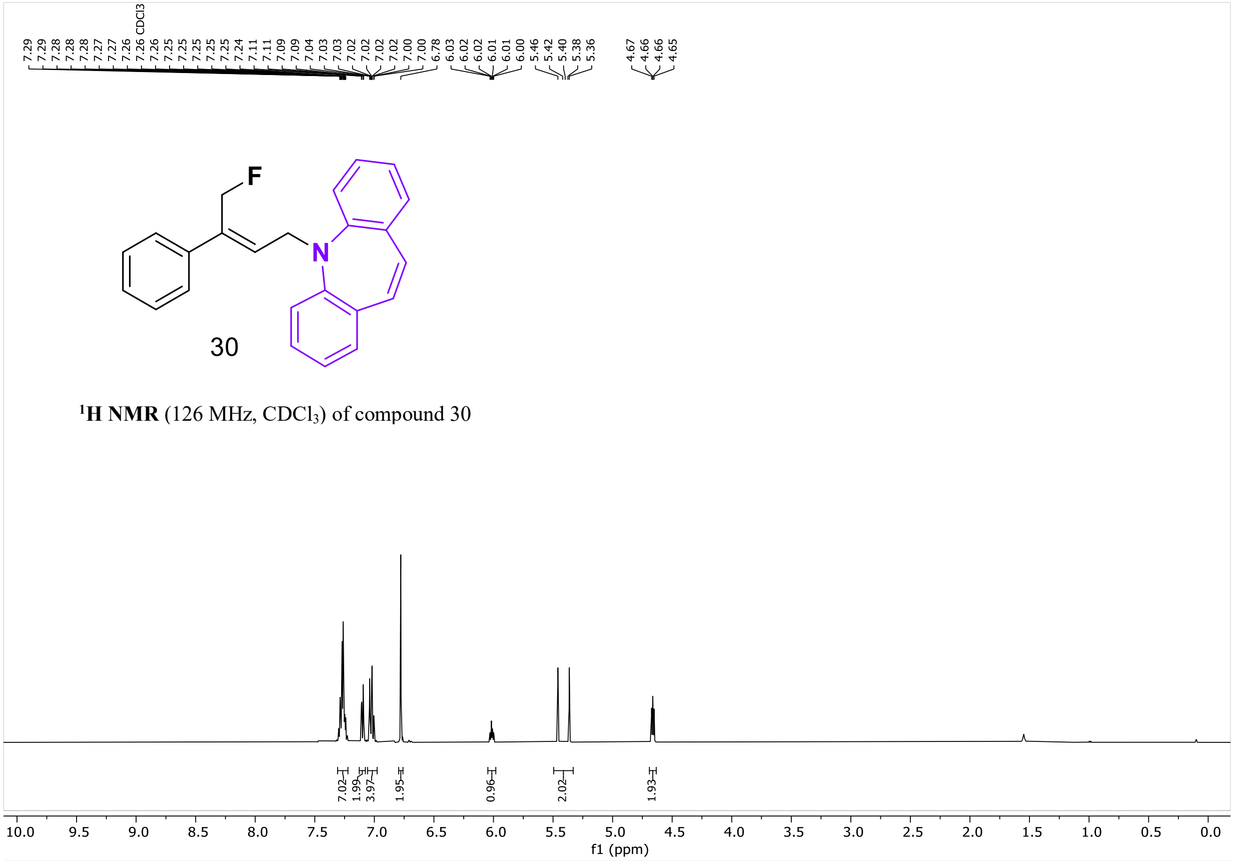

^{1}H NMR (126 MHz, $CDCl_3$) of compound 30

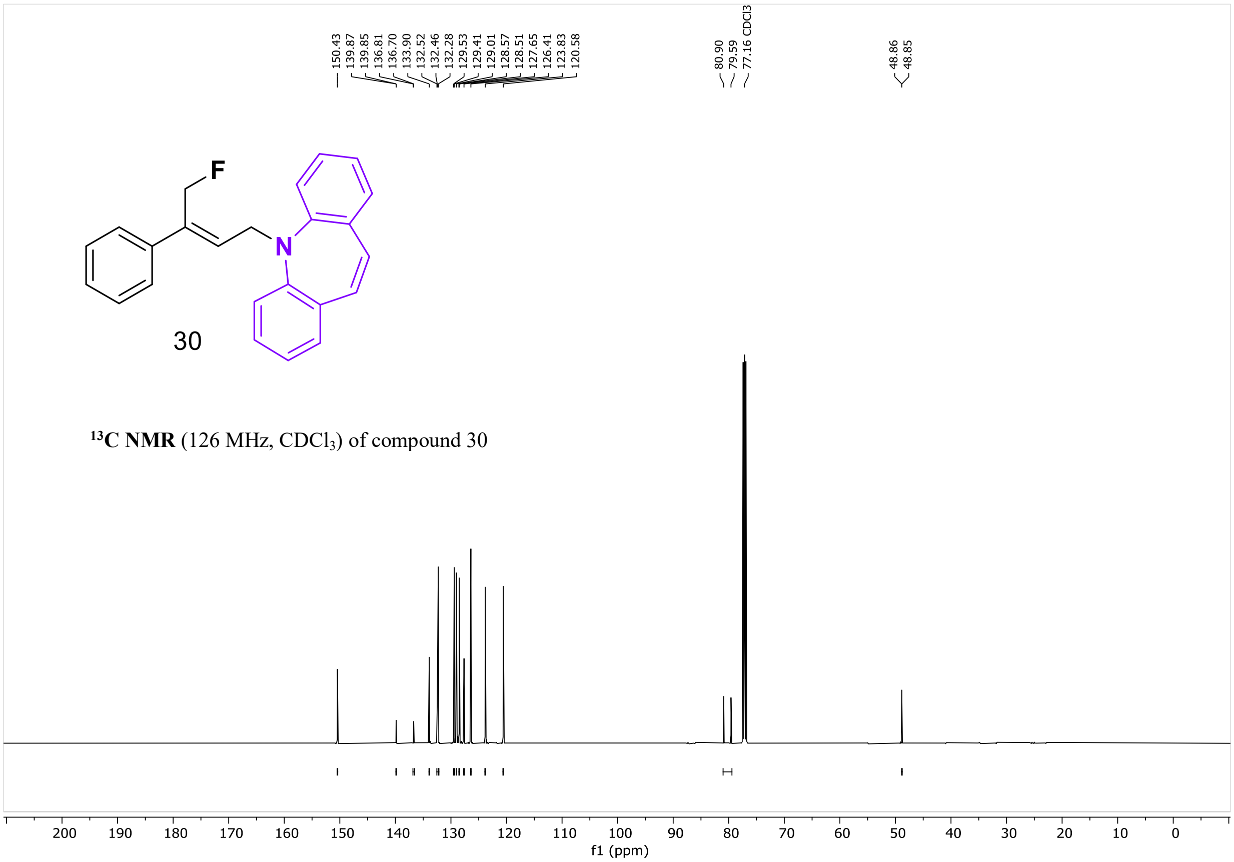

^{13}C NMR (126 MHz, $CDCl_3$) of compound 30

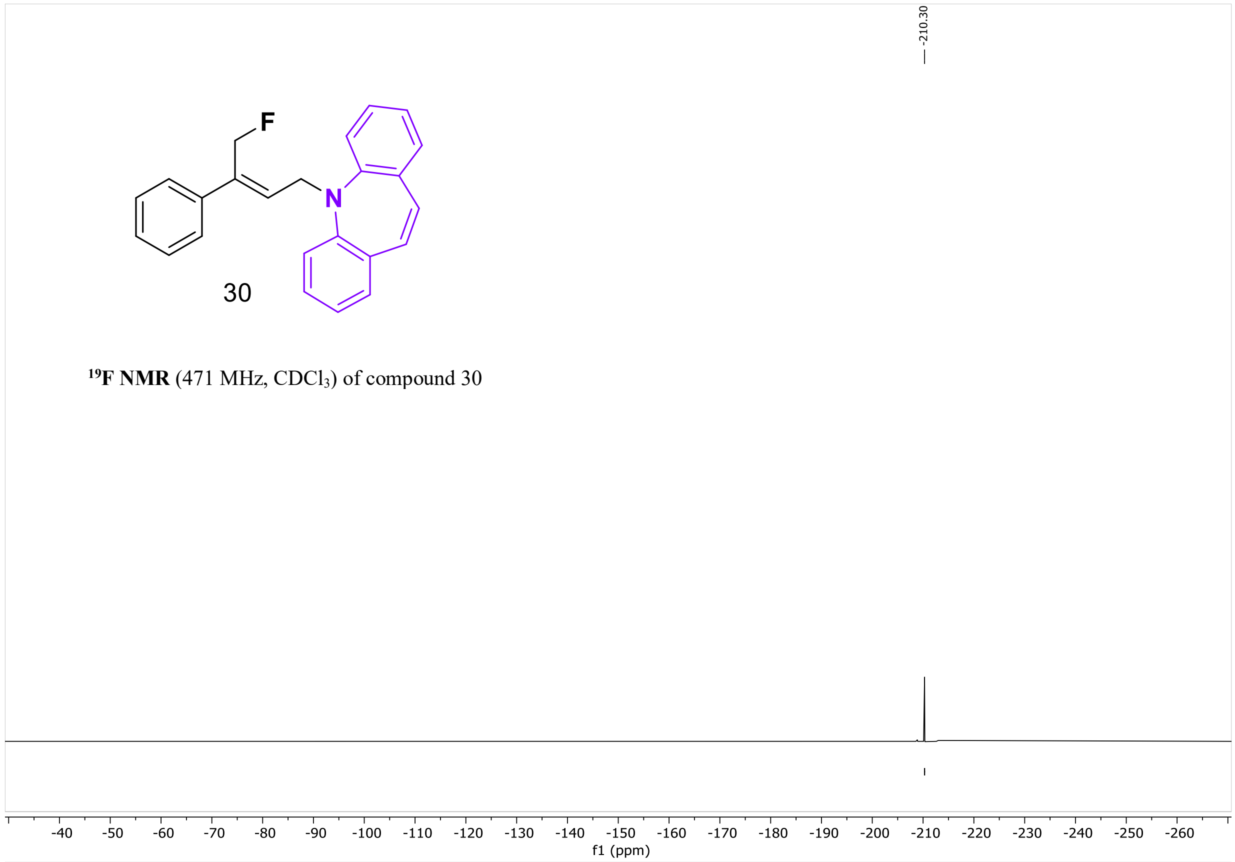

^{19}F NMR (471 MHz, $CDCl_3$) of compound 30

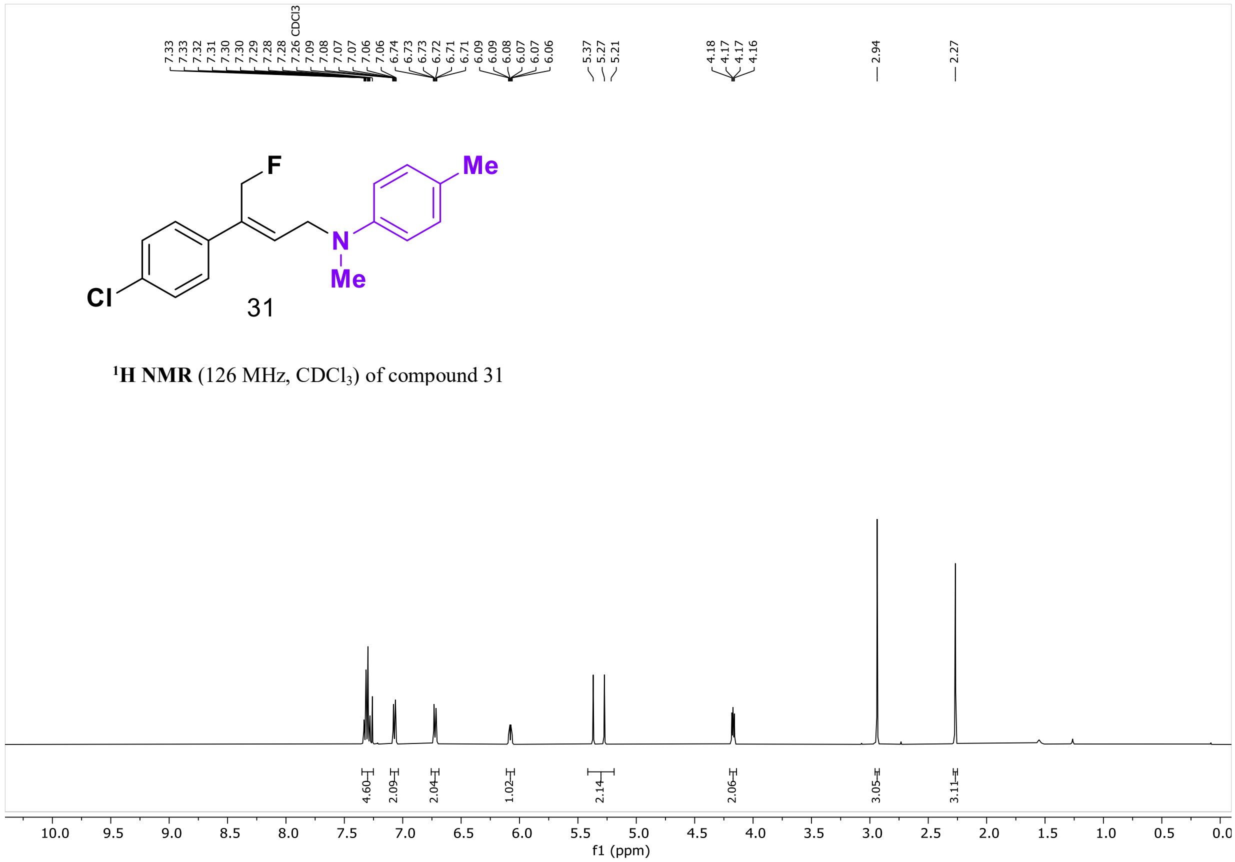

^{1}H NMR (126 MHz, $CDCl_3$) of compound 31

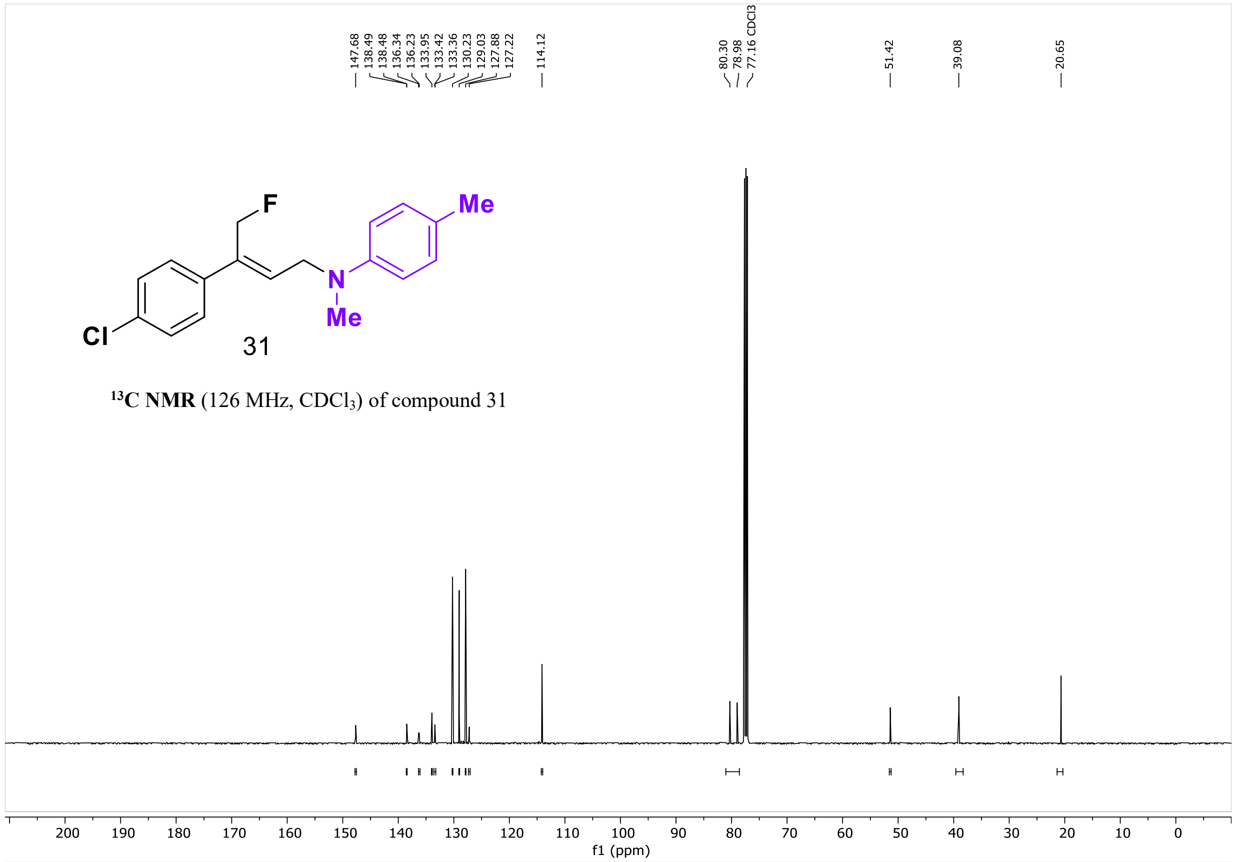

^{13}C NMR (126 MHz, $CDCl_3$) of compound 31

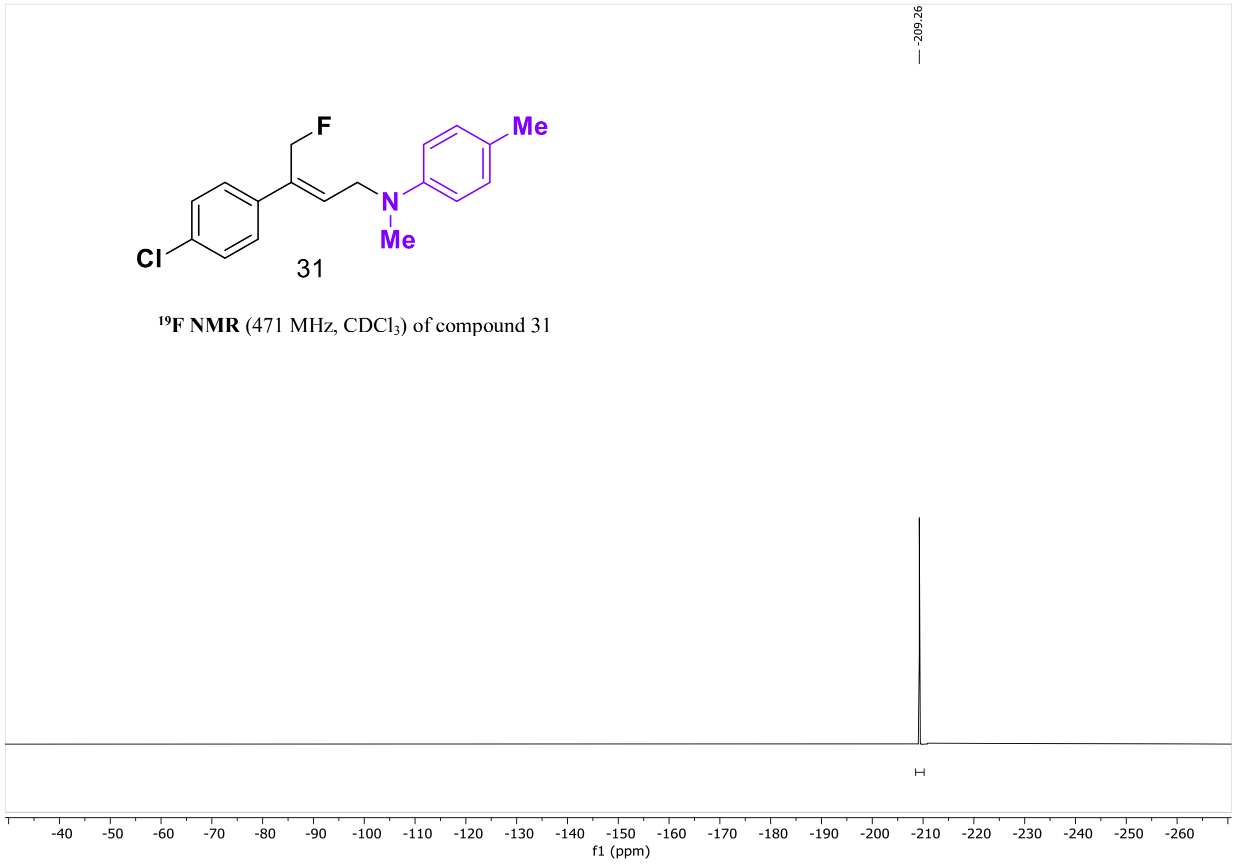

^{19}F NMR (471 MHz, $CDCl_3$) of compound 31

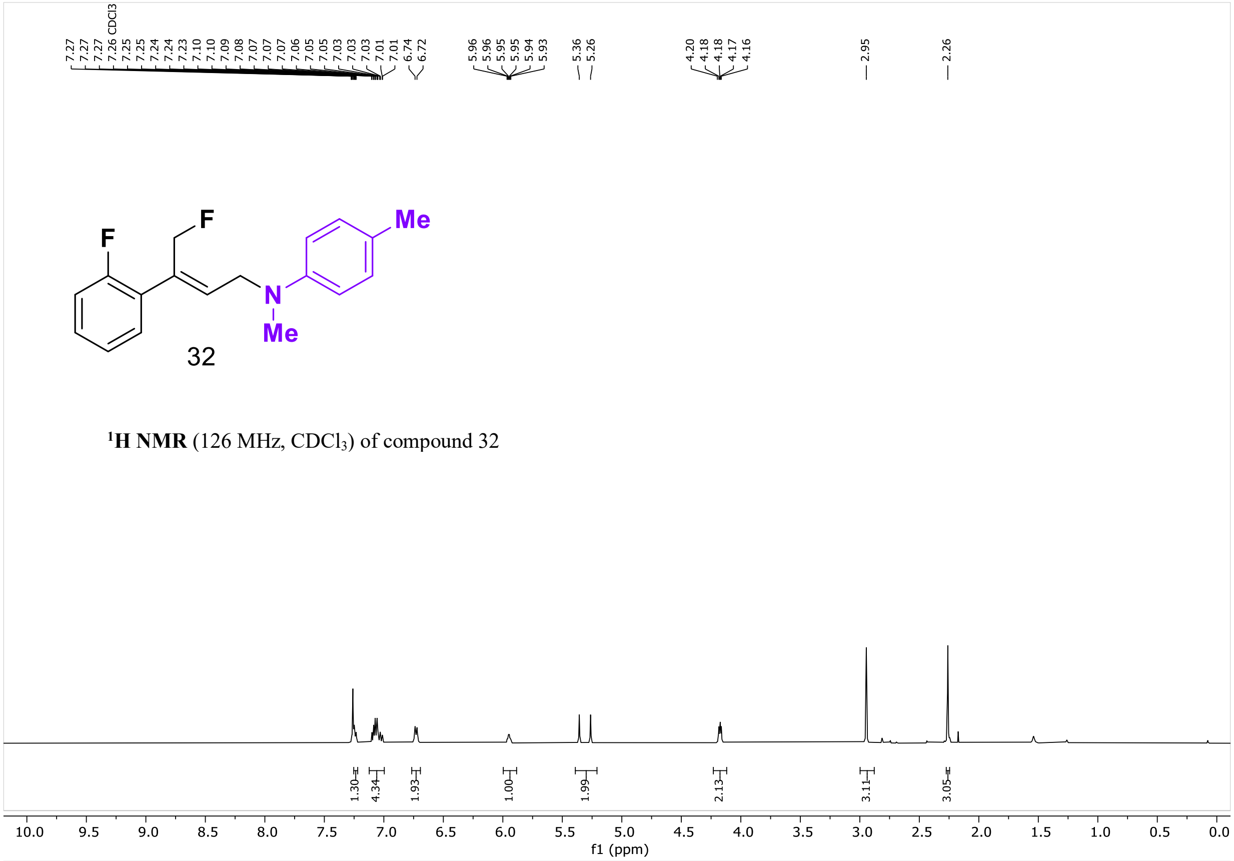

^{1}H NMR (126 MHz, $CDCl_3$) of compound 32

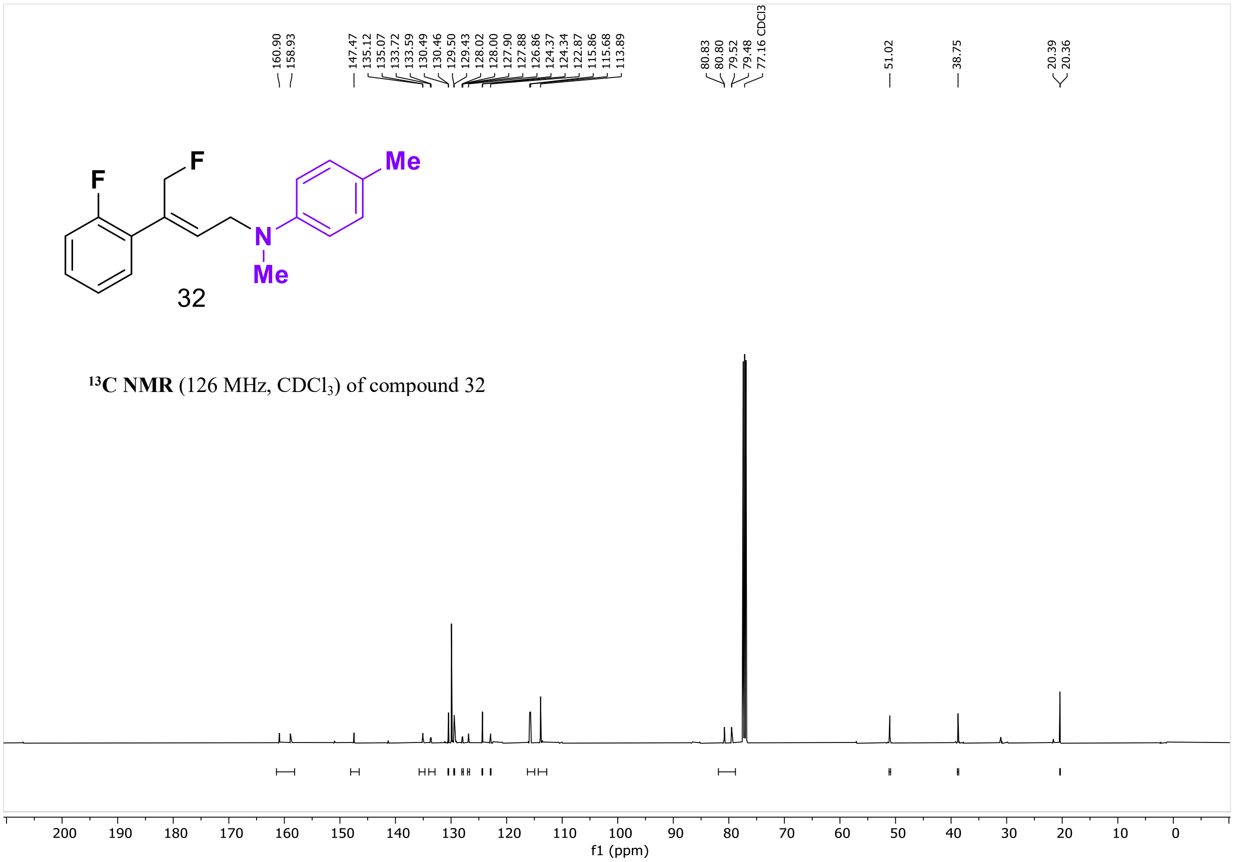

^{13}C NMR (126 MHz, $CDCl_3$) of compound 32

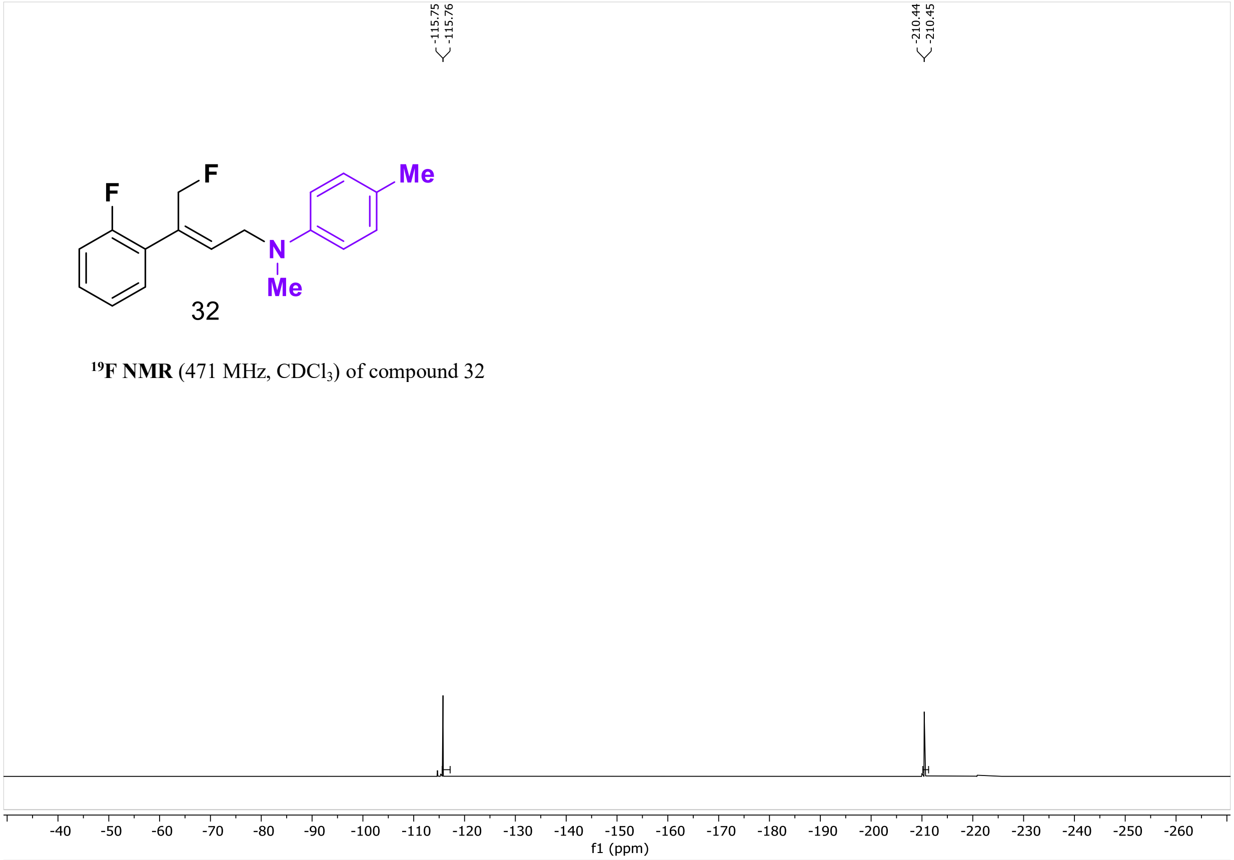

^{19}F NMR (471 MHz, $CDCl_3$) of compound 32

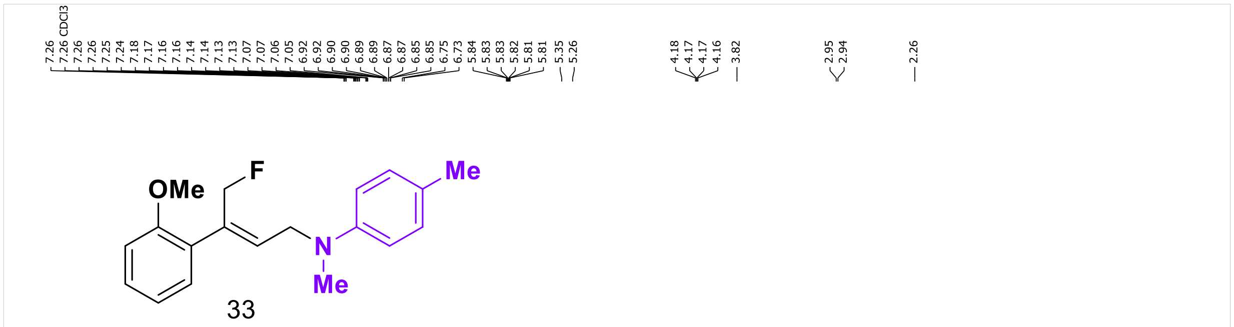

^{1}H NMR (126 MHz, $CDCl_3$) of compound 33

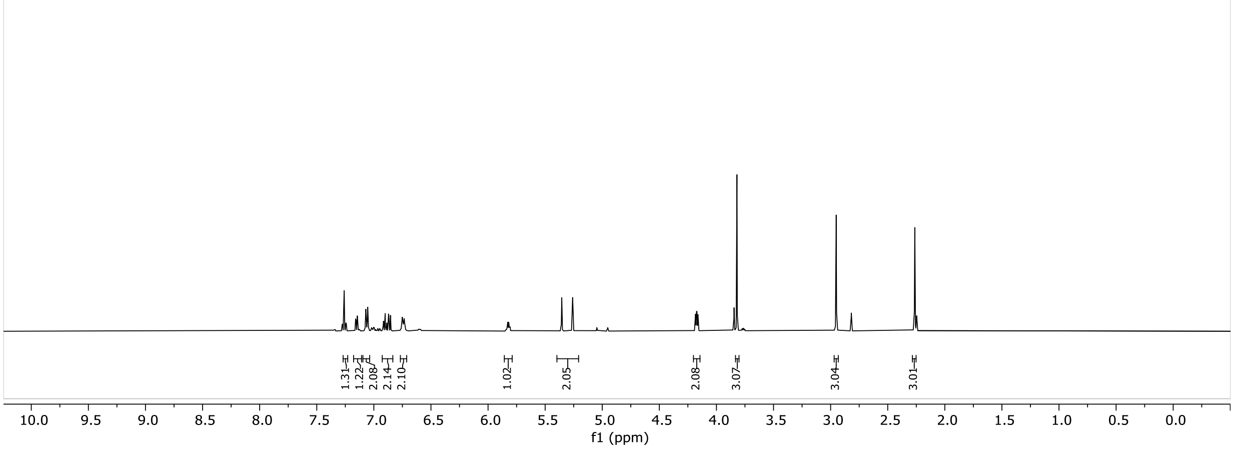

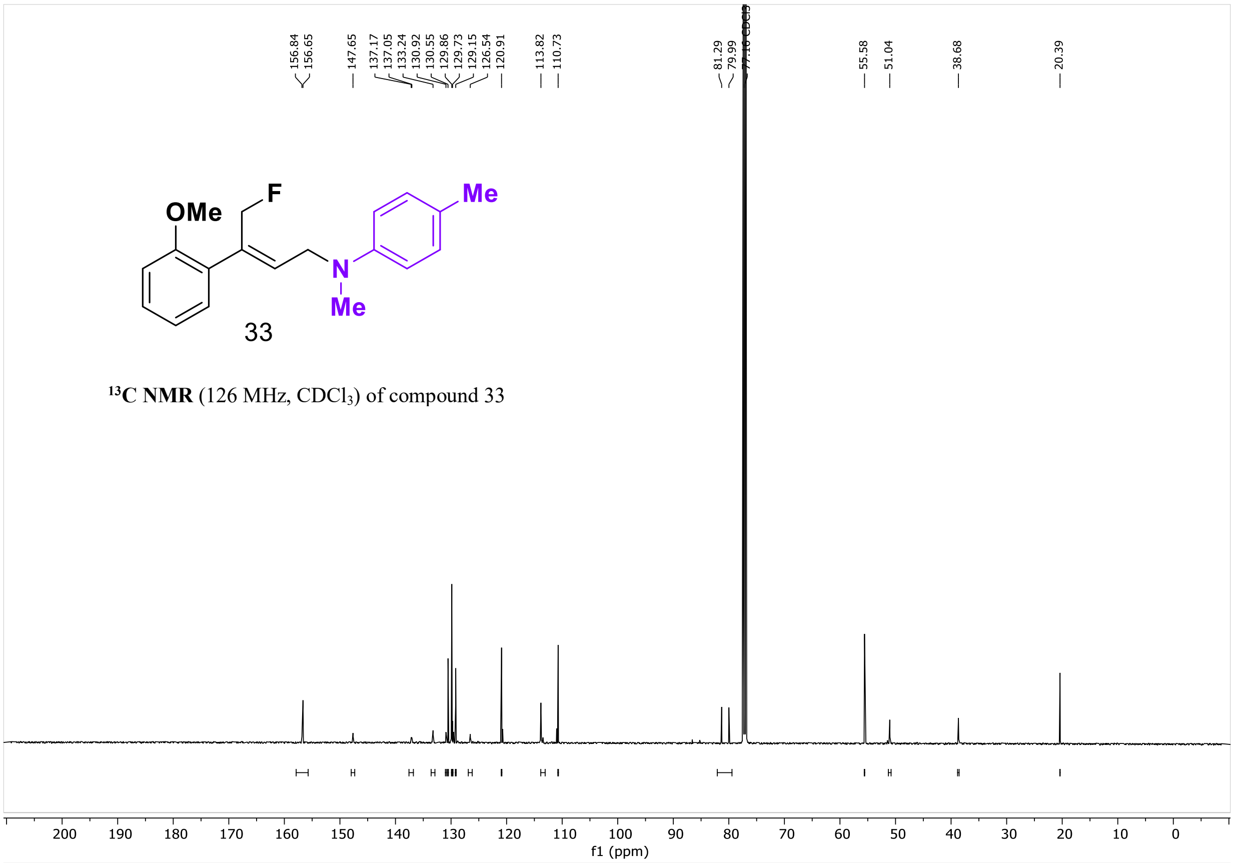

^{13}**C NMR** (126 MHz, $CDCl_3$) of compound 33

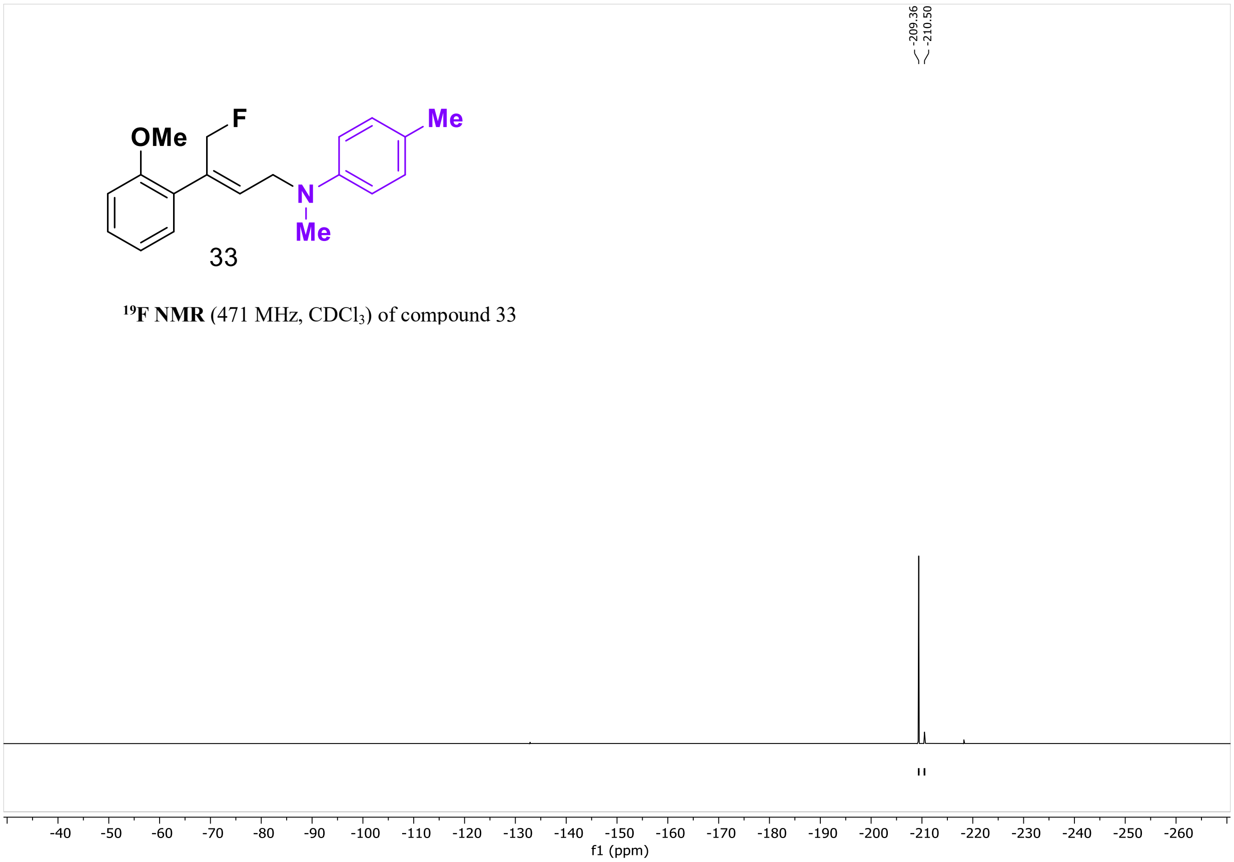

^{19}**F NMR** (471 MHz, $CDCl_3$) of compound 33

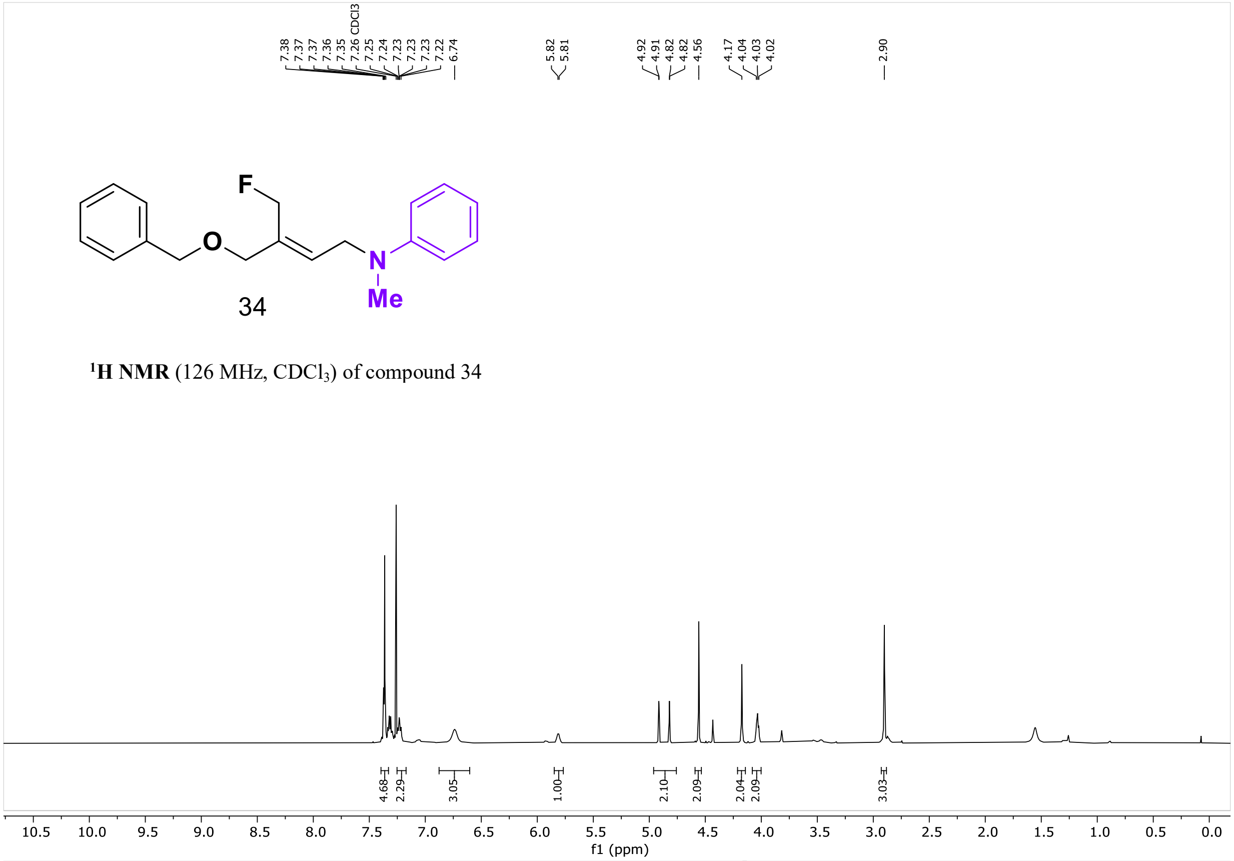

^{1}H NMR (126 MHz, $CDCl_3$) of compound 34

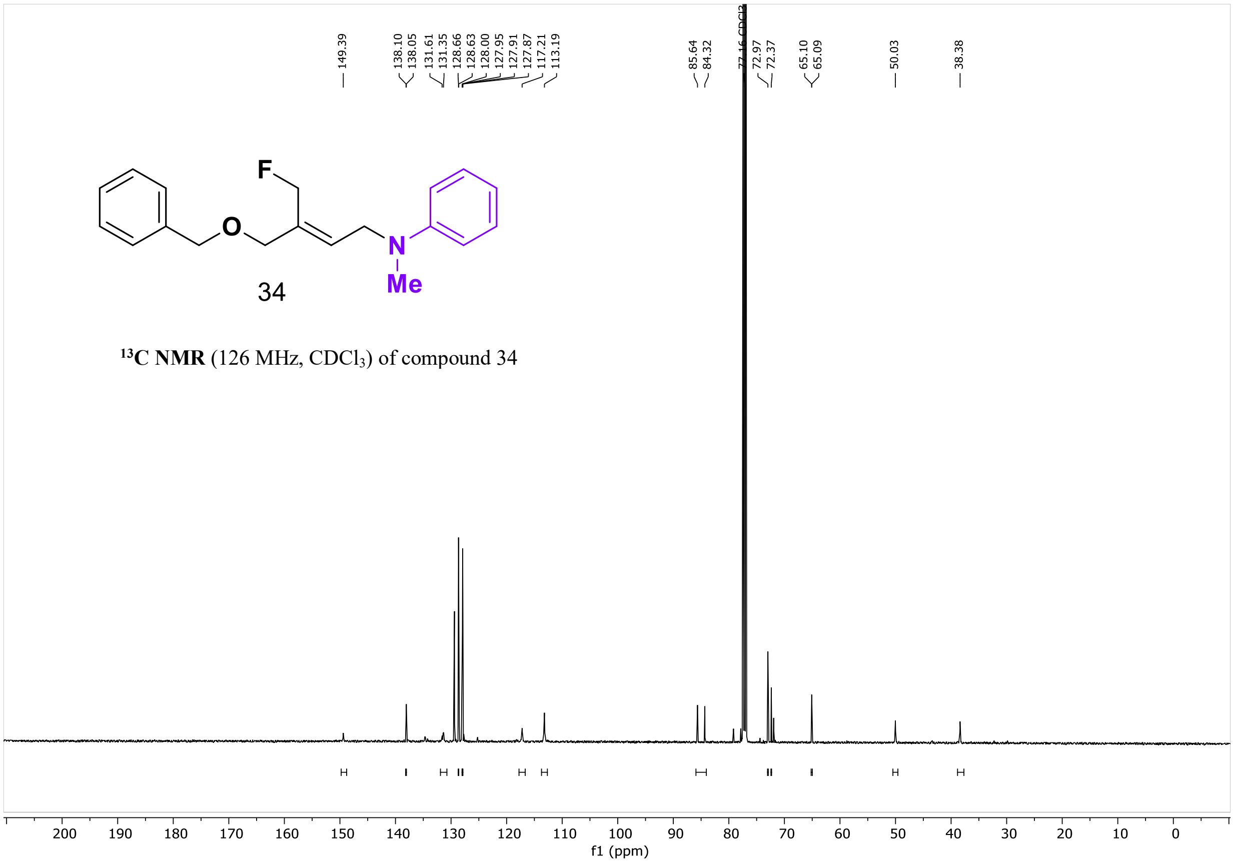

^{13}C NMR (126 MHz, $CDCl_3$) of compound 34

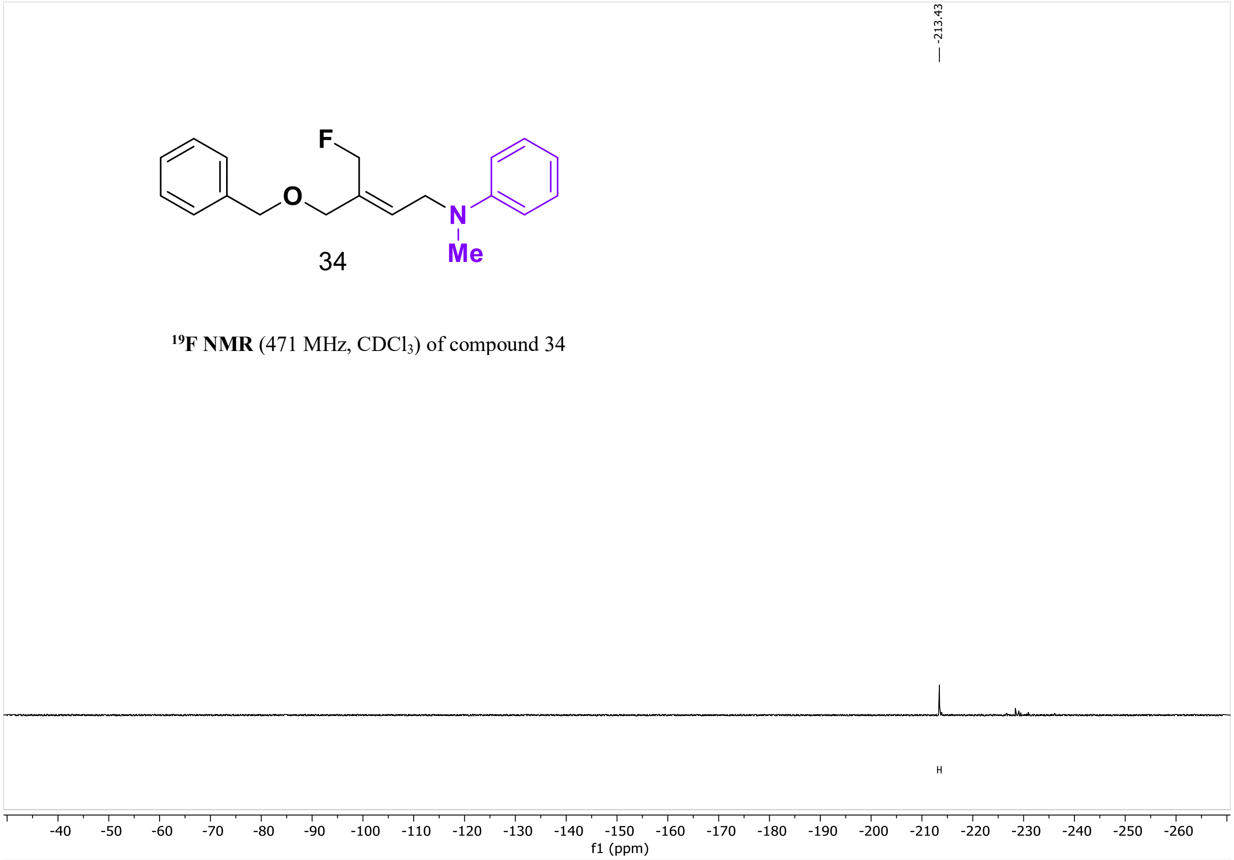

^{19}F NMR (471 MHz, $CDCl_3$) of compound 34

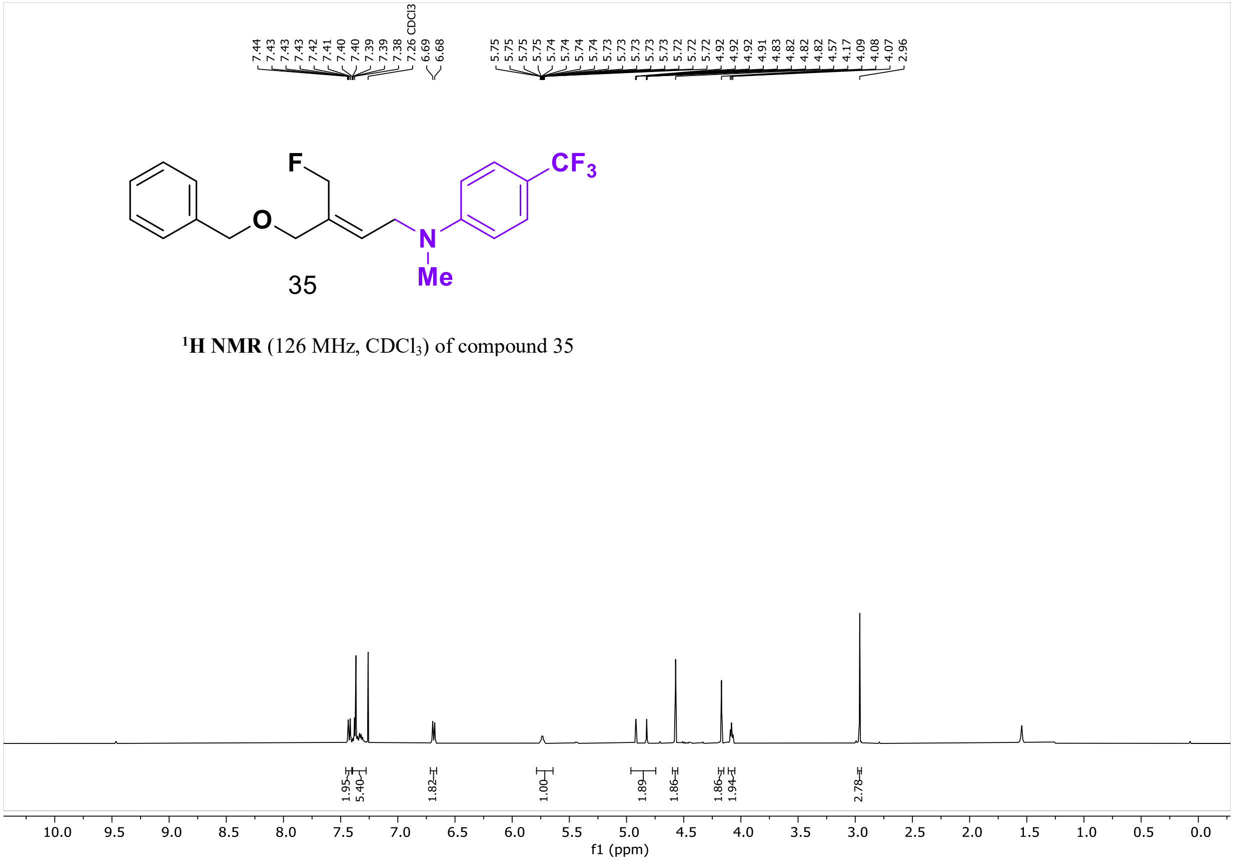

^{1}H NMR (126 MHz, $CDCl_3$) of compound 35

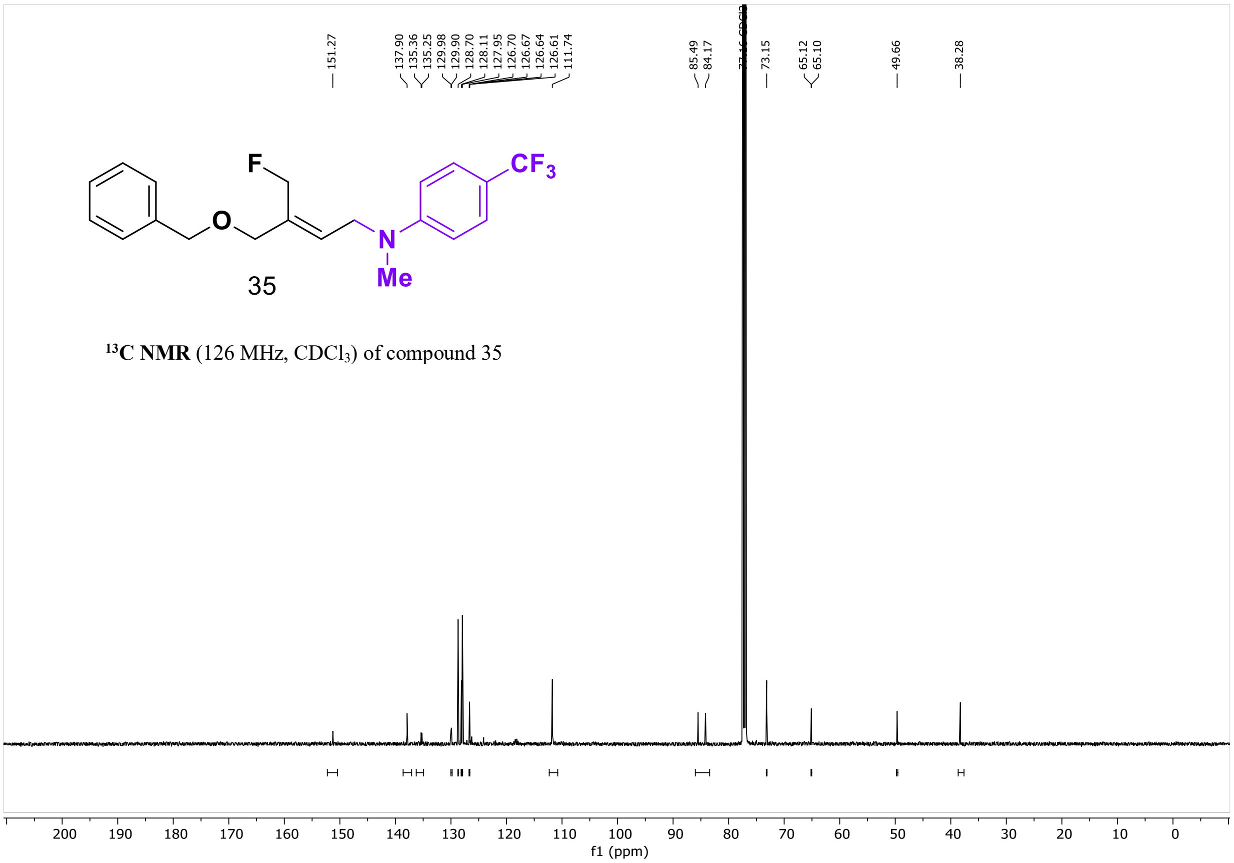

^{13}**C NMR** (126 MHz, $CDCl_3$) of compound 35

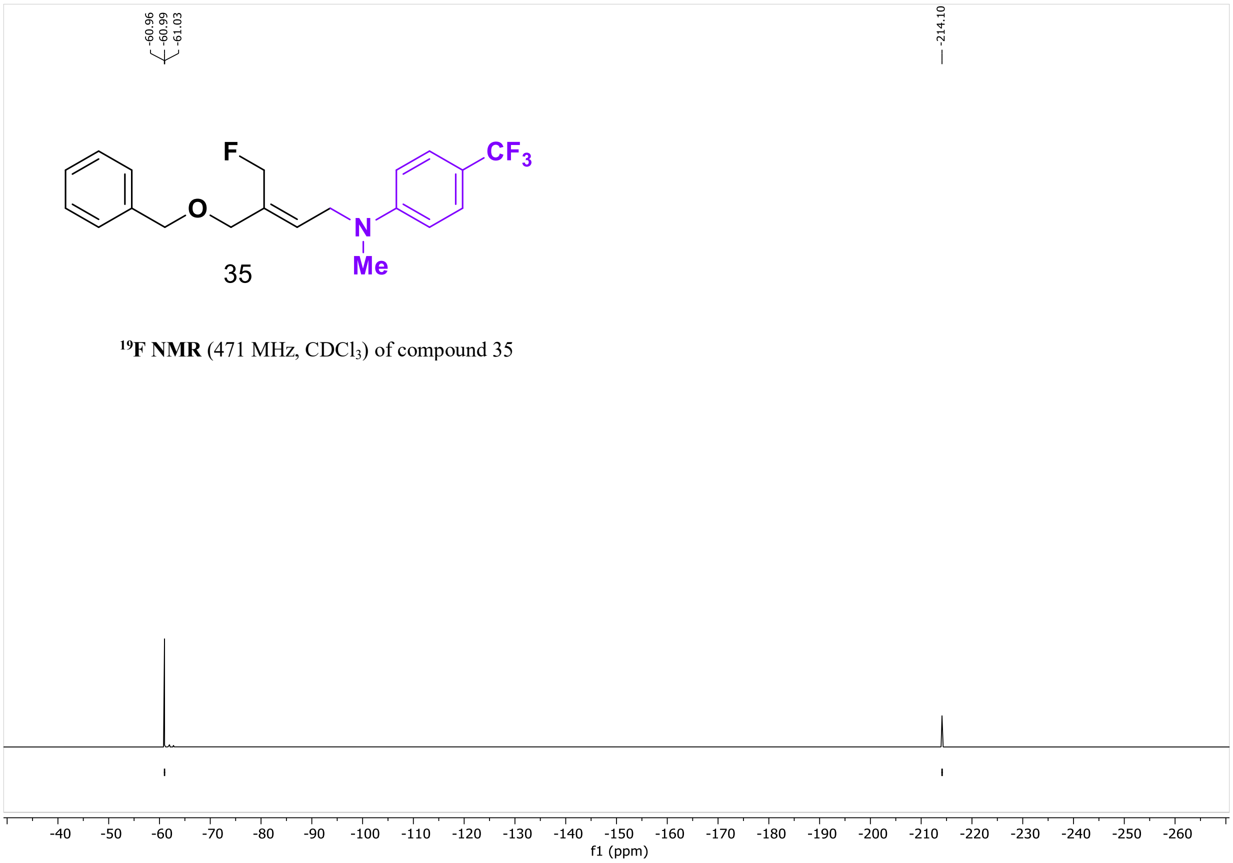

^{19}**F NMR** (471 MHz, $CDCl_3$) of compound 35

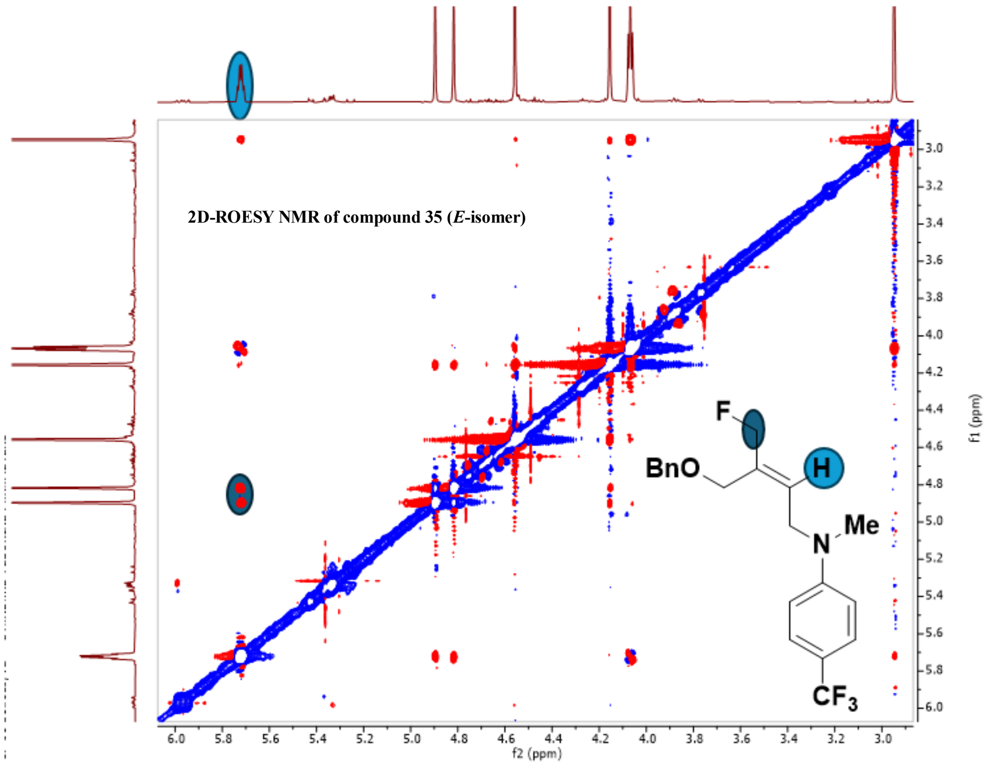
2D-ROESY NMR of compound 35 (*E*-isomer)
BnO
F
H
N
Me
CF3
f2 (ppm)
f1 (ppm)

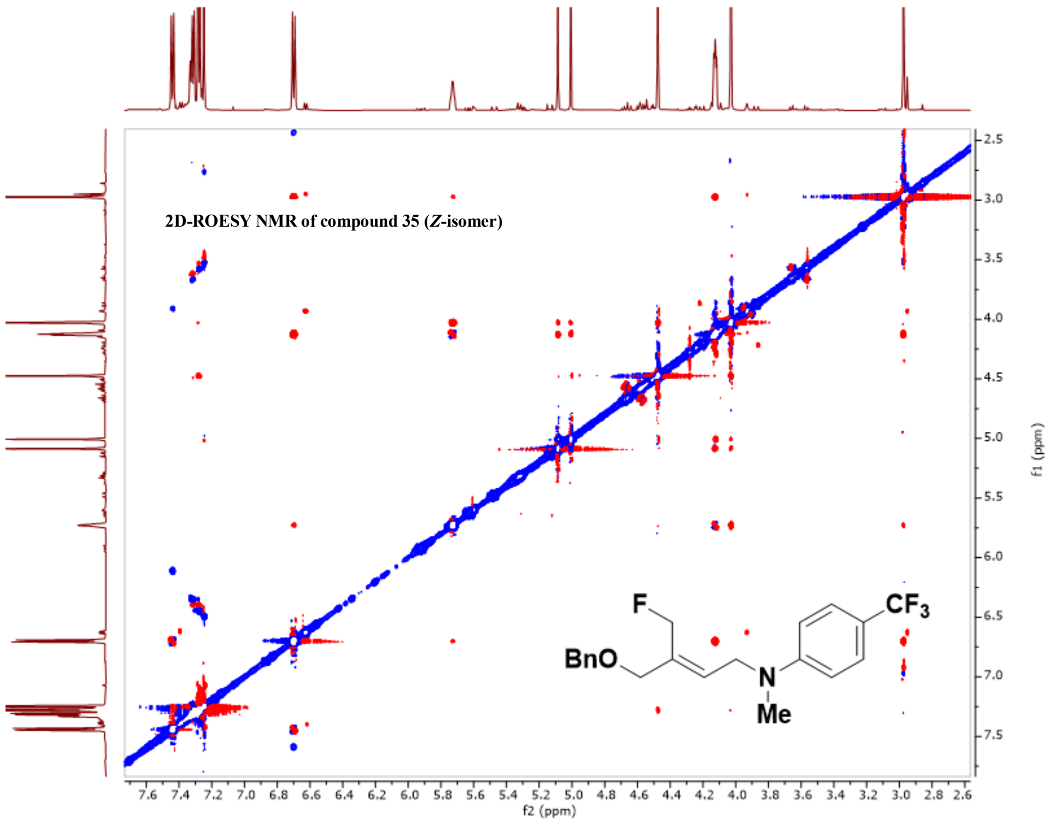
2D-ROESY NMR of compound 35 (*Z*-isomer)
BnO
F
N
Me
CF3
f2 (ppm)
f1 (ppm)

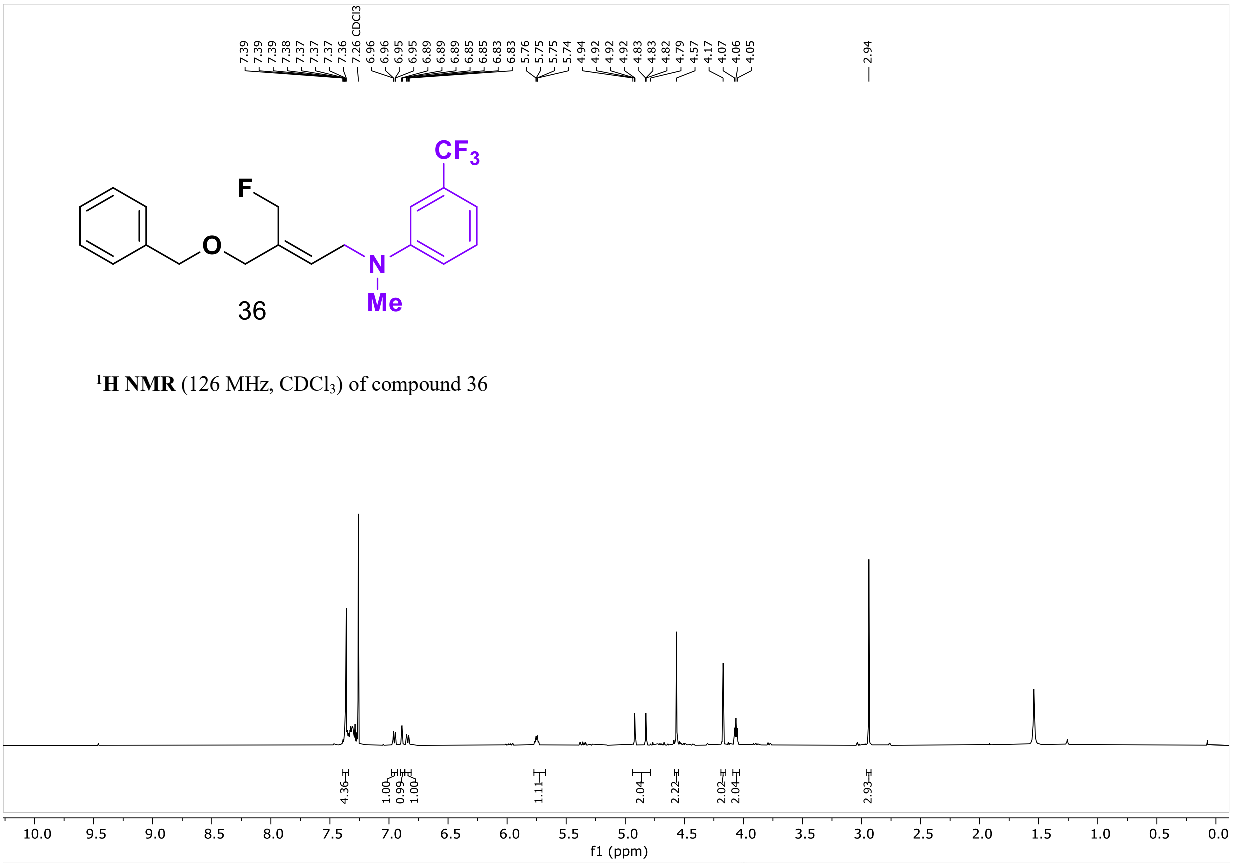

^{1}H NMR (126 MHz, $CDCl_3$) of compound 36

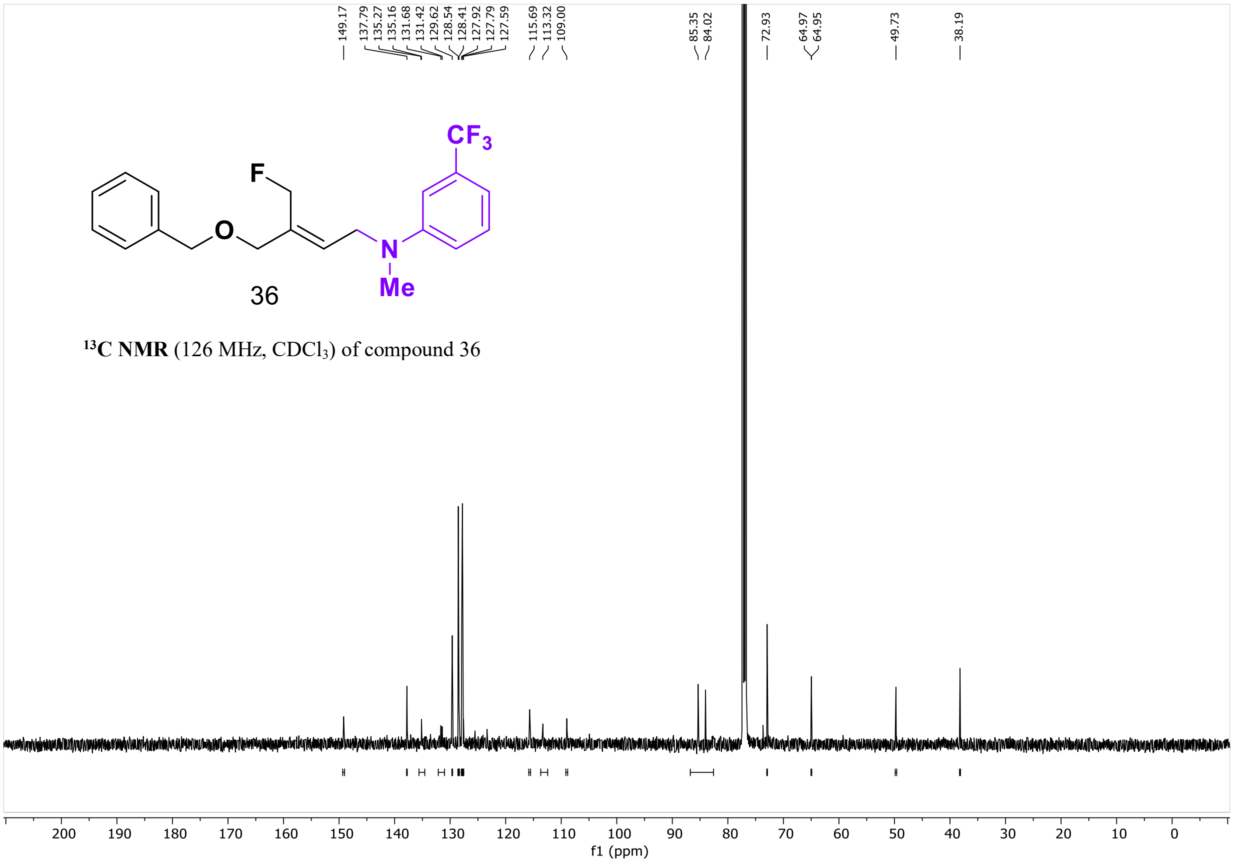

^{13}C NMR (126 MHz, $CDCl_3$) of compound 36

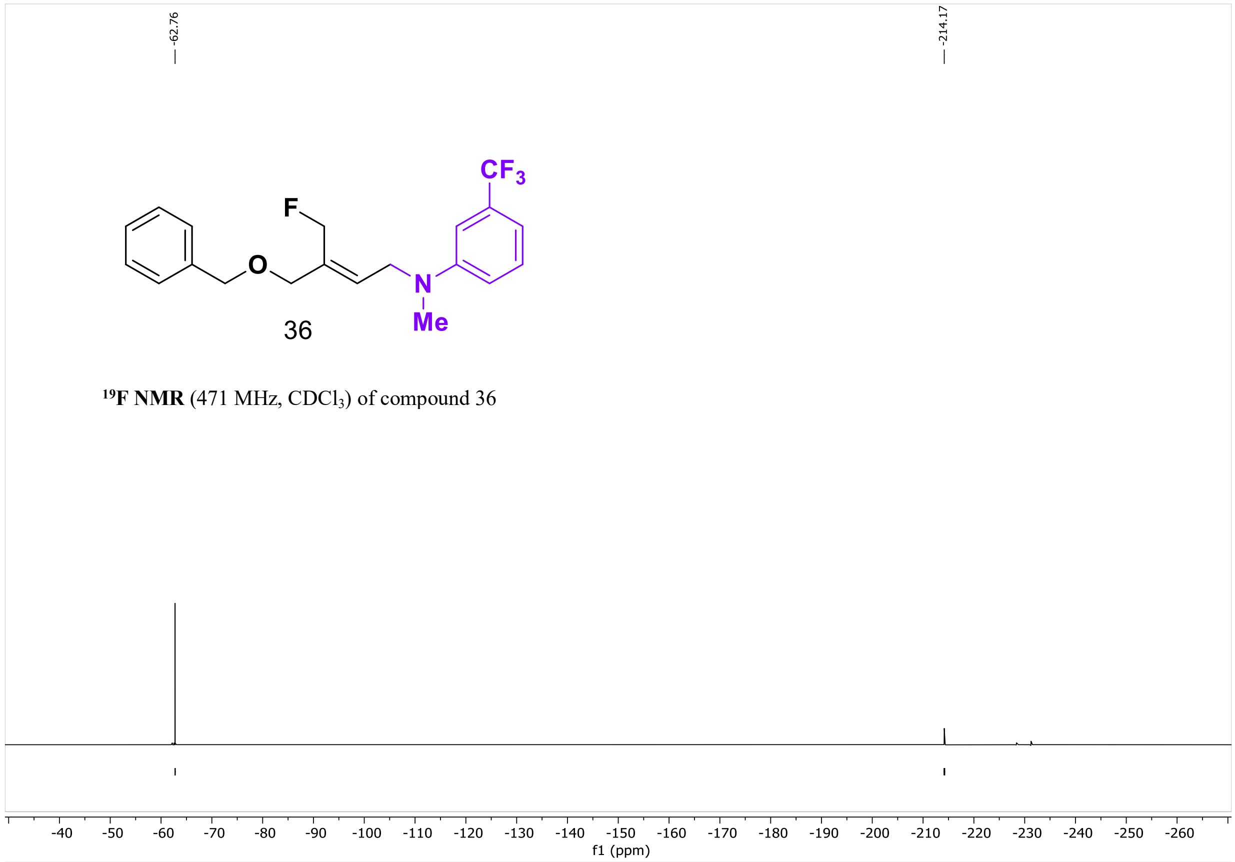

^{19}**F NMR** (471 MHz, $CDCl_3$) of compound 36

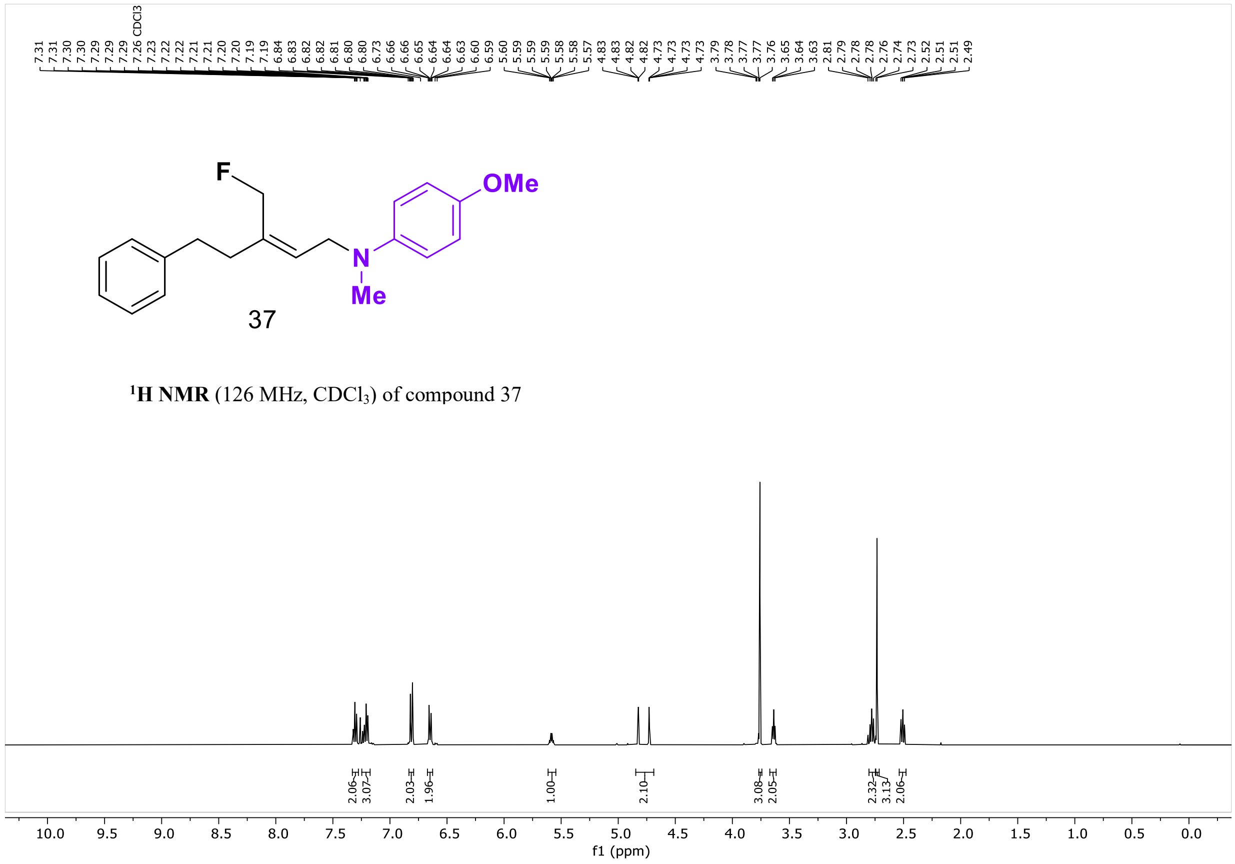

^{1}**H NMR** (126 MHz, $CDCl_3$) of compound 37

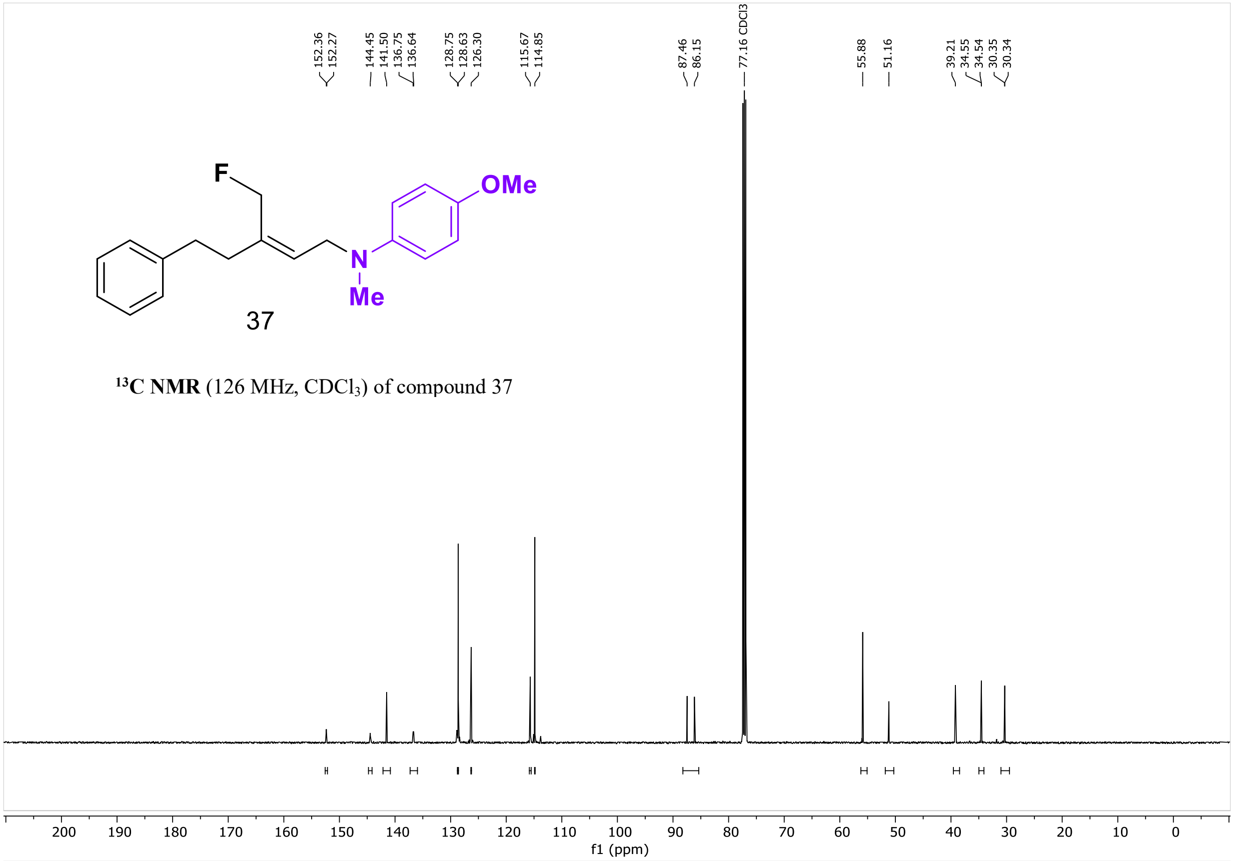

^{13}C **NMR** (126 MHz, $CDCl_3$) of compound 37

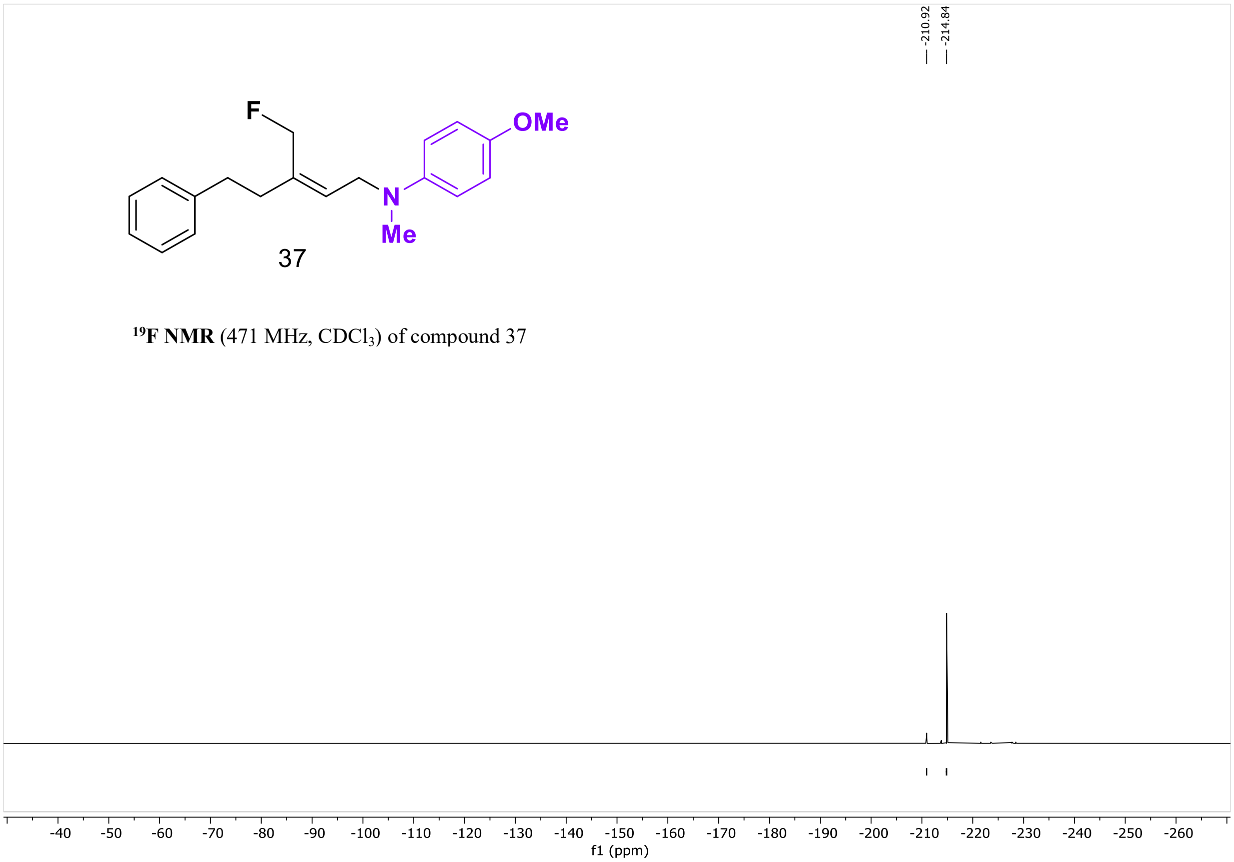

^{19}F **NMR** (471 MHz, $CDCl_3$) of compound 37

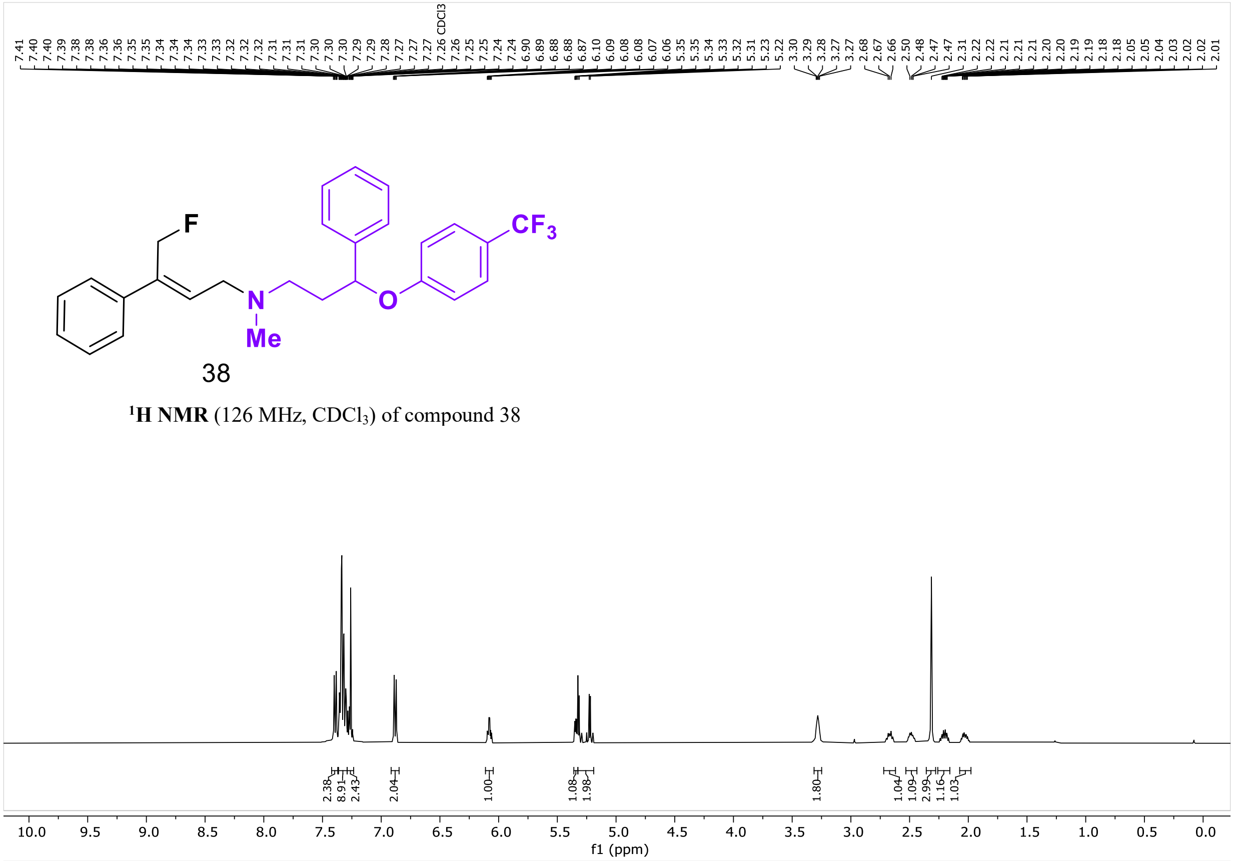

^{1}H NMR (126 MHz, $CDCl_3$) of compound 38

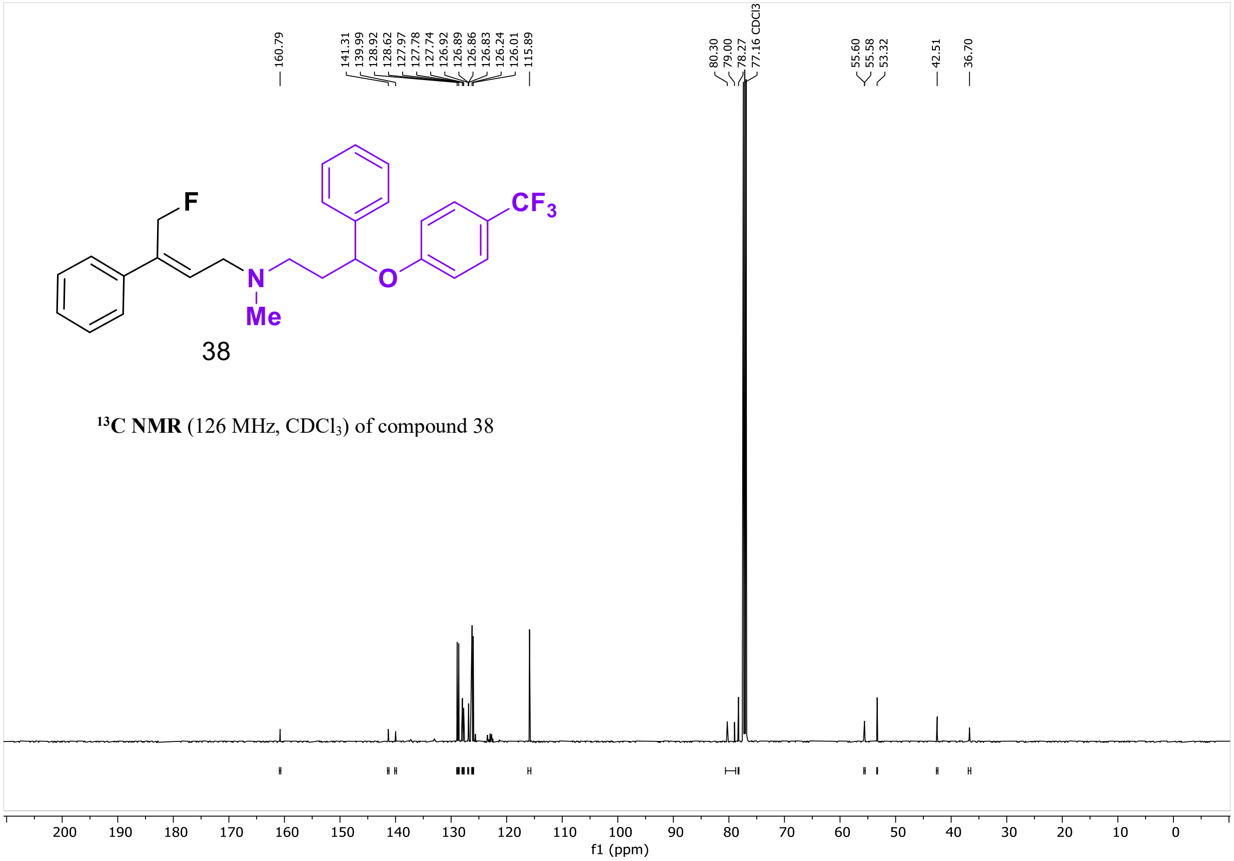

^{13}C NMR (126 MHz, $CDCl_3$) of compound 38

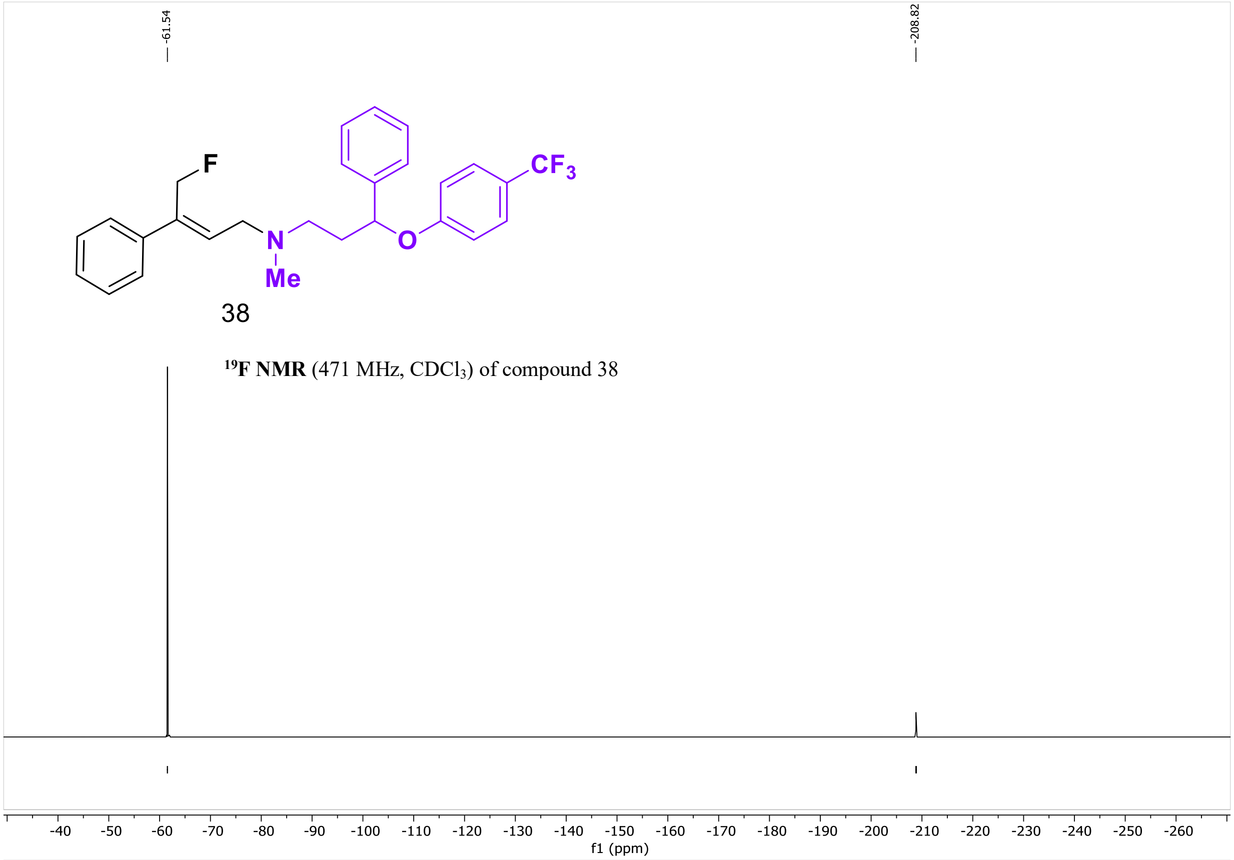

^{19}F NMR (471 MHz, $CDCl_3$) of compound 38

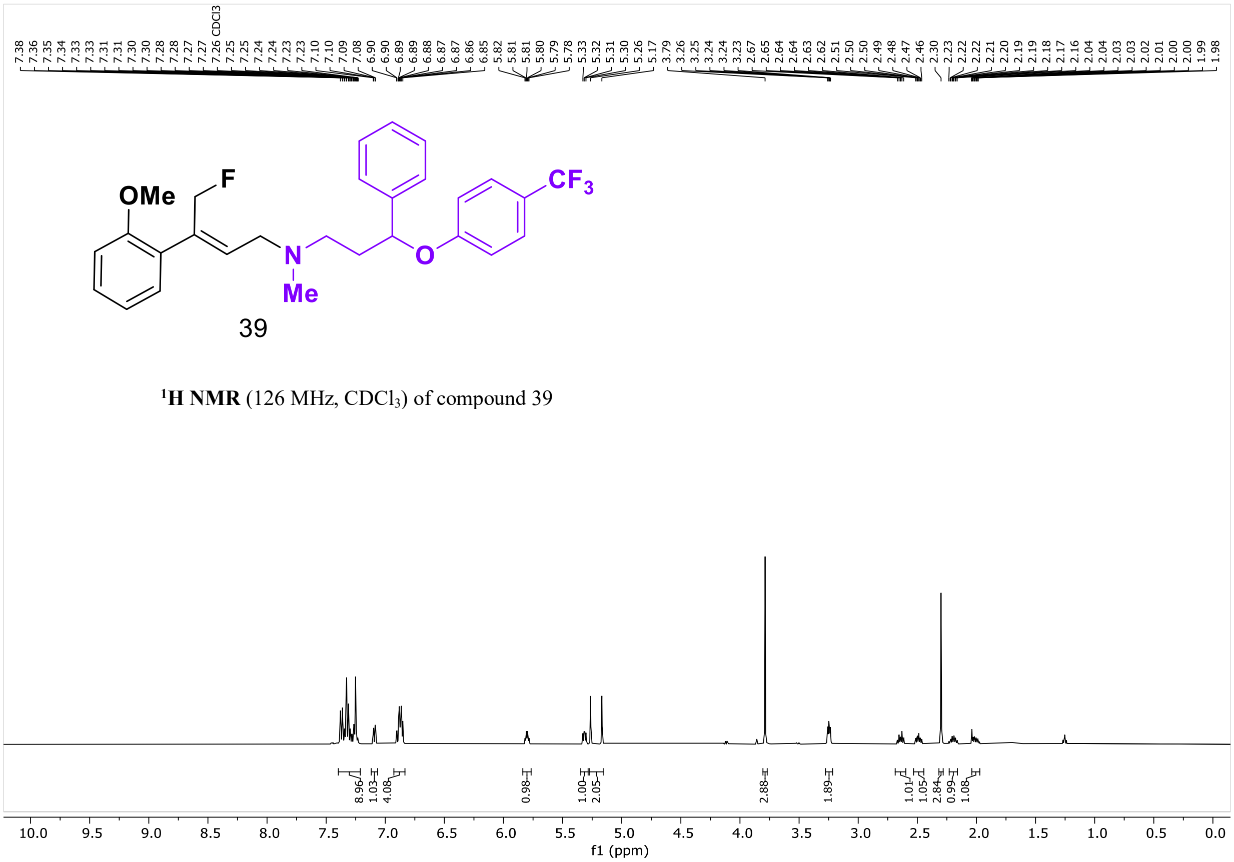

^{1}H NMR (126 MHz, $CDCl_3$) of compound 39

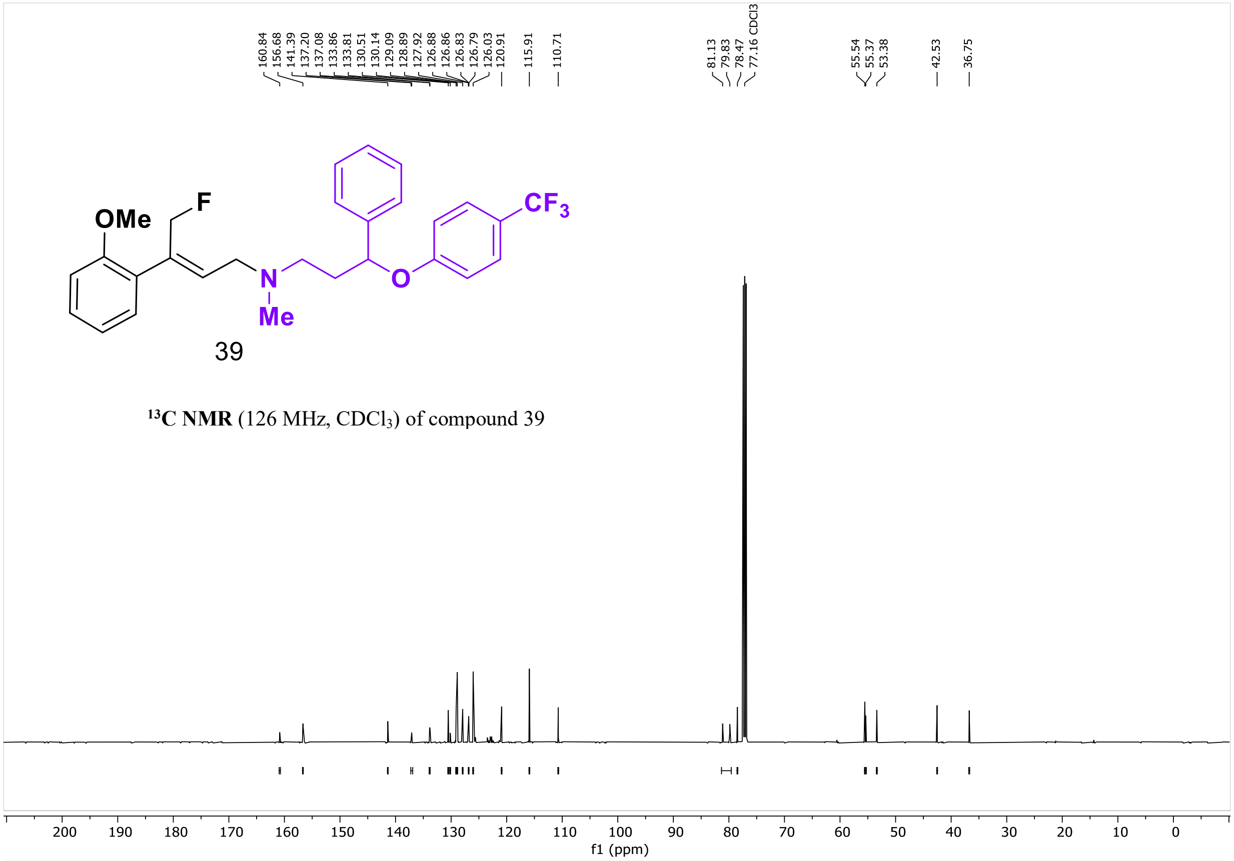

^{13}C **NMR** (126 MHz, $CDCl_3$) of compound 39

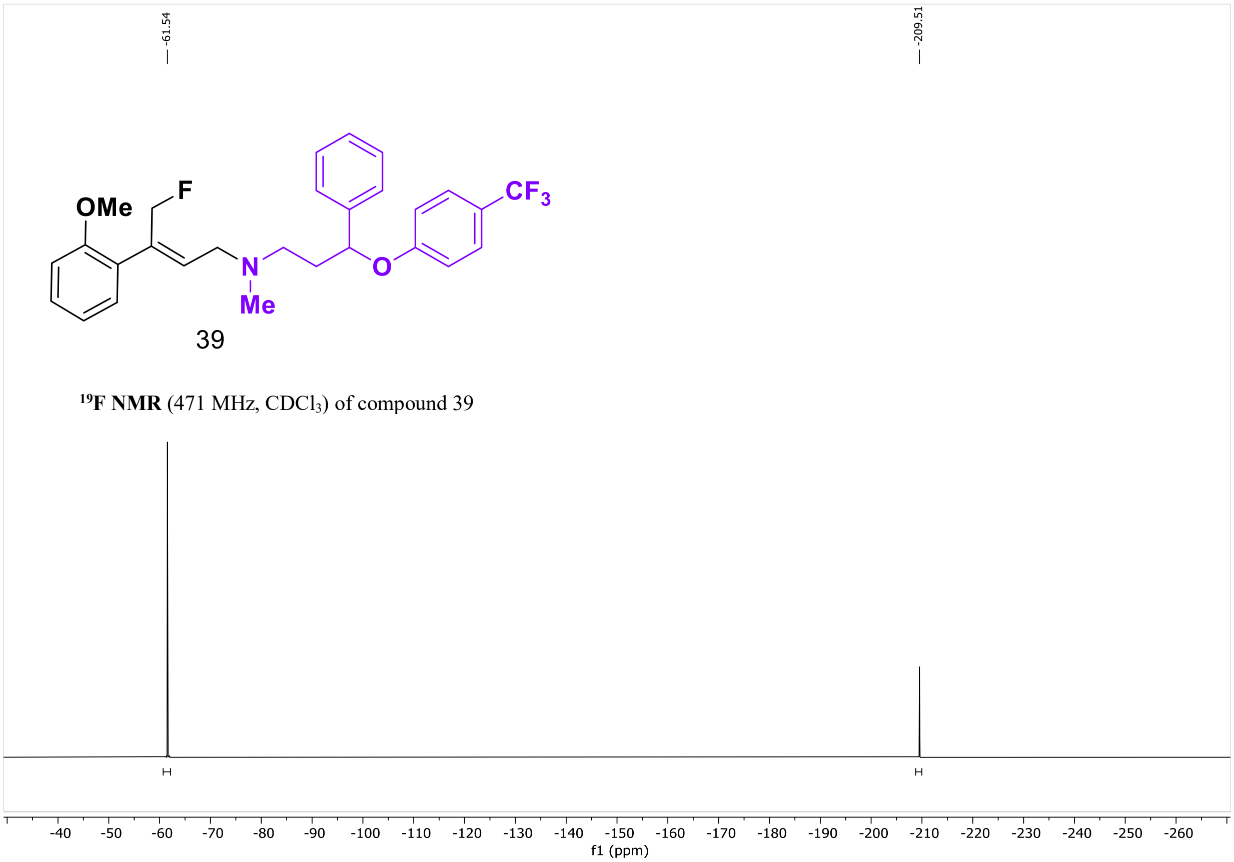

^{19}F **NMR** (471 MHz, $CDCl_3$) of compound 39

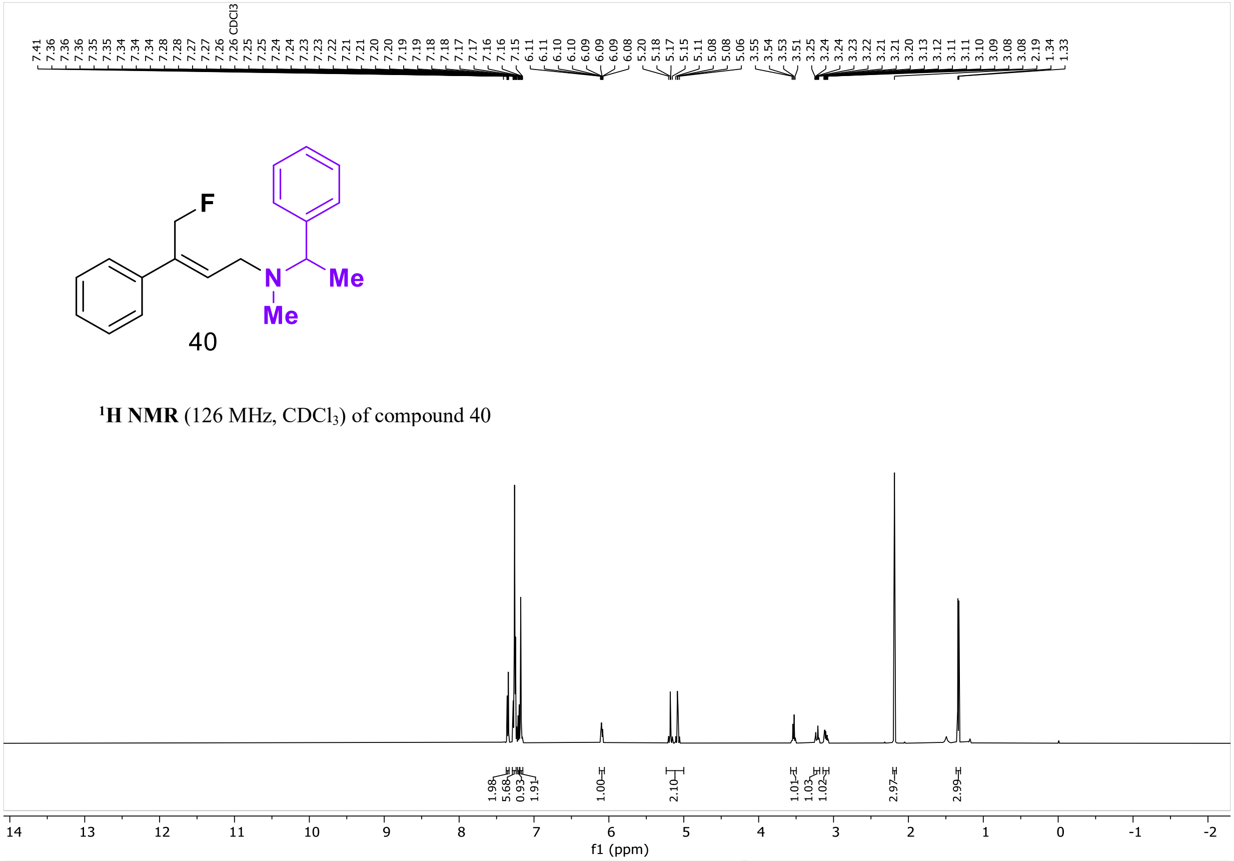

¹H NMR (126 MHz, $CDCl_3$) of compound 40

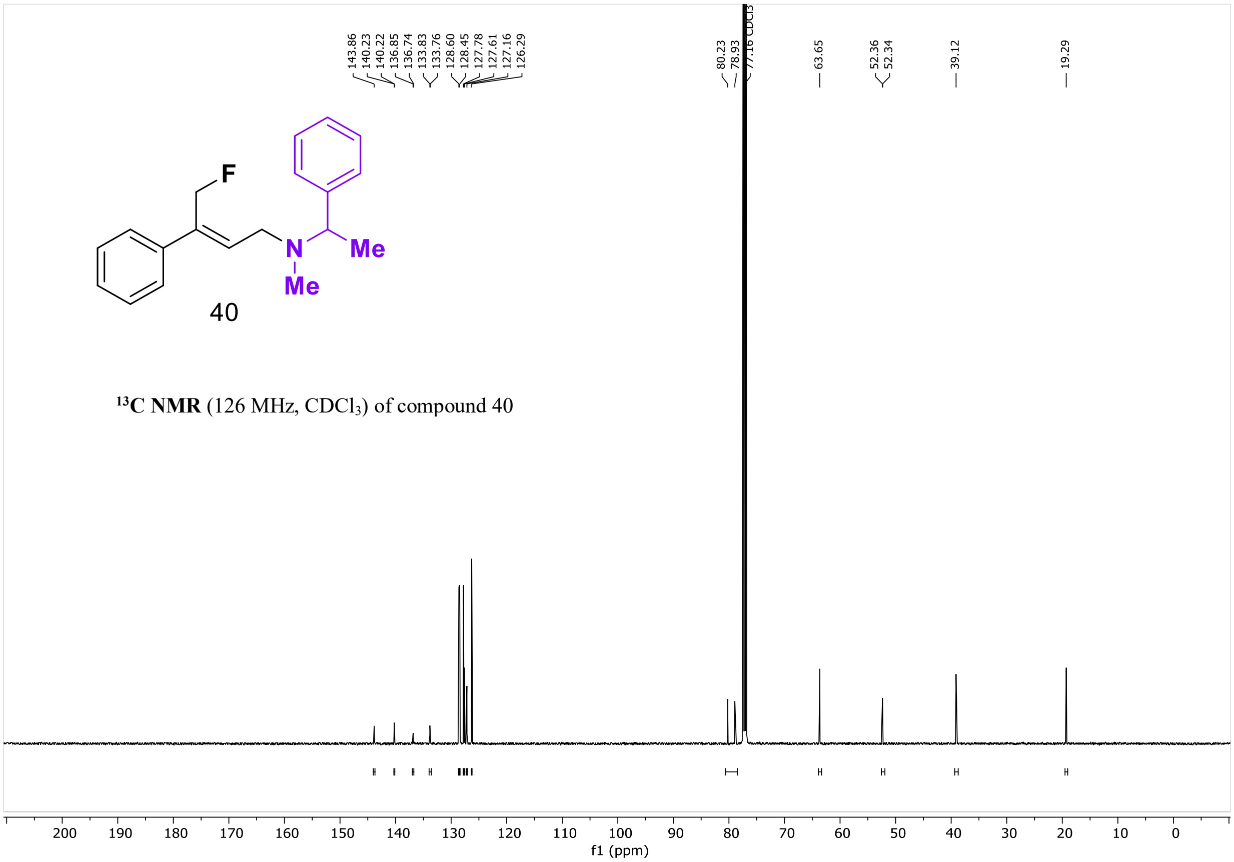

¹³C NMR (126 MHz, $CDCl_3$) of compound 40

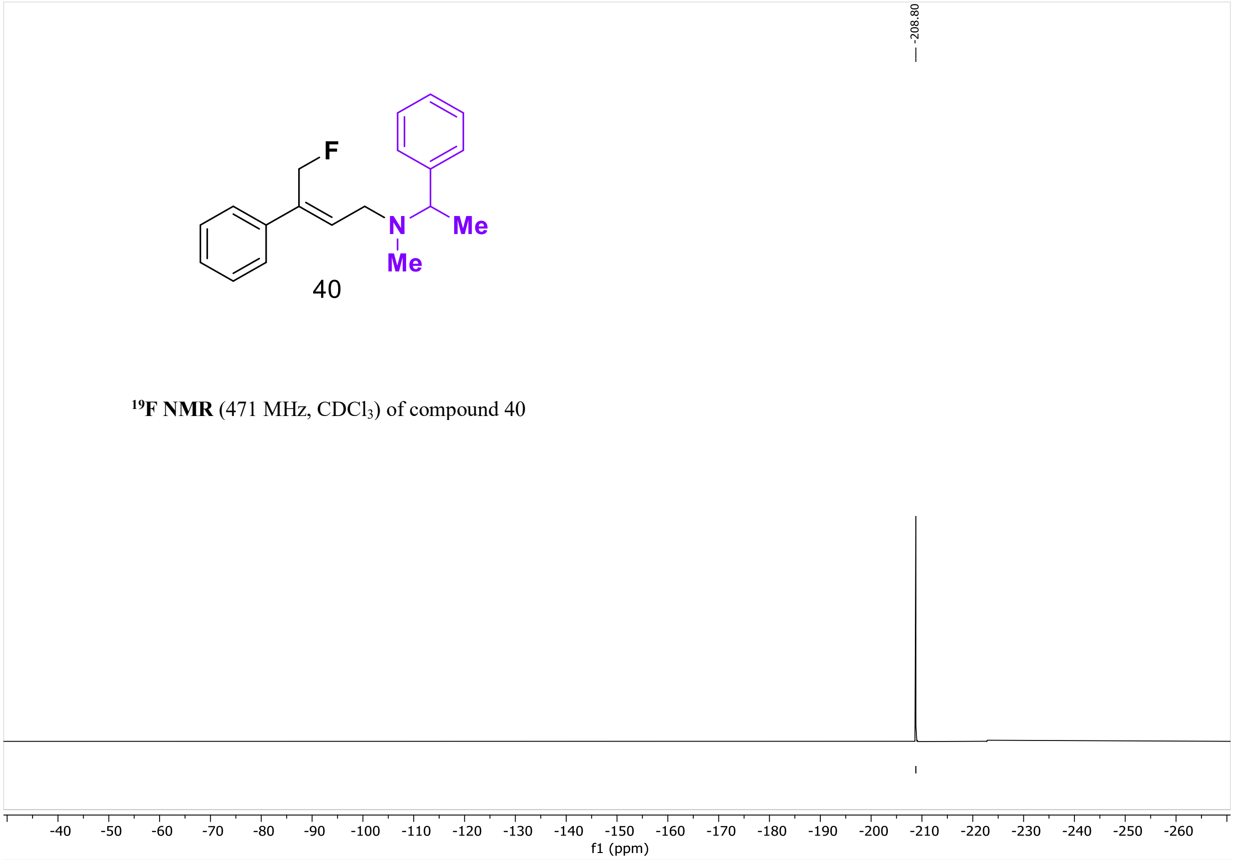

^{19}F NMR (471 MHz, $CDCl_3$) of compound 40

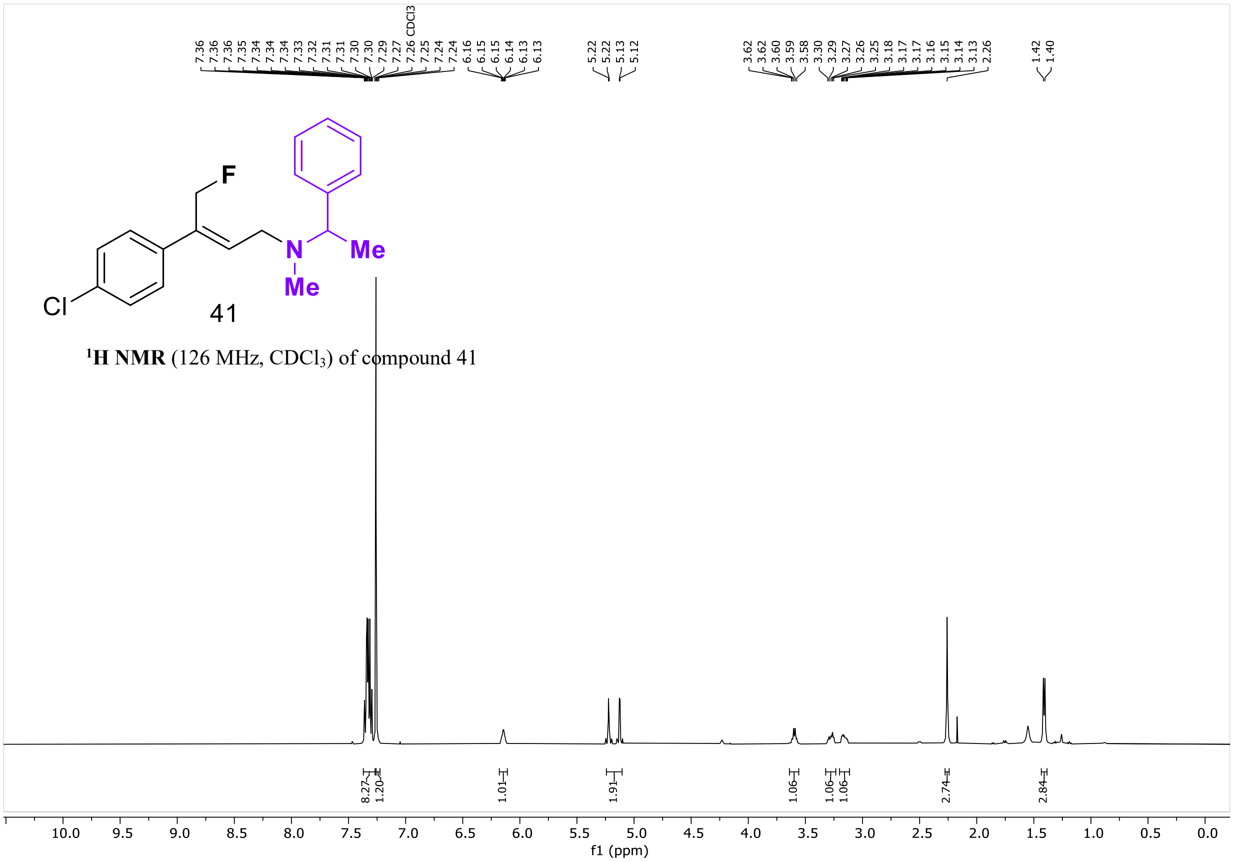

^{1}H NMR (126 MHz, $CDCl_3$) of compound 41

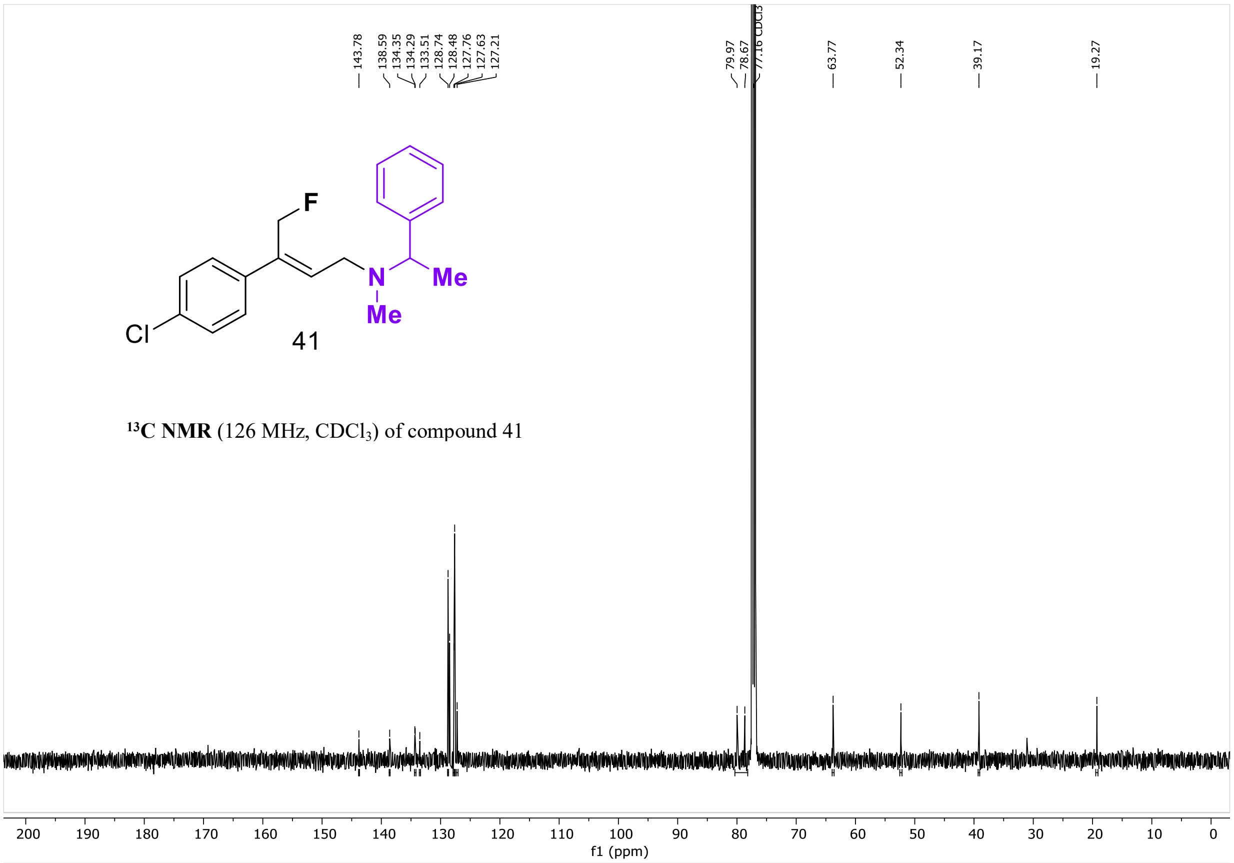

^{13}C NMR (126 MHz, $CDCl_3$) of compound 41

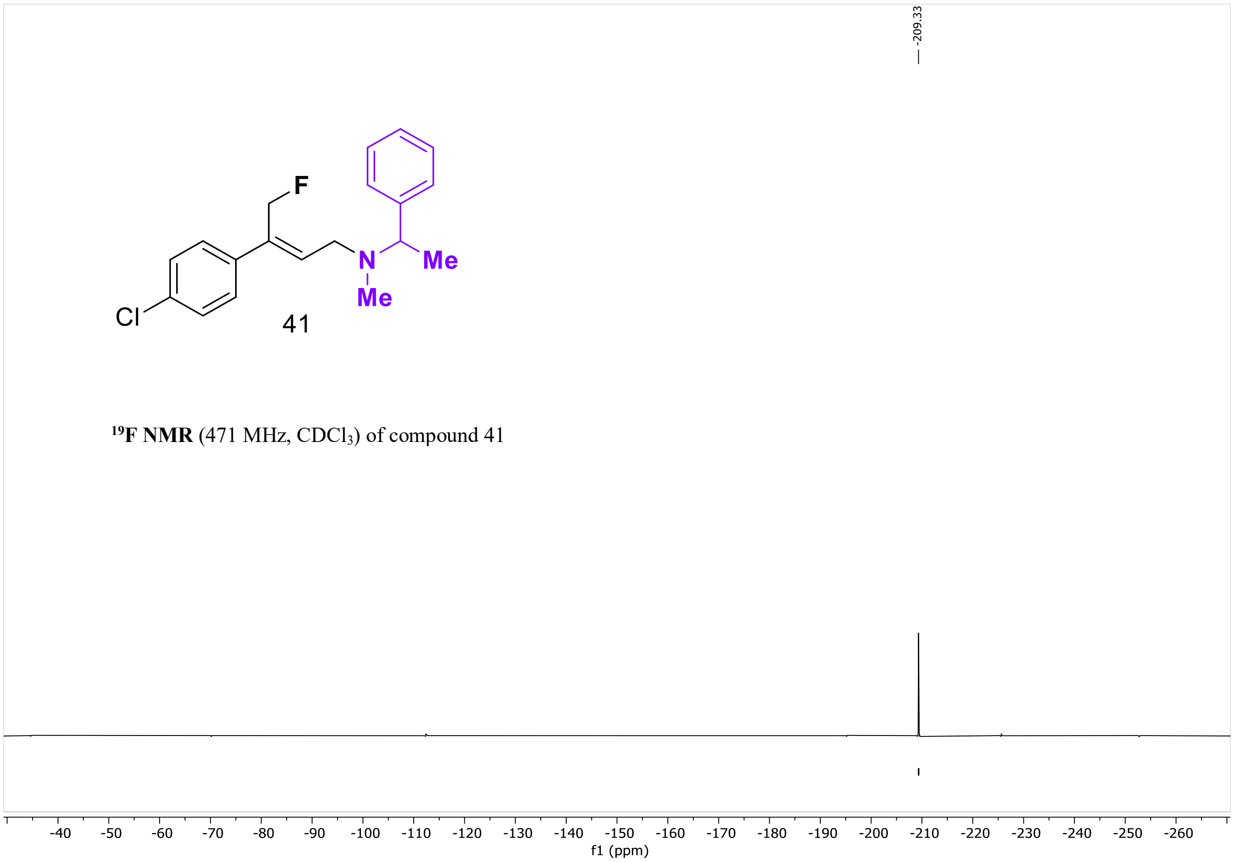

^{19}F NMR (471 MHz, $CDCl_3$) of compound 41

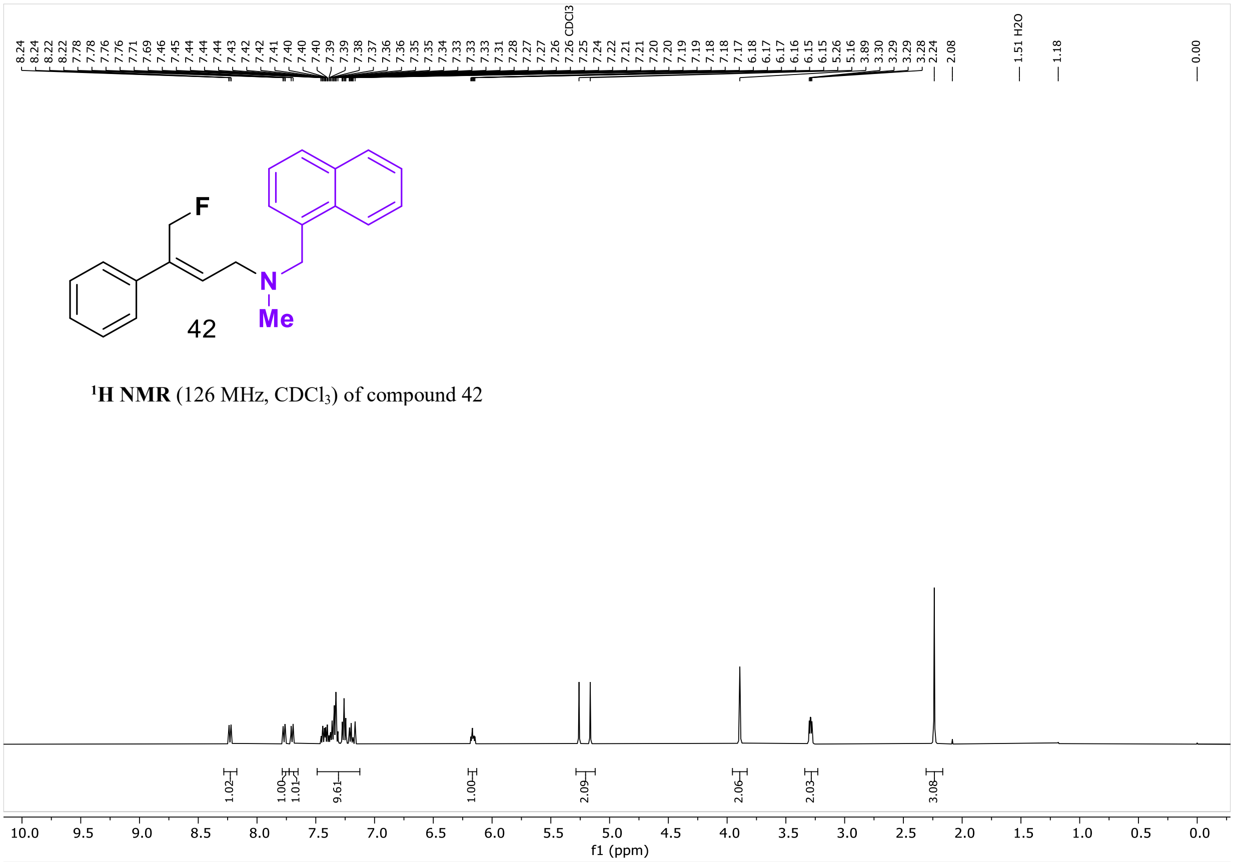

^{1}H NMR (126 MHz, $CDCl_3$) of compound 42

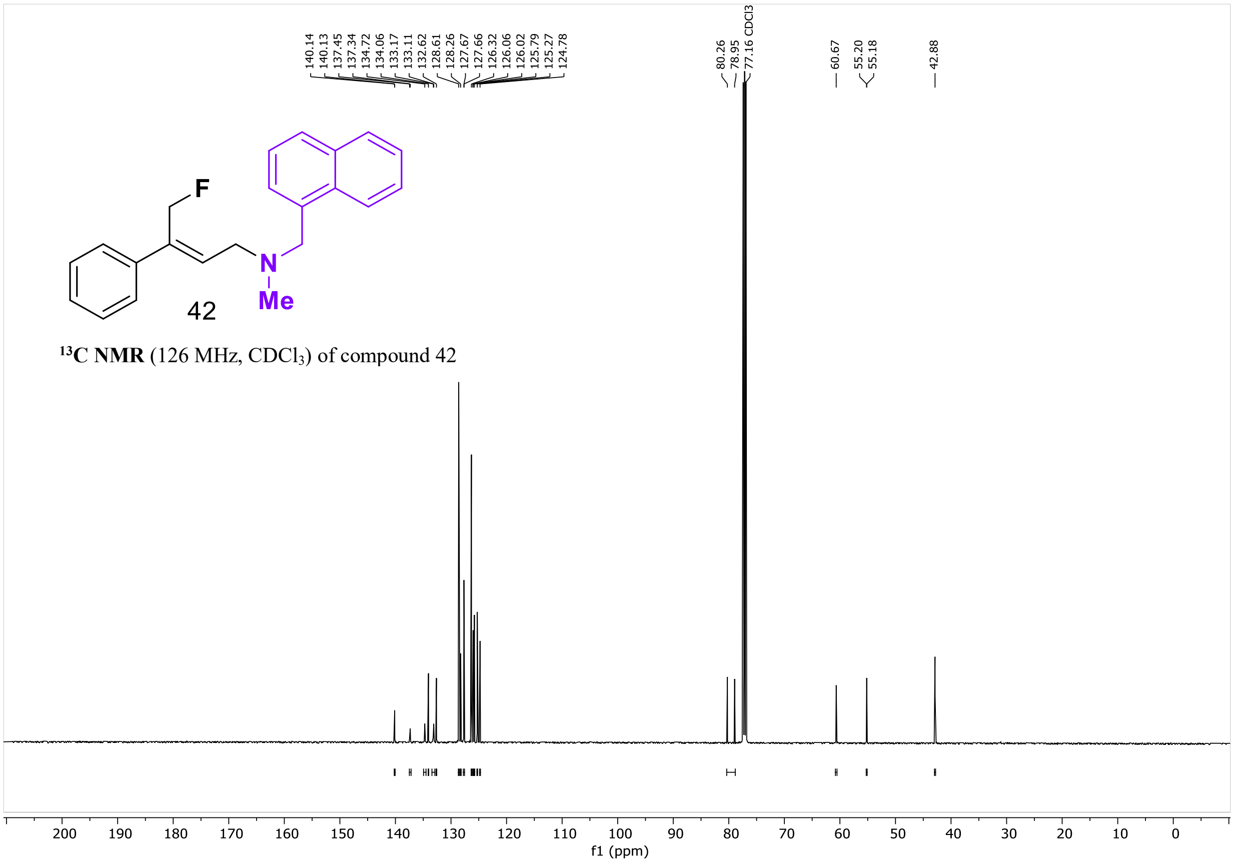

^{13}C NMR (126 MHz, $CDCl_3$) of compound 42

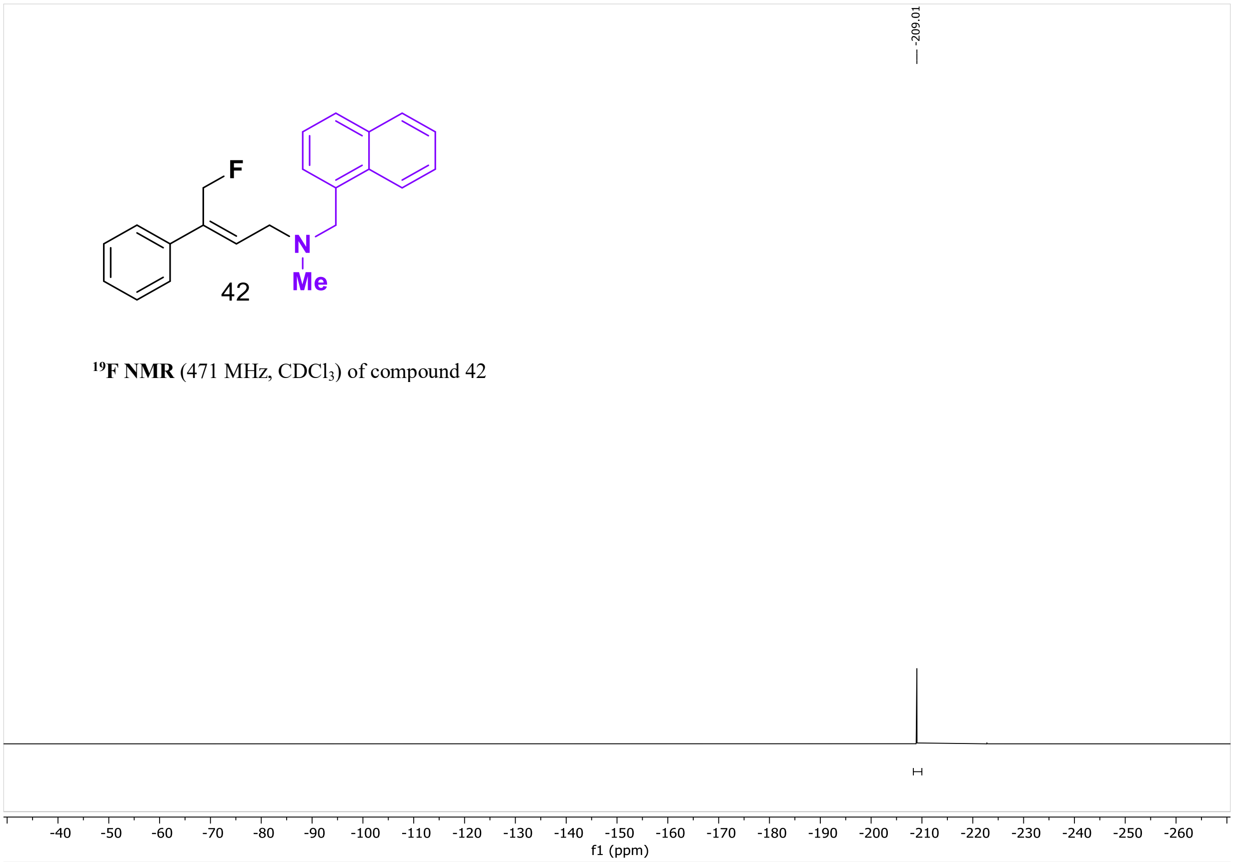

^{19}**F NMR** (471 MHz, $CDCl_3$) of compound 42

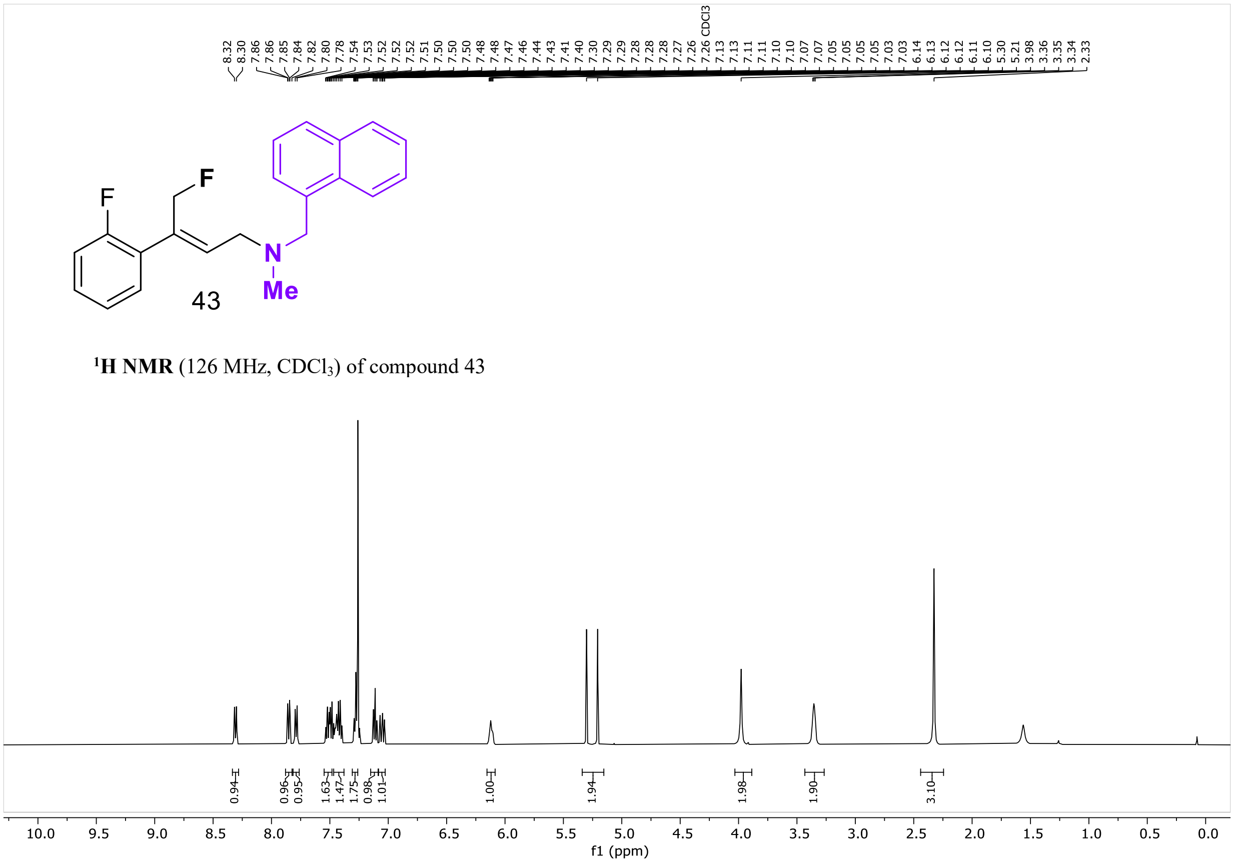

^{1}**H NMR** (126 MHz, $CDCl_3$) of compound 43

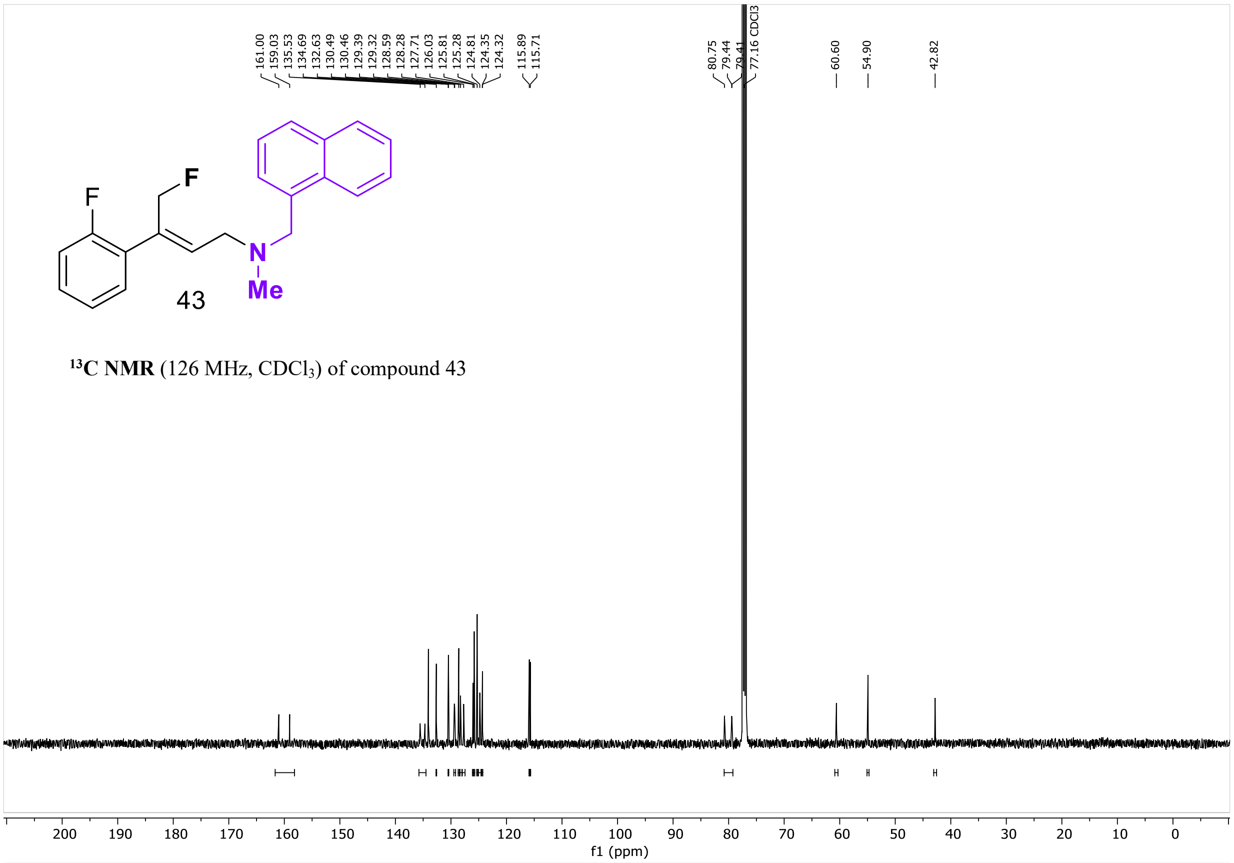

^{13}C NMR (126 MHz, $CDCl_3$) of compound 43

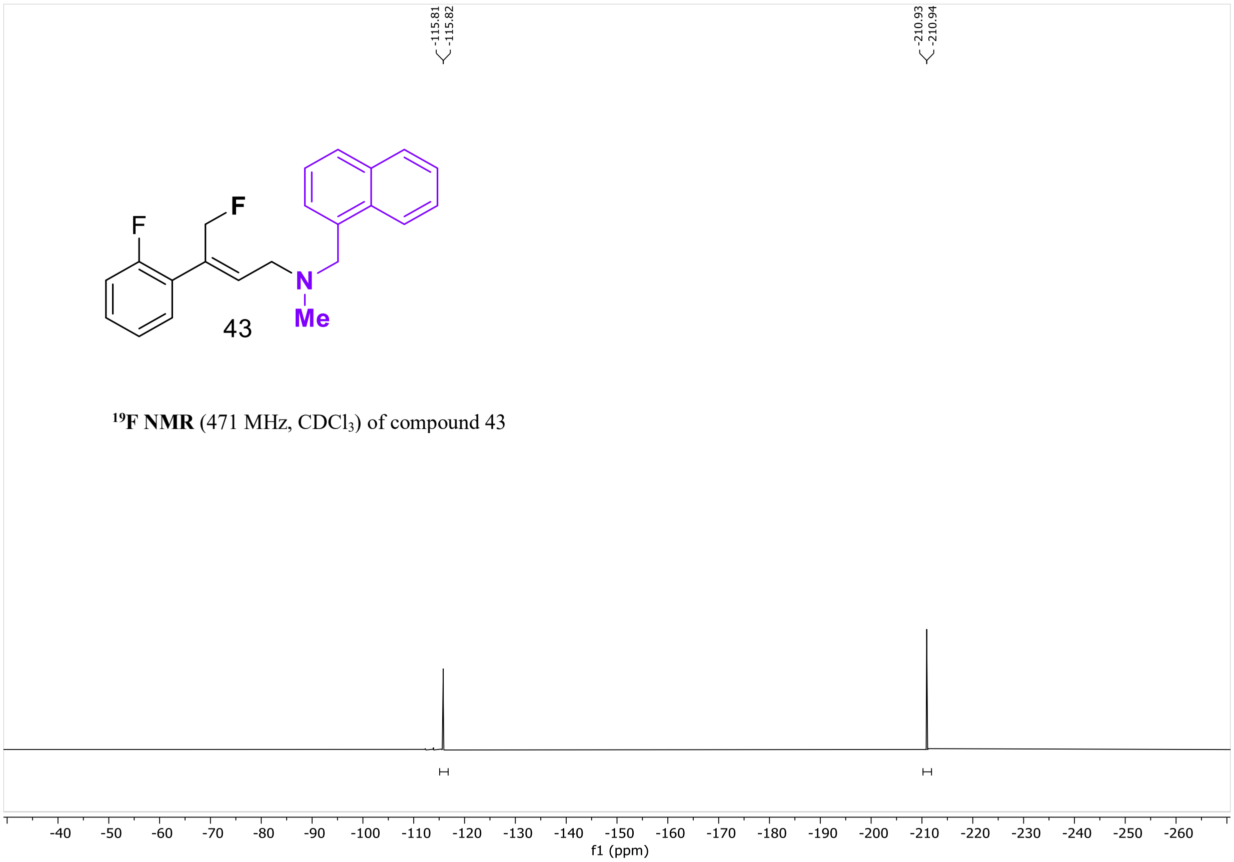

^{19}F NMR (471 MHz, $CDCl_3$) of compound 43

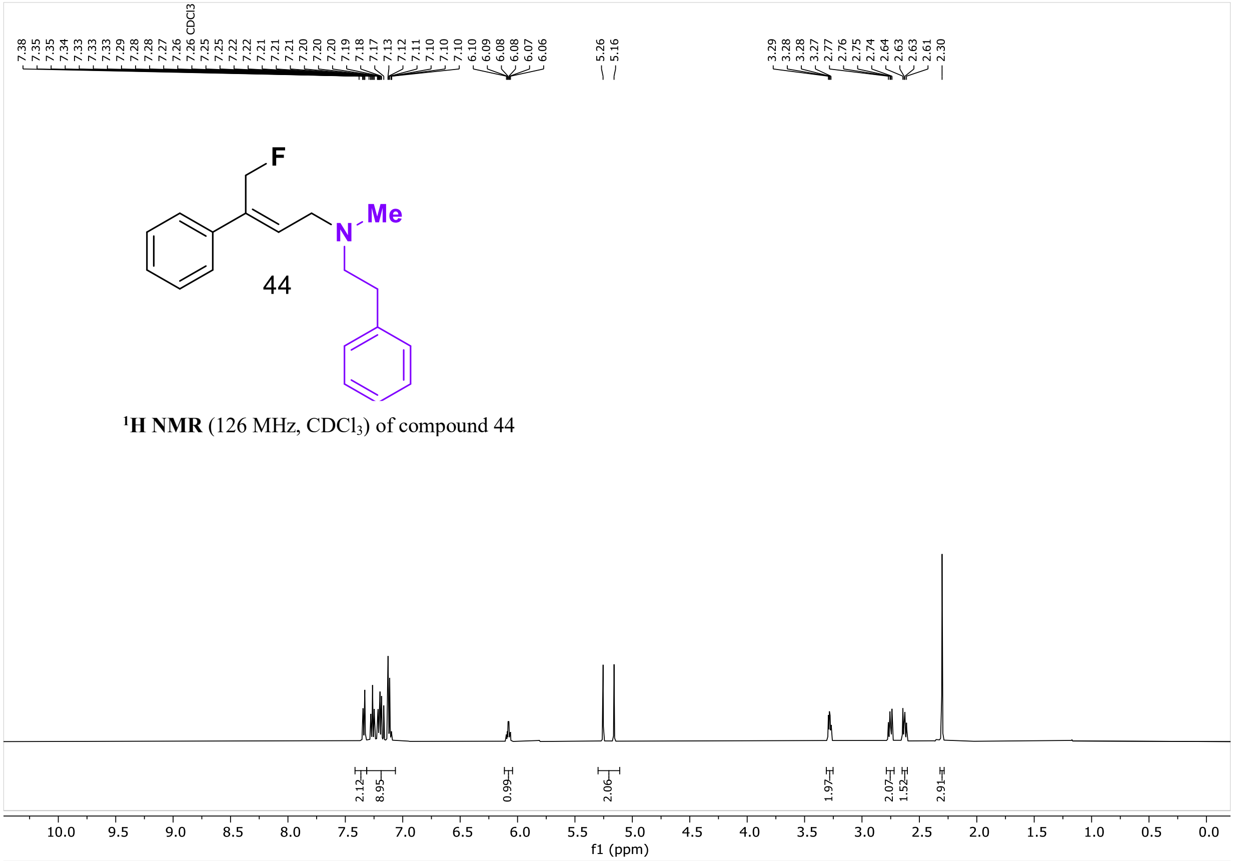

^{1}H NMR (126 MHz, $CDCl_3$) of compound 44

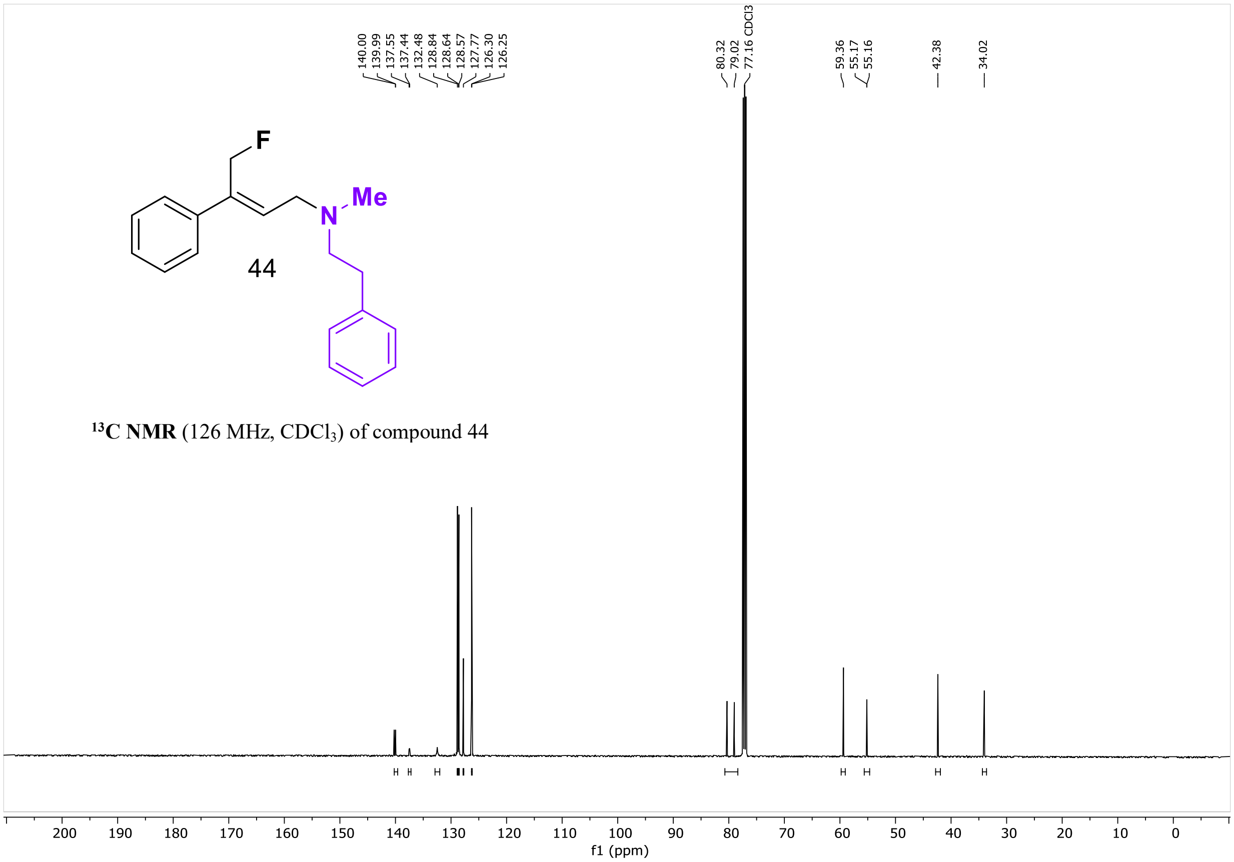

^{13}C NMR (126 MHz, $CDCl_3$) of compound 44

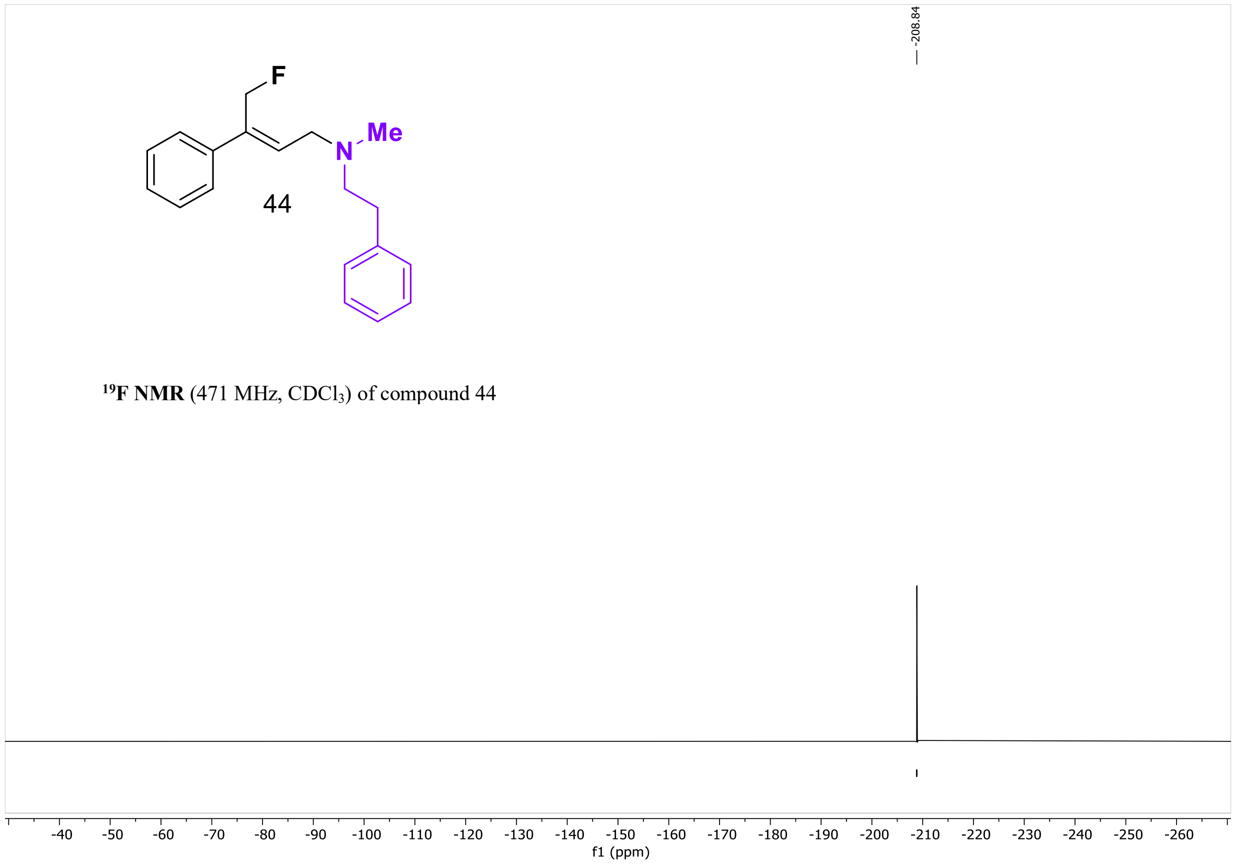

^{19}F NMR (471 MHz, $CDCl_3$) of compound 44

NMR of Starting Materials

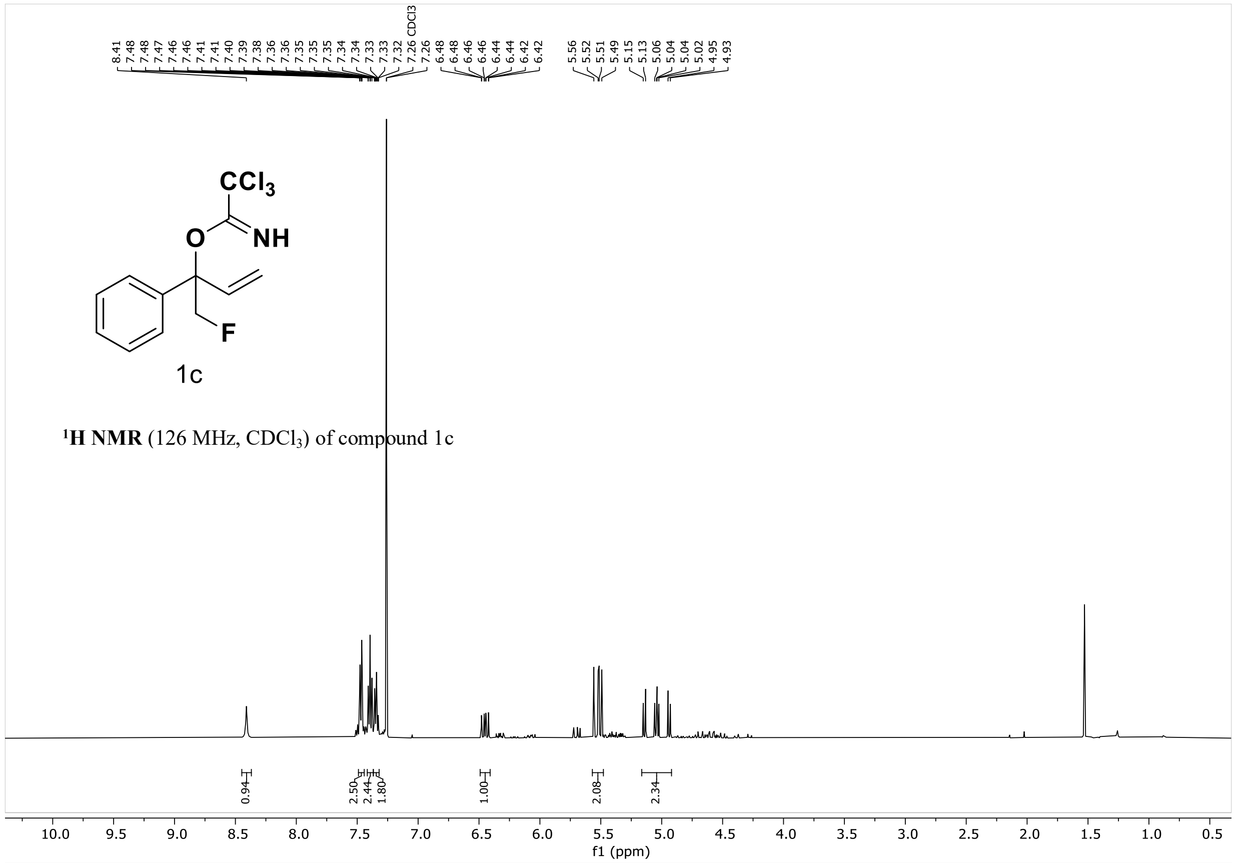

^{1}H NMR (126 MHz, $CDCl_3$) of compound 1c

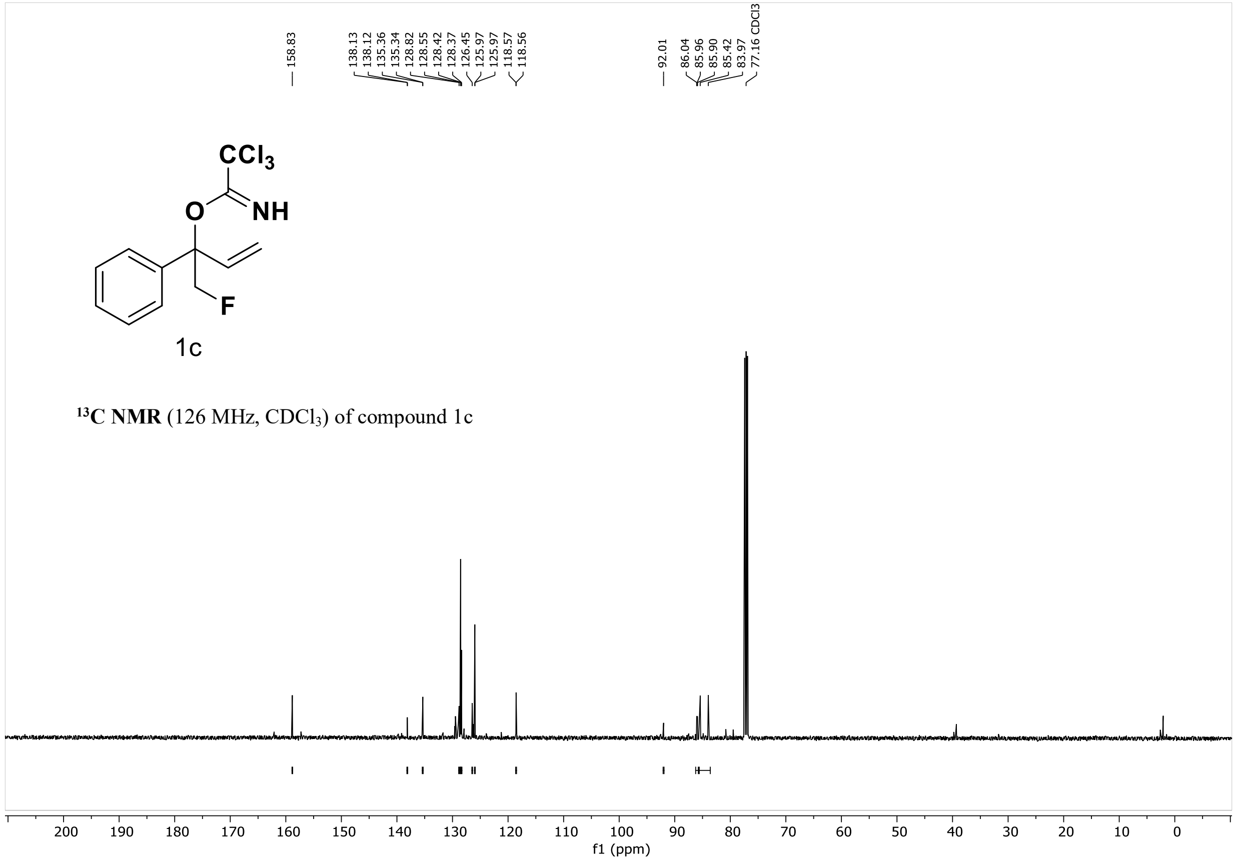

^{13}C NMR (126 MHz, $CDCl_3$) of compound 1c

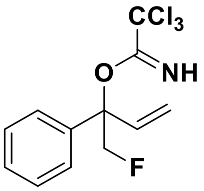

1c

^{19}F NMR (471 MHz, $CDCl_3$) of compound 1c

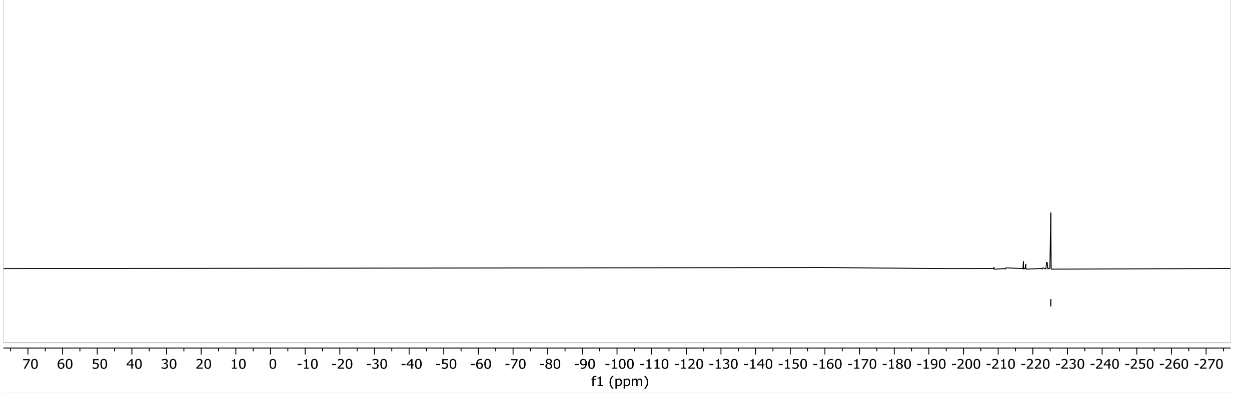

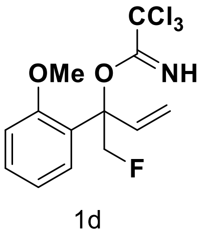

1d

^{1}H NMR (126 MHz, $CDCl_3$) of compound 1d

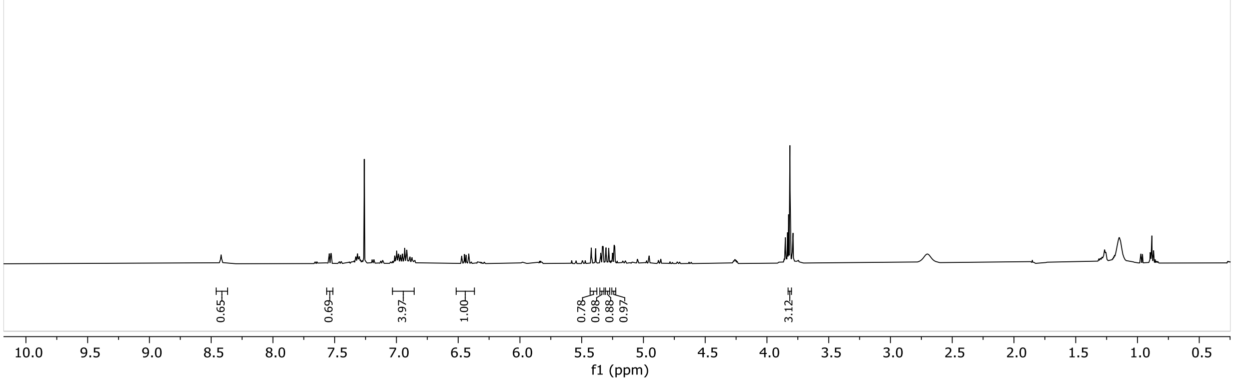

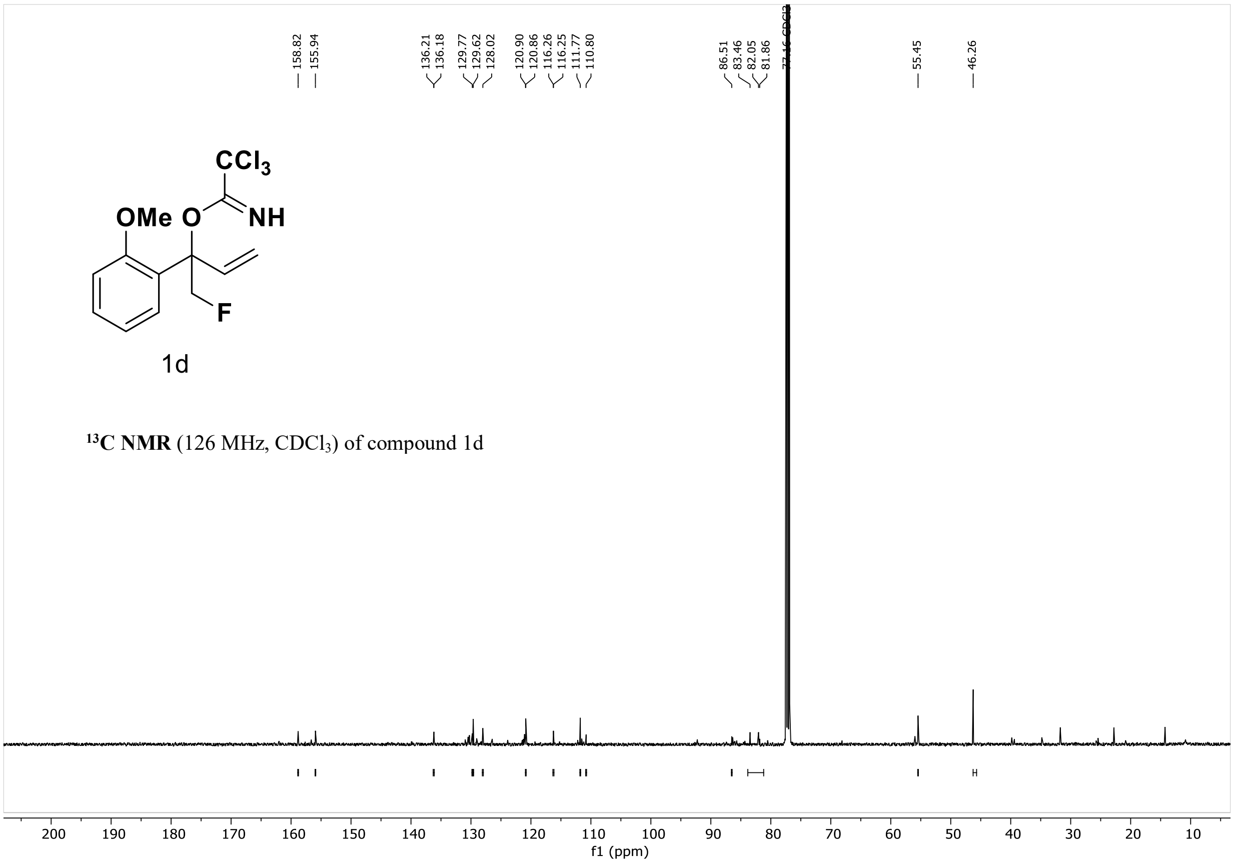

^{13}C NMR (126 MHz, $CDCl_3$) of compound 1d

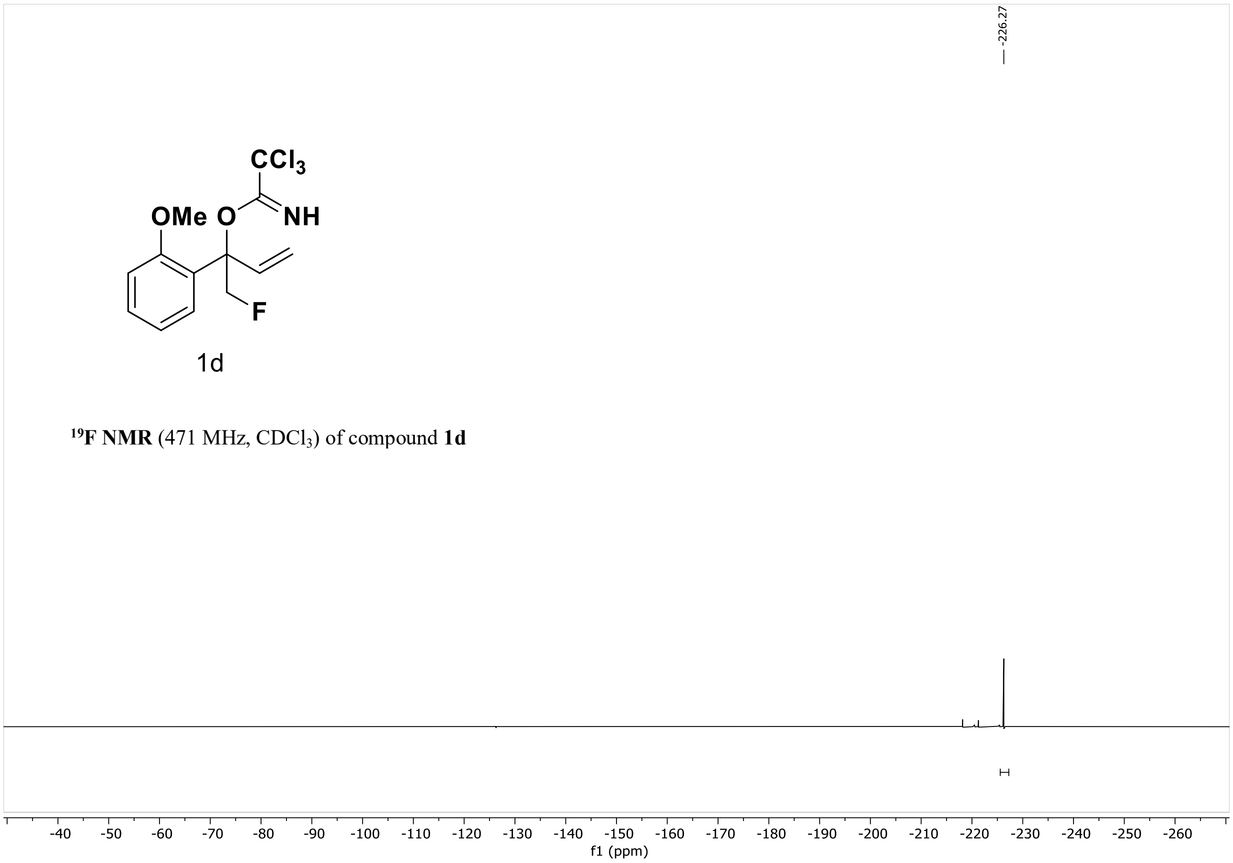

^{19}F NMR (471 MHz, $CDCl_3$) of compound **1d**

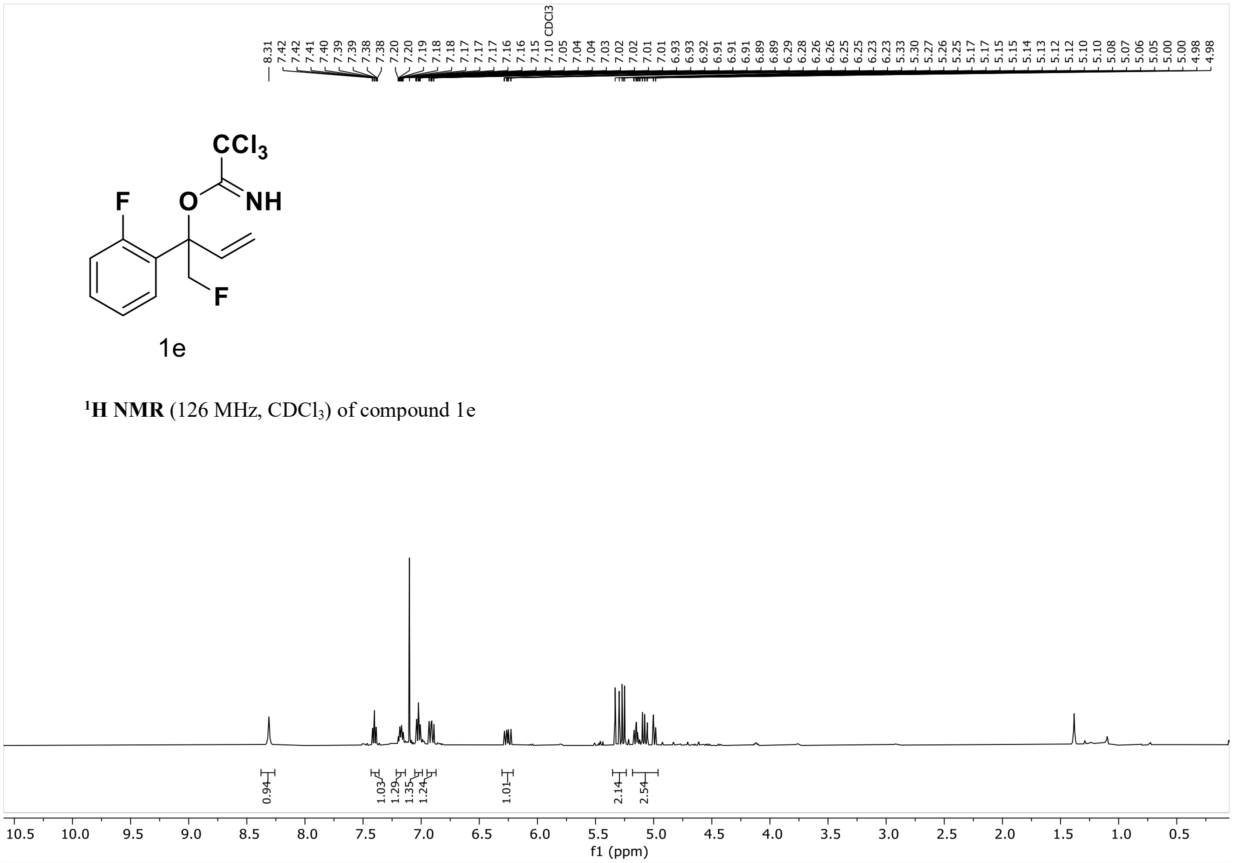

1e

^{1}H NMR (126 MHz, $CDCl_3$) of compound 1e

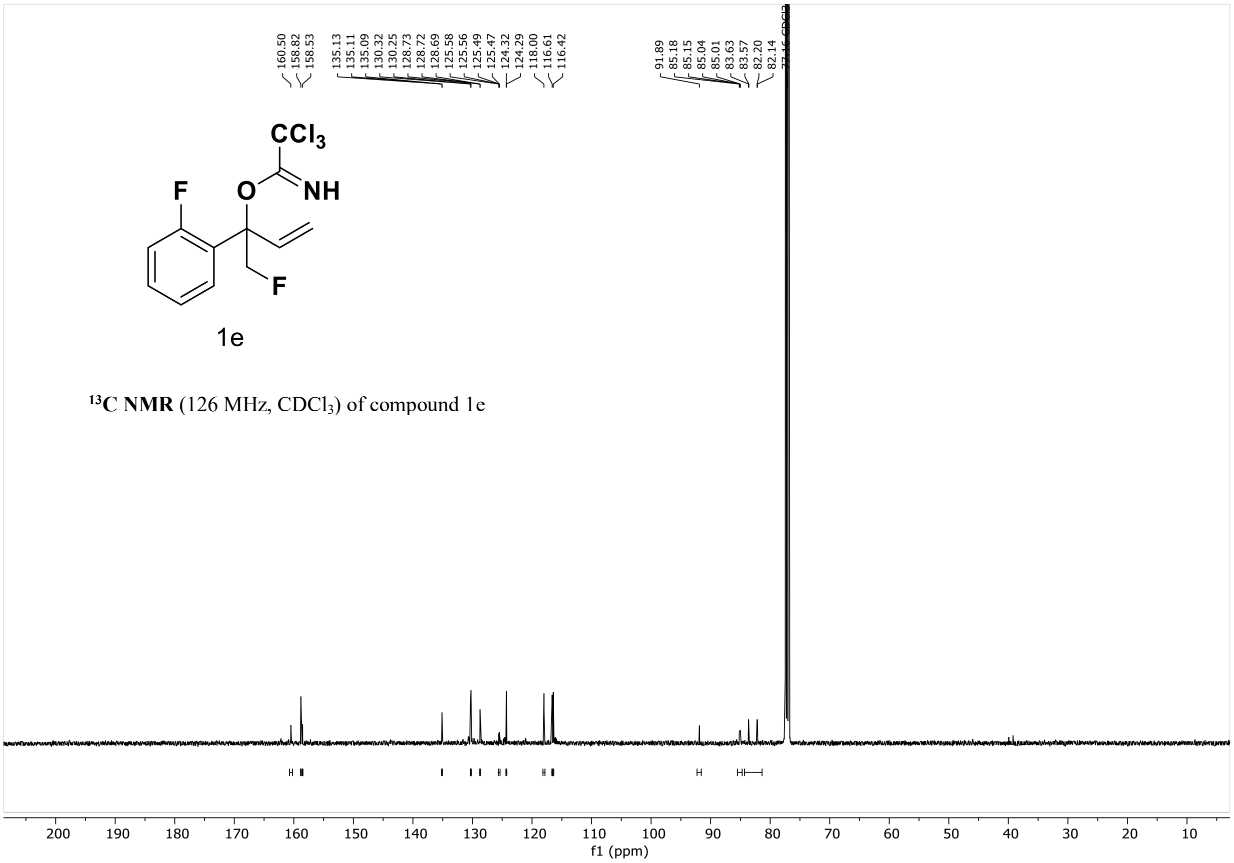

1e

^{13}C NMR (126 MHz, $CDCl_3$) of compound 1e

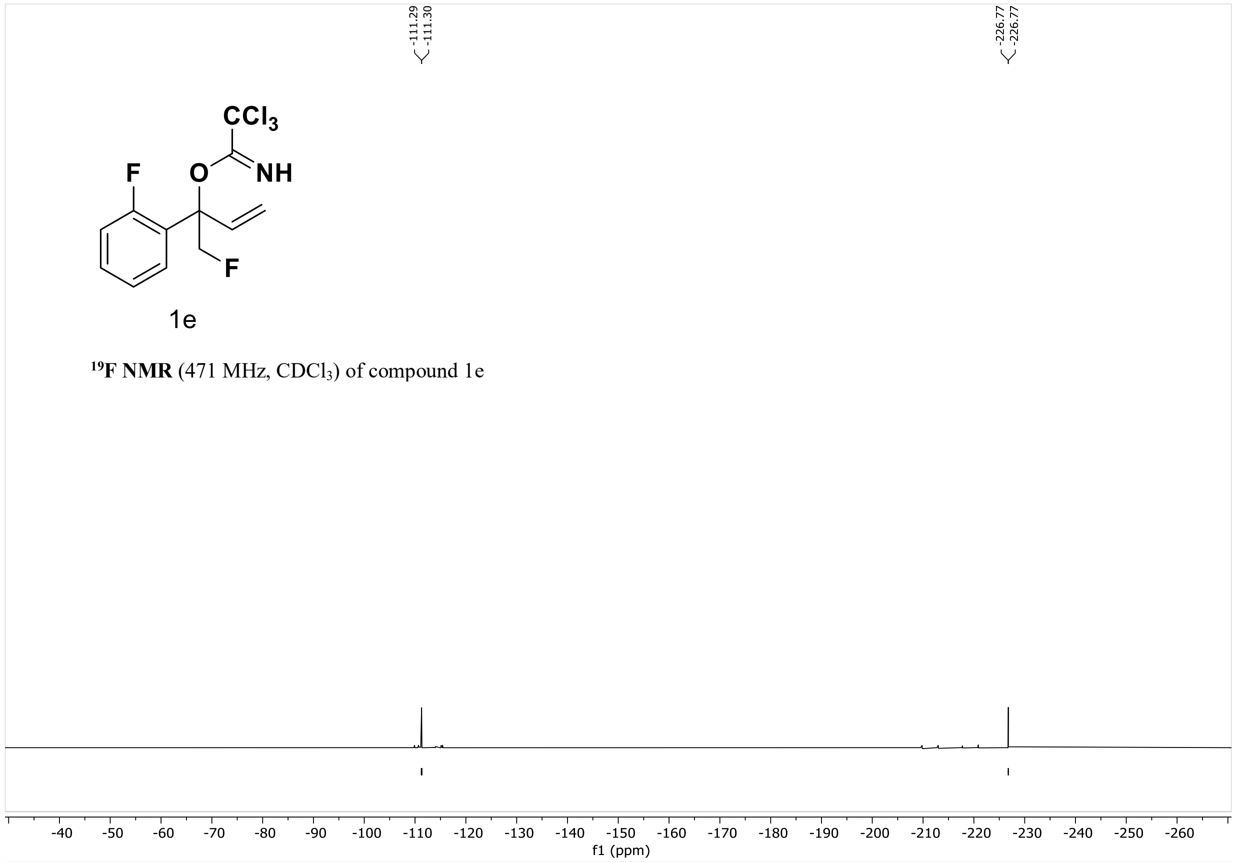

1e

^{19}F NMR (471 MHz, $CDCl_3$) of compound 1e

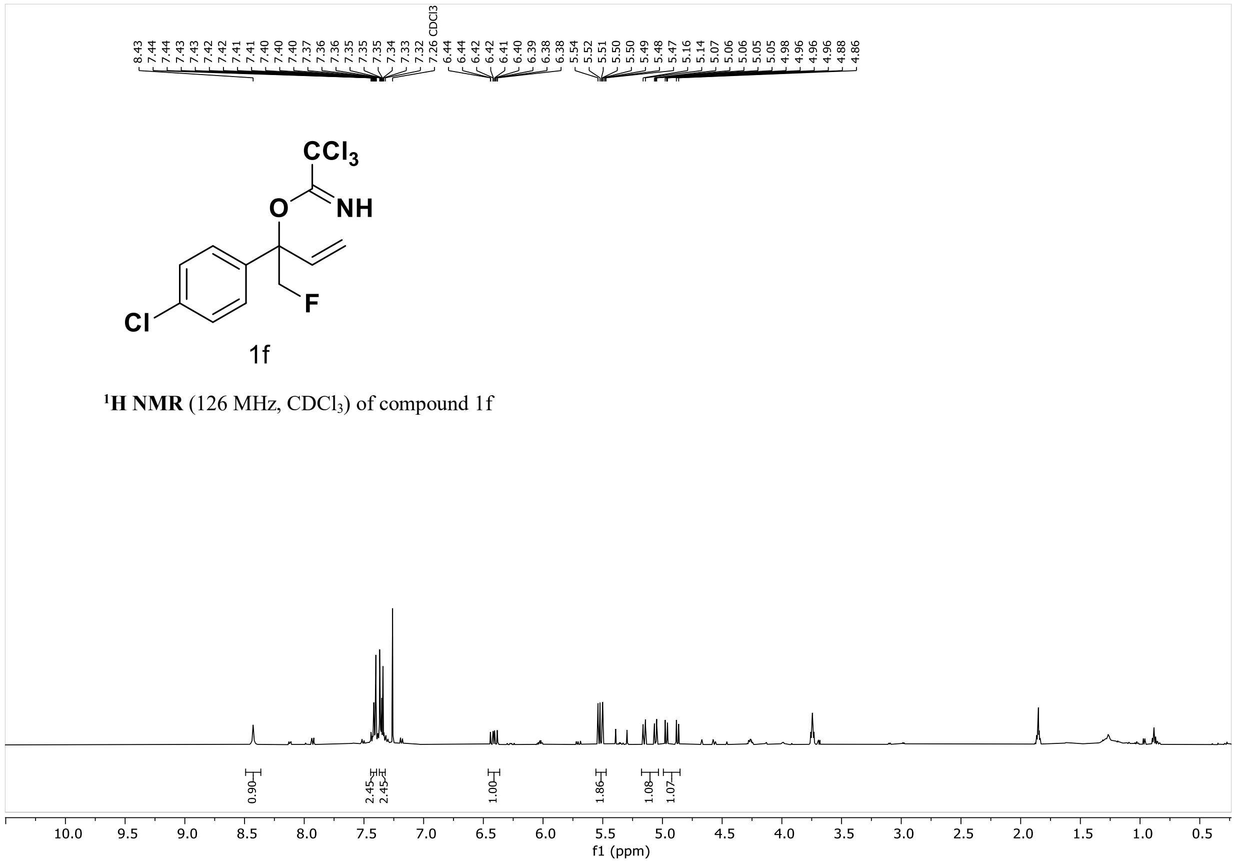

1f

^{1}H NMR (126 MHz, $CDCl_3$) of compound 1f

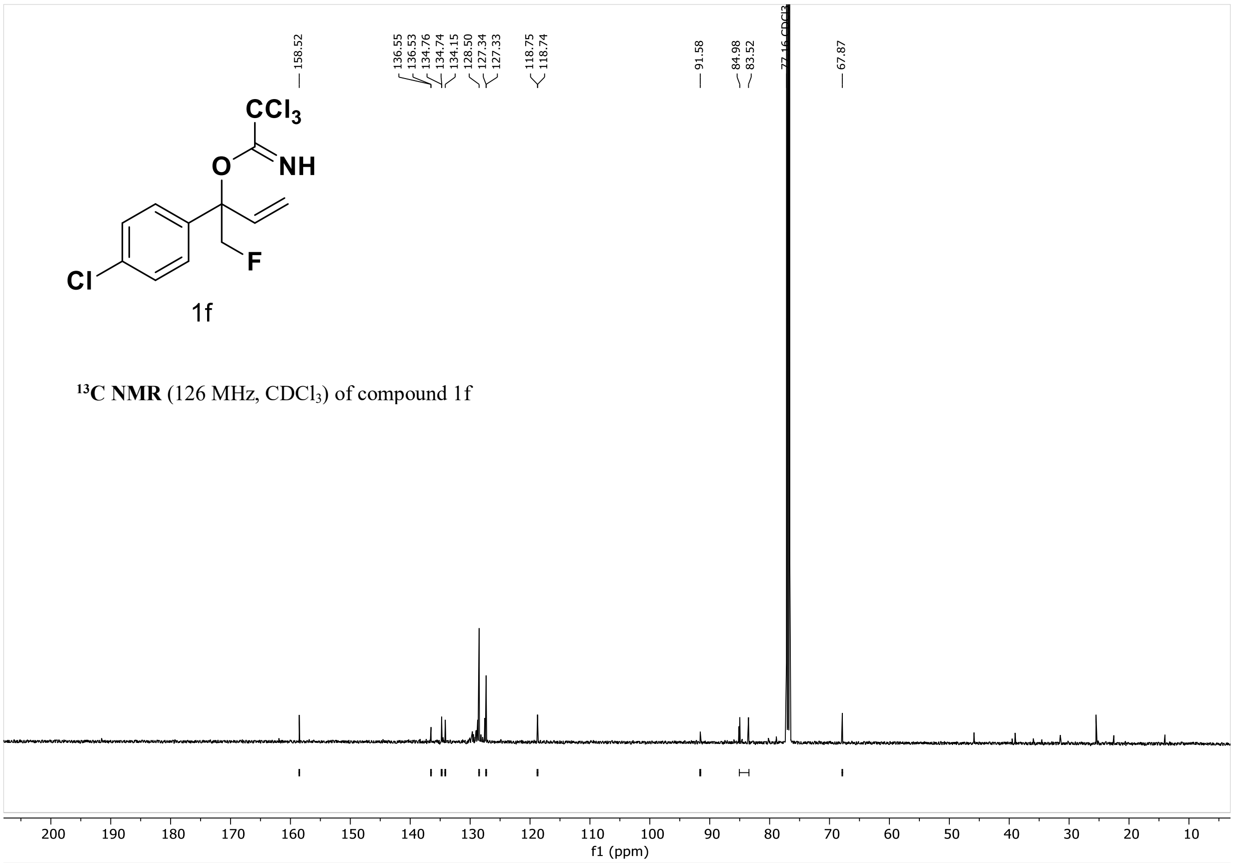

^{13}**C NMR** (126 MHz, $CDCl_3$) of compound 1f

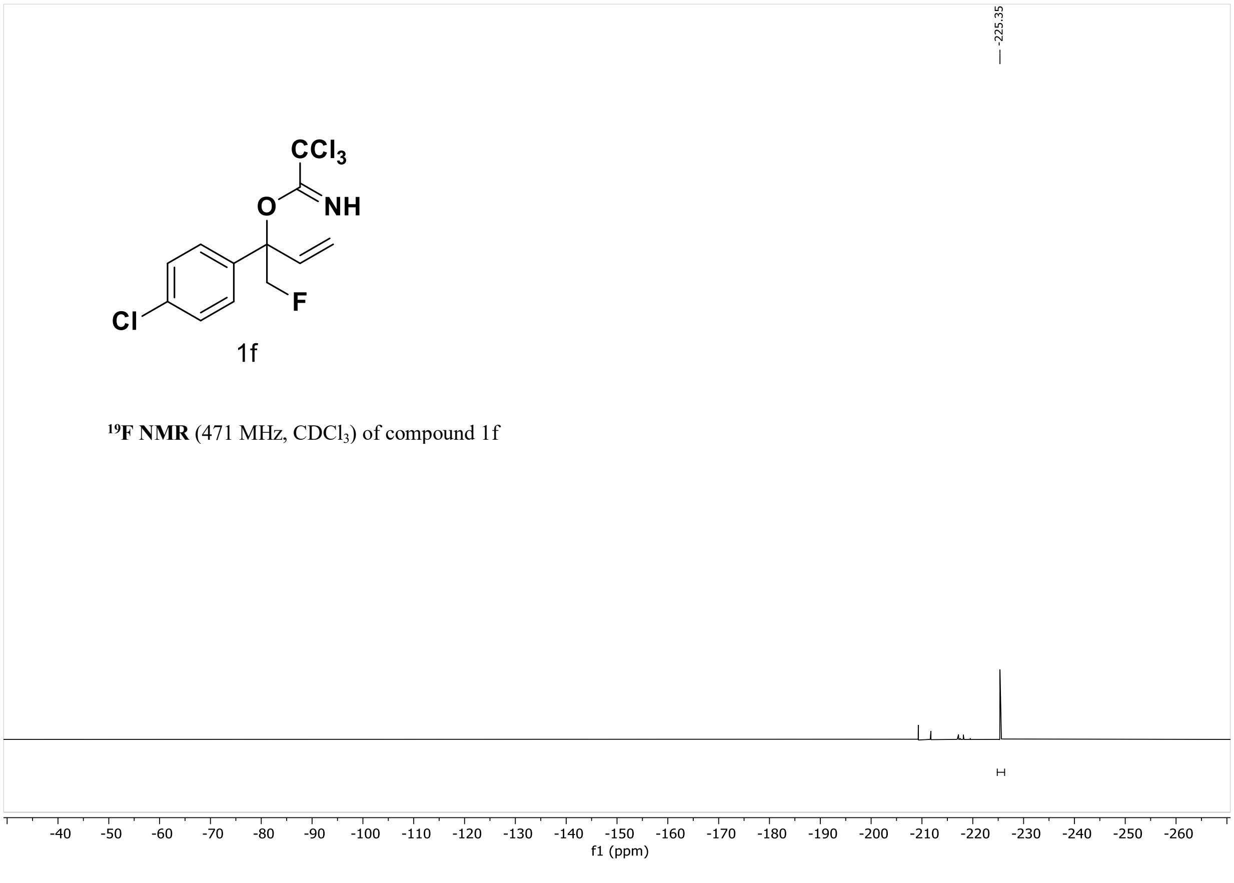

^{19}**F NMR** (471 MHz, $CDCl_3$) of compound 1f

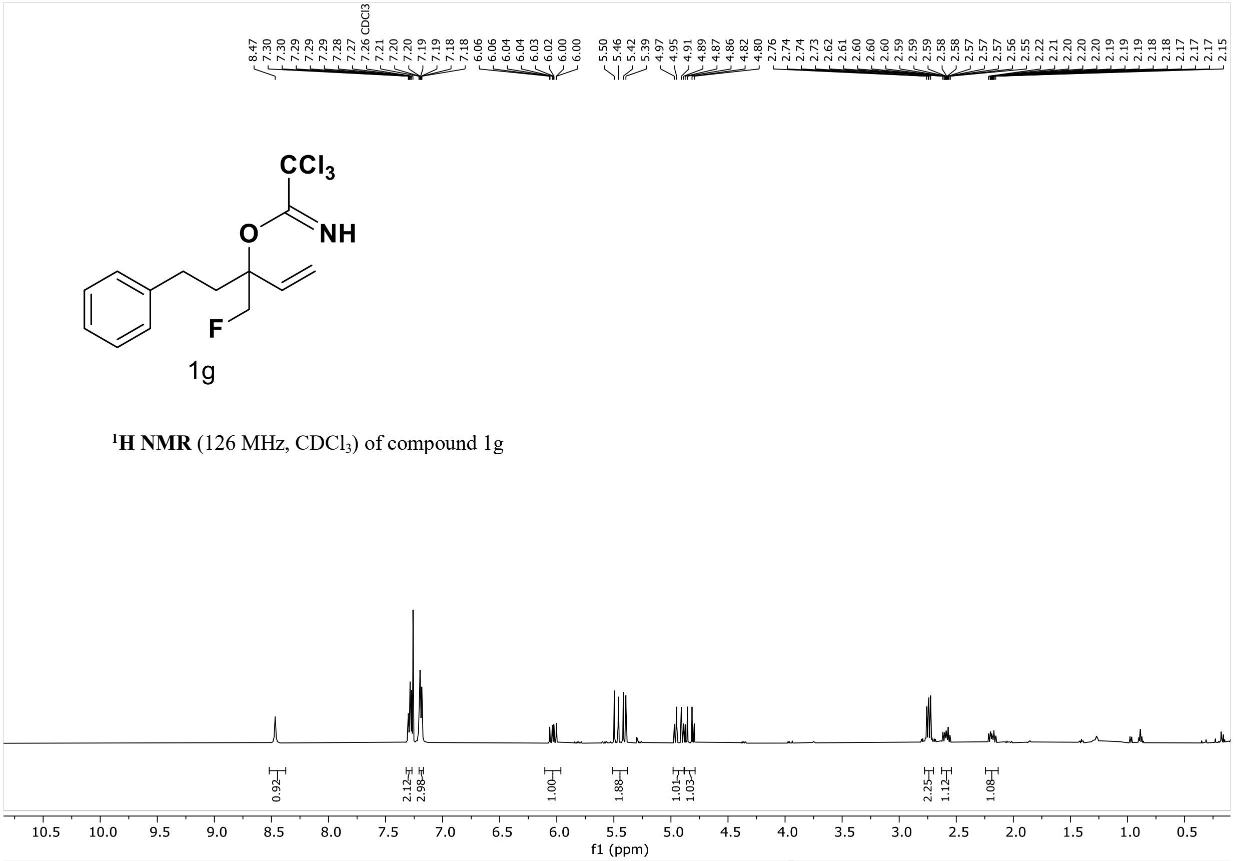

^{1}H NMR (126 MHz, $CDCl_3$) of compound 1g

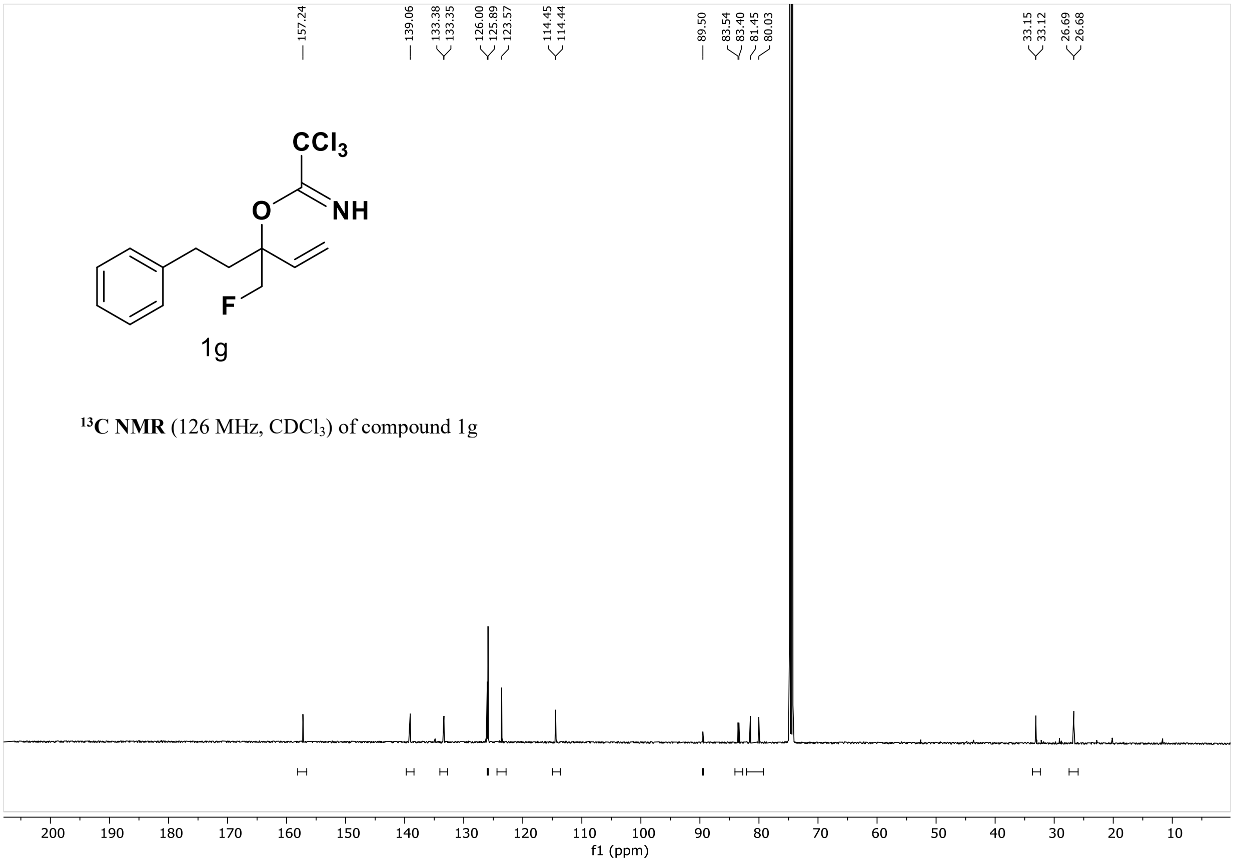

^{13}C NMR (126 MHz, $CDCl_3$) of compound 1g

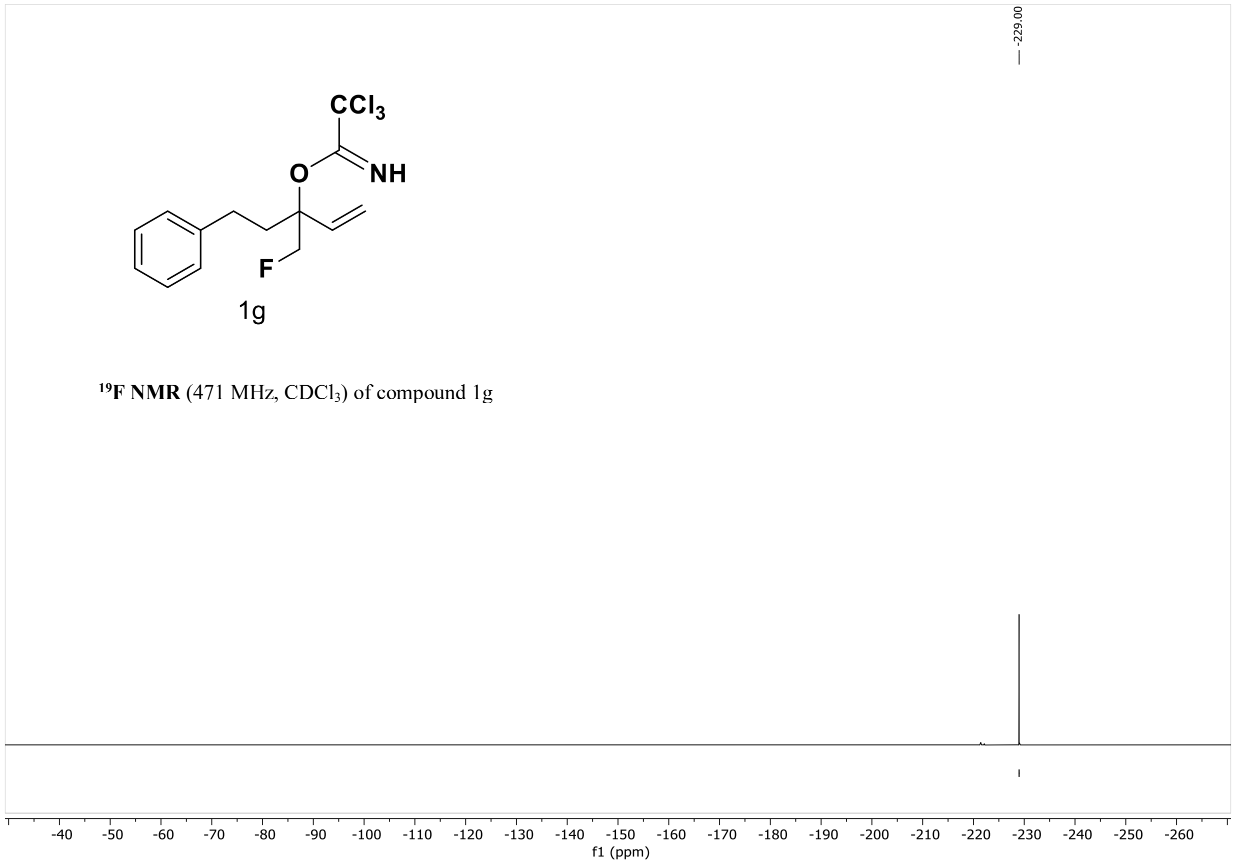

^{19}F NMR (471 MHz, $CDCl_3$) of compound 1g
